# Boundedness and Decay for the Teukolsky Equation on Kerr in the Full Subextremal Range |a|<M: Frequency Space Analysis

**DOI:** 10.1007/s00205-026-02173-9

**Published:** 2026-08-21

**Authors:** Yakov Shlapentokh-Rothman, Rita Teixeira da Costa

**Affiliations:** 1 Department of Mathematics, University of Toronto, 40 St. George Street, Toronto, ON Canada; 2 Department of Mathematical and Computational Sciences, University of Toronto Mississauga, 3359 Mississauga Road, Mississauga, ON Canada; 3 Department of Mathematics, Princeton University, Washington Road, Princeton, NJ 08544 USA; 4 Princeton Gravity Initiative, Princeton University, Jadwin Hall, Washington Road, Princeton, NJ 08544 USA; 5 Department of Pure Mathematics and Mathematical Statistics, University of Cambridge, Wilberforce Road, Cambridge, CB3 0WA UK

## Abstract

In this paper, and in the companion ([152]), we study the gauge-invariant components of linear perturbations of Kerr black hole spacetimes in the full subextremal range of parameters |a|<M. We show that, for all |a|<M, solutions of the Teukolsky equation of spin ±1 and spin ±2 arising from regular initial data remain bounded and decay in time. Our result makes completely explicit the dependence of these bounds on the Kerr black hole parameters, while being entirely self-contained. For completeness, we also include the case of the scalar wave equation, s=0. The present paper, which is an abridged version of our preprint ([151]), considers fixed frequency solutions of the transformed system of equations introduced by Dafermos et al. (Acta Math 222(1):1–214, 2019). We obtain estimates which are uniform in the separation parameters for subextremal |a|<M, as well as estimates away from the superradiant frequency threshold up to and including the extremal |a|=M. In the subextremal |a|<M case, these estimates are summed in our companion paper ([152]) to yield the conclusion of the proof of our main result.

## Introduction

The Einstein vacuum equations

1.1 $$\begin{aligned} \textrm{Ric}(g)=0 \end{aligned}$$

to be satisfied by a 4-dimensional Lorentzian manifold $(\mathcal {M},g)$ representing spacetime are the system of geometric partial differential equations which lie at the core of the theory of General Relativity.

Aside from the flat Minkowski space, several other explicit solutions of (1.1) are known, most notably the Kerr family of black holes [105]. Kerr black holes are axisymmetric, asymptotically flat spacetimes parametrized by a mass, M>0, and specific angular momentum, *a*, satisfying the bound |a|≦M. We say these are subextremal if |a|<M, with the spherically symmetric case a=0 being known as the Schwarzschild solution [145], and extremal if |a|=M.

Subextremal Kerr black holes have been shown to be isolated and, under stronger assumptions, unique in the class of regular stationary, asymptotically flat black hole solutions to (1.1) (see [13], the review [29] and references therein); thus, they play a privileged role in our understanding of vacuum General Relativity. For this reason, a great deal of literature on General Relativity is centered around the question

*What are the dynamics of perturbations of Kerr under the Einstein vacuum equations* (1.1)?

Indeed, the Einstein vacuum equations (1.1) can be seen as evolution equations for given data: they are well-posed for sufficiently regular data given on a Cauchy hypersurface (see [28, 65, 143]) or on a characteristic hypersurface (see [116, 141]). Thus, we can realize the previous question in the following more concrete problem, known as the black hole stability problem:

### Question I

*(Nonlinear stability)* In evolution under the Einstein vacuum equations (1.1), do initial data sets sufficiently close to that corresponding to a Kerr black hole give rise to a spacetime whose exterior

- remains uniformly close to said member of the Kerr family (orbital stability),
- and asymptotically approaches a Kerr solution with nearby mass and specific angular momentum (asymptotic stability)?

The answer to this question is thought to be positive, at least in the subextremal |a|<M case. In a remarkable series of recent works, two groups have shown that this holds under the restriction that a=0 or |a|≪M. Dafermos, Holzegel, Rodnianski and Taylor showed in [49] the stability of the Schwarzschild (a=0) subfamily in full finite codimension, that is for initial data lying in the full codimension 3 set of vacuum data (identified in evolution) whose hyperbolic development under (1.1) asymptotes towards Schwarzschild to the future; see also the recent [50] for partial results in the |a|≪M setting. Klainerman and Szeftel, who had already obtained in [107] the nonlinearly stability of the Schwarzschild subfamily under the special class of polarized axisymmetric perturbations of Cauchy data, then showed in [110] and jointly with Giorgi in [80] that the very slowly rotating (|a|≪M) Kerr subfamily is nonlinearly stable; see also the companion papers by these authors [79, 108, 109] and by Shen [146]. Prior to these works, stability for general perturbations of vacuum data for (1.1) had only been shown to hold for the Minkowski solution, first by the monumental work of Christodoulou and Klainerman [40] and then by an alternative method in [111]; see also [24, 93, 97, 104] for further developments along the lines of these two approaches.

In order to understand nonlinear stability, one must first understand *linear* stability, to that is the version of Question I where (1.1) are replaced by their linearization around the Kerr family. Indeed, the results mentioned above were preceded by a number of different works regarding linear stability for the |a|≪M subfamily. A seminal result by Dafermos, Holzegel and Rodnianski [48] showed that that the Schwarzschild (a=0) subfamily is linearly orbitaly stable, and linearly asymptotically stable within the |a|≪M Kerr family; their result has since been revisited by a number of authors [23, 85, 86, 100]. More recently, works by Andersson, Blue, Bäckdahl and Ma [9, 10, 119] as well as Häfner, Hintz and Vasy [82] have shown the decay of linear perturbations to the very slowly rotating Kerr (|a|≪M) subfamily.^1^

Question I and its linear version are more easily understood in the language of PDE, but to make (1.1) and its linearization into a concrete set of well-posed PDE there are two necessary ingredients. First, one must decide what should be their unknown: in line with the Newman–Penrose formalism [133] in the physics literature, we will consider perturbations in Question I to be defined at the level of the Riemann curvature and connection components expressed in terms of a null frame. Second, we need a particular choice of *gauge* to break the diffeomorphism invariance of these equations.

A choice of gauge is not required to analyze every single equation in the system of linearized (1.1) around Kerr spacetimes, as some quantities do not depend on the gauge at all. In fact, there is a particular choice of null frame, called the *algebraically special* frame, for which two components of the Riemann curvature tensor, known as *extreme* components $\upalpha ^{[\pm 2]}$, that vanish for Kerr spacetimes, are easily seen to be *linearly* gauge-invariant^2^ for their perturbations. These quantities actually completely characterize *all* the linearly gauge-invariant degrees of freedom, in the sense that all solutions to the linearized (1.1) for which the extreme curvature components vanish are, up to gauge, merely linearized Kerr spacetimes [164]. In fact, one expects that the extreme curvature components should also control the gauge *dependent* quantities: a number of different authors have proposed schemes to accomplish this, from [36] to the aforementioned [9, 48, 79], and the more recent [21, 22] to which we refer for comments on the regularity of such schemes.

In the linear version of Question I, the extreme curvature components can, thus, be seen as the geometric degrees of freedom of gravitational perturbations of Kerr spacetimes. Remarkably, Teukolsky [157] discovered that their linear evolution fully *decouples* from the remaining degrees of freedom! They satisfy the so-called Teukolsky equations

1.2 $$\begin{aligned} \begin{aligned} \bigg [\Box _g&+\frac{2s}{\rho ^2}(r-M)\partial _r +\frac{2s}{\rho ^2}\left(\frac{a(r-M)}{\Delta }+i\frac{\cos \theta }{\sin ^2\theta }\right)\partial _\phi \\&+\frac{2s}{\rho ^2}\left(\frac{M(r^2-a^2)}{\Delta }-r -ia\cos \theta \right)\partial _t +\frac{1}{\rho ^2}\left(s-s^2\cot ^2\theta \right)\bigg ]\upalpha ^{[s]} =0\,, \end{aligned} \end{aligned}$$

with s=±2. Here, $\Box _g$ is the covariant scalar wave operator on the Kerr spacetime, and we have used Boyer–Lindquist coordinates $(t,r,\theta ,\phi )\in \mathbb {R}\times (r_+,\infty )\times \mathbb {S}^2$ and the notation

$$\begin{aligned} \Delta :=(r-r_+)(r-r_-)\,, \qquad r_\pm :=M\pm \sqrt{M^2-a^2}\,, \qquad \rho ^2=r^2+a^2\cos ^2\theta \,. \end{aligned}$$

In (1.2), the unknown $\upalpha ^{[{s}]}$ is an *s*-spin weighted function (see Definition 3.1), which can be defined for any $s\in \frac{1}{2} \mathbb {Z}$. The case s=0, corresponding to the scalar wave equation on the Kerr background, has been studied in a number of works (see already Section 1.5) and was finally understood in the full subextremal |a|<M range of Kerr parameters in the work of the first author with Dafermos and Rodnianski [57, 58]. Arguably, it is the case s=±1 which more closely resembles the s=±2 case discussed above: in the former setting, (1.2) describes the dynamics of linearly gauge-invariant components of the Maxwell tensor for electromagnetic perturbations of Kerr. The analysis of the Teukolsky equation (1.2) for $s=\pm 1,\pm 2$ was previously carried out for the Schwarzschild (a=0) and very slowly rotating Kerr (|a|≪M) subfamily in [47, 48, 80, 118, 119, 137], and was crucial to the aforementioned (non)linear stability results in this restricted class of Kerr spacetimes (see also [25]).

In this paper, and in its companion [152], we obtain for the first time quantitative results for the Teukolsky equation (1.2) with $s=\pm 1,\pm 2$ in the entire subextremal |a|<M range of Kerr parameters.

### Main Theorem

Fix $s\in \{\pm 1,\pm 2\}$, M>0 and |a|<M. On the exterior of a Kerr black hole spacetime with parameters (*a*, *M*), general solutions to the Teukolsky equation (1.2) arising from sufficiently regular initial data on a Cauchy surface ($\{\tau =0\}$)

(i) have uniformly bounded energy fluxes through a suitable spacelike foliation of the black hole exterior, through the future event horizon, $\mathcal {H}^+$, and through future null infinity, $\mathcal {I}^+$, in terms of an energy flux of initial data at the same level of regularity
  $$\begin{aligned} {E}_p(\tau _2)\leqq B(a,M,s) {E}_p(\tau _1)\,, \qquad \forall \tau _2\geqq \tau _1\geqq 0\,, \end{aligned}$$
  where $E_p$, for p∈[0,2) denotes a ($r^p$-)weighted time translation invariant Sobolev energy norm on the leaves of a suitable spacelike foliation of the black hole exterior, up to and including $\mathcal {H}^+$;
(ii) satisfy a suitable version of “integrated local energy decay” with loss of derivatives at trapping
  $$\begin{aligned} {I}^\textrm{deg}_p(\tau _1,\tau _2)\leqq B(a,M,s) {E}_p(\tau _1)\,, \qquad \forall \tau _2\geqq \tau _1\geqq 0\,, \end{aligned}$$
  where ${I}^\textrm{deg}_p$, for p∈[0,2) denotes a ($r^p$-)weighted time translation invariant Sobolev energy norm, which is (at trapping) one order of differentiability below $E_p$, on the time slab between two leaves of the aforementioned foliation of the black hole exterior;
(iii) and satisfy similar statements for higher-order energies.

These statements are quantitative, in the sense that the bounds are explicit in terms of *a*, *M* and *s*.

As an immediate corollary of our Main Theorem, we settle the gauge-invariant aspect of the linearized version of Question I and its electromagnetic counterpart

### Main Corollary

Consider the setting of Main Theorem. On any subextremal Kerr black hole, solutions to the Teukolsky equation (1.2) with $s\in \{\pm 1,\pm 2\}$

- remain uniformly bounded, both pointwise and in energy;
- asymptotically decay to 0, both pointwise and in energy, at a suitable inverse polynomial rate.

These statements are quantitative, in the sense that the bounds depend explicitly on the Kerr parameters, *s*, and a suitable initial data norm.

We defer to our companion paper [152] for the precise definitions of the norms and foliation alluded to in the above results. In non-technical terms, the reader can think of $E_p$ as an *r*-weighted $H^{|s|+1}$ Sobolev norm on constant (hyperboloidal) time leaves, and $I^\textrm{deg}_p$, up to differences in *r*-weights, as a spacetime $H^{|s|}$ Sobolev norm in the slab between leaves. In particular, our Main Theorem yields control of the full Sobolev norms of the Teukolsky variables $\upalpha ^{[{s}]}$, cf. [48]. Furthermore, the *r*-weights in our norms in Main Theorem and Main Corollary do not assume so-called “peeling” decay of the data, which is considered to be unphysical, see [37, 101–103] and references therein.

We emphasize the fact that our energy boundedness statement, in our Main Theorem and part (a) of our Main Corollary, does not lose derivatives and thus can be seen as a true orbital stability result for the zero solution to (1.2). The asymptotic stability result in part (b) of our Main Corollary, which follows from Main Theorem by the $r^p$ method of Dafermos and Rodnianski [53, 125], naturally loses derivatives and is not expected to provide a sharp decay rate (see Ma–Zhang [132] and Millet [123] for sharp decay statements, albeit assuming “peeling” decay of initial data).^3^ Nevertheless, we expect that our (decay) results are sufficient for nonlinear applications, see the recent [50] and references therein. Thus, our results pave the way for a positive resolution of (the linearization of) Question I in the full subextremal range |a|<M.

Let us now turn to a brief overview of the proof of our Main Theorem. As we have mentioned, an analogue of our results in the case s=0 of (1.2) has been obtained in [57].^4^ Though the principal symbol of the Teukolsky equation (1.2) is the same in the case of s≠0, the additional $\partial _t$ derivatives preclude a direct application of the the scalar wave analysis.

The additional first order terms in (1.2) for s≠0, when compared to s=0, are present already for the Schwarzschild a=0 subfamily. To address these difficulties, Dafermos, Holzegel and Rodnianski introduced in [47, 48] a transformed system, inspired by [33] (see already Section 1.1), which has since been used and adapted by several other authors [80, 118, 119, 137]. For $s\in \mathbb {Z}$ and $0\leqq k\leqq |s|$, we let $\uppsi ^{[{s}]}_{(k)}$ be obtained by taking *k* appropriately *r*-weighted null derivatives of $\upalpha ^{[{s}]}$, to that is

1.3 $$\begin{aligned} \uppsi _{(0)}^{[s]}=j_{s,|s|}^{-1}(r)\upalpha ^{[{s}]}\,;\qquad \uppsi ^{[{s}]}_{(k+1)} = j_{s,k}^{-1}(r)\mathcal {L} \uppsi _{(k)}^{[s]}\,,\,\, k=0,...,|s|-1\,, \end{aligned}$$

where $\mathcal {L}$ denotes null vector fields. It turns out that a judicious choice of *r*-weights $j_{s,k}$ leads to the elimination of any $\partial _t$ first order terms in the evolution equation for the k=|s| level transformed variable: $\Psi ^{[{s}]}=\uppsi ^{[{s}]}_{(|s|)}$ satisfies

1.4 $$\begin{aligned} \mathfrak {R}^{[{s}]} \Psi ^{[{s}]} = a \sum _{k=0}^{|s|-1}\mathfrak {J}^{[{s}]}_{(k)}\hspace{-2pt}\left(\uppsi ^{[{s}]}_{(k)}, \partial _\phi \uppsi ^{[{s}]}_{(k)} \right), \end{aligned}$$

where $\mathfrak {J}^{[{s}]}_{(k)}$, k=0,⋯,|s| are *r*-dependent linear operators which can be made explicit and $\mathfrak {R}^{[{s}]}$ behaves like a wave operator for spin-weighted, rather than scalar, functions. For a=0, the latter is sometimes called the Regge–Wheeler operator when s=±2 and the Fakerell–Ipser operator when s=±1. In the aforementioned works, which pertain to the |a|≪M setting, the above transformation allowed for a (almost) direct proof of boundedness and decay of $\Psi ^{[{s}]}$, through the techniques developed for the scalar wave equation since the 2000s (see for instance the lecture notes [56]), with the boundedness and decay of $\upalpha ^{[{s}]}$ then following by integration of (1.3).

In the general subextremal case |a|<M, where the right hand side of (1.4) is *not* small, a simple but crucial insight of our work is that the wave equations satisfied by each of the transformed variables (not just the top level one), though undesirable on their own, can be cast as a hierarchical wave system with a good structure. Indeed, for k=0,⋯,|s|-1, we have

$$\begin{aligned} \tilde{\mathfrak {R}}^{[{s}]}_{(k)} \uppsi ^{[{s}]}_{(k)} = \sum _{j=0}^{k-1}\mathfrak {J}^{[{s}]}_{(k),(j)}\hspace{-2pt}\left(\uppsi ^{[{s}]}_{(j)}, \partial _\phi \uppsi ^{[{s}]}_{(j)} \right), \end{aligned}$$

where the differential operators $\tilde{\mathfrak {R}}^{[{s}]}_{(k)}$, like the s≠0 Teukolsky operator, contain first order derivatives with possibly anti-damping behavior; however, using (1.3), these equations can be rewritten as

1.5 $$\begin{aligned} \mathfrak {R}^{[{s}]}_{(k)} \uppsi ^{[{s}]}_{(k)} = (|s|-k)W^{[{s}]}_{(k)} \uppsi ^{[{s}]}_{(k+1)}+\sum _{j=0}^{k-1}\mathfrak {J}^{[{s}]}_{(k),(j)}\hspace{-2pt}\left(\uppsi ^{[{s}]}_{(j)}, \partial _\phi \uppsi ^{[{s}]}_{(j)} \right), \end{aligned}$$

where $\mathfrak {R}^{[{s}]}_{(k)}$ no longer contain additional $\partial _t$ when compared with $\Box _g$, $\mathfrak {J}^{[{s}]}_{(k),(j)}$ is as before but for $0\leqq j<k\leqq |s|$, and $W^{[{s}]}_{(k)}(r)$ is a function of *r* which can be made explicit (and is not *a*-small). In (1.5) we have recast the bad $\partial _t$ terms of the Teukolsky equation and $\tilde{\mathfrak {R}}^{[{s}]}_{(k)}$ operator as a coupling term to the next level transformed variable. Thus, the *k*th level equation is coupled to the *j*th level, j<k, unless k=0 and to the (k+1)th unless k=|s|; for example for |s|=2, we haveIn our analysis, we will replace the hyperbolic equation (1.2) by the *transformed system* composed of the hyperbolic equations (1.4) and (1.5) together with the transport equations (1.3). To see how, in practice, the structure described is leveraged already in the present paper, the reader may wish to jump ahead to Section 1.3.3 and 1.3.4.

In our analysis of the transformed system (1.3)–(1.5), we will apply a mix of physical and frequency space methods. As understood by Carter [27] for s=0 and generalized by Teukolsky [157] for $s\in \frac{1}{2}\mathbb {Z}$, the Teukolsky equation admits separable solutions and, hence, so does the aforementioned transformed system. For the Teukolsky equation (1.2), these solutions take the form

1.6 $$\begin{aligned} \upalpha ^{[s],\,a,\,\omega }_{m\Lambda }(t,r,\theta ,\phi ) = e^{-i\omega t}e^{im\phi }S^{[s],\,a\omega }_{m\Lambda }(\theta )(r^2+a^2)^{\frac{1}{2}}\Delta ^{\frac{s}{2}}u_{m\Lambda }^{[{s}],\,a,\,\omega }(r) \end{aligned}$$

in Boyer–Lindquist coordinates, where $S^{[s],\,a\omega }_{m\Lambda }(\theta )$ are spin-weighted spheroidal harmonics and Λ are spin-weighted spheroidal eigenvalues. Here, $u_{m\Lambda }^{[{s}],\,a,\,\omega }$ solves the Teukolsky radial ODE

1.7 $$\begin{aligned} \frac{\Delta }{r^2+a^2}\frac{\textrm{d}}{{\textrm{d}}r}\left[\frac{\Delta }{r^2+a^2}\frac{\textrm{d}}{{\textrm{d}}r}u_{m\Lambda }^{[{s}],\,a,\,\omega }\right]+\left(\omega ^2-{V}_{m\Lambda }^{[{s}],\,a,\,\omega }(r)\right)u_{m\Lambda }^{[{s}],\,a,\,\omega } = H_{m\Lambda }^{[{s}],\,a,\,\omega },\nonumber \\ \end{aligned}$$

for an explicit potential ${V}_{m\Lambda }^{[{s}],\,a,\,\omega }(r)$ and vanishing inhomogeneity, $H_{m\Lambda }^{[{s}],\,a,\,\omega }\equiv 0$. To ensure that (1.6) has (qualitatively) finite energy along a suitable spacelike hypersurface up to and including the future event horizon, we further require that $u_{m\Lambda }^{[{s}],\,a,\,\omega }$ have so-called *outgoing boundary conditions*, see already Definition 4.5.

If we knew *a priori* that general solutions to (1.2) admit a Fourier transform in the variable *t*, then the ansatz (1.6) is much more than an ansatz: general solutions could then be expressed as an infinite superposition of modes of the form (1.6) with $(\omega ,m,\Lambda )\in \mathbb {R}$. This is a consequence of the completeness of the spin-weighted spheroidal harmonics and of Plancherel’s theorem.^5^ It follows that any *r*-weighted $L^2$ estimates for the radial ODE (1.7) which are uniform in the *real* frequency parameters $(\omega ,m,\Lambda )$ immediately imply analogous estimates for the PDE (1.2). A similar statement can be made for our transformed system (1.3)–(1.5): assuming enough integrability in *t*, the study of these PDEs can be essentially reduced to the study of the corresponding radial ODEs. We defer to Section 1.4 for the precise details of this reduction, which is carried out in detail in out companion paper [152].

Let us remark already that, in practice, to meet the integrability-in-time assumption of the first paragraph, and also to remove it later (see Section 1.4), it will be convenient to apply cutoffs to the PDEs and, therefore, work with inhomogeneous versions of the PDEs and corresponding radial ODEs. Thus, in general, $H_{m\Lambda }^{[{s}],\,a,\,\omega }\not \equiv 0$. In fact, the first step in any frequency analysis is of the Teukolsky equation (1.2) is to establish *mode stability*, to that is to show that $H_{m\Lambda }^{[{s}],\,a,\,\omega }\equiv 0$ will imply $u_{m\Lambda }^{[{s}],\,a,\,\omega }\equiv 0$, within the class of outgoing solutions.^6^ To phrase this result in a more concrete way, denote by $u^{[{s}]}_{\mathcal {H}^+}$ (resp. $u^{[{s}]}_{\mathcal {I}^+ }$) the radial components of separable solutions of the *homogeneous* Teukolsky equation (1.2) which are characterized by some real $(\omega ,m,\Lambda )$ and which are regular, with respect to the algebraically special frame in which the Teukolsky variable is defined, at the future event horizon (resp. future null infinity) of Kerr. We define the Wronskian

$$\begin{aligned} \mathfrak {W}^{[{s}]}(\omega ,m,\Lambda ):=\frac{\Delta }{r^2+a^2}\left[\frac{\textrm{d}}{{\textrm{d}}r}\left(u^{[{s}]}_{\mathcal {I}^+}\right)\cdot u^{[{s}]}_{\mathcal {H}^+}-\frac{\textrm{d}}{\textrm{d}r}\left(u^{[{s}]}_{\mathcal {H}^+}\right)\cdot u^{[{s}]}_{\mathcal {I}^+}\right]\,. \end{aligned}$$

Mode stability states that $\mathfrak {W}^{[{s}]}\ne 0$ for every $(\omega ,m,\Lambda )$ with $\omega \ne 0$; more precisely, we have

### Theorem 0

Fix $s\in \frac{1}{2}\mathbb {Z}$, M>0 and |a|<M. For each set of real frequency parameters $\mathcal {A}$ such that $C_{\mathcal {A}}:=\sup _{\mathcal {A}} \left(|\omega |+|\omega |^{-1}+|m|+|\Lambda |\right)<\infty$, there is a constant $B(a,M,|s|,C_{\mathcal {A}})>0$ such that

1.8 $$\begin{aligned} \left|\mathfrak {W}^{[{s}]}\right|^{-1}\leqq B(a,M,|s|,C_{\mathcal {A}})<\infty \text { for }(\omega ,m,\Lambda )\in \mathcal {A}. \end{aligned}$$

It follows that, for suitable $H_{m\Lambda }^{[{s}],\,a,\,\omega }(r)$, outgoing solutions of (1.7) satisfy pointwise (and integrated in *r*) estimates in terms of $H_{m\Lambda }^{[{s}],\,a,\,\omega }(r)$ in an $L^2_{\omega }$-sense, see for example [150, 152]. These statements are quantitative, in the sense that the constants in the bounds can be explicitly computed in terms of *a*, *M*, *s* and $C_{\mathcal {A}}$.

The result also holds for |a|=M if $\omega \ne \frac{am}{2Mr_+}$, with a constant *B* depending on $\left|\omega -\frac{am}{2Mr_+}\right|$ as well.

Some comments concerning Theorem 0 are in order. Let us first note that, the conservation law for (1.7) can be used to infer the qualitative conclusion that $\mathfrak {W}^{[{s}]}\ne 0$ in a subset of the space of frequency parameters, but not its entirety unless a=0. Indeed, for $|s|\in \left\rbrace 0, 1, 2\right\lbrace$, *superradiance*, to that is

$$\begin{aligned} \omega \left(\omega -\frac{am}{2Mr_+}\right)<0\,, \end{aligned}$$

leads to a breakdown in coercivity for the conservation law, preventing one from inferring mode stability directly. The lack of coercivity in the case of |a|≪M can be cured by making use, for instance, of the redshift effect associated to the nondegenerate event horizon [53]. That the Teukolsky equation (1.2) remains modally stable for the entire subextremal |a|<M [12, 15, 44, 150, 155, 166] and even for extremal |a|=M [155] Kerr family is quite surprising. Indeed, this property seems very specific to the algebraic structure of the Kerr metric and the Teukolsky equation (1.2), see [126, 149]. Though the aforementioned result [166] due to Whiting was the first to uncover the qualitative mode stability of (1.2), the quantitative bounds (1.8) on $\mathfrak {W}^{[{s}]}$ are due to the two authors [150, 155, 156]^7^, and the consequences sketched for the inhomogeneous (1.7) are proven in [150] for s=0 and in our companion [152] for general $s\in \mathbb {Z}$, to which we defer a precise formulation.

Theorem 0 covers an arbitrarily large region of the $(\omega ,m,\Lambda )$ parameter space, but one which is necessarily finite: the constants in the bounds grow uncontrollably as $|\omega |+|m|+|\Lambda |\rightarrow \infty$ or $\omega \rightarrow 0$. In understanding these two regimes, it is useful to consider again the transformed system (1.3)–(1.5); more precisely, we consider inhomogeneous versions of the associated radial ODEs. For instance, by abuse of notation writing $\uppsi ^{[{s}]}_{(k)}$ and $\Psi ^{[{s}]}$ for their radial components, the radial ODE associated to the latter takes the form

1.9 $$\begin{aligned}  &   \frac{\Delta }{r^2+a^2}\frac{\textrm{d}}{\textrm{d}r}\left[\frac{\Delta }{r^2+a^2}\frac{\textrm{d}}{\textrm{d}r}\Psi ^{[{s}]}\right]+\left(\omega ^2-\mathcal {V}_{m\Lambda }^{[{s}],\,a,\,\omega }(r)\right)\Psi ^{[{s}]} \nonumber \\  &   \quad = a \sum _{k=0}^{|s|-1}\mathfrak {J}^{[{s}]}_{(k)}\hspace{-2pt}\left(\uppsi ^{[{s}]}_{(k)}, im \uppsi ^{[{s}]}_{(k)} \right)+\mathfrak G^{[{s}]}, \end{aligned}$$

for an explicit potential $\mathcal {V}_{m\Lambda }^{[{s}],\,a,\,\omega }(r)$ and having added an inhomogeneity $\mathfrak G^{[{s}]}$. We say that solutions to such transformed radial ODEs have outgoing boundary conditions if they arise, via (1.3), from outgoing solutions to the Teukolsky radial ODE (1.2). Our main result is

### Theorem 1

Fix $s\in \{0,\pm 1,\pm 2\}$, M>0 and |a|<M. There exists a set $\mathcal {A}$ of real frequency parameters with $C_{\mathcal {A}}:=\sup _{\mathcal {A}} \left(|\omega |+|\omega |^{-1}+|m|+|\Lambda |\right)<\infty$ and some constants B=B(a,M,|s|) such that we have the following sets of estimates uniformly in $(\omega ,m,\Lambda )\notin \mathcal {A}$.

(i) Boundary term estimates: for outgoing solutions to (1.9),
  1.10 $$\begin{aligned} \begin{aligned}&\left(\omega -\frac{am}{2Mr_+}\right)^2\left|\Psi ^{[{s}]}\right|^2_{r=r_+}+\omega ^2\left|\Psi ^{[{s}]}\right|^2_{r=\infty } \\&\leqq B(a,M,s) \sum _{k=0}^{|s|}\int _{r_+}^\infty \mathfrak {G}^{[{s}]}_{(k)}\cdot \left(\uppsi ^{[{s}]}_{(k)},\partial _r\uppsi ^{[{s}]}_{(k)}\right) \textrm{d}r. \end{aligned} \end{aligned}$$
(ii) Integrated in *r* estimates: for outgoing solutions to (1.9), there is a value $r_\textrm{trap}(\omega ,m,\Lambda )$ which is either 0 or satisfies the uniform bounds $|r_\textrm{trap}|+|r_\textrm{trap}-r_+|^{-1}\leqq B(a,M,s)$ such that
  1.11 $$\begin{aligned}  &   \int _{r_+}^\infty \frac{\Delta }{r^4}\left\rbrace \left|\frac{\Delta }{r^2}\partial _r\Psi ^{[{s}]}\right|^2\right.\nonumber \\  &   \quad \left. \!+\!\left[\left(\omega ^2\!+\!\frac{r\!-\!M}{r^2}\Lambda \right)\left(1\!-\!\frac{r_\textrm{trap}(\omega ,m,\Lambda )}{r}\right)^2\!+r^{-1}(s^2\!+\!r^{-1})\right] \left|\Psi ^{[{s}]}\right|^2\right\lbrace \textrm{d}r \nonumber \\  &   \quad \leqq B(a,M,s) \sum _{k=0}^{|s|} \int _{r_+}^\infty \mathfrak {G}^{[{s}]}_{(k)}\cdot \left(\uppsi ^{[{s}]}_{(k)},\partial _r\uppsi ^{[{s}]}_{(k)}\right) \textrm{d}r. \end{aligned}$$

Here, $\mathfrak G^{[{s}]}_{(k)}$ is an inhomogeneity in the radial ODE for $\uppsi ^{[{s}]}_{(k)}$ and $\mathfrak {G}^{[{s}]}_{(k)}\cdot \left(\uppsi ^{[{s}]}_{(k)},\partial _r\uppsi ^{[{s}]}_{(k)}\right)$ is a nontrivial, $(\omega ,m,\Lambda )$-dependent, function of the inhomogeneity and radial part of the transformed variable, see already Theorem 6.3. These statements are quantitative, in the sense that the constants in the bounds can be explicitly computed in terms of *a*, *M* and *s*.

The result also holds for |a|=M except for $\omega \approx \frac{am}{2Mr_+}$; see already Theorem 6.3 and Remark 6.6 for the precise statement in this case.

By combining Theorem 1 with Theorem 0, we obtain uniform in frequency estimates for the Teukolsky and transformed radial ODEs which are compatible with our Main Theorem: in fact, (1.10) should be seen as a frequency space version of boundedness of energy fluxes through the future event horizon and future null infinity, while (1.11) is the frequency space version of an integrated local energy decay statement. Thus, Theorem 1 captures the rich structure of electromagnetic and gravitational waves on Kerr black holes, from superradiance to trapping and to their (lack of) interaction for |a|<M, and it is the heart of the proof of our Main Theorem. We again refer the interested reader again to Section 1.4 and the introduction to [152] for an explanation of the technical aspects of upgrading Theorem 1 to our Main Theorem and Main Corollary in our companion [152].

Let comment on the |a|→M limit of Theorem 1. Already by studying the proof of [57, Proposition 9.1] in the case s=0 and |a|<M, is not hard to see that that the main difficulty in passing from the subextremal |a|<M to the full black hole range |a|≦M is dealing with the double limit $|a|\rightarrow M, m\rightarrow \upomega _+$, and that for all other admissible frequencies identical techniques should yield the desired estimates. In this work, we demonstrate that this is the case not only for s=0 (where the statement is morally contained already in [57]), but for |s|=1,2 as well. Beyond the case of axisymmetric [20] or fixed *m* solutions [70], a quantitative or even qualitative understanding of the limit $|a|\rightarrow M, m\rightarrow \upomega _+$ remains elusive and thought to be quite challenging already for the scalar wave s=0, see Section 1.3.1.

Let us also briefly comment on the the boundary term estimates (1.10) in Theorem 1. The outgoing boundary conditions assumed in the statement are natural for the Cauchy evolution of the Teukolsky equation (1.2) and the transformed system (1.3)–(1.5). In a scattering evolution, to that is where data for the PDEs is posed on the asymptotically far past/future (past/future event horizon and null infinity) and evolved to the asymptotically far future/past, the natural boundary conditions induced on the radial ODEs are more general. Noting that Theorem 0 easily implies boundary term estimates in $\mathcal {A}$, see [155], under such general boundary conditions, an analogue of estimate (1.10) from Theorem 1 for $\mathcal {A}^c$ for general boundary conditions would be the heart of a proof of a scattering theory for the Teukolsky equation (1.2). Indeed, this is arguably the case for the s=0 scattering theory developed by the first author with Dafermos and Rodnianski in [58]. In our preprint [151] on which the present paper is based, we have fleshed out this slightly more involved frequency space analysis. We showed that, letting $\mathfrak {G}_{(k)}^{[{s}]}\equiv 0$, (1.10) for outgoing boundary conditions is replaced, in the case of general boundary conditions, by

1.12 $$\begin{aligned} \begin{aligned}&\omega ^2\frac{\mathfrak {C}_s}{\mathfrak {D}_s^{\mathcal {I}}}\left|A^{[{s}]}_{\mathcal {I}^+}\right|^2+\left(\omega -\frac{am}{2Mr_+}\right)^2\left|A^{[{s}]}_{\mathcal {H}^+}\right|^2\\&\quad \leqq B(a,M,s)\left[\omega ^2\left|A^{[{s}]}_{\mathcal {I}^-}\right|^2+\left(\omega -\frac{am}{2Mr_+}\right)^2\frac{\mathfrak {C}_s}{\mathfrak {D}_s^{\mathcal {H}}}\left|A^{[{s}]}_{\mathcal {H}^-}\right|^2\right]\,,\,\, s\leqq 0,\\&\omega ^2\left|A^{[{s}]}_{\mathcal {I}^+}\right|^2+\left(\omega -\frac{am}{2Mr_+}\right)^2\frac{\mathfrak {C}_s}{\mathfrak {D}_s^{\mathcal {H}}}\left|A^{[{s}]}_{\mathcal {H}^+}\right|^2\\&\quad \leqq B(a,M,s)\left[\omega ^2\frac{\mathfrak {C}_s}{\mathfrak {D}_s^{\mathcal {I}}}\left|A^{[{s}]}_{\mathcal {I}^-}\right|^2+\left(\omega -\frac{am}{2Mr_+}\right)^2\left|A^{[{s}]}_{\mathcal {H}^-}\right|^2\right]\,, \,\, s>0 \end{aligned} \end{aligned}$$

for $s=\pm 1,\pm 2$, where $A^{[{s}]}_{\mathcal {H}^\pm }$ can be interpreted as the amplitude of data for $\Psi ^{[{s}]}$ on the future/past event horizon, $A^{[{s}]}_{\mathcal {I}^\pm }$ can be interpreted similarly for the future/past null infinity, and where the radial Teukolsky–Starobinsky constants $\mathfrak {C}_s$ [106, 154, 159], and the $\mathfrak {D}_s^{\mathcal {I}}$ and $\mathfrak {D}_s^{\mathcal {H}}$ are explicit, real, non-negative constants which depend only on the black hole parameters (*a*, *M*), the spin |*s*|, and the frequency triple $(\omega ,m,\Lambda )$. In view of the properties of $\mathfrak C_s(\omega ,m,\Lambda )$ for high frequencies, see [43], we do not expect (1.12) can be easily extended to the nonphysical spins |s|>2; see our preprint [151] or the thesis [156] for further details. In contrast, in the setting of Theorem 1, the proof given here has a very mild dependence on $\mathfrak C_s(\omega ,m,\Lambda )$ and these are used only in a sector of frequency space where we expect them to be very well-behaved, see Section 1.3.4. Thus, we expect our Theorem 1 to hold more generally for any $s\in \mathbb {Z}$.

The preceding paragraph naturally leads us to a second crucial insight of our work: to make use of the conservation law associated to the Teukolsky equation (1.20) in the separated picture. We refer to this as the Teukolsky–Starobinsky energy, since the constants $\mathfrak C_s(\omega ,m,\Lambda )$ play a key role and the conserved quantity, for |s|=1,2, is coercive for all non-superradiant frequencies. Though the Teukolsky–Starobinsky energy was known already to Teukolsky [159], it was not, to the best of our knowledge, used in any prior works regarding boundedness and/or decay for (1.20). Here it is particularly useful, as it ascends to a conservation law, with similar coercivity properties, for *each* of the transformed variables! That such conserved quantities exist is highly nontrivial: for instance, looking at the top level transformed equation (1.4) it is easy to see that Killing vector field do *not* generate conservation laws unless a=0. In the case a=0, the energies for (1.4) generated by causal Killing vector fields coincide with a Teukolsky–Starobinsky energy.

To sum up, the main challenge we face in establishing Theorem 6.3 (and, indeed, our Main Theorem) is that for general subextremal |a|<M and s≠0, the analysis of the Teukolsky equation (1.2) does not reduce to the analysis of the scalar wave equation in [57] but instead has genuinely new features associated with the s≠0 first order terms in the former equation. Indeed, we were unable to reduce (1.2) to the s=0 case, since by following the transformations of [48] we obtained a transformed equation, (1.4), which clearly couples *strongly*, unless |a|≪M, to the Teukolsky equation (1.2). On the flip side, we are able to tap into the special structure of the additional first order terms in the Teukolsky equation (1.2): namely that they are such that (1.2) admits a conservation law at least in the separated picture; and that they are such that (1.2) can be used to generate a *system* of wave equations with a particular hierarchical structure. We will show that, by applying the scalar wave methods to both the seemingly good hyperbolic equation (1.4) and the seemingly bad hyperbolic equations (1.5) (which include the Teukolsky equation (1.2) as the case k=0), and using the structure equations (1.3) connecting them, we can close estimates for the entire transformed system. The exact gains that we obtain from exploiting the structure of the entire system depend heavily on the frequency regime considered, and are best understood in light of the s=0 techniques. Thus, we direct the reader to Section 1.3 for a detailed explanation.

Let us close with an outline of the remainder of this introduction. In Section 1.1, we review the transformed system introduced above as (1.3)–(1.5) and discuss the motivation for considering it. In Section 1.2 we discuss the separability of the Teukolsky equation (1.2) and give a broad overview of its main features as seen in frequency space. These are then captured in the proof of Theorem 1 of this paper, which we discuss in detail in Section 1.3. Our companion [152] is briefly outlined in Section 1.4, where we emphasize the central role that Theorem 1 plays in the proof of our Main Theorem. We conclude the introduction with a more detailed review of relevant related work in Section 1.5.

### From the Teukolsky equation to a transformed system of PDEs

As the Teukolsky equation (1.2) is a wave-type equation, one is tempted to apply the techniques that have been developed to study the s=0 case, $\Box _g\alpha ^{[{0}]}=0$, on black hole spacetimes in the past decade. However, there are some fundamental differences. For instance, as (1.2) does not arise from a Lagrangian formulation, there is no clear suitably coercive physical space conserved energy when s≠0 (though there is one in the separated picture; see already Section 1.2.2). Moreover, the first order terms present in (1.2) have poor decay in *r* as $r\rightarrow \infty$ and $r\rightarrow r_+$. These novel features for s≠0 represent substantial difficulties in order for us to apply to (1.2) the methods which have been developed over the past two decades to analyze wave equations on black hole spacetimes. A natural question that arises, then, is whether one can substitute the s≠0 Teukolsky equation by another PDE which behaves more like the s=0 case.

#### A transformed system in Schwarzschild (a=0)

Let us begin by reviewing how the difficulties discussed in the previous paragraphs are overcome for a=0. In [48], the authors substitute the s=±2 Teukolsky equation by the equation satisfied by a variable $\Psi ^{[{\pm 2}]}$ which is obtained from $\upalpha ^{[{\pm 2}]}$ by taking two appropriately *r*-weighted derivatives in a suitable null direction. The idea for this goes back to the fixed-frequency work of Chandrasekhar for Schwarzschild [33], who constructed differential transformations (more precisely, Darboux transformations [78]) connecting the Bardeen–Press [26] equation (the Teukolsky equation (1.2) for a=0) and the Regge–Wheeler equation [142] or the Zerilli equation [169] for, respectively, odd and even perturbations of Schwarzschild. Indeed, the equation for $\Psi ^{[{\pm 2}]}$ is known as the Regge–Wheeler equation [142]:1.13Here *L* and $\underline{L}$ are linearly independent null vectors and  is the (±2)-spin-weighted Laplacian (see already Section 3.1). Note that (1.13) is completely decoupled from the Teukolsky equation (1.2), does not involve any first order terms in $\partial _t$ or $\partial _\phi$, cf. (1.4), and the zeroth order terms are of a repulsive nature. The latter property implies that the Regge–Wheeler equation (1.13) behaves even better than the scalar wave equation on Schwarzschild! A similar situation occurs in the s=±1 case: as observed by [137] (from which we have borrowed the notation; note the idea goes back further [35]), $\phi ^{[{\pm 1}]}$, obtained from $\upalpha ^{[{\pm 1}]}$ by taking one appropriately *r*-weighted derivative in a suitable null direction, satisfies a good decoupled wave equation, known as the Fackerell–Ipser [66] equation.

#### A transformed system for general subextremal Kerr (|a|<M)

In the general subextremal |a|<M Kerr case, it would be convenient to obtain a transformation, similarly to the a=0 case, which eliminates the first order terms in (1.2). Several attempts have been made to generalize Chandrasekhar’s transformations for Kerr spacetimes, such as [30, 92, 147]. Following the latter paper, if we assume that a new variable $\Psi ^{[{s}]}$ is again obtained by taking |*s*| *r*-weighted derivatives along a suitable null direction, estimates for $\upalpha ^{[{s}]}$ can be obtained by integrating the transport equations relating $\Psi ^{[{s}]}$ to the Teukolsky variable, as in the Schwarzschild case. Thus, recall, from the preamble to this introduction, (1.3) i.e

$$\begin{aligned} \uppsi ^{[{s}]}_{(0)}=j_{s,0}^{-1}\upalpha ^{[{s}]}\,,\qquad \uppsi ^{[{s}]}_{(k+1)}=j_{s,k}^{-1}\mathcal {L}\uppsi ^{[{s}]}_{(k)}\,, \quad 0\leqq k \leqq |s|-1\,, \qquad \Psi ^{[{s}]}:=\uppsi ^{[{s}]}_{|s|}\,, \end{aligned}$$

for $\mathcal {L}$ representing a null direction and $j_{s,k}=j_{s,k}(r)$ be some *r*-weights for $k\in \{0,...,|s|\}$. One can easily check that there is no choice of *r*-weights $j_{s,k}$ which would lead to a full decoupling of the equation for $\Psi ^{[{s}]}$ from the equation for $\upalpha ^{[{s}]}$ unless a=0. As we must accept some degree of coupling between the equation for $\Psi ^{[{s}]}$ and those for $\uppsi ^{[{s}]}_{(k)}$, 0≦k<|s|, the goal now is to choose our weights $j_{s,k}(r)$ in a way which, first and foremost, produces an equation for $\Psi ^{[{s}]}$ without the troublesome $\partial _t$ first order derivatives we encounter in the Teukolsky equation (1.2) and, secondly, “minimizes” the degree of coupling.

In our preprint [151] (see also [156]), we demonstrated that the choice

1.14 $$\begin{aligned} \uppsi _{(0)}^{[s]}=(r^2+a^2)^{-|s|+1/2}\Delta ^{|s|/2(1+{{\,\textrm{sign}\,}}s)}\upalpha ^{[{s}]}\,;\nonumber \\ \qquad \frac{\Delta }{(r^2+a^2)^2}\uppsi ^{[{s}]}_{(k+1)} = \mathcal {L} \uppsi _{(k)}^{[s]}\,,\,\, k=0,...,|s|\,, \end{aligned}$$

where $\mathcal {L}=L$ when s<0 and $\mathcal {L}=\underline{L}$ when s>0 is sufficient to satisfy the first requirement of the above paragraph, and to have the coupling to the variables indexed by k=0,...,|s| occur only through dependence on those variables and their azimuthal, $\partial _\phi$, derivative. Indeed, we show that if, for any $s\in \mathbb {Z}$, $\upalpha ^{[{s}]}$ solves the homogeneneous Teukolsky equation (1.2), then the PDE satisfied by $\Psi ^{[{s}]}$ is (cf. the compressed preliminary version given in (1.4))

1.15 $$\begin{aligned}  &   \mathfrak {R}^{[{s}]}\Psi ^{[{s}]}=a\mathfrak {J}^{[{s}]},\nonumber \\  &   \qquad \mathfrak {J}^{[{s}]}=\sum _{k=0}^{|s|-1} \frac{\Delta }{(r^2+a^2)^2} \left[ c^{\Phi }_{s,\,|s|,\,k}(r) \partial _\varphi +c^\textrm{id}_{s,\,|s|,\,k}(r)\right]\uppsi ^{[{s}]}_{(k)}, \end{aligned}$$

where $c_{s,\,|s|,\,k}^\Phi$ and $c_{s,\,|s|,\,k}^\textrm{id}$ are bounded functions in $[r_+,\infty )$ that can be explicitly computed by a recursive formula. The operator $\mathfrak {R}^{[{s}]}$, given by1.16where  is the *s*-spin-weighted laplacian (see already Section 3.1), can be seen as an analogue of $\Box _g$ (acting on the radiation field) for spin-weighted functions: it reduces to the wave operator acting on the radiation field when s=0, it has no first order terms, and its zeroth order terms, which are of a repulsive nature, are in fact better behaved than for s=0. This choice is actually a generalization to $s\in \mathbb {Z}$ of that developed in [47] for s=±2, and reduces to the transformations already introduced for s=±1 in [137] and s=±2 in [48].

We should also address the second requirement of the transformation theory: that our choices minimize the degree of coupling between (1.15) and (1.2). Indeed, the coupling terms in the right hand side of (1.15) have “good” *r*-weights, that decay with Δ as $r\rightarrow r_+$ and with $r^{-2}$ as $r\rightarrow \infty$, and only see the lower order transformed variables and their azimuthal derivatives. Perhaps the most convincing evidence for the weak strength of the coupling is the fact that, if $0\ne |a|\ll M$, thanks to *a* appearing as a multiplicative factor, it can be controlled in [47] solely by using transport equations (3.8). We note that the independent work [118, 119] in the very slowly rotating |a|≪M setting is based on the similar strategy, but relies on a slightly different transformed system.

#### Alternatives to our transformed system

If our main goal in the transformation of the Teukolsky equation (1.2) was to obtain a fully decoupled new equation for the entire |a|≦M Kerr family, our attempts above seem inadequate. An approach more likely to come to fruition would be to consider frequency space transformations with no clear physical space representation. This path was taken in much of the physics literature on rotating a≠0 Kerr: for s=±2, [30, 34, 147] have indeed succeeded in finding transformed quantities that satisfy decoupled equations by considering transformations that can only be interpreted in the separated picture. We do not pursue such transformations here, as having a physical space hierarchy is useful in the physical space analysis of our companion [152].

### From the wave equations to radial ODEs

Let $\upalpha ^{[{s}]}$ be a solution to the Teukolsky equation (1.2). By taking a Fourier–Laplace transform with respect to *t* and noting (1.2) commutes with $\partial _\phi$, we can formally decompose $\upalpha ^{[{s}]}$ into functions

1.17 $$\begin{aligned} \upalpha ^{[s],\,a, \, \omega }_{m}(t,r,\theta ,\phi ) = e^{-i\omega t} e^{im\phi }\tilde{\upalpha }^{[s],\,a,\, \omega }_m(r,\theta )\,, \end{aligned}$$

for $\omega \in \mathbb {C}$ and *m* an integer or half-integer depending on *s*. Plugging in the ansatz (1.17) into the Teukolsky equation (1.2), we obtain a PDE for $\tilde{\upalpha }^{[s],\,a, \,\omega }_m$ in the *r* and θ variables. This PDE is clearly separable if a=0, as (1.2) commutes with the generators of the spherical symmetries of the spacetime.

For rotating Kerr black holes, a≠0, it would seem that there are not enough symmetries for the same result to hold; yet, as we will see shortly in Section 1.2.1, it does. In this section, we use this separability to describe the main features of the Teukolsky equation and transformed system. We conclude, in Section 1.2.6, with a reflection on the role of separation of variables in our work and future applications.

#### Carter’s constant, formal separation and the Teukolsky radial ODE

Besides the conserved quantities induced by the stationary, $\partial _t$, and axisymmetric, $\partial _\phi$, Killing fields on Kerr, it turns out that there is a hidden conserved quantity for geodesics. The conservation law was first found by Carter [27], with the quantity being subsequently known as *Carter’s constant*, and was later shown to be associated with a Killing tensor [167]. Using this additional symmetry, Carter was able to show that, if s=0, the PDE for $\tilde{\alpha }^{[s],\,a, \,\omega }_m(r,\theta )$ admits separable solutions in the entire |a|≦M range; his result was then extended by Teukolsky [157] for s≠0 by analogy.

In what follows, a separable solution characterized by the frequency triple $(\omega ,m,\Lambda )$, where *m* is an integer or half-integer depending on *s* and $\omega ,\Lambda \in \mathbb {C}$, is written as in (1.6):

1.18 $$\begin{aligned} \upalpha _{m\Lambda }^{[{s}],\,a,\,\omega }(t,r,\theta ,\phi ) = e^{-i\omega t}e^{im\phi }S^{[s],\,a\omega }_{m\Lambda }(\theta )(r^2+a^2)^{-1/2}\Delta ^{-s/2}u_{m\Lambda }^{[{s}],\,a,\,\omega }(r)\,. \end{aligned}$$

Here, $S^{[s],\,a\omega }_{m\Lambda }$ satisfies an angular ODE together with boundary conditions that constrain the values of the separation constant Λ; for example (see for instance [155, Proposition 2.1])

1.19 $$\begin{aligned} \omega \in \mathbb {R}\implies \Lambda \in \mathbb {R}\,;\qquad \operatorname {Im}\omega >0\implies \operatorname {Im}\left[\left(\Lambda -a^2\omega ^2\right)\overline{\omega }\right]<0\,. \end{aligned}$$

The function $u^{[s],\,a,\, \omega }_{m\Lambda }$ satisfies the radial ODE given before as (1.7), which is written in full as

1.20 $$\begin{aligned}  &   \left(u^{[{s}]}\right)''+\left(\omega ^2-V^{[{s}]}\right)u^{[{s}]}=0,\nonumber \\  &   V^{[{s}]} = \frac{\Delta \Lambda +4Mram\omega -a^2m^2}{(r^2+a^2)} + s^2\frac{(r-M)^2}{(r^2+a^2)^2}+\frac{\Delta \left(a^2\Delta +2Mr(r^2-a^2)\right)}{(r^2+a^2)^4}\nonumber \\  &   \qquad \qquad -2is\frac{\omega \left(r(r^2+a^2)+M(a^2-3r^2)\right)+am(r-M)}{(r^2+a^2)^2}, \end{aligned}$$

with ′ denoting a derivative with respect to a modified radial coordinate $r^*$ that maps $r=r_+$ to $r^*=-\infty$ leaving r=∞ unchanged. We note the last line of the potential, which decays at a significantly slower rate as $r\rightarrow \infty$ than the first two lines, is only present when s≠0.

#### The Teukolsky–Starobinsky conservation law for the Teukolsky equation

As was mentioned already in the preamble to this introduction, the Killing energy associated to the vector field $\partial _t$ is not conserved for the Teukolsky equation (1.2) when s≠0, due to the additional first order terms. Nonetheless, a conservation law for the Teukolsky equation exists in the separated picture. It follows from the fact that, since $V^{[{s}]}=\overline{V^{[{-s}]}}$ in (1.20), the Wronskian between $u^{[{+s}]}$ and $\overline{u^{[{-s}]}}$ is conserved for the homogeneous Teukolsky equation. When $(\omega ,m,\Lambda )$ are real, using the Teukolsky–Starobinsky identities [106, 154, 159] to map solutions of the radial ODE of spin +s to spin -s or vice-versa, we can upgrade the conservation of the Wronskian to a conservation law involving only one sign of spin; we refer to the conserved quantity as the Teukolsky–Starobinsky energy. Taking s≧0, the Teukolsky–Starobinsky energy conservation takes the form

1.21 $$\begin{aligned}  &   (2\omega )^{2s}|u^{[{+s}]}|^2(\infty ) +\frac{\omega -m\upomega _+}{\omega }\nonumber \\  &   \quad \times \left\rbrace \begin{array}{lr} \frac{\prod _{j=1}^{|s|}\left\rbrace \left[4Mr_+(\omega -m\upomega _+)\right]^2+(s-j)^2(r_+-r_-)^2\right\lbrace }{(r_+-r_-)^{s}},\,& |a|<M\\ \left[4M^2(\omega -m\upomega _+)\right]^{2s},\,& |a|=M \end{array}\right\lbrace ^{-1} \!\!\!\!\!\!\!\mathfrak {C}_s |u^{[{+s}]}|^2(-\infty ) =0, \nonumber \\  &   \frac{\mathfrak {C}_s}{(2\omega )^{2s}}|u^{[{-s}]}|^2(\infty )+\frac{\omega -m\upomega _+}{\omega }\nonumber \\  &   \quad \times \left\rbrace \begin{array}{lr} \frac{\prod _{j=0}^{|s|-1}\left\rbrace \left[4Mr_+(\omega -m\upomega _+)\right]^2+(s-j)^2(r_+-r_-)^2\right\lbrace }{(r_+-r_-)^{s}},\,& |a|<M\\ \left[4M^2(\omega -m\upomega _+)\right]^{2s},\,& |a|=M \end{array}\right\lbrace |u^{[{-s}]}|^2(-\infty )=0\nonumber \\ \end{aligned}$$

where we used the notation $\upomega _+:=\frac{a}{2Mr_+}$, and $\mathfrak {C}_s$ are the so-called Teukolsky–Starobinsky constants, which depend only on *a*, *M*, |*s*| and the frequency triple $(\omega ,m,\Lambda )$. For |s|≦2, these constants satisfy

$$\begin{aligned} \mathfrak {C}_0=1\,,\,\,\mathfrak {C}_s\geqq 0\,,\text { for } |a|\leqq M\,; \qquad \mathfrak {C_s}\geqq (2|s|)^{2|s|}>0\,,\text { for } a=0\,, \end{aligned}$$

though for |s|>2 and a≠0, $\mathfrak {C}_s$ can take negative values [43].

For s≠0, the Teukolsky–Starobinsky energy assumes the role that the Killing energy has for s=0. It is also clear that it descends to a conserved energy for the transformed system, which we also call the Teukolsky–Starobinsky energy. For instance, by abuse of notation letting $\Psi ^{[{s}]}$ denote its radial part, we have for s≧0

1.22 $$\begin{aligned} \begin{aligned}&|\Psi ^{[{+s}]}|^2(\infty )+\frac{\omega -m\upomega _+}{\omega }\frac{\mathfrak C_s}{\mathfrak D_s^{\mathcal {H}}}|\Psi ^{[{+s}]}|^2(-\infty )=0, \\&\quad \frac{\mathfrak C_s}{\mathfrak D_s^{\mathcal {I}}}|\Psi ^{[{-s}]}|^2(\infty )+\frac{\omega -m\upomega _+}{\omega }|\Psi ^{[{-s}]}|^2(-\infty )=0\,, \end{aligned} \end{aligned}$$

where $\mathfrak D_s^{\mathcal {H}}$ and $\mathfrak D_s^{\mathcal {I}}$ are explicitly computable constants, connecting boundary terms of the Teukolsky and transformed variables, which depend only on *a*, *M*, |*s*| and the frequency triple $(\omega ,m,\Lambda )$. At least for |s|≦2, these constants satisfy

$$\begin{aligned} \mathfrak D_s^{\mathcal {I}},\mathfrak D_s^{\mathcal {H}}\geqq 0\,,\text { for } |a|\leqq M\,; \qquad \mathfrak D_s^{\mathcal {I}}=\mathfrak D_s^{\mathcal {H}}=\mathfrak C_s\,,\text { for}\,\, a=0\,. \end{aligned}$$

Note that, for s=0, (1.21) reduces to the fixed frequency space version of the Killing energy identity for (1.2) with the vector field $\partial _t$, and for a=0 (1.22) reduces to the fixed frequency space version of the Killing energy identity for (1.4) for the vector field $\partial _t$. It is, therefore, natural to wonder whether the Teukolsky–Starobinsky energy can be viewed as a physical space energy associated to some multiplier. Indeed, for a=0, since the *transformed* equation (1.4) takes the form of a wave equation on spin-weighted functions, it is clear that it conserves the physical space Killing energy associated to $\partial _t$, and this will imply a physical space conservation law for the *Teukolsky* equation (1.2) at a high level of differentiability; see also [120]. For general |a|≦M, as we have argued, the natural conservation law manifests itself at the level of the Teukolsky equation and not (directly at the level of) the transformed system.

The physical space analogue to the observation that the Wronskian between $u^{[{+s}]}$ and $\overline{u^{[{-s}]}}$ is conserved is the observation that changing *s* to -s in (1.2) is equivalent to mapping *t* to -t. To find a replacement for the Teukolsky–Starobinsky identities, we have to move from the independent Teukolsky equation (1.2) to the entire system of linearized Einstein vacuum equations around Kerr, where Weyl identities (see for example [1, 148, 159], or see [120] for a derivation in the simpler a=0 setting) show that there is a certain redundancy between the spin ±|s|, for |s|=1,2, extreme components in the Newman–Penrose formalism. Thus, it seems plausible that these observations can be combined to produce a physical space energy which combines the ±s cases of (1.2), but not necessarily for the individual spin signs in the full |a|≦M range as in (1.21) and (1.22).^8^

#### Superradiance and mode stability for the Teukolsky equation

In virtue of the separability property of the Teukolsky equation, which admits solutions (1.18) for $\omega ,\Lambda \in \mathbb {C}$ and $m\in \frac{1}{2}\mathbb {Z}$ integer or half-integer depending on *s*, one can immediately ask whether *mode solutions* exist with $\operatorname {Im}\omega >0$ or $\omega \in \mathbb {R}$. Mode solutions are solutions to the homogeneous (1.2) which take the separable form (1.18) and have outgoing boundary conditions, see already Definition 4.5, which means that they have (qualitatively) finite energy on a suitable spacelike hypersurface; note such solutions, if they exist, are exponentially growing in time, or bounded but non-decaying, respectively. We have seen already that the answer is no, to that is mode stability holds (see Theorem 0); in the remainder of this section, we recall to the reader why this result is not immediate and give context to the versions of the result we have already cited in the preamble to the introduction.

For a=0, mode stability is in fact immediate from the Teukolsky–Starobinsky energy conservation, which for integer *s* takes the form (1.21), and the fact that $\mathfrak {C}_s>0$. For s=0, where it is the Killing field $\partial _t$ which gives rise to the conservation law, we can interpret this result as a consequence of the fact that $\partial _t$ is timelike everywhere in the black hole exterior, except at the horizon.

On the other hand, if a≠0, there is a region close to the event horizon, called the ergoregion, where $\partial _t$ becomes spacelike. Thus waves coming in from null infinity may acquire more energy when passing through the ergoregion, which could, of course, be worrisome from the point of view of the stability statements we are after. As an example, let $\omega \in \mathbb {R}\backslash \{0\}$ and s=0; conservation of the Killing energy associated to $\partial _t$ (equivalently, specializing (1.21) to s=0) gives the identity

1.23 $$\begin{aligned} \omega (\omega -m\upomega _+)\left|u^{[{0}]}(-\infty )\right|^2+\omega ^2\left|u^{[{0}]}(+\infty )\right|^2=0\,, \qquad \upomega _+:=\frac{a}{2Mr_+}\,. \end{aligned}$$

Clearly, if $\omega (\omega -m\upomega _+)\geqq 0$ (note this is always true if a=0), we conclude that $u^{[{0}]}(\pm \infty )=0$, which implies u≡0 by standard ODE theory. In this case, the wave does not gain any extra energy, and we see that the wave equation is modally stable. On the other hand, (1.23) fails to provide an upper bound on the values *u* can take if the frequency parameters are *superradiant*, to that is if

1.24 $$\begin{aligned} \omega (\omega -m\upomega _+)< 0 \,. \end{aligned}$$

Similar statements holds true for real ω and spin $s=\pm 1,\pm 2$ when one considers the frequency space Teukolsky–Starobinsky energy conservation (1.21). For $\operatorname {Im}\omega >0$, though the identity following from the Teukolsky–Starobinsky energy conservation does not take the form (1.21), one can again see (at least in the s=0 case) that there are frequencies for which mode stability cannot be inferred from it. In conclusion, the conservation law for the Teukolsky equation of spin $s\in \{0,\pm 1, \pm 2\}$ does not guarantee its mode stability for rotating, a≠0, Kerr black holes.

Yet, remarkably, as we have mentioned, mode stability does hold for the Teukolsky equation (1.2), as summarized in Theorem 0. The pioneering result is due to Whiting [166], who showed that, for subextremal |a|<M parameters, there are no nontrivial mode solutions with $\operatorname {Im}\omega >0$ for any $s\in \frac{1}{2} \mathbb {Z}$. Whiting’s proof is based on an injective transformation of the s≦0 mode solutions into solutions of the scalar wave equation on a spacetime with no ergoregion: the Killing energy with respect to $\partial _t$ is conserved for the new solutions, and $\partial _t$ is never spacelike in the exterior in the new spacetime. Specifically, he considers an *integral* transformation of the radial component,

1.25 $$\begin{aligned} \tilde{u}(x) :&=(x^2+a^2)^{1/2}(x-r_-)^{-s}(x-r_+)^{-2iM\omega }e^{-i\omega x} \nonumber \\&\quad \times \int _{r_+}^\infty \left\rbrace e^{\frac{2i\omega }{r_+-r_-}(x-r_-)(r-r_-)}(r-r_-)^{i\frac{2Mr_-\omega -am}{r_+-r_-}}(r-r_+)^{-i\frac{2Mr_+\omega -am}{r_+-r_-}} e^{i\omega r}\right.\nonumber \\&\quad \qquad \qquad \left.\times \Delta ^{-s/2}(r^2+a^2)^{-1/2}u^{[{s}]}(r)\right\lbrace \textrm{d}r\,, \end{aligned}$$

and a *differential* transformation of the angular component which are well-defined for s≦0; to conclude mode stability for s>0 from the s≦0 result, he then appeals to the Teukolsky–Starobinsky identities we have mentioned in the previous section. More recently, the first author understood how to extend Whiting’s radial transformation (1.25) to $\omega \in \mathbb {R}\backslash \{0\}$, thus establishing a real mode stability result for the wave equation, s=0, on subextremal, |a|<M, Kerr in [150]; this was later generalized to $s\in \frac{1}{2}\mathbb {Z}$ [15] and to the wave equation s=0 in the Kerr–Newman (Kerr’s charged cousin) subextremal black hole [38]. Whiting’s transformation (1.25) fails in the extremal limit |a|→M; however, the second author has shown that another transformation of the mode solutions, specifically tailored to a degenerate horizon, can be employed to show mode stability for extremal |a|=M Kerr as well [155], both in the upper half-plane and on the real axis. An alternative proof of mode stability for |a|<M which replaces (1.25) and the Teukolsky–Starobinsky identities with a more direct analysis of the spectral symmetries of (1.20) was recently given by the second author with Casals in [44]. (See also [31, 41] for adaptations to similar black hole families.) We emphasize that the known proofs of mode stability on the real axis, of which we have given an exhaustive account in the previous paragraph, all crucially use the separability property of the Teukolsky equation (1.2).

To conclude the section, let us remaind the reader that, as we have mentioned in the preamble, we will be interested in the decompositions into separable solutions of the form (1.18) only for **real frequencies** $(\omega , m,\Lambda )$. In this case, one can show separable solutions form a complete basis of a space of “sufficiently integrable in time” solutions to the Teukolsky equation (1.2) (see already Section 1.4). Thus, from this point onwards, $(\omega , m,\Lambda )$ will always denote a set of real frequency parameters.

#### Trapping in the separated transformed system

Another important feature of Kerr black holes is the presence of *trapped* null geodesics. These are null geodesics which never intersect the event horizon nor escape to future null infinity, $\mathcal {I}^+$. An analysis of the equations of null geodesic flow easily yields that the future trapped null geodesics on the Schwarzschild black hole asymptote to a hypersurface of codimension one, characterized by r=3M in Boyer–Lindquist coordinates, usually called the photon sphere. By contrast, for rotating Kerr a≠0, they do not asymptote to a single hypersurface and there is a range of *r* values for which trapping can occur, see for example [35, Section 62]. However in the separated picture, trapping on a≠0 Kerr is immediately seen to be similar to that on Schwarzschild (a=0).

To understand the previous statement from the point of view of the problem at hand, let us consider our transformed system from Section 1.1, which naturally inherits the separability property of the Teukolsky equation: if $\uppsi ^{[{s}]}_{(k)}$ be obtained from a solution to (1.2) of the form (1.18) by the procedure in Section 1.1 and, by abuse of notation, we identify the variables with their radial component, we can write the radial ODE corresponding to (1.15) as

1.26 $$\begin{aligned} \begin{aligned}&\left(\Psi ^{[{s}]}\right)''+\left(\omega ^2-\mathcal {V}^{[{s}]}\right)\Psi ^{[{s}]}\\&=a\sum _{k=0}^{|s|-1} \frac{\Delta }{(r^2+a^2)^2} \left[ im c^{\Phi }_{s,\,|s|,\,k}(r) +c^\textrm{id}_{s,\,|s|,\,k}(r)\right]\uppsi ^{[{s}]}_{(k)}, \\&\begin{aligned} \mathcal {V}^{[{s}]}&= \frac{\Delta \Lambda +4Mram\omega -a^2m^2}{(r^2+a^2)} + s^2\frac{\Delta }{(r^2+a^2)^2}\\&\quad +\frac{\Delta }{(r^2+a^2)^4}\left[(1-2s^2)a^2\Delta +2(1-s^2)Mr(r^2-a^2)\right]. \end{aligned} \end{aligned} \end{aligned}$$

Note that this radial ODE has a real potential (because left the hand side of (1.15) has no first order derivatives) and that it behaves even better when s≠0 than in the wave equation case s=0 due to an additional, repulsive, term in the potential in the former case.

Let us focus on the radial ODE (1.26) either for s=0, or for any $s\in \mathbb {Z}$ if we also disregard all coupling terms. It is easy to see that, for each frequency triple $(\omega ,m,\Lambda )$, the potential admits a unique maximum at $r=r_{\max }(\omega ,m,\Lambda )$. As the frequency triple $(\omega ,m,\Lambda )$ is (unstably) trapped if $\mathcal {V}(r_\textrm{max})=\omega ^2$, in which case we set that $r_\textrm{trap}=r_\textrm{max}$,^9^ we find that the set where trapping can occur corresponds to a single *r*-value as in the Schwarzschild (a=0) case, but where this value of *r* now depends on the frequency triple.

Returning to the physical space picture, one notices that, unless |a|≪M is sufficiently small, some of the *r* values for which trapping are inside the ergoregion. Thus one could be concerned with the existence of waves that are trapped inside a certain *r*-range and continuously interact with the ergoregion to increasingly gain energy; such waves would likely not be bounded or decay in time. In the separated picture however, at least for subextremal, |a|<M, black holes, one can immediately see that this does not happen. There (see [57] for the s=0 case), one can show that solutions of the form (1.18) which are superradiant, to that is for which (1.24) holds, and high energy ($|\omega |\gg 1$) are quantitatively non-trapped in the sense that there is an ϵ>0 such that

1.27 $$\begin{aligned} \omega (\omega -m\upomega _+)\leqq 0\,, \,\, |\omega |\gg 1 \implies \mathcal {V}^{[{s}]}(r_\textrm{max})-\omega ^2\geqq \epsilon \omega ^2\gg 1\,. \end{aligned}$$

We note that ϵ can be made independent of ω in the entire subextremal Kerr range |a|<M, though in the limit |a|→M we have that $\epsilon \rightarrow 0$ as $\omega \rightarrow m\upomega _+$.

We emphasize that (1.27) is one of the main observations that allowed for a proof of boundedness and decay for the wave equation, s=0, in the full subextremal |a|<M range [57]. Its failure in the extremal |a|=M case is one of the main reasons why general solutions to the wave equation on extremal Kerr black holes are still poorly understood, see Section 1.5.2 below for a brief overview of the literature.

#### Alternatives to separation of variables for a=0 and |a|≪M

Only in this perturbative setting of the very slowly rotating |a|≪M Kerr subfamily, where one need not appeal to the mode stability of Section 1.2.3, can one hope to be able to avoid separation of variables altogether. This hope is well-founded: several techniques bypassing explicit separation of variables have been developed for s=0 and |a|≪M, which we now review briefly.

Uniform boundedness of energy for the scalar wave equation s=0 in the very slowly rotating case |a|≪M was first proven in [55]. The argument had the interesting feature that it was able to avoid any detailed analysis of trapping, and in particular the use of separation of variables, by only relying on a decomposition of the solution into a superradiant and non-superradiant piece.

In order to show decay, rather than just boundedness, one must also understand trapping. One reason to consider some kind of frequency space analysis in proving a result such as our Main Theorem comes from the observation, due to Alinhac [14], that unless a=0, already for s=0, there are no classical physical space vector field multipliers which can capture trapping (see Section 1.2.4). Microlocal analysis based on a local reduction to Euclidean space provides an alternative framework to connect the unstable trapping of the geodesic flow with so-called positive commutator estimates; this approach has been used successfully, for s=0, to establish integrated energy decay statements in the very slowly rotating |a|≪M Kerr case [160] (also later in the work [82]). See also [60, 83, 168], and the references therein for results which use microlocal analysis to relate the high frequency behavior of waves with the underlying normal hyperbolic trapping. If one imposes control on higher norms in the initial data, thus leaving the scope of Alinhac’s observation, an analogue of our Main Theorem for s=0 and |a|≪M can also be established using a physical space multiplier, based on the Killing tensor representing Carter’s hidden conservation law, to capture trapping [7, 76].

#### Is separation of variables necessary in the full subextremal |a|<M Kerr family?

Yes: as long as there is no proof of mode stability (Theorem 0) which bypasses separation of variables, see Section 1.2.3, separation of variables cannot be entirely abolished from the analysis of perturbations of general subextremal |a|<M Kerr black holes. In our series of works, we view separation of variables as a concrete, convenient and explicit approach to frequency analysis which is intimately tied to the Kerr geometry and allows us to treat several aspects of the Teukolsky equation simultaneously under the same formalism. In addition to mode stability, we will also take advantage of the separation of variables to employ, when needed, the s≠0 Teukolsky–Starobinksy energy conservation of Section 1.2.2, see already 1.3.4. It also allows for a very explicit characterization of trapping as an unstable phenomenon which decouples from superradiance, see Section 1.2.4, and occurs only at the top level of derivatives, see already Section 1.3.3. While high frequency phenomena such as trapping may not require a complete, global separation of variables to be understood (see our comments on energy boundedness in Section 1.4), applying separation of variables to understand frequency phenomena should not be seen an (insurmountable) obstacle to applications to nonlinear problems, see [50].

### Frequency space analysis: main difficulties and ideas

In this section, we outline the proof of Theorem 1. In a nutshell, this result is proved by applying multiplier currents to the separated version of the transformed system from Section 1.1, in particular to the radial ODE (1.26). These currents, which are of virial and energy type, are tailored to the frequency triple $(\omega ,m,\Lambda )$, though they result in uniform estimates. Roughly speaking, we employ virial currents to produce control over a bulk term with good properties, such as that in (1.11); energy currents are used to control boundary terms as in (1.10). In this section, we give an overview of our strategy to build such currents and, thus, prove Theorem 1. The discussion here is quite detailed, as we do not assume familiarity with the relevant prior works [57] for s=0 and [47] for s=±2 with |a|≪M, with which we will nevertheless compare our approach.

For readability, in complicated formulas, we will drop the superscripts [*s*] from the transformed variables $\Psi ^{[{s}]}$ and $\uppsi ^{[{s}]}_{(k)}$ unless doing so can cause confusion.

**Table 1:**
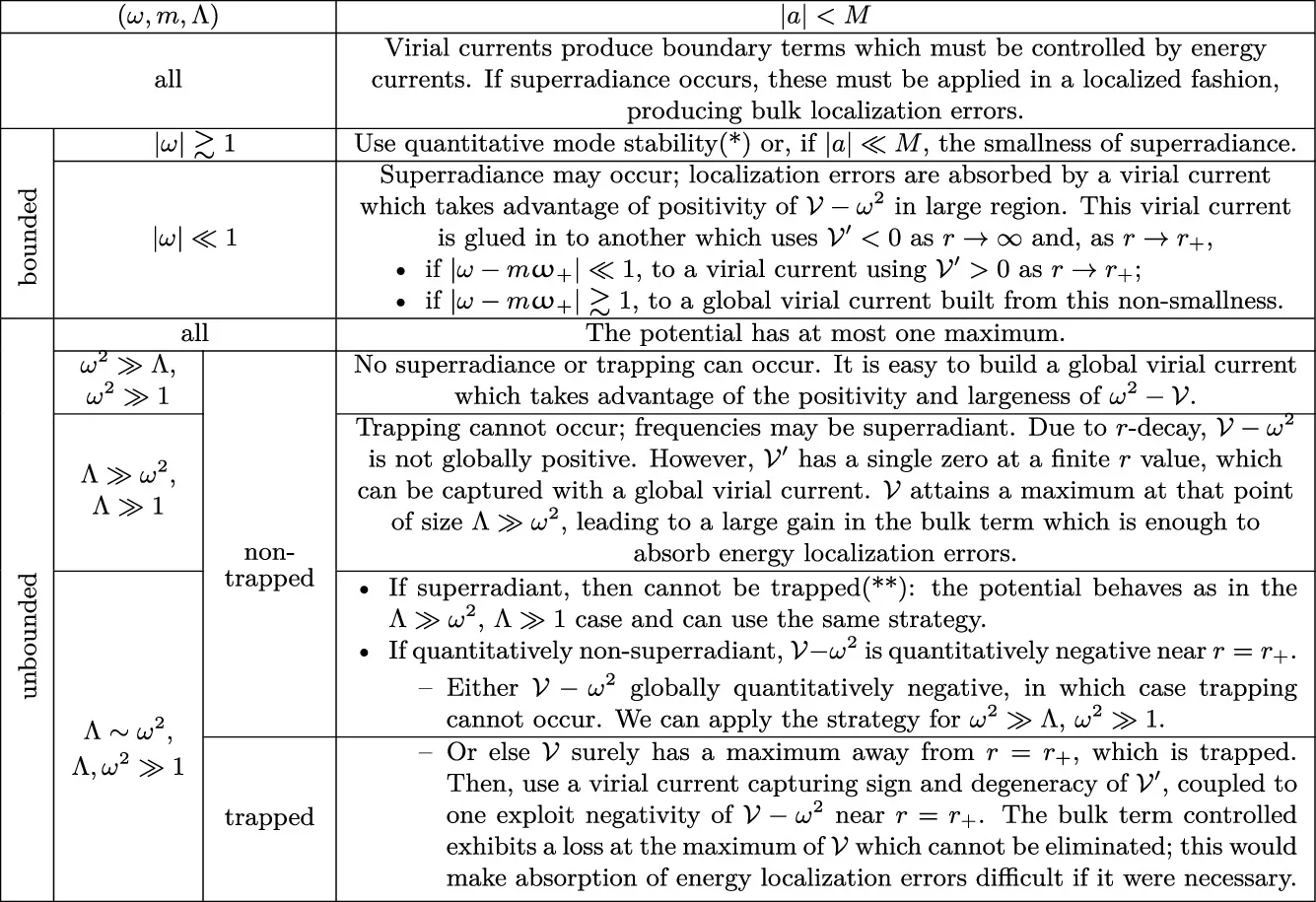
The frequency space analysis of the operator $\mathfrak {R}^{[{s}]}$ (ignoring coupling) in the full subextremal range |a|<M and their treatment. The analysis is based on the $\Box _{g}$ case addressed in [57]. We denote by $\mathcal {V}$ the potential in the radial ODE associated with these operators

(*) The quantitative control of mode stability only degenerates in the double limit |a|→M, $|\omega -m\upomega _+|\rightarrow 0$ [155]

(**) If |a|<M, the two alternatives given for $\omega ^2\sim \Lambda \gg 1$ exhaust the space of possible frequencies (see Section 1.2.4), because superradiant frequencies are non-trapped by a quantifiable amount. As |*a*|/*M* increases, this amount becomes smaller; when |a|=M, the conclusion holds only if frequencies approaching the superradiant threshold are excluded

#### Scalar wave techniques: the transformed operator $\mathfrak {R}^{[{s}]}$

Since $\mathfrak {R}^{[{s}]}$ can be seen as a spin-weighted analogue of the s=0 case, where $\mathfrak {R}^{[{0}]}$ is the wave operator $\Box _g$ acting on the radiation field, the techniques we rely on for its analysis (while ignoring the coupling terms of (1.15)) are precisely those one would consider when studying the scalar wave equation on a Kerr background. Thus, while the contents of this section build heavily on all of the previous literature, they are based especially on the results for the wave equation on subextremal Kerr [56–58] and those for the Teukolsky equation of spin |s|=1,2, respectively [48, 137], on Schwarzschild.

Recall the transformed radial ODE (1.26) with any $s\in \mathbb {Z}$, focusing only on the left hand side (to that is ignoring the coupling) or setting s=0. In the analysis of this ODE, the main phenomena to keep in mind are superradiance, mode stability and trapping, which have been discussed in the previous Section 1.2. Concretely, we partition the space of admissible frequency parameters as follows (see Table 1 for a summary).

- *High frequencies*. When one or more of the frequency parameters is large, we again want to build targeted virial multiplier currents for the radial ODE under consideration. The frequency-independent part of the potential becomes a lower order correction to the behavior of solutions of the radial ODE; to determine what the virial currents should look like, we are mainly interested to know whether it is Λ (a proxy for the size of the potential) or $\omega ^2$ which is driving the ODE. If $\omega ^2\gg \Lambda$ or if $\Lambda \gg \omega ^2$ are large, we can exploit the largeness of the difference between $\omega ^2$ and the potential or vice-versa and there is no trapping. Only when $\omega ^2\sim \Lambda$ do we not know whether it is $\omega ^2$ or the potential which is dominating and, then, trapping can occur; implying a (*r*-localized) loss in our estimates.
- *Bounded frequencies*. Either (i) or (ii), as below, hold.

(i) $|\omega |\ll 1$. In this case, it is the frequency-independent part of the potential that drives the radial ODE; one can construct virial multiplier currents that take advantage of its properties.
(ii) $1\lesssim |\omega |\lesssim 1$, to that is $(\omega ,m,\Lambda )\in \mathcal {A}$ where $\mathcal {A}$ is the set which is excluded from Theorem 1. Nevertheless, let us remark how one can obtain estimates in this case. In the very slowly rotating case |a|≪M, we use the smallness of this parameter to construct suitable multiplier currents (see also [54] for s=0). In the general subextremal case |a|<M, multiplier currents near the $r^*\rightarrow \pm \infty$ ends allows us to have the desired control up to bounded $|r^*|$ bulk errors. These are estimated by using Theorem 0 to build, and estimate, a Green’s formula for the radial ODE (see our companion [152]).

For $(\omega ,m,\Lambda )\not \in \mathcal {A}$, where $\mathcal {A}$ is as in Theorem 1, the previous discussion is our guide in the construction of virial multiplier currents for the radial ODE (1.26) which, coupling $\mathfrak {J}^{[{s}]}$ aside, are effective in capturing the properties of $\mathfrak {R}^{[{s}]}$ in a good bulk estimate of the type of (1.11). Note, however, that these virial currents produce boundary terms with a bad sign which must be controlled by applications of some energy current. If superradiance (1.24) occurs, then one of the boundary terms in our energy current will have bad sign (recall for example (1.23) for s=0), hence the current must be applied in a localized fashion, thus producing localization errors. It is fortunate that, on subextremal, |a|<M, Kerr black holes, if trapping occurs and brings about the expected loss in our estimates, the frequency parameters cannot be superradiant, hence, one does not need to deal with such localization errors.

As we let $\mathfrak {J}^{[{s}]}$ back into (1.26), the necessity of appealing to energy currents in the above procedure leads to question: which energy? Indeed, in principle we can both appeal to the Killing energy currents associated to $\partial _t$ and $\partial _t+\upomega _+\partial _{\phi }$, which are conserved by scalar waves (s=0) but not by (1.26) due to $\mathfrak {J}^{[{s}]}$; or we can appeal to Teukolsky–Starobinsky energy currents, which are conserved by (1.26) but involve complicated frequency weights, see (1.22). Though it is tempting (and somewhat easier, see our preprint [151]) to use the latter, we instead rely mostly, to that is when possible, on Killing energy currents. This allows us to minimize certain technical difficulties associated with summing the frequency estimates in our companion [152].

#### Transport techniques: a first approach to the coupling $\mathfrak {J}^{[{s}]}$

For each k<|s|, $\uppsi _{(k)}^{[s]}$ is related to $\Psi ^{[{s}]}$ by a sequence of |s|-k transport equations, as given in (1.14). By abuse of notation, as usual let us identify a fixed frequency $\uppsi _{(k)}^{[s]}$ with its radial component; then (1.14) read in the frequency space picture as

1.28 $$\begin{aligned} \frac{\Delta }{(r^2+a^2)^2}\uppsi _{(k+1)}^{[s]}=-{{\,\textrm{sign}\,}}s \left(\uppsi _{(k)}^{[s]}\right)'-i\left(\omega -\frac{am}{r^2+a^2}\right)\uppsi _{(k)}^{[s]}\,. \end{aligned}$$

In this section, we review some of the consequences of (1.28) and their implications for our treatment of the coupling errors, due to $\mathfrak {J}^{[{s}]}$, in our implementation of the scalar wave techniques outlined in the Section 1.3.1.

An immediate estimate one can obtain from the transport equation (1.28) is, for instance,

1.29 $$\begin{aligned}&\int _A^B c'(r)\left|\uppsi _{(k)}\right|^2\textrm{d}r^* - 2\left[c(r)\left|\uppsi _{(k)}\right|^2\right]_{r^*=A}^{r^*=B} \nonumber \\&\quad \leqq 4\int _A^B \frac{\Delta ^2}{(r^2+a^2)^4}\frac{c^2}{c'}\left|\uppsi _{(k+1)}\right|^2\textrm{d}r^*\,, \end{aligned}$$

where *c*(*r*) is a continuous, piecewise $$C^1$$, *r*-weight with $c^{\prime }>0$, and $$A,B\in \mathbb {R}\cup \{\pm \infty \}$$. Commuting (1.28) with $$\partial _{r^*}$$ and then repeating the steps leading to (1.29), we also obtain

1.30 $$\begin{aligned} \int _A^B&c'\left(\omega -\frac{am}{r^2+a^2}\right)^2\left|\uppsi _{(k)}\right|^2\textrm{d}r^*\nonumber \\&\leqq \left[c(r)\left|\uppsi _{(k)}'\right|^2\right]_{r^*=A}^{r^*=B}+\int _{A}^B \frac{2\Delta ^2}{(r^2+a^2)^4}\left(\frac{2c^2}{c'}+c'\right)\left|\uppsi _{(k+1)}'\right|^2\textrm{d}r^* \nonumber \\&\qquad +\int _A^B \frac{16 r^2\Delta ^2}{(r^2+a^2)^6}\frac{c^2}{c'}a^2m^2|\uppsi _{(k)}|^2\textrm{d}r^*\,. \end{aligned}$$

Estimates (1.29) and (1.30) provide an easy way of climbing the hierarchy, to that is relating integrated estimates for $\uppsi _{(k)}$ to integrated estimates for $\uppsi _{(k+1)}$ and, eventually, to integrated estimates for Ψ. Less immediate consequences of the transport equation (1.28) are the identity

1.31 $$\begin{aligned} \begin{aligned}&- c\operatorname {Im}\left[im\omega \overline{\Psi }\uppsi _{(k)}\right]{{\,\textrm{sign}\,}}s\\&\quad = -\left[cm\left(\operatorname {Im}\left[\overline{\Psi }\uppsi _{(k)}\right]+\frac{am^2(r^2+a^2)}{2\Delta }\left|\uppsi _{(k)}\right|^2\right)\right]'\\&\qquad +mc\operatorname {Im}\left[\overline{\Psi }'\uppsi _{(k)}\right]+\frac{1}{2}\left(\frac{(r^2+a^2)c}{\Delta }\right)'am^2\left|\uppsi _{(k)}\right|^2 \,, \end{aligned} \end{aligned}$$

for k=|s|-1 and a similar identity which can be obtained for k<|s|-1, see (the proof of) Lemma 6.20, which was originally obtained in [47, Propositions 5.3.1 and 8.4.1]. These identities apply to terms arising from the interaction of Killing energy currents with coupling terms on the right hand side of (1.26): after Cauchy–Schwarz, we see that the errors reduce to $a^2(m^2+1)|\uppsi _{(k)}|^2$ bulk terms with k<|s|.

If |a|≪M is taken to be sufficiently small, then estimates (1.29), (1.30) and (1.31) are enough to show that any error due to the interaction of the virial or Killing energy currents with the coupling terms on the right hand side of (1.26) is lower order and can be controlled by the left hand side of the bulk estimate (1.11). Indeed, the only situation where the smallness of |*a*| is not enough to conclude directly from (5.12) is if there is trapping, to that is if $r_\textrm{trap}(\omega ,m,\Lambda )\ne 0$. In that case, we notice that since

1.32 $$\begin{aligned} \left(\omega -\frac{am}{r^2+a^2}\right)^2-a^2m^2 > rsim \omega ^2 > rsim m^2\,, \end{aligned}$$

one has that coupling terms $a^2(m^2+1)|\uppsi _{(k)}|^2$ can be controlled, via (1.30), by the non-degenerate $|\Psi '|^2$ bulk term on the left hand side of the bulk estimate (1.11). In other words, the transport estimates (1.30) and (1.31) show that, as in the a=0 case, $\uppsi _{(k)}$ with k<|s| do not experience trapping as long as |a|≪M.

In the general |a|≦M case, not only does the above procedure show that $\uppsi _{(k)}$ with k<|s| are not trapped fail, but also we have problems already at the level of the bounded frequency range: without *a* as a small parameter, (1.29) suggests that there can be no gain in smallness when comparing coupling errors with Ψ bulk terms. Thus, it is unclear at this point whether the coupling on the right hand side of (1.26) should even be seen as *error* terms! Table 2 summarizes these observations.

**Table 2:**
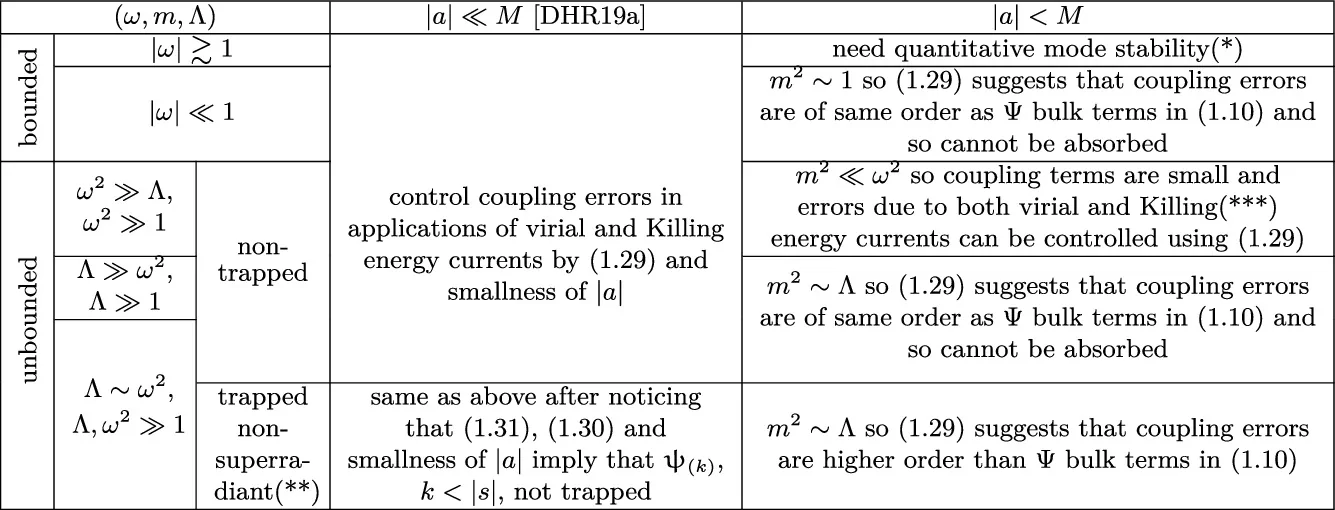
Difficulties associated with the analysis of coupling $\mathfrak {J}^{[{s}]}$ in (1.15), in the case |a|≪M and in the full subextremal range |a|<M. We assume here that the left hand side of (1.15) has been treated by the methods contained in Table 1 and examine coupling errors arising from those methods using solely the consequences of the transport relations (1.28) identified in Section 1.3.2

(*), (**) See the analogous notes in Table 1

(***) For technical reasons related to adding up the frequency space estimates, we will not use this strategy

In the next sections, we break down the issues arising in each of the frequency ranges identified in Table 2 and explain the novel ideas required to deal with them.

#### Elliptic estimates: the coupling $\mathfrak {J}^{[{s}]}$ for high frequencies

In the case of $\omega ^2+m^2+|\Lambda |\gg 1$, recall that, in view of the results in the last Section 1.3.2, the coupling errors which we still need to contend with are bulk terms of the form $a^2(m^2+1)|\uppsi _{(k)}|^2$, with suitable *r*-weights. Indeed, the rudimentary transport estimate (1.29) loses derivatives. It only allows us to say that such errors are controlled by suitably *r*-weighted $a^2(m^2+1)|\Psi |^2$ bulk terms and so, unless |a|≪M, it provides no gain in smallness and, in fact, even leads to a term which is too higher order, cf. (1.11), if $(\omega ,m,\Lambda )$ is trapped.

To deal with the coupling in the transformed equation (1.26), estimates using the transport identity (1.28) seem insufficient. However, as we have already noted in the preamble to this introduction, there are still more equations we can use. By their constructions, Ψ is not the only transformed variable that verifies a wave-type equation; for all k<|s|, $\uppsi _{(k)}$ also satisfy wave-type equations, which translate into the radial ODEs, cf. (1.20) and (1.26),

1.33 $$\begin{aligned}&\left(\uppsi _{(k)}^{[s]}\right)''+(|s|-k)\frac{2(r^3-3Mr^2+a^2r+a^2M)}{(r^2+a^2)^2}\left(\uppsi _{(k)}^{[s]}\right)'+\left(\omega ^2-\tilde{\mathcal {V}}_{(k)}^{[s]}\right)\uppsi _{(k)}^{[s]}\nonumber \\&\quad = \sum _{i=0}^{k-1}\frac{\Delta }{(r^2+a^2)^2} \left[ c^{\Phi }_{s,\,k,\,i}(r) im+c^\textrm{id}_{s,\,k,\,i}(r)\right]\uppsi ^{[{s}]}_{(i)}\,, \end{aligned}$$

for some $c^{\Phi }_{s,\,k,\,i}(r)$ and $c^\textrm{id}_{s,\,k,\,i}(r)$ which are bounded for $r\in [r_+,\infty )$. Unlike $\mathcal {V}$, the potential $\tilde{\mathcal {V}}_{(k)}$ has a nontrivial imaginary component with poor decay as $r^*\rightarrow \pm \infty$, see already (4.19), but with a special structure: using (1.28), we rewrite the above as a radial ODE for $\uppsi _{(k)}$ which is coupled to $\uppsi _{(k+1)}$ and $\uppsi _{(j)}$, j<k,

1.34 $$\begin{aligned}&\left(\uppsi _{(k)}^{[s]}\right)''+\left(\omega ^2-\mathcal {V}_{(k)}^{[s]}-\frac{4iamr\Delta }{(r^2+a^2)^3}(|s|-k){{\,\textrm{sign}\,}}s\right)\uppsi _{(k)}^{[s]}\nonumber \\&\quad = {{\,\textrm{sign}\,}}s (|s|-k)\frac{2(r^3-3Mr^2+a^2r+a^2M)\Delta }{(r^2+a^2)^4}\uppsi _{(k+1)}^{[s]}\nonumber \\&\qquad +\sum _{i=0}^{k-1}\frac{\Delta }{(r^2+a^2)^2} \left[ c^{\Phi }_{s,\,k,\,i}(r) im+c^\textrm{id}_{s,\,k,\,i}(r)\right]\uppsi ^{[{s}]}_{(i)}\,. \end{aligned}$$

In the above, $\mathcal {V}_{(k)}$ is a scalar wave like potential:

$$\begin{aligned} \mathcal {V}_{(k)}^{[s]}=\operatorname {Re}\tilde{\mathcal {V}}_{(k)}^{[s]}=\frac{\Delta (\Lambda +|s|+k(2|s|-k-1)) +4Mram\omega -a^2m^2}{(r^2+a^2)^2}+\dots \,, \end{aligned}$$

where we have neglected the terms with faster decay as $r\rightarrow \infty$. Again using (1.28), we can recast (1.34) as a constraint equation relating $\uppsi _{(k+1)}$ to $\uppsi _{(j)}$, j≦k:

1.35 $$\begin{aligned}&w\left(\Lambda -2am\omega +|s|+k(2|s|-k-1)+\dots \right)\uppsi ^{[{s}]}_{(k)}\nonumber \\&\quad ={{\,\textrm{sign}\,}}s \left(\uppsi ^{[{s}]}_{(k+1)}\right)'-i\left(\omega -\frac{am}{r^2+a^2}\right)\uppsi ^{[{s}]}_{(k+1)}+{{\,\textrm{sign}\,}}s (|s|-k-1)\nonumber \\  &\frac{2(r^3-3Mr^2+a^2r+a^2M)\Delta }{(r^2+a^2)^4}\uppsi ^{[{s}]}_{(k+1)} \nonumber \\&\qquad \quad +\sum _{j=0}^{k-1}\frac{\Delta }{(r^2+a^2)^2}\left(ac_{s,\,k,\,j}^{\Phi }im+ ac_{s,\,k,\,j}^{\textrm{id}}\right)\uppsi ^{[{s}]}_{(j)}\,. \end{aligned}$$

We have rewritten (1.35) in a suggestive way: if $\Lambda -2am\omega \sim \Lambda > rsim \omega ^2$ is sufficiently large, then (1.35) leads to an elliptic estimate of the form

1.36 $$\begin{aligned} \int _{-\infty }^\infty \frac{\Delta \Lambda }{(r^2+a^2)^2}|\uppsi _{(k)}|^2\textrm{d}r^*\lesssim \int _{-\infty }^\infty \frac{\Delta }{(r^2+a^2)^2}|\uppsi _{(k+1)}|^2\,, \end{aligned}$$

which does not lose derivatives, cf. the transport estimate (1.29). The ellipticity condition $\Lambda -2am\omega \sim \Lambda$ clearly holds in the angular dominated regime $\Lambda \gg \omega ^2$, and it is also not hard to see that it is a necessary condition for $(\omega ,m,\Lambda )$ to be trapped. Thus, (1.36) implies that, in the full Kerr black hole range |a|≦M, the lower level transformed variables, $\uppsi _{(k)}$ with k<|s|, never experience trapping.

Let us note that, for trapped frequencies, (1.36) suggests only that the coupling errors are at the same order of the bulk terms of Ψ on the left hand side of (1.11), but we do not yet have any smallness. Actually, this is only due to the fact that (1.11) and the methods outlined in Section 1.3.1 are not sharp from the point of view of the loss at trapping. For instance, a Hardy estimate shows that control over a $|\Psi '|^2$ bulk term without degeneration implies that we do not need to lose a full time/angular derivative at $r_\textrm{trap}$, but merely half; this shows that the coupling errors, treated via (1.36), are actually small.

Finally, in the regime of non-ellipticity, to that is when $\Lambda -2am\omega \sim \Lambda$ does not hold, we cannot call on (1.36) to close our estimates and the previous transport estimates also do not give us the necessary smallness. Our strategy will be to revisit the scalar wave techniques of Section 1.3.1. This will be done in a manner very similar to the case of bounded frequencies, which we address in detail in the next section.

We summarize the above in Table 3 below, though see Section 6.2.1 for a more detailed discussion.

#### Scalar wave techniques revisited: the coupling $\mathfrak {J}^{[{s}]}$ for bounded frequencies

We now turn to the bounded frequency regime, when $|\omega |+|m|+|\Lambda |\lesssim 1$, and focus on the case $|\omega |\ll 1$. From the transport estimate (1.29), it is not hard to see that the coupling errors arising from the application of virial and Killing energy currents to the (1.26) become small as $r^*\rightarrow ({{\,\textrm{sign}\,}}s)\infty$. However, in a bounded $|r^*|$ region, there is no gain from (1.29) unless |a|≪M, see also Table 2.

As in the high frequencies, we will move from transport estimates (1.28) to estimates for the wave equations (1.33) and (1.34) verified by the lower level transformed variables. For each k=0,⋯,|s|-1, we attempt employ the scalar wave techniques of Section 1.3.1:

- In the region $r^*\rightarrow \pm \infty$, by applying the methods of Section 1.3.1 to (1.34), one obtains an estimate for large $|r^*|$ bulk terms of $\uppsi _{(k)}$ and its boundary terms, up to bulk errors arising from both the coupling to $\uppsi _{(j)}$ with j<k (as before) and due to $\uppsi _{(k+1)}$. The latter enjoy improved *r*-weights as $r^*\rightarrow \pm \infty$, so this *forward* coupling has smallness even if the *backward* coupling to $\uppsi _{(j)}$, j<k, does not.
- In the bounded $|r^*|$ region, the virial currents suggested in Section 1.3.1 are insensitive to the imaginary component of the potential in the equation. Thus, by applying them to (1.33), one obtains an estimate for bounded $|r^*|$ bulk terms of $\uppsi _{(k)}$, up to non-small coupling errors to $\uppsi _{(j)}$ with j<k and a small error in $\uppsi _{(k)}$ to be absorbed by the $r^*\rightarrow \pm \infty$ choice of currents. We emphasize that, critically, this estimate does not see any coupling to $\uppsi _{(k+1)}$; and this is quite fortunate as, if it did, we would not be able to infer any smallness from their *r* weights in a bounded $|r^*|$ region.

Thus, putting these together, we see that we obtain a hierarchy of estimates for $\uppsi _{(k)}$, k=0,⋯|s|-1: $\uppsi _{(0)}$ is estimated in terms of a small $\uppsi _{(1)}$ error, $\uppsi _{(1)}$ is estimated in terms of a small $\uppsi _{(2)}$ error and a non-small $\uppsi _{(0)}$ error, etc. This hierarchy of estimates closes, leading to the desired conclusion for bounded frequencies.

There are some subtleties in implementing the above procedure. Perhaps the most important one has to do with the energy currents considered in the scalar wave techniques: here we have glossed over the fact that these currents, unlike the virial currents, are applied in a (semi-)global fashion, leading to coupling errors which cannot be boxed into a single one of the two categories above. Since |ω| is small, it is not hard to see that errors due to the Killing $\partial _t$ energy current are also small and thus do not significantly affect our scheme. In turn, applying the Killing energy current induced by $\partial _t+\upomega _+ \partial _{\phi }$ is not problematic only if the strength of superradiance, $|\omega -m\upomega _+|$, is also small. When am≠0 quantitatively, this is not the case and so we find it necessary to replace this Killing current near $r=r_+$ by a current associated to the Teukolsky–Starobinsky energy of Section 1.2.2: as this is a conserved quantity for the system, it does not produce any coupling errors.

We summarize the above in Table 3 below, though see Section 6.2.2 for a more detailed discussion.

**Table 3:**
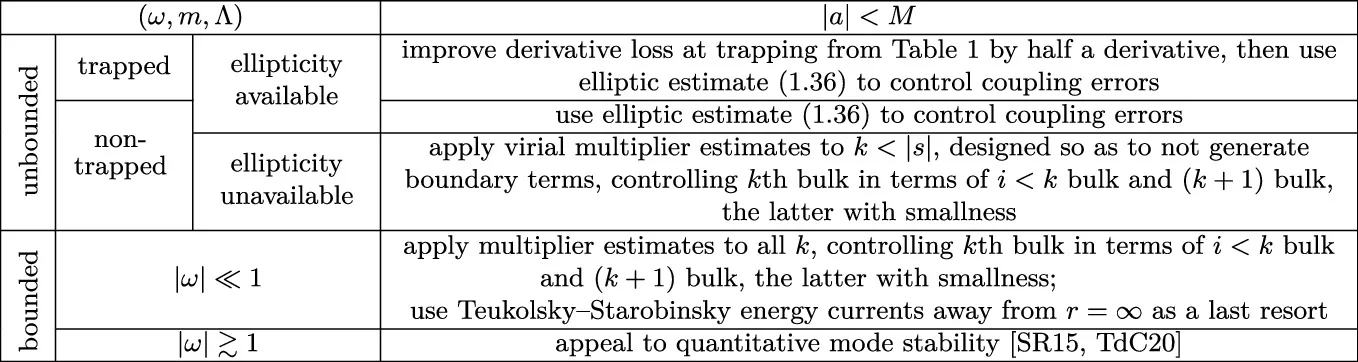
Analysis of coupling $\mathfrak {J}^{[{s}]}$ in (1.15) in the full subextremal range |a|<M, cf. Tables 1 and 2

### Physical space analysis: a summary of our companion paper

In the preamble to this introduction and in Section 1.2, we have already explained that, if general solutions to (1.2) and to the transformed system (1.3)–(1.5) are $L^2$ in the Boyer–Lindquist *t*, then by Plancherel and the completeness of spin-weighted spheroidal harmonics, they can be decomposed into an infinite superposition of separable solutions (1.6) with $\omega \in \mathbb {R}$ and (m,Λ) discrete. Thus, under the $L^2_t$ assumption, the ODE estimates in Theorems 0 and 1 can be immediately summed to produce PDE estimates. It is not hard to see, however, that the $L^2_t$ assumption does not hold for solutions to our PDEs arising from data on an (arbitrary) Cauchy hypersurface $\Sigma _0$. To meet this assumption of sufficient integrability, we must therefore modify our equations by adding cutoffs. Indeed, Theorems 0 and 1 are the key conceptual inputs to obtain our Main Theorem, applied over and over in slightly different (cutoff) settings and with different technical steps.

We turn to a brief description of the implementation in [152] which, in spite of significant technical difficulties for the case s≠0, follows along the lines of the work of the first author with Dafermos and Rodnianski [57] in the case s=0. We apply our frequency analysis in three distinct instances, characterized by three different types of cutoffs:

- *Time cutoffs* to the past of $\Sigma _0$. For solutions to the PDEs which are assumed to be future integrable, to that is $L^2_t$ in the future of $\Sigma _0$, we combine Theorem 1 with Theorem 0 to obtain a statement of the form
  1.37 $$\begin{aligned} I^\textrm{deg}(0,\tau ) \lesssim E(0)\,, \quad \forall \,\tau >0\,. \end{aligned}$$
  Here, for a suitable foliation $\{\Sigma _\tau \}_{\tau }$ generated by the flow of the stationary Killing field on $\Sigma _0$, E(τ) denotes Sobolev energy norm on $\Sigma _\tau$, and $I^\textrm{deg}(0,\tau )$ denotes an *r*-weighted Sobolev norm on the time slab between $\Sigma _0$ and $\Sigma _{\tau }$ at the same level of regularity as *E*(0) everywhere except at trapping (where it is half a derivative below the regularity of *E*). Indeed, if we cutoff to the past of $\Sigma _0$, then the inhomogeneities arising in the Teukolsky equation and transformed system can be computed explicitly in terms of the nontrivial gradient of the cutoff function, which is localized to a finite time slab around $\Sigma _0$. Thus, while showing that the right hand side of (1.11) should be controlled by the Sobolev norm of the initial data without derivative loss (beyond that expected by trapping) is somewhat technical,^10^ conceptually it is not too surprising that (1.37) should hold.
- *Radial cutoffs*. Under the same future integrability assumption, one then shows that, using the subscript $[-R^*,R^*]$ to indicate a restriction to a region away from $r^*=\pm \infty$, a past extension of the solution satisfies
  1.38 $$\begin{aligned} I^\textrm{deg}_{[-R^*,R^*]}(-\infty ,\infty ) \lesssim _{R^*} E(0) \,. \end{aligned}$$
  In proving this result, we actually do not need to invoke Theorem 0 but rather only the more precise version of our Theorem 1 given in Section 6.
- *Time cutoffs* to the past of $\Sigma _0$ and future of $\Sigma _{\tau }$. If we do not assume that the solutions of our PDEs have any sort of past or future integrability, then these are the kind of cutoffs which we should consider in order to apply our frequency analysis. Following the same steps as for past cutoffs, to that is by combining our Theorem 1 with Theorem 0 and carefully estimating the inhomogeneity errors, we have
  1.39 $$\begin{aligned} I^\textrm{deg}(0,\tau ) \lesssim E(\tau )+ E(0) \,, \end{aligned}$$
  where we emphasize that the constant *B* is independent of τ.

The three instances of our frequency analysis in the above list essentially yield the proof of our Main Theorem. Notice that (1.37) is already an integrated local energy decay statement as in our Main Theorem, though for solutions of the PDEs which are *a priori* assumed to be future integrable. Under the same assumption, (1.38) allows us to derive the energy flux boundedness. Indeed, suppose that we break up the solutions to the PDEs into *N* components, indexed by i=1,⋯,N, such that the *i*th component experiences trapping only in a small range of *r*-values (this is a frequency space construction!). Then, for each *i* we can construct a vector field which is timelike in the black hole exterior and has good commutation properties with $\mathfrak {R}^{[{s}]}$ in this trapped set so that in

$$\begin{aligned} E_{[-R^*,R^*]}(\tau )\leqq \sum _{i=1}^N E^{(i)}_{[-R^*,R^*]}(\tau )&\lesssim _{R^*} \sum _{i=1}^N I^{\textrm{deg},(i)}_{[-R^*,R^*]}(-\infty ,\infty ) \\&= I^{\textrm{deg}}_{[-R^*,R^*]}(-\infty ,\infty ) \lesssim _{R^*} E(0)\,, \end{aligned}$$

the second inequality holds. We can view this inequality as a *microlocal* (in the sense that it is partly frequency space, partly physical space) way of expressing the disjointness of trapping and superradiance. Note that, in the above, the first inequality is just the triangle inequality, the equality is due to Plancherel and we finally conclude from (1.38). For |a|<M, obtaining this bound is only nontrivial aspect of the energy boundedness problem: the nondegenerate horizon (for $r\rightarrow r_+$) and asymptotic flatness (for $r\rightarrow \infty$) of Kerr yield that the flux through the large $|r^*|$ part of $\Sigma _\tau$ is easily controlled.

To conclude the proof of our Main Theorem, we use (1.39) to argue that *a priori* assuming solutions of the relevant PDE to be *future* integrable is without loss of generality. This is clearly true in the case of Schwarzschild, a=0, and even very slowly rotating Kerr, |a|≪M, for which we have

$$\begin{aligned} E(\tau ) \lesssim E(0)+ |a|{I}^\textrm{deg}(0,\tau ) \lesssim E(0)\,, \end{aligned}$$

from (1.39). For general |a|<M, the above is replaced by

$$\begin{aligned} E^0(\tau ) \lesssim E^0(0)+ {I}^{\textrm{deg}, 1}(0,\tau ) \,, \end{aligned}$$

where a numerical subscript J=0,1,⋯ denotes a Sobolev norm whose regularity is *J* derivatives above the standard norms above; this estimate clearly loses derivatives. However, from (1.39), noting that the loss at trapping is only of half a derivative, and recalling that the obstacle to *a priori* energy boundedness is the ergorregion, we can show that

$$\begin{aligned} E^{\textrm{deg}, J}(0,\tau ) \lesssim _m I^{\textrm{deg}, 0}(0,\tau ) \,,\quad \forall J\gg 1 \end{aligned}$$

for any solutions of our PDE which are assumed to be supported on a fixed azimuthal *m* mode. This estimate allows us to gain back a large number of derivatives and conclude our density argument for fixed *m*. By the orthogonality of *m* modes, we deduce that the property that general solutions to the Teukolsky equation (1.2) are future integrable holds for the entire subextremal |a|<M Kerr family.

### Related works and further reading

Our work comes after more than a decade of intense research on stability of Schwarzschild and Kerr, of which we now give a brief account, using the Teukolsky equation (1.2) as a reference.

#### Schwarzschild (a=0) and subextremal (|a|<M) Kerr

The wave equation case, s=0, which can already be seen as the “poor man’s stability problem,” was settled in the full subextremal range |a|<M by Dafermos, Rodnianski and the first author in [57], where boundedness and decay of (1.2) were established. The first result has also been generalized to the subextremal Kerr–Newman family of charged, rotating black holes in [39]. We also note the earlier results on the Schwarzschild family which are summarized in the lecture notes [56], and their extensions to very slowly rotating |a|≪M Kerr in the independent works [7, 54, 160]. For applications to nonlinear wave equations, see for instance [50, 98, 113–115, 117, 135, 158].

For electromagnetic perturbations, s=±1, the work of [137] to which we have alluded was preceded by a proof, using different methods, of linear stability to perturbations of Cauchy data by Blue [25] (see also [153]). We also note the extensions to very slowly rotating Kerr [8, 118]. Pasqualotto’s work on the s=±1 the linear setting was the basis for his resolution of a nonlinear problem, the analogue of Question I for the Maxwell–Born–Infeld model of nonlinear electromagnetism [136]. Moreover, together with [48], to which we return below, it was an important ingredient in the proof of linear stability for the charged version of Schwarzschild (a=0), known as Reissner–Nordström spacetime, in [74, 75].

Gravitational perturbations, s=±2, have received the most attention in recent years. As we mentioned, boundedness and decay of (1.2) was first established in [48], who also used this fact to derive linear stability for the entire system of perturbations in a well-posed double null gauge. Later, Johnson established the linear stability of Schwarzschild in a generalized wave gauge [100] (see also the more recent [94, 95]); approaches based on metric perturbations were developed in [86]. More recently, boundedness and decay of $\upalpha ^{[{\pm 2}]}$ was generalized to very slowly rotating Kerr by Dafermos, Holzegel and Rodnianski [47] and Ma [119] independently. [10] have constructed a gauge in which these results could be exploited to obtain estimates for the rest of the metric components in the linearized setting. Using different methods, [82] have also shown decay for linearized metric perturbations in a generalized wave gauge for very slowly rotating, |a|≪M, Kerr spacetimes. We note also the papers [68, 69]. Finally, we of course emphasize again that the linear analyses mentioned here have recently been bootstrapped into full nonlinear stability proofs of stability of the |a|≪M Kerr subfamily, and we direct the reader to the preamble of this introduction for more precise statatements and references.

Gravitational and electromagnetic perturbations should be considered together for perturbations of electrovacuum spacetimes, to that is in the analogue of Question I for the Kerr-Newman family of black holes. For the spherically symmetric Reissner–Nördstrom subfamily, a proof of linear stability was carried out by Giorgi in her thesis [73–75]. More recently, the problem of linear stability for very slowly rotating Kerr-Newman has been independently tackled by Giorgi [77] and He [81] using different methods.

For results concerning the half-integer spin cases $s=\pm 1/2,\pm 3/2$ of the Teukolsky equation (1.2), see [88, 124] and references therein, respectively.

The aforementioned linear results are, for the most part, concerned with boundedness and decay of solutions to wave-type equations such as (1.2), but where the emphasis is on obtaining sufficient decay for nonlinear applications rather than uncovering what is the sharp decay, or Price’s law [138], that the linear models allow. However, motivated by the problem of strong cosmic censorship (see, for example the introduction of [51]), there has been a great deal of development in the direction of Price’s law. The seminal paper in this direction is due to Dafermos–Rodnianski [52]. For sharp decay estimates for solutions to the wave equation on the subextremal Kerr black hole family, see [2, 6, 97] and references therein. We also mention again the sharp decay results of Ma and Zhang [131, 132] and, using different methods, of Millet [123] for the s≠0 Teukolsky equation (1.2).

Finally, let us note that most of the above references refer to stability analyses for perturbations of Cauchy data under (1.1) and various linear models. Questions relating to stability for scattering data are not yet fully understood even for Minkowski spacetime in 3+1 dimensions (though note [165] for 4+1 and higher dimensions). Nevertheless, in the linear setting, Masaood has obtained a scattering theory for the linearized (1.1) around Schwarzschild a=0 in [120, 121]. In the full subextremal range |a|<M, a scattering theory has been obtained only in the scalar wave case [58], though see our preprint [151] for uniform-in-frequency scattering estimates (see also [71] for an earlier, fixed *m*, scattering theory for very slowly rotating Kerr-dS). We also point out work [46], where the authors construct a nontrivial family of spacetimes solving (1.1) whose exterior geometry settles down to that of a fixed Kerr black hole.

#### A remark on extremal (|a|=M) Kerr

Conspicuously absent from most of the previous discussion is the extremal Kerr case, |a|=M. In fact, as we have said, it is not clear whether in this limiting case boundedness and decay for (1.2), let alone Question I, should hold: already in the linearized setting, a mixture of stability and instability results have been obtained. In [20], Aretakis showed that higher order transversal derivatives of axisymmetric solutions to the wave equation along the event horizon blow up, a phenomenon that is known as Aretakis instability; the result was extended to the Teukolsky equation (1.2) for general integer spin in [112]. In spite of this instability, axisymmetric solutions to the wave equation, s=0, are bounded and do decay, albeit at a slower rate than in the subextremal, |a|<M, case [19]. In recent work, Gajic [70] has shown that for nonzero azimuthal modes there is an instability which sets in even at a lower level of differentiability than in the axismmetric case, leading to an even slower decay rate for solutions to the wave equation s=0 conjectured in the physics literature [32], see also [11]. This analysis makes use of the mode stability of extremal Kerr [155].

A lot of progress has been made on spherically symmetric toy model for extremal Kerr, extremal Reissner–Nordström, in the case of the scalar wave equation, s=0. Aretakis in [17, 18] and Aretakis, Angelopoulos and Gajic in [3] have have studied the Cauchy problem and scattering, respectively. As expected for Kerr, the sharp decay rates for the wave equation, obtained by the aforementioned authors in [4], are significantly slower than for subextremal Reissner–Nordström. Apetroaie [16] has extended the analysis to the gauge-invariant components of linearized gravitational and electromagnetic perturbations. It is unclear what the implications of the slow decay and Aretakis instability in the linear setting could be for the full nonlinear problem as described in Question I; see however [5] for stability and instability results concerning nonlinear wave equations satisfying the null condition on the extremal Reissner–Nordström black hole background, the numerics [129] concerning nonlinear stability of the extremal Reissner–Nordström exterior in the Einstein-scalar field model, and Conjecture IV.2 in [49].

#### Nonzero cosmological constant

To conclude the discussion of work inspired by Question I, it is important to note that this question has also been studied for solutions to the Einstein equations with a cosmological constant, Λ, to that is

1.40 $$\begin{aligned} \textrm{Ric}(g)=\Lambda g\,. \end{aligned}$$

In the case Λ>0, stability of the trivial solution of (1.40), known as de Sitter space, under perturbations of Cauchy data was shown in [67]. The problem is considerably easier than the stability of Minkowski settled in [40], as Λ>0 ensures faster decay rates for perturbations than for Λ=0. A breakthrough work of Hintz and Vasy [96] (see also [63, 64, 122]) showed the same is true for the slowly rotating Kerr–de Sitter family, the Λ>0 cousin of Kerr family, thus answering the Λ>0 analogue of Question I in the affirmative. It is conjectured that stability holds for the entire subextremal Kerr-de Sitter family. In [140], Petersen and Vasy showed decay for the wave equation on the full subextremal Kerr-de Sitter spacetime assuming a mode stability statement and making use of the methods introduced by Vasy in [161]. Mode stability is still open in the case Λ>0 and general subextremal *a*; it is known only in perturbative regimes, for instance for $|a|\ll \Lambda ,M$ [59], exploiting “closeness” to Schwarzschild-de Sitter, and for $M^2\Lambda \ll 1$ [84], exploiting “closeness” to (asymptotically flat) Kerr.

In the case Λ<0, the initial value problem for the Einstein equations (1.40) requires an additional boundary condition at infinity. Assuming reflecting boundary conditions at conformal infinity, one expects instability rather than stability already for the trivial solution, known as Anti-de Sitter space [45]. This has indeed been proven for (1.40) coupled to several spherically symmetric matter models by Moschidis [127, 128]. In the linear setting, [91] has shown that the (massive) scalar wave equation decays on Kerr-AdS spacetimes under the Hawking–Real bound [90] decay, though only very slowly due to the presence of *stably* trapped geodesics. In view of [72], this result is likely to be extended to the s≠0 Teukolsky equations, at least for very slowly rotating Kerr-Anti de Sitter. Several nonlinear toy model equations on these black hole spacetimes have been studied in the mathematics and physics literature, see for example [42] and references therein. In contrast, for dissipative boundary conditions at conformal infinity, one expects stability and we direct the reader to [87] for some linear results in this direction.

### Outline and acknowledgments

The set of this paper is organized as follows

- **Sections 2, 3 and 4.** These are very introductory sections, which the reader acquainted with the topics discussed in Sections 1.1 and 1.2 of this introduction can consider skipping. In Section 2, we recall to the reader the Kerr background geometry and fix our notation for relevant vector fields, operators and other shorthand notation. We also introduce the class of smooth *s*-spin-weighted functions, allowing us to make sense of (1.2). In Section 3, we introduce the main PDEs we study, the Teukolsky equation and the transformed system. In Section 4, we derive the angular and radial ODEs associated to the differential equations of the previous section under the separability assumption. We also perform a crude analysis of the angular ODE which allows us to obtain rudimentary bounds for the separation constant Λ in terms of the remaining frequency parameters, ω and *m*. For the radial ODEs, we discuss the boundary conditions for the solutions we seek to consider and we recall the precise statement of the radial Teukolsky–Starobinsky identities.
- **Section 5.** We introduce a series of tools for our analysis of the relevant ODEs. For instance, we introduce multiplier currents, such as virial currents, the classical energy currents from scalar wave analysis, and the Teukolsky–Starobinsky energy current discussed in Section 1.2.2 of this introduction. These are tools which are essential to the analysis of the relevant radial ODEs in the ensuing section. We also state and prove basic transport estimates for the transformed system.
- **Section 6.** This is the main section of the paper, which is devoted to the proof of Theorem 1. A precise statement is given in Section 6.1. Section 6.2 then gives an overview of the proof, explaining the (logic behind the) partition of frequency space into the many distinct frequency regimes which we will consider separately in our analysis, and roughly how different mulitpliers and techniques come together to yield the necessary estimates in each regime. We then turn to the proof of our main theorem which, as we justify in the aforementioned section, is divided into two broad classes of frequency regimes: for the bounded frequency regimes the theorem is shown in Section 6.4 and, for the high frequency regimes, in Section 6.3. The estimates for bounded and high frequency are combined in Section 6.5 to yield Theorem 1.

## The Kerr Spacetime

### The manifold and metric

In this section, we introduce the Kerr exterior. We refer the reader to [54, Section 2] for more detail.

For Kerr black hole parameters (*a*, *M*) satisfying M>0 and |a|≦M, we define

2.1 $$\begin{aligned} r_\pm :=M\pm \sqrt{M^2-a^2}. \end{aligned}$$

The Kerr exterior spacetime, $\mathcal {R}$, is a manifold-with-boundary which is covered by Kerr-star coordinates $(t^*,r,\theta ^*,\phi ^*)\in \mathbb {R}\times [r_+,\infty )\times \mathbb {S}^2$ globally, apart from the usual degeneration of spherical coordinates. The future event horizon is defined to be $\mathcal {H^+}:=\partial \mathcal {R}=\{r=r_+\}$.

When we are interested in $\textrm{int}(\mathcal {R})$ only, it will be convenient to consider Boyer–Lindquist coordinates $(t,r,\theta ,\phi )\in \mathbb {R}\times (r_+,\infty )\times \mathbb {S}^2$. These are obtained from Kerr-star coordinates by relations

2.2 $$\begin{aligned} t(t^*,r):=t^*-\overline{t}(r), \quad \quad \theta :=\theta ^*\,, \quad \quad \phi (\phi ^*,r):=\phi ^* -\overline{\phi }(r) \mod 2\pi ,\nonumber \\ \end{aligned}$$

for appropriate functions $\overline{\phi }$ and $\overline{t}(r)$, which can be defined so as to vanish for r≧9/4M (see [54, Section 2.4]). With respect to Boyer–Lindquist coordinates, the Kerr metric is given by

2.3 $$\begin{aligned} \begin{aligned} g_{a,M}&:= -\frac{\Delta -a^2\sin ^2\theta }{\rho ^2}\textrm{d}t^2- \frac{4Mar\sin ^2\theta }{\rho ^2} \textrm{d}t\textrm{d}\phi \\&\qquad +\left( \frac{(r^2+a^2)^2-\Delta a^2 \sin ^2\theta }{\rho ^2}\right) \sin ^2\theta \textrm{d}\phi ^2 +\frac{\rho ^2}{\Delta }\textrm{d}r^2+\rho ^2 \textrm{d}\theta ^2\,, \end{aligned} \end{aligned}$$

where

2.4 $$\begin{aligned} \rho ^2:= r^2+a^2\cos ^2\theta , \quad \quad \Delta :=r^2-2Mr+a^2=(r-r_+)(r-r_-) . \end{aligned}$$

It will also be convenient to define a new radial coordinate $r^*:(r_+,\infty )\rightarrow (-\infty ,\infty )$ which is the unique function satisfying $r^*(3M)=0$ and

2.5 $$\begin{aligned} \frac{\textrm{d}r^*}{\textrm{d}r}=\frac{r^2+a^2}{\Delta } \text {, with } \Delta :=r^2-2Mr+a^2=(r-r_+)(r-r_-). \end{aligned}$$

Finally, throughout the paper, we rely on the notation

2.6 $$\begin{aligned} w:=\frac{\Delta }{(r^2+a^2)^2}\,, \end{aligned}$$

for an *r*-weight that will be heavily used.

### Relevant vector fields and differential operators

We will often use the shorthand notation $T:=\partial _{t^*}$ and $\Phi :=\partial _{\phi ^*}$, where $t^*$ and $\phi ^*$ are part of the Kerr-star system of coordinates. Moreover, letting

$$\begin{aligned} \upomega _+:=\frac{a}{2Mr_+}\,, \end{aligned}$$

the vector field $K:=T+\upomega _+\Phi$ is the null generator of the future event horizon, $\mathcal {H}^+$.

Recall the definition of $r^*$ in (2.5). In $(t,r^*,\theta ,\phi )$ coordinates, let

$$\begin{aligned} L:=\partial _{r^*}+T+\frac{a}{r^2+a^2}\Phi \,, \qquad \underline{L}:=-\partial _{r^*}+T+\frac{a}{r^2+a^2}\Phi \,, \end{aligned}$$

as define two principal null directions on Kerr, with the normalization condition $g(L,\underline{L})=-2\rho ^2w$.

## The Teukolsky Equation and the Transformed System

### Smooth spin-weighted functions

In this paper and in the companion [152], we consider partial differential equations whose unknown is a spin-weighted function. The present section will contain the definitions of the function spaces we will use. Here, $s\in \frac{1}{2}\mathbb {Z}$ is a parameter.

Letting $(\theta ,\phi ^*)$ denote standard spherical coordinates in $\mathbb {S}^2$, consider the vector fields

3.1 $$\begin{aligned} \begin{aligned} \tilde{Z}_1 = -\sin \phi ^* \partial _\theta + \cos \phi ^* \left( -is \csc \theta - \cot \theta \partial _{\phi ^*}\right) , \\ \tilde{Z}_2 = - \cos \phi ^* \partial _\theta - \sin \phi ^* \left( -is \csc \theta - \cot \theta \partial _{\phi ^*}\right) , \quad \tilde{Z}_3 = \partial _{\phi ^*} \,. \end{aligned} \end{aligned}$$

We recall the following notion of smooth *s*-spin-weighted function (see [47, Section 2.2.1]):

#### Definition 3.1

*(Smooth spin-weighted functions)* A 0-smooth weighted function is a smooth function. For s≠0, we have the following.

(i) Let *f* be a complex-valued function of $(\theta ,\phi ^*)\in \mathbb {S}^2$. We say *f* is a smooth *s*-spin-weighted function on $\mathbb {S}^2$, and write $f\in \mathscr {S}^{[{s}]}_\infty$, if for any $k_1,k_2,k_3 \in \mathbb {N}_0$,
  $$\begin{aligned} (\tilde{Z}_1)^{k_1} (\tilde{Z}_2)^{k_2} (\tilde{Z}_3)^{k_3} f \end{aligned}$$
  is a function of $(\theta ,\phi ^*)$ which is smooth for $\theta \ne 0,\pi$ and such that
  $$\begin{aligned} e^{is\phi }(\tilde{Z}_1)^{k_1} (\tilde{Z}_2)^{k_2} (\tilde{Z}_3)^{k_3} f \text { and }e^{-is\phi }(\tilde{Z}_1)^{k_1} (\tilde{Z}_2)^{k_2} (\tilde{Z}_3)^{k_3} f \end{aligned}$$
  extend continuously to, respectively, θ=0 and $\theta =\pi$.
(ii) Now, fix Kerr parameters M>0 and |a|≦M. Consider a function *f* of the Kerr-star variables $(t^*,r,\theta ,\phi ^*)\in \mathbb {R}\times [r_+,\infty )\times \mathbb {S}^2$. We say *f* is a smooth *s*-spin-weighted function on $\mathcal {R}$, and write $f\in \mathscr {S}_\infty ^{[s]}(\mathcal {R})$, if for any $k_4,k_5 \in \mathbb {N}_0$, the function
  $$\begin{aligned} (\partial _{t^*})^{k_4} (\partial _{r})^{k_5} f\Big |_{(t^*,r)=(T^*,R)} \end{aligned}$$
  is a smooth *s*-spin-weighted function on $\mathbb {S}^2$ for any $T^*\in \mathbb {R}$ and $R\in [r_+,\infty )$.

#### Examples 3.2

The spin-weighted laplacian3.2where $\tilde{Z}_1$, $\tilde{Z}_2$ and $\tilde{Z}_3$ are defined in (3.1) and the spinorial gradient3.3are smooth differential operators on $\mathscr {S}_\infty ^{[s]}$.

The angular operatoris a smooth differential operator on $\mathscr {S}_\infty ^{[s]}(\mathcal {R})$.

### The Teukolsky equation

We say $\upalpha ^{[{s}]}$ is a smooth solution of the *inhomogeneous* Teukolsky equation if $\Delta ^s\upalpha ^{[{s}]}\in \mathscr {S}_\infty ^{[s]}(\mathcal {R})$ and

3.4 $$\begin{aligned} \rho ^{2}\mathfrak {T}^{[s]}\upalpha ^{[s]}=F^{[{s}]}, \end{aligned}$$

for some $\Delta ^{1+|s|}F^{[{s}]}\in \mathscr {S}_\infty ^{[s]}(\mathcal {R})$, where $\mathfrak {T}^{[s]}$ is a differential operator given by3.5We call (3.4) a homogeneous equation if $F^{[{s}]}$ vanishes.

### The transformed system

In this section, based on the framework of [92] and inspired by the transformations of Dafermos, Holzegel and Rodnianski in [47, 48], we introduce a system of equations derived from the Teukolsky equation (3.4). To discuss this transformed system, it will be useful to have the following notation

$$\begin{aligned} \mathcal {L} = {\left\{ \begin{array}{ll} L, \quad & s<0\\ \underline{L}, \quad & s>0 \end{array}\right. }\,,\quad \underline{\mathcal {L}} = {\left\{ \begin{array}{ll} \underline{L}, \qquad & s<0\\ L, \quad & s>0 \end{array}\right. }, \end{aligned}$$

for the null vector fields introduced in Section 2.2 when acting on the space of smooth *s*-spin weighted functions, as introduced in Definition 3.1, with s≠0.

#### Proposition 3.3

Fix $s\in \mathbb {Z}$. Let $\upalpha ^{[{s}]}$ be a solution to the inhomogeneous Teukolsky equation (3.4) with inhomogeneity $F^{[{s}]}$. Let $\uppsi ^{[{s}]}_{(0)}$ and $\mathfrak G^{[{s}]}_{(0)}$ be rescaling of these functions given by

3.6 $$\begin{aligned} \uppsi ^{[{s}]}_{(0)}&:=(r^2+a^2)^{-|s|+1/2}\Delta ^{|s|/2(1+\textrm{sign}\,s)}\alpha ^{[{s}]}\,, \end{aligned}$$

3.7 $$\begin{aligned} \mathfrak G^{[{s}]}_{(0)}&:=w(r^2+a^2)^{-|s|+1/2}\Delta ^{|s|/2(1+\textrm{sign}\,s)}F^{[{s}]}\,. \end{aligned}$$

For k=1,⋯,|s|, let $\uppsi ^{[{s}]}_{(k)}$ and $\mathfrak G^{[{s}]}_{(k)}$ be defined by the system of transport equations

3.8 $$\begin{aligned} \uppsi ^{[{s}]}_{(k)}&:=\frac{1}{w}\mathcal {L}\uppsi ^{[{s}]}_{(k-1)}\,, \end{aligned}$$

3.9 $$\begin{aligned} \mathfrak G^{[{s}]}_{(k)}&:=\mathcal {L}\left(w^{-1}\mathfrak G^{[{s}]}_{(k-1)}\right)\,. \end{aligned}$$

Then, for k=0,...,|s|, $\uppsi ^{[{s}]}_{(k)}$ satisfy

3.10 $$\begin{aligned} \mathfrak {R}^{[{s}]}_{(k)}\uppsi ^{[{s}]}_{(k)}={{\,\textrm{sign}\,}}s (|s|-k)w'\uppsi ^{[{s}]}_{(k+1)}+\mathfrak {J}^{[{s}]}_{(k)}+\mathfrak {G}^{[{s}]}_{(k)}, \end{aligned}$$

where3.11

3.12 $$\begin{aligned}&\qquad \qquad \qquad + \frac{a^2\Delta ^2}{(r^2+a^2)^4}\left[1-2|s|-2k(2|s|-k-1)\right] \nonumber \\&\qquad \qquad \qquad +\frac{2Mr(r^2-a^2)\Delta }{(r^2+a^2)^4}\left[1-3|s|+2s^2-3k(2|s|-k-1)\right],\nonumber \\&\qquad \quad \mathfrak {J}^{[{s}]}_{(k)}:=-aw\sum _{j=0}^{k-1}\left(c_{s,\,k,\,j}^{\Phi }(r)\Phi + c_{s,\,k,\,j}^{\textrm{id}}(r)\right)\uppsi ^{[{s}]}_{(j)}\,, \end{aligned}$$

where $\mathfrak {J}^{[{s}]}_{(0)}\equiv 0$, and where for j=0,⋯,k and k=1,⋯,|s|, $c_{s,\,k,\, j}^\textrm{id}$ and $c_{s,\,k,\,j}^\Phi$ are functions of *r* which satisfy the following conditions:

- $a c_{s,\,k,\,j}^\textrm{id}$ should be replaced by $c_{s,\,k,\,j}^\textrm{id}$ if and only if |s|≧2, $1\leqq k\leqq |s|-1$ and j=k-1;
- $c_{s,\,k,\, j}^\textrm{id}$ and $c_{s,\,k,\,j}^\Phi$ can be explicitly computed through a recursive relation (3.17) initialized by (3.18);
- $c_{s,\,k,\, j}^\textrm{id}$ and $c_{s,\,k,\,j}^\Phi$ are, at most, of *O*(1) as $r^*\rightarrow \pm \infty$, their derivatives with respect to $r^*$ are, at most, of *O*(*w*) as $r^*\rightarrow \pm \infty$, and for 1≦k<|s| we have $(a c_{s,\,k,\,k-1}^\textrm{id})'=|a| O(w)$ as $r^*\rightarrow \pm \infty$.

In particular, $\Psi ^{[{s}]}:=\uppsi ^{[{s}]}_{(|s|)}$ solves the transformed equation

3.13 $$\begin{aligned} \mathfrak {R}^{[{s}]}\Psi ^{[{s}]}=\mathfrak {J}^{[{s}]}+\mathfrak {G}^{[{s}]}, \end{aligned}$$

where3.14

3.15 $$\begin{aligned} \mathfrak {J}^{[{s}]}&:=\mathfrak {J}^{[{s}]}_{(|s|)}= -\sum _{k=0}^{|s|-1} aw \left[ c^{\Phi }_{s,\,|s|,\,k}(r) \Phi +c^\textrm{id}_{s,\,|s|,\,k}(r)\right]\uppsi ^{[{s}]}_{(k)}\,. \end{aligned}$$

Finally, note that using (3.8), for k<|s|, we can recast (3.10) as the transport equation3.16

#### Proof

Assume s≠0; otherwise the result follows easily by a computation with the rescalings (3.6) and (3.7). The proof follows by a recursive procedure. Indeed, to obtain the wave equation (3.10) at *k*th level, we start from the (k-1)th wave equation (3.10). Using

$$\begin{aligned} \mathcal {L}\underline{\mathcal {L}}=\underline{\mathcal {L}}\mathcal {L}+[\mathcal {L},\underline{\mathcal {L}}]= \underline{\mathcal {L}}\mathcal {L}+{{\,\textrm{sign}\,}}s \frac{4arw}{r^2+a^2}\Phi , \end{aligned}$$

we simplify the terms containing two null derivatives using the definition of $\uppsi ^{[{s}]}_{(k)}$; we thus obtain the (k-1)th transport relation (3.16). Then, we divide by *w* and apply $\mathcal {L}$. We once again simplify terms using the definitions of $\uppsi ^{[{s}]}_{(j)}$, j=0,⋯,k, except for the terms with two null derivatives of $\uppsi ^{[{s}]}_{(k)}$. To deal with these, we note that

$$\begin{aligned} \underline{\mathcal {L}}\mathcal {L}=\frac{1}{2}\left(L\underline{L}+\underline{L}L\right)+{{\,\textrm{sign}\,}}s\frac{2raw}{r^2+a^2}\Phi \,. \end{aligned}$$

With our choice of weights, the PDEs for the transformed quantities take the form

$$\begin{aligned} \underline{\mathcal {L}}\mathcal {L}\uppsi ^{[{s}]}_{(k)} -{{\,\textrm{sign}\,}}s (|s|-k)\frac{w'}{w}\mathcal {L}\uppsi ^{[{s}]}_{(k)}+\sum _{X\in \mathfrak {X}} \sum _{i=0}^k c_{s,\,k,\,j}^X X \uppsi ^{[{s}]}_{(j)}=\mathfrak G_{(k)}, \end{aligned}$$

where  and coefficients $c^X_{s,\,k,\,j}$ satisfy the recursive relation

3.17 $$\begin{aligned} {\left\{ \begin{array}{ll} c_{s,\,k,\,0}^X& =-{{\,\textrm{sign}\,}}s \Big (\frac{c_{s,\,k-1,\,0}^X}{w}\Big )' \\ c_{s,\,k,\,j}^X& =-{{\,\textrm{sign}\,}}s\Big (\frac{c_{s,\,k-1,\,j}^X}{w}\Big )'+c_{s,\,k-1,\,j-1}^X \quad \text { for } j=1,...,k-1 \end{array}\right. }\,, \end{aligned}$$

initialized by the relations, for j=k=1,...,|s|,3.18The recursive relations for each $c_{s,\,k,\,j}^X$ can be used to reduce these coefficients, for any j=0,...,k-1, to a linear combination of $r^*$ derivatives, weighted by $w^{-1}$, of $c_{s,\,j,\,j}^X$, for j=0,...,k-1. We would like to check if, for j≦k-1,

3.19 $$\begin{aligned} c_{s,\,k,\,j}^X= a w \tilde{c}_{s,\,k,\,j}^X \end{aligned}$$

for some function $\tilde{c}_{s,\,k,\,j}^X$, which is at most of *O*(1) and $\left(\tilde{c}_{s,\,k,\,j}^X\right)'=O(w)$ as $r^*\rightarrow \pm \infty$. For , the claim clearly holds, sinceFor X=Φ, the claim follows from the simple computation

$$\begin{aligned}  &   \underbrace{\left(\frac{1}{w}\cdots \left(\frac{c_{s,\,k,\,k}^\Phi }{w}\right)'\cdots \right)'}_{n\,\text { weighted derivatives}} = -4^{\lfloor n/2\rfloor }(-1)^{\lceil n/2\rceil }{{\,\textrm{sign}\,}}s(2|s|-2k+1)a^nw{\left\{ \begin{array}{ll} \frac{r^2-a^2}{r^2+a^2} & \text { if }n \text { odd} \\ \frac{a r }{r^2+a^2} & \text { if } n\, \text {even} \end{array}\right. }. \end{aligned}$$

For $X=\textrm{id}$, we compute that

$$\begin{aligned} \frac{1}{w}\underbrace{\left(\frac{1}{w}\cdots \left(\frac{a^2 w'}{w}\right)'\cdots \right)'}_{n \text { weighted derivatives}}&= a^2 O(1) \text { as }r^*\rightarrow \pm \infty \,, \\ \frac{1}{w}\underbrace{\left(\frac{1}{w}\cdots \left(\frac{r(r^2-a^2)}{(r^2+a^2)^2}\right)'\cdots \right)'}_{n\, \text {weighted derivatives}}&= \frac{(-1)^{\lceil n/2\rceil }(16a^2)^{\lfloor n/2\rfloor }}{(r^2+a^2)^2}{\left\{ \begin{array}{ll} (r^4-6a^2r^2+a^4) & \text {if}\, n\, \text { odd} \\ r(r^2-a^2) & \text {if}\, n\, \text { even} \end{array}\right. }\,. \end{aligned}$$

By the recursive relations, we see that the only possible obstruction to the claim (3.19) is for $c_{s,\,k,\, k-1}^\textrm{id}$, when we take n=1 in the above equality and so $(16a^2)^{\lfloor n/2\rfloor } =1$. For s=±1, this is not an issue, however, as

$$\begin{aligned} c_{\pm 1,\,1,\, 0}^\textrm{id}=\left(\frac{c_{\pm 1,\, 0,\, 0}^\textrm{id}}{w}\right)'=-\frac{a^2 w'}{w} \,, \end{aligned}$$

since $$[1-3|s|+2s^2-3k(2|s|-k-1)]=0$$ for (s,k)=(±1,0). □

#### Remark 3.4

*(Computation of the auxiliary functions)* In Proposition 3.3, we have written the equations for the transformed system (3.10) and (3.13) in terms of some functions $c_{s,\,k,\,j}^\Phi$ and $c_{s,\,k,\,j}^\textrm{id}$, j<k=0,...,|s|, that can be explicitly computed: for j=k, these are given by (3.18), whereas for j=0,...,k-1 they are obtained from the recursive relation (3.17). We compute the resulting functions in the physically interesting cases |s|=1,2. For |s|=1,

$$\begin{aligned} ac_{\pm 1,\,1,\,0}^\textrm{id}=\pm \frac{2a(r^3-3Mr^2+a^2r+a^2M)}{(r^2+a^2)^2}\,, \qquad c_{\pm 1,\,1,\,0}^\Phi = -\frac{2(r^2-a^2)}{r^2+a^2}\,. \end{aligned}$$

For |s|=2, cf. [47, Eq. (57)],

$$\begin{aligned}&ac_{\pm 2,\,2,\,1}^\textrm{id}=\mp \frac{20a(r^3-3Mr^2+a^2r+a^2M)}{(r^2+a^2)^2}\,, \qquad  &   c_{\pm 2,\,2,\,1}^\Phi = - 8\frac{r^2-a^2}{r^2+a^2}\,,\\&ac_{\pm 2,\,2,\,0}^\textrm{id}=-\frac{6a^2(r^4+10Mr^3-6a^2Mr-a^4)}{(r^2+a^2)^2}\,, \qquad  &   c_{\pm 2,\,2,\,0}^\Phi = \pm 24\frac{a r}{r^2+a^2}\,,\\&ac_{\pm 2,\,1,\,0}^\textrm{id}=\pm \frac{6r(Mr^3-a^2r^2-3Mr a^2-a^4)}{(r^2+a^2)^2}\,, \qquad  &   c_{\pm 2,\,1,\,0}^\Phi =-6\frac{r^2-a^2}{r^2+a^2}\,. \end{aligned}$$

## The Teukolsky and the Transformed System of ODEs

In this section, we introduce the ordinary differential equations arising from a formal separation of variables of the Teukolsky equation (3.4) and of the transformed system introduced in Proposition 3.3.

### Admissible frequencies

For $\omega \in \mathbb {R}$ and $m\in \frac{1}{2}\mathbb {Z}$, it will be convenient to define that

4.1 $$\begin{aligned} \xi := -i\frac{2M r_+}{r_+-r_-}(\omega -m\upomega _+) ,\quad \beta :=2iM^2(\omega -m\upomega _+), \quad \upomega _+:=\frac{a}{2Mr_+}.\nonumber \\ \end{aligned}$$

For the remainder of this paper, we will be interested in the following parameters:

#### Definition 4.1

*(Admissible frequencies)* Fix $s\in \frac{1}{2}\mathbb {Z}$, M>0 and |a|≦M.

I. We say the frequency parameter *m* is admissible with respect to *s* if $m-s\in \mathbb {Z}$.
II. We say the frequency pair (*m*, *l*) is admissible with respect to *s* if *m* is admissible with respect to *s* and $l-\max \{|m|,|s|\}\in \mathbb {Z}_{\geqq 0}$.
III. We say the frequency triple (ω,m,l) is admissible with respect to *s* if the pair (*m*, *l*) is admissible with respect to *s* and $\omega \in \mathbb {R}$. We say it is admissible with respect to *s* and *a* if, additionally, $\omega \in \mathbb {R}$ with $\omega \in \mathbb {R}\backslash \{0\}$ and, if |a|=M, $\omega \ne m\upomega _+$. In this case, we write $(\omega ,m,l)\in \mathcal {F}_{\textrm{ admiss}(a,M,s)}$.
IV. We say the frequency triple $(\omega ,m,\Lambda )$ is admissible with respect to *s* if *m* is admissible with respect to *s*, if $\omega \in \mathbb {R}$ with $\omega \in \mathbb {R}\backslash \{0\}$ and, if |a|=M, $\omega \ne m\upomega _+$, and if $\Lambda \in \mathbb {R}$ satisfies the following conditions in terms of *m* and aω:
  - if s=0, $$\Lambda \geqq 2|am\omega |$$ and $$\Lambda \geqq m^2$$;
  - $$\Lambda \geqq \max \{|m|,|s|\}(\max \{|m|,|s|\}+1)-s^2-2|s||a\omega |$$;
  - $$|\Lambda | \leqq \Lambda +s^2+a^2\omega ^2+4|s||a\omega |$$;
  - assuming q:=aω/m, if q<0 then, for sufficiently large |*m*|, $\Lambda -2am\omega \geqq \frac{1}{2} |q|m^2$; if $0\leqq q\leqq \frac{1}{2}$ then, for sufficiently large |*m*| and $|m|q^2$, $\Lambda \geqq m^2$.
  In this case, we write $(\omega ,m,\Lambda )\in \mathcal {F}_{\textrm{ admiss}(a,M,s)}$.

### The angular ODE

In this section, we introduce the ODE to be satisfied by the angular part of the Teukolsky variable under separation of variables, and derive some of the properties of the separation constant.

#### Proposition 4.2

(Smooth spin-weighted spheroidal harmonics) Fix $s\in \frac{1}{2}\mathbb {Z}$, let *m* be admissible with respect to *s*, and assume $\nu \in \mathbb {R}$. Consider the angular ODE

4.2 $$\begin{aligned} \begin{aligned}&-\frac{1}{\sin \theta }\frac{\textrm{d}}{\textrm{d}\theta }\left(\sin \theta \frac{\textrm{d}}{\textrm{d}\theta }\right)S_{m,\boldsymbol{\uplambda }}^{[s],\,(\nu )}(\theta )\\&+ \left(\frac{(m+s\cos \theta )^2}{\sin ^2\theta }-\nu ^2\cos ^2\theta +2\nu s \cos \theta \right)S_{m,\boldsymbol{\uplambda }}^{[s],\,(\nu )}(\theta )\\&=\boldsymbol{\uplambda }_{m}^{[s],\,(\nu )} S_{m,\boldsymbol{\uplambda }}^{[s],\,(\nu )}(\theta ) , \end{aligned} \end{aligned}$$

with the boundary condition that $e^{im\phi }S_{m,\boldsymbol{\uplambda }}^{[s],\,(\nu )}$ is a non-trivial smooth *s*-spin-weighted function (on $\mathbb {S}^2$, see Definition 3.1).

For each $\nu \in \mathbb {R}$, there are countably many solutions to the problem; using $l \in \mathbb {Z}_{\geqq \max \{|m|,|s|\}}$ as an index, an orthonormal (with respect to $L^2(\sin \theta \textrm{d}\theta \textrm{d}\phi )$) basis for the solutions is provided by the so-called *s*-spin-weighted spheroidal harmonics with spheroidal parameter ν, denoted by $e^{im\phi }S_{ml}^{[s],\,(\nu )}$; we denote the corresponding eigenvalues by $\boldsymbol{\uplambda }_{ml}^{[s],\,(\nu )}$. The parameter *l* can be chosen so that $\boldsymbol{\uplambda }_{ml}^{[s],\,(0)}=l(l+1)-s^2$ for ν=0 and $\boldsymbol{\uplambda }_{ml}^{[s],\,(\nu )}$ varies smoothly with ν. The eigenvalues satisfy

4.3 $$\begin{aligned} \boldsymbol{\uplambda }^{[-s],\,(-\nu )}_{-m,\,l}=\boldsymbol{\uplambda }^{[s],\,(\nu )}_{ml}=\boldsymbol{\uplambda }^{[-s],\,(\nu )}_{ml}\,. \end{aligned}$$

Finally, $\left\rbrace e^{im\phi }S_{ml}^{[s],\,(\nu )}\right\lbrace _{ml}$ form a complete basis for $\mathscr {S}^{[s]}(\infty )$.

#### Proof

For $\nu \in \mathbb {R}$, the operatoris self-adjoint and it follows from Sturm-Liouville theory that it has a countable set of eigenfunctions, which form a complete basis of the space of *s*-spin-weighted spheroidal functions, and countable set of corresponding eigenvalues (see, for instance, [47]). For each *s*, *m* and ν, these can be indexed by $l\in \mathbb {Z}_{\max \{|s|,|m|\}}$ so that, in particular, $\lambda _{ml}^{[s],\,(\nu )}$ is smooth in ν and $\boldsymbol{\uplambda }_{ml}^{[s],\,(0)}=l(l+1)-s^2$ for ν=0 (see [130, Section 3.22, Proposition 1]). The invariance of  under the map $(m,s,\nu )\mapsto (-m,-s,-\nu )$ establishes the first equality in (4.3), and the invariance under $(s,\theta )\mapsto (-s, \pi -\theta )$ establishes the second (see [139, Section IIIa)]). □

#### Lemma 4.3

(Properties of the angular eigenvalues) Let $\boldsymbol{\Lambda }_{ml}^{[s],\,(\nu )}$ be defined by

4.4 $$\begin{aligned} \boldsymbol{\Lambda }^{[s],\,(\nu )}_{ml}:=\boldsymbol{\uplambda }^{[s],\,(\nu )}_{ml}+\nu ^2 \end{aligned}$$

where $\boldsymbol{\uplambda }^{[s],\,(\nu )}_{ml}$ is the angular eigenvalue identified in Proposition 4.2.

- $\boldsymbol{\Lambda }^{[s],\,(\nu )}_{ml}$ depends only on *l*, *m*, ν and |*s*|.
- Basic bounds for s=0: $\boldsymbol{\Lambda }^{[0],\,(\nu )}_{ml} \geqq 2|m\nu |$ and $\boldsymbol{\Lambda }^{[0],\,(\nu )}_{ml} \geqq m^2$.
- Basic bounds for general |*s*|: $\boldsymbol{\Lambda }^{[s],\,(\nu )}_{ml} \geqq l(l+1)-s^2-2|s||\nu |$ and $\left|\boldsymbol{\Lambda }^{[s],\,(\nu )}_{ml}\right| \leqq \boldsymbol{\Lambda }^{[s],\,(\nu )}_{ml}+s^2+\nu ^2+4|s||\nu |$. label
- Set q=ν/m. If q<0 then, for sufficiently large |*m*|, $\boldsymbol{\Lambda }^{[s],\,(\nu )}_{m l} -2\,m\nu \geqq \frac{1}{2} |q|m^2$; if $0\leqq q\leqq \frac{1}{2}$ then, for sufficiently large |*m*| so that $|s|/|m|\leqq q^2$, $\boldsymbol{\Lambda }^{[s],\,(\nu )}_{m l}\geqq m^2$.

Setting $\nu =a\omega$, Lemma 4.3 provides a justification for condition IV of Definition 4.1.

#### Proof (Proof of Lemma 4.3)

Proposition 4.2 immediately implies (a) as well as the second bound in (b).

For the remaining properties, it is useful to consider the variational definition of $\boldsymbol{\Lambda }^{[s],\,(\nu )}_{ml}$: let $\Xi ^{[{s}]}$ be an *s*-spin-weighted spheroidal harmonic normalized to have unit $L^2(\sin \theta \textrm{d}\theta \textrm{d}\phi )$ norm (see Proposition 4.2); then, by construction, $\boldsymbol{\Lambda }^{[s],\,(\nu )}_{ml}$, defined in (4.4), satisfies

4.5 $$\begin{aligned} \begin{aligned} \boldsymbol{\Lambda }^{[s],\,(\nu )}_{ml}=\int _0^\pi \int _0^{2\pi } \left(\left|\partial _\theta \Xi ^{[{s}]}\right|^2+V_{\textrm{ang}}(\theta )\left|\Xi ^{[{s}]}\right|^2\right)\sin \theta \textrm{d}\theta \textrm{d}\phi , \end{aligned} \end{aligned}$$

where, setting q=ν/m

4.6 $$\begin{aligned} \begin{aligned} V_{\textrm{ang}}(\theta )&=\frac{(m+s\cos \theta )^2}{\sin ^2\theta } +\nu ^2\sin ^2\theta +2\nu s \cos \theta \\&= \frac{m^2}{\sin ^2\theta }(1-q\sin ^2\theta )^2+\frac{2sm\cos \theta }{\sin ^2\theta }(1+q\sin ^2\theta ) +\frac{s^2\cos ^2\theta }{\sin ^2\theta }+2m\nu . \end{aligned} \end{aligned}$$

In the case ν=0 (see Proposition 4.2), $\boldsymbol{\Lambda }^{[s],\,(\nu )}_{ml}=l(l+1)-s^2$. Hence, as $V_{\textrm{ang}}=V_{\textrm{ang}}|_{\nu =0}+ \nu ^2\sin ^2\theta +2\,s\nu \cos \theta$, we can immediately obtain the first bound in (c), and the second then follows easily:

$$\begin{aligned} \left|\boldsymbol{\Lambda }^{[s],\,(\nu )}_{ml}\right|\leqq l(l+1)+\nu ^2+2|s||\nu |\leqq \boldsymbol{\Lambda }^{[s],\,(\nu )}_{ml}+s^2+\nu ^2+4|s||\nu |,. \end{aligned}$$

The first bound in (b) follows from the fact that, if s=0, Young’s inequality gives

$$\begin{aligned} V_{\textrm{ang}}(\theta )=\frac{m^2}{\sin ^2\theta } +\nu ^2\sin ^2\theta \geqq 2|m\nu | . \end{aligned}$$

Finally, we turn to (d). For q<0, we have

$$\begin{aligned} V_{\textrm{ang}}(\theta )-2m\nu&\geqq 2s\nu \cos \theta -2m\nu = 2|q|(m^2-sm \cos \theta )\geqq |q|m^2\,, \text { if } |m|\geqq 2|s|\,,\\ V_{\textrm{ang}}(\theta )-2m\nu&\geqq \frac{s^2}{\sin ^2\theta } \left[2^2 (1+|q|\sin ^2\theta )^2-2\times 2\max \{1,|q|\sin ^2\theta \}\right]\\&\geqq 4s^2|q| \geqq |q|m^2\,, \text { if }|m|\leqq 2|s|\,, \end{aligned}$$

where we have used the first two lines of (4.6), respectively. In case q∈[0,1], we have

$$\begin{aligned}  &   |(1+q\sin ^2\theta )\cos \theta |\\  &   \leqq {\left\{ \begin{array}{ll} 1, & q\in \left[0,\frac{1}{2}\right] \\ \frac{2}{3\sqrt{3}}\sqrt{\frac{(q+1)^3}{q}}\leqq \frac{4\sqrt{2}}{3\sqrt{3}}\frac{1}{\sqrt{q}}, & q\in \left[\frac{1}{2},1\right] \end{array}\right. },\\  &   \qquad (q-1)^2\leqq \frac{(1-q\sin ^2\theta )^2}{\sin ^2\theta }\leqq \frac{1}{\sin ^2\theta }, \end{aligned}$$

and thus we easily obtain from the last line of (4.6) that

$$\begin{aligned} V_{\textrm{ang}}(\theta )\geqq 2qm^2+ (q-1)^2m^2\left(1-\left\rbrace \begin{array}{lr} 1, & q\in \left[0,\frac{1}{2}\right]\\ \frac{4\sqrt{2}}{3\sqrt{3}}\frac{1}{\sqrt{q}}, & q\in \left[\frac{1}{2},1\right] \end{array}\right\lbrace \frac{|s|}{|m|}\right)\geqq m^2\,, \end{aligned}$$

as long *m* is sufficiently large depending on *s*, *q* and 1-q that

$$\begin{aligned} \frac{|s|}{|m|}\leqq \left\rbrace \begin{array}{lr} 1, & q\in \left[0,\frac{1}{2}\right]\\ \frac{4\sqrt{2}}{3\sqrt{3}}\frac{1}{\sqrt{q}}, & q\in \left[\frac{1}{2},1\right] \end{array}\right\lbrace ^{-1}\min \left\rbrace 1, \frac{q^2}{(1-q)^2}\right\lbrace \leqq {\left\{ \begin{array}{ll} \frac{q^2}{(1-q)^2}\leqq 4q^2, & q\in \left[0,\frac{1}{2}\right]\\ \frac{3\sqrt{3}}{4\sqrt{2}}\sqrt{q}, & q\in \left[\frac{1}{2},1\right] \end{array}\right. }. \end{aligned}$$

This concludes the proof. □

### The radial ODEs

In this section, we introduce the radial ODEs whose analysis is the main goal of the present paper: the Teukolsky radial ODE and some of its properties are given in Section 4.3.1, while a transformed system of radial ODEs is given in Section 4.3.2. Here and throughout the remainder of the paper, by abuse of notation, we write *L* and $\underline{L}$ are for the separated picture analogues of the vector fields thus far considered:

$$\begin{aligned} L=\frac{\textrm{d}}{\textrm{d}r^*}-i\omega +\frac{iam}{r^2+a^2}\,,\quad \underline{L}=-\frac{\textrm{d}}{\textrm{d}r^*}-i\omega +\frac{iam}{r^2+a^2}\,. \end{aligned}$$

#### The Teukolsky radial ODE and its boundary conditions

For some spin $s\in \mathbb {Z}$ and a∈[-M,M], let $(\omega ,m,\Lambda )$ be an admissible frequency triple with respect to *a* and *s*. We say $\upalpha _{m\Lambda }^{[{s}],\,a,\,\omega }(r)$ is a solution fo the the inhomogeneous Teukolsky radial ODE if

4.7 $$\begin{aligned}&\left[\Delta ^{-s}\frac{\textrm{d}}{\textrm{d}r}\left(\Delta ^{s+1}\frac{\textrm{d}}{\textrm{d}r}\right) +\frac{[\omega (r^2+a^2)-am]^2-2is(r-M)[\omega (r^2+a^2)-am]}{\Delta }\right] \upalpha _{m\Lambda }^{[{s}],\,a,\,\omega }(r)\nonumber \\&\qquad \quad +\left(4is\omega r -\Lambda -s+2am\omega \right)\upalpha _{m\Lambda }^{[{s}],\,a,\,\omega }(r)=\frac{\Delta ^{|s|/2(1-{{\,\textrm{sign}\,}}s)}}{(r^2+a^2)^{|s|}}F_{m\Lambda }^{[{s}],\,a,\,\omega }(r)\,, \end{aligned}$$

for a given inhomogenity $F_{m\Lambda }^{[{s}],\,a,\,\omega }(r)$. Defining

4.8 $$\begin{aligned} u_{m\Lambda }^{[{s}],\,a,\,\omega }:=\Delta ^{s/2}(r^2+a^2)^{1/2}\upalpha _{m\Lambda }^{[{s}],\,a,\,\omega }, \end{aligned}$$

the Teukolsky radial ODE (4.7) can also be rewritten as

4.9 $$\begin{aligned} \left(u_{m\Lambda }^{[{s}],\,a,\,\omega }\right)'' + \left(\omega ^2- V^{[s]}\right) u_{m\Lambda }^{[{s}],\,a,\,\omega } =H_{m\Lambda }^{[{s}],\,a,\,\omega },\nonumber \\ \qquad H_{ml}^{[{s}],\,a,\,\omega }:=\frac{\Delta ^{|s|/2+1}}{(r^2+a^2)^{|s|+3/2}} F_{m\Lambda }^{[{s}],\,a,\,\omega }, \end{aligned}$$

letting ′ denote $r^*$ derivatives. Here, $V^{[{s}]}=V^{[{s}]}_0+V_1^{[s]}+iV^{[{s}]}_2$, where

4.10 $$\begin{aligned} \begin{aligned} V^{[{s}]}_0&=\frac{4Mra m\omega -a^2m^2+\Delta \Lambda _{ml}^{[s]}}{(r^2+a^2)^2}, \\ V^{[{s}]}_1&=s^2\frac{(r-M)^2}{(r^2+a^2)^2}+\frac{\Delta }{(r^2+a^2)^4}\left(a^2\Delta +2Mr(r^2-a^2)\right) \geqq 0 , \\ V^{[{s}]}_2&=-\frac{2s\omega \left(r(r^2+a^2)+M(a^2-3r^2)\right)+2sam(r-M)}{(r^2+a^2)^2}. \end{aligned} \end{aligned}$$

We call (4.7) and (4.9) homogeneous radial ODEs if $F_{m\Lambda }^{[{s}],\,a,\,\omega }=H_{m\Lambda }^{[{s}],\,a,\,\omega }=0$.

To discuss boundary conditions for (4.7) and (4.9), it is convenient to introduce some notation:

##### Definition 4.4

Fix M>0, |a|≦M, $s\in \frac{1}{2}\mathbb {Z}$ and an admissible frequency triple $(\omega ,m,\Lambda )$ with respect to *s* such that $\omega \in \mathbb {R}\backslash \{0,m\upomega _+\}$.

1. Define $\upalpha ^{[s],\, a,\,\omega }_{ml,\,\mathcal {H}^+}$ and $\upalpha ^{[s],\, a,\,\omega }_{ml,\,\mathcal {H}^-}$ to be the unique classical solutions to the homogeneous radial ODE (4.7) with boundary conditions
  - if |a|<M,
    i. $\upalpha ^{[s],\, a,\,\omega }_{ml,\,\mathcal {H}^\pm }(r)(r-r_+)^{\mp \xi + s(1\pm 1)/2}$ are smooth at $r=r_+,$
    ii. $$\left((r-r_+)^{\mp \xi + s(1\pm 1)/2}\upalpha ^{[s],\, a,\,\omega }_{ml,\,\mathcal {H}^\pm }\right)\big |_{r=r_+}=1;$$
  - if |a|=M,
    i. $(r-M)^{\pm 2iM\omega +s(1\pm 1)}e^{\mp \beta (r-M)^{-1}}\upalpha ^{[s],\, a,\, \omega }_{ml,\,\mathcal {H}^\pm }(r)$ are smooth at r=M,
    ii. $$\left((r-M)^{\pm 2iM\omega +s(1\pm 1)}e^{\mp \beta (r-M)^{-1}}\upalpha ^{[s],\, a,\,\omega }_{ml,\,\mathcal {H}^\pm }\right)\big |_{r=M}=1.$$
2. Define $\upalpha ^{[s],\, a,\,\omega }_{ml,\,\mathcal {I}^+}$ and $\upalpha ^{[s],\, a,\,\omega }_{ml,\,\mathcal {I}^-}$ to be the unique classical solution to the homogeneous radial ODE (4.7) and boundary conditions
  - $$\upalpha ^{[s],\, a,\,\omega }_{ml,\,\mathcal {I}^\pm }\sim e^{\pm i\omega r}r^{\pm 2Mi\omega -s(1\pm 1)-1}$$ asymptotically as $$r\rightarrow \infty .$$^11^
  - $$\left(e^{\mp i\omega r}r^{\mp 2Mi\omega +s(1\pm 1)+1}\upalpha ^{[s],\, a,\,\omega }_{ml,\,\mathcal {I}^\pm }\right)\big |_{r=\infty }=1.$$

Finally, we define $u^{[s],\, a,\,\omega }_{ml,\,\mathcal {I}^\pm }$ and $u^{[s],\, a,\,\omega }_{ml,\,\mathcal {H}^\pm }$ by the following rescaling of the previously introduced functions:

$$\begin{aligned} u^{[s],\, a,\,\omega }_{ml,\,\mathcal {I}^\pm }=(r^2+a^2)^{1/2}\upalpha ^{[s],\, a,\,\omega }_{ml,\,\mathcal {I}^\pm }\,, \qquad u^{[s],\, a,\,\omega }_{ml,\,\mathcal {H}^\pm }=(r^2+a^2)^{1/2}\upalpha ^{[s],\, a,\,\omega }_{ml,\,\mathcal {H}^\pm }\,. \end{aligned}$$

By the standard theory of ODEs (we refer the reader to [134, Chapters 5 and 7] or [62, 99] for more detail), any solution to (4.7) has a representation in terms of those defined in Definition 4.4. In this paper, we are interested in solutions with particular boundary conditions.

##### Definition 4.5

Fix M>0, |a|≦M, $s\in \frac{1}{2}\mathbb {Z}$ and an admissible frequency triple $(\omega ,m,\Lambda )$ with respect to *a* and *s* such that $\omega \in \mathbb {R}\backslash \{0,m\upomega _+\}$. Assume $(r^2+a^2)^{1/2}F_{m\Lambda }^{[{s}],\,a,\,\omega }=O(1)$ as $r^*\rightarrow \pm \infty$. We say that $\upalpha _{m\Lambda }^{[s],\,a,\,\omega }$ is a solution to (4.7) with outgoing boundary conditions if there are $a^{[{s}]}_{\mathcal {H}^+},a^{[{s}]}_{\mathcal {I}^+}\in \mathbb {C}$ depending only on $(\omega ,m,\Lambda )$ such that

4.11 $$\begin{aligned} \begin{aligned} \Delta ^{\frac{|s|}{2}(1+{{\,\textrm{sign}\,}}s)}\upalpha _{m\Lambda }^{[{s}],\,a,\,\omega }{=}a^{[{s}]}_{\mathcal {H}^+}\cdot \Delta ^{\frac{|s|}{2}(1+{{\,\textrm{sign}\,}}s)}\upalpha ^{[s],\, a,\,\omega }_{m\Lambda ,\,\mathcal {H}^+}+ O(r-r_+)\,,\\ r^{1-|s|(1-{{\,\textrm{sign}\,}}s)}\upalpha _{m\Lambda }^{[{s}],\,a,\,\omega }= a^{[{s}]}_{\mathcal {I}^+}\cdot r^{1-|s|(1-{{\,\textrm{sign}\,}}s)}\upalpha ^{[s],\, a,\,\omega }_{m\Lambda ,\,\mathcal {I}^+} +O(r^{-1})\,, \end{aligned} \end{aligned}$$

respectively as $r\rightarrow r_+$ and $r\rightarrow \infty$. If we assume the stronger decay $(r^2+a^2)^{1/2}F_{m\Lambda }^{[{s}],\,a,\,\omega }=O(w^{2|s|})$ as $r^*\rightarrow \pm \infty$, then the second term in the above equalities is lower order.

Furthermore, the asymptotic expansions for the solutions in Definition 4.4 then yield the so-called radial Teukolsky–Starobinsky identities. These identities, originally due to [144, 159] for |s|=1,2 and later generalized to all spins in [106], relate solutions of the homogeneous radial ODE (4.7) with spin +s and spin -s.

##### Proposition 4.6

(Radial Teukolsky–Starobinsky identities) Fix $s\in \frac{1}{2}\mathbb {Z}\backslash \{0\}$, M>0 and |a|≦M. Let $(\omega ,m,\Lambda )$ be an admissible frequency triple with respect to *s* and *a* satisfying $\omega \in \mathbb {R}\backslash \{0,m\upomega _+\}$. Dropping most subscripts, assume assume $(r^2+a^2)^{1/2}F_{m\Lambda }^{[{\pm s}],\,a,\,\omega }=O(w^{2|s|})$ as $r^*\rightarrow \infty$ and $r^*\rightarrow -\infty$ and let $\upalpha _{m\Lambda }^{[{\pm s}],\,a,\,\omega }$ be outgoing solutions to the radial ODE (4.7) of spin ±s. Then,

4.12 $$\begin{aligned} \begin{aligned} \Delta ^s \left(-\frac{r^2+a^2}{\Delta }\underline{L}\right)^{2s}\left(\Delta ^s \upalpha _{m\Lambda }^{[{+ s}],\,a,\,\omega }\right)&= {\left\{ \begin{array}{ll} a^{[{+s}]}_{\mathcal {I}^{+}}\mathfrak {C}_s^{(1)}\upalpha ^{[-s],\, a,\,\omega }_{m\Lambda ,\,\mathcal {I}^+}+O(r^{-1})\\ a^{[{+s}]}_{\mathcal {H}^{+}}\mathfrak {C}_s^{(4)}\upalpha ^{[-s],\, a,\,\omega }_{m\Lambda ,\,\mathcal {H}^+}+O(r-r_+) \end{array}\right. }\,, \\ \left(\frac{r^2+a^2}{\Delta }L\right)^{2s}\upalpha _{m\Lambda }^{[{- s}],\,a,\,\omega }&= {\left\{ \begin{array}{ll} a^{[{-s}]}_{\mathcal {I}^{+}}\mathfrak {C}_s^{(3)}\upalpha ^{[+s],\, a,\,\omega }_{m\Lambda ,\,\mathcal {I}^+}+O(r^{-1})\\ a^{[{-s}]}_{\mathcal {H}^{+}}\mathfrak {C}_s^{(2)}\upalpha ^{[+s],\, a,\,\omega }_{m\Lambda ,\,\mathcal {H}^+}+O(r-r_+) \end{array}\right. }\,, \end{aligned} \end{aligned}$$

where $\mathfrak {C}_{s}^{(i)}$ are given by

$$\begin{aligned}  &   \mathfrak {C}_s^{(1)}=(2i\omega )^{2s},\\  &   \quad \mathfrak {C}_s^{(2)}={\left\{ \begin{array}{ll} (-2\beta )^{2s} & \text { if } |a|=M \\ \prod _{j=0}^{2s-1}\left(2\xi +s-j\right)& \text { if } |a|<M \end{array}\right. }, \qquad \mathfrak {C}_s^{(3)}=\frac{\mathfrak {C}_s}{\mathfrak {C}_s^{(1)}}, \quad \mathfrak {C}_s^{(4)}=\frac{\mathfrak {C}_s}{\mathfrak {C}_s^{(2)}}, \end{aligned}$$

and $\mathfrak {C}_s$ is the so-called radial Teukolsky–Starobinsky constant. The latter is explicitly computable in terms of *s*, *M*, *a* and $(\omega ,m,\Lambda )$. For instance,4.13

##### Proof

The result follows by explicit computations using asymptotic series expansions, see for instance [155, Proposition 2.14] for details. □

##### Remark 4.7

*(Teukolsky–Starobinsky constants)* We don’t expect that the requirement of outgoing boundary conditions is necessary to compute $\mathfrak C_s$. Dropping most subscripts, for $\upalpha ^{[{\pm s}]}$ solutions of the homogenenous radial ODE (4.7) of spin ±s, if we set

$$\begin{aligned} P^{[+s]}:=\Delta ^s \upalpha ^{[{+s}]}\,,\qquad P^{[-s]}:=\upalpha ^{[{-s}]}\,, \end{aligned}$$

then, at least for $s\in \{\frac{1}{2},1,\frac{3}{2},2\}$ one can check that $P^{[\pm s]}$ is an eigenfunction of the operator

$$\begin{aligned} \Delta ^s\left(\hat{\mathcal {D}}^{\mp }\right)^{2s}\left[\Delta ^s\left(\hat{\mathcal {D}}^{\pm }\right)^{2s}\right]\,, \qquad \hat{\mathcal {D}}^{+}:=-\frac{r^2+a^2}{\Delta }\underline{L}, \,\, \hat{\mathcal {D}}^{-}:=\frac{r^2+a^2}{\Delta }L\,, \end{aligned}$$

for the same eigenvalue, with eigenvalue $\mathfrak C_s=\mathfrak C_s(\omega ,m,\Lambda )\in \mathbb {R}$. A similar property holds for solutions to the angular ODE (4.2): at least for $s\in \{\frac{1}{2},1,\frac{3}{2},2\}$, solutions of the angular ODE (4.2) with spin ±s are eigenfunctions of the operator

$$\begin{aligned} \left(\sin \theta \right)^{2s}\left(\frac{\hat{\mathcal {L}}_{s}^\mp }{\sin \theta }\right)^{2s}\left(\sin \theta \right)^{2s}\left(\frac{\hat{\mathcal {L}}_{s}^\pm }{\sin \theta }\right)^{2s}\,, \qquad \hat{\mathcal {L}}^{\pm }= \frac{\textrm{d}}{\textrm{d}\theta }\pm \left(\frac{m}{\sin \theta }-a\omega \cos \theta \right)\,, \end{aligned}$$

for the same eigenvalue. The latter eigenvalue, $\mathfrak B_s=\mathfrak B_s(|s|,\omega ,m,l)$ is called the angular Teukolsky–Starobinsky constant and satisfies $(-1)^{2s}\mathfrak B_s\geqq 0$ (see [155, Section 2.3]).

Let us now take $\Lambda =\boldsymbol{\Lambda }_{ml}^{[s],a\omega }$ to compare the angular and radial Teukolsky–Starobinksy constants. Explicit computations (see [106], for instance, or [35, Sections 70 and 81] for $s=\pm 1, \pm 2$) show that

4.14 $$\begin{aligned} \mathfrak {C}_s= (-1)^{2s}\mathfrak {B}_s\geqq 0\,, \quad |s|\leqq 2\,,\quad \quad \mathfrak {C}_2= (-1)^{2s}\mathfrak {B}_s+144M^2\omega ^2\geqq 144M^2\omega ^2\geqq 0\,, \end{aligned}$$

although $\mathfrak {C}_s-(-1)^{2s}\mathfrak {B}_s\geqq 0$. For $s\in \{\frac{1}{2},1,\frac{3}{2}\}$, we have $\mathfrak {C}_s>0$ for each finite (ω,m,l) (see [155, Section 2.3]), but this constant can be arbitrarily small as $\omega \rightarrow 0$ for some admissible choices of (*m*, *l*) [43]. In contrast, for s=2, noting that $144M^2\omega ^2>0$ whenever $\omega \ne 0$ and that, otherwise,

$$\begin{aligned} \mathfrak {C}_s\geqq (-1)^{2\,s}\mathfrak {B}_s=\left[l(l+1)-s^2+|s|\right]^{2|s|}\geqq \left[2|s|\right]^{2|s|} \,, \quad 0<|s|\leqq 2\,, \quad a\omega =0\,, \end{aligned}$$

we deduce from (4.14) that $\mathfrak {C}_s\geqq b> 0$. Thus, one can show quantitatively that there are no real algebraically special frequencies for this spin. To our knowledge, this interesting feature of gravitational perturbations has not been observed before. Finally, we remark that, for |s|>2, the constant $\mathfrak {C}_s$ can take negative values for some choices of a≠0 and (ω,m,l).

#### The transformed radial ODEs and their boundary conditions

For some spin $s\in \mathbb {Z}$, a∈[-M,M], and $(\omega ,m,\Lambda )$ admissible, let $\alpha _{m\Lambda }^{[{s}],\,a,\,\omega }$ solve the Teukolsky radial ODE (4.7) with inhomogeneity $F_{m\Lambda }^{[{s}],\,a,\,\omega }$. Let $\uppsi _{({0}),\,m\Lambda }^{[{s}],\,a,\,\omega }$ and $\mathfrak G_{({0}),\,m\Lambda }^{[{s}],\,a,\,\omega }$ be rescalings of the Teukolsky variable and inhomogeneity:

4.15 $$\begin{aligned} \uppsi _{({0}),\,m\Lambda }^{[{s}],\,a,\,\omega }&:=(r^2+a^2)^{-|s|+1/2}\Delta ^{|s|/2(1+{{\,\textrm{sign}\,}}s)}\alpha _{m\Lambda }^{[{s}],\,a,\,\omega }=w^{-|s|/2}u_{m\Lambda }^{[{s}],\,a,\,\omega }\,, \end{aligned}$$

4.16 $$\begin{aligned} \mathfrak G_{({0}),\,m\Lambda }^{[{s}],\,a,\,\omega }&:= w(r^2+a^2)^{1/2}F_{m\Lambda }^{[{s}],\,a,\,\omega }=w^{-s/2}H_{m\Lambda }^{[{s}],\,a,\,\omega } \,. \end{aligned}$$

From these, for k=1,...,|s| we define

4.17 $$\begin{aligned} \uppsi _{({k}),\,m\Lambda }^{[{s}],\,a,\,\omega }&:=w^{-1}\mathcal {L}\uppsi _{({k-1}),\,m\Lambda }^{[{s}],\,a,\,\omega }\,, \end{aligned}$$

4.18 $$\begin{aligned} \mathfrak {G}_{({k}),\,m\Lambda }^{[{s}],\,a,\,\omega }&:=\mathcal {L}\Big (w^{-1}\mathfrak G_{({k-1}),\,m\Lambda }^{[{s}],\,a,\,\omega }\Big )\,, \end{aligned}$$

with $\mathcal {L}=L$ if s<0, $\mathcal {L}=\underline{L}$ if s>0 and $\mathcal {L}$ being the identity if s=0; here and throughout the section we are considering, by abuse of notation, *L* and $\underline{L}$ to be given by their separated picture analogues,

$$\begin{aligned} L=\frac{\textrm{d}}{\textrm{d}r^*}-i\omega +\frac{iam}{r^2+a^2}\,,\quad \underline{L}=-\frac{\textrm{d}}{\textrm{d}r^*}-i\omega +\frac{iam}{r^2+a^2}\,. \end{aligned}$$

Then, from the Teukolsky radial ODE (4.7), we derive the radial ODEs for $\uppsi _{({k}),\,m\Lambda }^{[{s}],\,a,\,\omega }$ when k=0,...,|s|:4.19 where $c_{s,\,k,\,i}^{\Phi },c_{s,\,k,\,i}^{\textrm{id}}=O(1)$ as $r^*\rightarrow \pm \infty$ are the functions introduced in Proposition 3.3 (recall $ac_{s,\,1,\,0}^{\textrm{id}}$ in (4.19) should be replaced by $c_{s,\,1,\,0}^{\textrm{id}}$ when |s|≠1), and where

4.20 $$\begin{aligned} \mathcal {V}_{({k}),\,m\Lambda }^{[{s}],\,a,\,\omega }&=\frac{\Delta \Lambda +4Mram\omega -a^2m^2}{(r^2+a^2)^2}+w\left[|s|+k(2|s|-k-1)\right]\nonumber \\&\quad +\frac{a^2\Delta w}{(r^2+a^2)^2} [1-2|s|-2k(2|s|-k-1)]\nonumber \\&\quad +\frac{2Mr(r^2-a^2)w}{(r^2+a^2)^2}[1-3|s|+2s^2-3k(2|s|-k-1)]\,. \end{aligned}$$

We let $\Psi _{m\Lambda }^{[{s}],\,a,\,\omega }:=\uppsi _{({|s|}),\,m\Lambda }^{[{s}],\,a,\,\omega }$ and $\mathfrak G_{m\Lambda }^{[{s}],\,a,\,\omega }:=\mathfrak G_{({|s|}),\,m\Lambda }^{[{s}],\,a,\,\omega }$; thus, the transformed radial ODE verified by the former is

4.21 $$\begin{aligned}&\left(\Psi _{m\Lambda }^{[{s}],\,a,\,\omega }\right)''+\left(\omega ^2-\mathcal {V}_{m\Lambda }^{[{s}],\,a,\,\omega }\right)\Psi _{m\Lambda }^{[{s}],\,a,\,\omega }\nonumber \\&\quad =\mathfrak {G}_{m\Lambda }^{[{s}],\,a,\,\omega }+aw\sum _{k=0}^{|s|-1}\left(im c_{s,\,k}^{\Phi }+ c_{s,\,k}^{\textrm{id}}\right)\uppsi _{({k}),\,m\Lambda }^{[{s}],\,a,\,\omega }\,, \end{aligned}$$

where $\mathcal {V}_{m\Lambda }^{[{s}],\,a,\,\omega }=\mathcal {V}^{[s]}_{0}+\mathcal {V}^{[{s}]}_{1}$ is real and given by

4.22 $$\begin{aligned} \begin{aligned} \mathcal {V}^{[{s}]}_{0}&:=\frac{4Mram\omega -a^2m^2+\Delta (\Lambda +s^2)}{(r^2+a^2)^2} \\&= \frac{\Delta (\Lambda +s^2-2am\omega )}{(r^2+a^2)^2}-\left(\omega -\frac{am}{r^2+a^2}\right)^2+\omega ^2, \\ \mathcal {V}^{[{s}]}_{1}&:=\frac{\Delta }{(r^2+a^2)^4}\left[(1-2s^2)a^2\Delta + 2(1-s^2)Mr(r^2-a^2)\right] . \end{aligned} \end{aligned}$$

Finally, note that using (4.17), for k<|s|, we can recast (4.19) as the transport equation

4.23 $$\begin{aligned}&\underline{\mathcal {L}}(w\uppsi _{({k+1}),\,m\Lambda }^{[{s}],\,a,\,\omega })-{{\,\textrm{sign}\,}}s (|s|-k)w'\uppsi _{({k+1}),\,m\Lambda }^{[{s}],\,a,\,\omega }\nonumber \\&\quad =-w\left[\Lambda -2am\omega +|s|+k(2|s|-k-1)\right]\uppsi _{({k}),\,m\Lambda }^{[{s}],\,a,\,\omega }\nonumber \\&\qquad -\frac{2Mr(r^2-a^2)\Delta }{(r^2+a^2)^4}\left[1-3|s|+2s^2-3k(2|s|-k-1)\right]\uppsi _{({k}),\,m\Lambda }^{[{s}],\,a,\,\omega }\nonumber \\&\qquad +\frac{2iamrw}{r^2+a^2}{{\,\textrm{sign}\,}}{s}\left(2|s|-2k-1\right)\uppsi ^{[{s}]}_{(k)}\nonumber \\&\qquad - \frac{a^2\Delta ^2}{(r^2+a^2)^4}\left[1-2|s|-2k(2|s|-k-1)\right]\uppsi _{({k}),\,m\Lambda }^{[{s}],\,a,\,\omega } \nonumber \\&\qquad +\sum _{j=0}^{k-1}w\left(ac_{s,\,k,\,j}^{\Phi }im+ ac_{s,\,k,\,j}^{\textrm{id}}\right)\uppsi _{({j}),\,m\Lambda }^{[{s}],\,a,\,\omega }+\mathfrak G_{({k}),\,m\Lambda }^{[{s}],\,a,\,\omega }\,. \end{aligned}$$

By the above construction, it is clear that $\uppsi _{({k}),\,m\Lambda }^{[{s}],\,a,\,\omega }$ inherit the boundary conditions of $\upalpha _{m\Lambda }^{[{s}],\,a,\,\omega }$. To make this precise, it is again convenient to fix some notation:

##### Definition 4.8

Fix M>0, |a|≦M, $s\in \frac{1}{2}\mathbb {Z}$ and an admissible frequency triple $(\omega ,m,\Lambda )$ with respect to *s* such that $\omega \in \mathbb {R}\backslash \{0,m\upomega _+\}$.

1. Define $\uppsi ^{[s],\, a,\,\omega }_{(k),\,m\Lambda ,\,\mathcal {H}^+}$ and $\uppsi ^{[s],\, a,\,\omega }_{(k),\,m\Lambda ,\,\mathcal {H}^-}$ as the unique solutions to the homogeneous radial ODE (4.19) with boundary conditions
  - if |a|<M,
    i. $\uppsi ^{[s],\, a,\,\omega }_{(k),\,m\Lambda ,\,\mathcal {H}^\pm }(r)(r-r_+)^{\mp \xi - \frac{1}{2}(|s|-k)(1\mp {{\,\textrm{sign}\,}}s)}$ are smooth at $r=r_+,$ and
    ii. $$\left|\left(w^{-\frac{|s|-k}{2}}\Delta ^{\pm \frac{|s|-k}{2}{{\,\textrm{sign}\,}}s}(r-r_+)^{\mp \xi }\uppsi ^{[s],\, a,\,\omega }_{(k),\,ml,\,\mathcal {H}^\pm }\right)\Big |_{r=r_+}\right|^2=1;$$
  - if |a|=M,
    i. $(r-M)^{\pm 2iM\omega -(|s|-k)(1\mp {{\,\textrm{sign}\,}}s)}\uppsi ^{[s],\, a,\,\omega }_{(k),\, ml,\,\mathcal {H}^\pm }(r)e^{\mp \beta (r-M)^{-1}}$ are smooth at r=M, and
    ii. $$\left|\left(w^{-\frac{|s|-k}{2}}(r-M)^{\pm 2iM\omega \pm (|s|-k){{\,\textrm{sign}\,}}s}e^{\mp \beta (r-M)^{-1}}\uppsi ^{[s],\, a,\,\omega }_{(k),\,ml,\,\mathcal {H}^\pm }\right)\Big |_{r=M}\right|^2=1.$$
2. Define $\uppsi ^{[s],\, a,\,\omega }_{(k),\,ml,\,\mathcal {I}^+}$ and $\uppsi ^{[s],\, a,\,\omega }_{(k),\,ml,\,\mathcal {I}^-}$ as the unique classical solutions to the homogeneous radial ODE (4.19) with boundary conditions
  - $\uppsi ^{[s],\, a,\,\omega }_{(k),\,ml,\,\mathcal {I}^\pm }\sim e^{\pm i\omega r}r^{\pm 2Mi\omega -(|s|-k)(1\pm {{\,\textrm{sign}\,}}s)}$ asymptotically as $r\rightarrow \infty ,$ and
  - $$\left|\left(e^{\mp i\omega r}r^{\mp 2iM\omega }w^{-\frac{|s|-k}{2}}\Delta ^{\pm \frac{|s|-k}{2}{{\,\textrm{sign}\,}}s}\uppsi ^{[s],\, a,\,\omega }_{(k),\,ml,\,\mathcal {I}^\pm }\right)\big |_{r=\infty }\right|^2=1.$$

##### Definition 4.9

*(Outgoing boundary conditions)*

Fix M>0, |a|≦M, $s\in \mathbb {Z}$, $k\in \{0,\dots ,|s|\}$ and an admissible frequency triple $(\omega ,m,\Lambda )$ with respect to *a* and *s* such that $\omega \in \mathbb {R}\backslash \{0,m\upomega _+\}$. Assume $\mathfrak G_{({k}),\,m\Lambda }^{[{s}],\,a,\,\omega }=O(w)$ as $r^*\rightarrow \pm \infty$ and that, for every j<k and for j=k+1, $\uppsi _{({j}),\,m\Lambda }^{[{s}],\,a,\,\omega }$ are bounded as $r^*\rightarrow \pm \infty$. We say that $\uppsi _{({k}),\,m\Lambda }^{[{s}],\,a,\,\omega }$ is a solution to (4.19) with outgoing boundary conditions if there are $A^{[{s}]}_{k,\mathcal {H}^+},A^{[{s}]}_{k,\mathcal {I}^+}\in \mathbb {C}$ depending only on $(\omega ,m,\Lambda )$ such that

4.24 $$\begin{aligned}  &   \uppsi _{({k}),\,m\Lambda }^{[{s}],\,a,\,\omega }= A^{[{s}]}_{k,\mathcal {H}^+}\cdot \uppsi ^{[s],\, a,\,\omega }_{(k), m\Lambda ,\,\mathcal {H}^+}+ O(r-r_+),\nonumber \\  &   \uppsi _{({k}),\,m\Lambda }^{[{s}],\,a,\,\omega }= A^{[{s}]}_{k,\mathcal {I}^+}\cdot \uppsi ^{[s],\, a,\,\omega }_{(k), m\Lambda ,\,\mathcal {I}^+} +O(r^{-1}), \end{aligned}$$

respectively as $r\rightarrow r_+$ and $r\rightarrow \infty$.

Furthermore, the asymptotic expansions for the solutions in Definition 4.4, acted on by suitably weighted $\mathcal {L}$ derivatives, provide us a way of relating the coefficients of the expansions (4.11) and (4.24):

##### Lemma 4.10

Fix M>0, |a|≦M, $s\in \mathbb {Z}_{>0}$, and an admissible frequency triple $(\omega ,m,\Lambda )$ with respect to *a* and *s* such that $\omega \in \mathbb {R}\backslash \{0,m\upomega _+\}$. Assume that, for all k=0,⋯,|s|, $\mathfrak G_{({k}),\,m\Lambda }^{[{s}],\,a,\,\omega }=O(w)$ as $r^*\rightarrow \pm \infty$ and $\uppsi _{({k}),\,m\Lambda }^{[{s}],\,a,\,\omega }$ are solutions to (4.19) arising from an outgoing solution $\upalpha _{m\Lambda }^{[{s}],\,a,\,\omega }$ to (4.7) via (4.17). Then, $\uppsi _{({k}),\,m\Lambda }^{[{s}],\,a,\,\omega }$ have outgoing boundary conditions and, moreover, we can relate the coefficients of (4.11) and (4.24) by

$$\begin{aligned} \left|A^{[{+ s}]}_{k,\,\mathcal {I}^+}\right|^2&=(2\omega )^{2k}\left|a^{[{+ s}]}_{\mathcal {I}^+}\right|^2\,, \\ \left|A^{[{ -s}]}_{k,\,\mathcal {H}^+}\right|^2&=2Mr_+\sum _{j=0}^{k-1}\left([4Mr_+(\omega -m\upomega _+)]^2+(s-j)^2(r_+-r_-)^2\right)\\&\qquad \times \left\rbrace \begin{array}{lr} 1, & |a|=M\\ (r_+-r_-)^{-2k}, & |a|<M \end{array}\right\lbrace \left|a^{[{- s}]}_{\mathcal {H}^+}\right|^2\,, \end{aligned}$$

and, if we assume additionally the stronger decay $\mathfrak G_{({k}),\,m\Lambda }^{[{s}],\,a,\,\omega }=O(w^{1+(|s|-k)})$ as $r^*\rightarrow (-{{\,\textrm{sign}\,}}s) \infty$,

$$\begin{aligned} \left|A^{[{- s}]}_{k,\,\mathcal {I}^+}\right|^2&=\frac{\mathfrak D_{s,k}^{\mathcal {I}}}{(2\omega )^{2k}}\left|a^{[{- s}]}_{\mathcal {I}^+}\right|^2\,, \\ \left|A^{[{ +s}]}_{k,\,\mathcal {H}^+}\right|^2&=\frac{(2Mr_+)^{1-2(|s|-k)}\mathfrak D_{s,k}^{\mathcal {H}}}{\sum _{j=1}^{k}\left([4Mr_+(\omega -m\upomega _+)]^2+(s-j)^2(r_+-r_-)^2\right)}\\&\quad \times \left\rbrace \begin{array}{lr} 1, & |a|=M\\ (r_+-r_-)^{2|s|}, & |a|<M \end{array}\right\lbrace \left|a^{[{+ s}]}_{\mathcal {H}^+}\right|^2\,, \end{aligned}$$

where $\mathfrak D_{s,k}^{\mathcal {I}}$ and $\mathfrak D_{s,k}^{\mathcal {H}}$ are constants which are explicitly computable in terms of *s*, *M*, *a* and $(\omega ,m,\Lambda )$. For instance,4.254.26

##### Proof

The result follows by explicit computations, and we refer the reader to the thesis [156, Chapter I.3.2.4] for further details. □

## A Toolbox for Frequency Space Estimates

### The separated current templates

In this section, we introduce the current templates which will be used, in Section 6 to obtain estimates on solutions of the radial ODEs in Sections 4.3. For the latter, we rewrite the ODEs in the general form

5.1 $$\begin{aligned} U''+W U'+(\omega ^2-V)U=H\,, \end{aligned}$$

where ω is real and *V*, *W*, *H* are known functions of $r^*$ with *W* being real-valued.

#### The virial current templates

Let $U(r^*)$ be sufficiently regular. For $h=h(r^*)$ a $C^1$ and piecewise $C^2$, $y=y(r^*)$ a $C^0$ and piecewise $C^1$ and $f=f(r^*)$ a $C^2$ and piecewise $C^3$ functions, we define the frequency-localized virial currents

5.2 $$\begin{aligned} \begin{aligned} Q^h[U]&=h\operatorname {Re}(U\overline{U}')-\frac{1}{2} h'|U|^2+\frac{1}{2}hW|U|^2, \\ Q^y[U]&=y|U'|^2+y(\omega ^2-\operatorname {Re}V)|U|^2, \\ Q^f[U]&=f|U'|^2+\left(f(\omega ^2-\operatorname {Re}V) -\frac{1}{2} f'' +\frac{1}{2} f'W\right)|U|^2+f'\operatorname {Re}(U\overline{U}'), \end{aligned} \end{aligned}$$

which, if *U* satisfies the model ODE (5.1), have derivatives

5.3 $$\begin{aligned} \begin{aligned} (Q^h[U])'&=h|U'|^2+h\left(\operatorname {Re}V+\frac{1}{2} W'-\omega ^2\right)|U|^2\\&\quad -\frac{1}{2} \left(h''-W h'\right)|U|^2+h\operatorname {Re}(H\overline{U}), \\ \left(Q^y[U]\right)'&=(y'-2yW)|U'|^2+y'\omega ^2|U|^2-(y\operatorname {Re}V)'|U|^2\\&\quad -2y(\operatorname {Im}V)\operatorname {Im}(U\overline{U}')+2y\operatorname {Re}(H\overline{U}'), \\ (Q^f[U])'&=2(f'+fW)|U'|^2+\left(-f\operatorname {Re}V'-\frac{1}{2} f'''+\frac{1}{2} Wf''\right)|U|^2\\&\quad -2f(\operatorname {Im}V) \operatorname {Im}(U\overline{U}')\\&\quad +f'\operatorname {Re}(H\overline{U})+2f\operatorname {Re}(H\overline{U}'). \end{aligned} \end{aligned}$$

These identities are obtained multiplying (5.1) by $h\overline{U}$, $2y\overline{U}'$ and $f'\overline{U}+2f\overline{U}'$, respectively, and taking the real part. For our transformed system, the multiplier identities for *h* and *y* can be rewritten as follows:

##### Lemma 5.1

Fix $s\in \mathbb {Z}$ and $0\leqq k\leqq |s|$. Define $\uppsi _{(k)}$ by (4.17) and recall the definitions of virial current templates in (5.2). Replacing *U* by $\uppsi _{(k)}$, we have from (5.3)

5.4 $$\begin{aligned} (Q^h)'[\uppsi _{(k)}]&=h|\uppsi _{(k)}'|^2+h\left[\mathcal {V}_{(k)}-\frac{1}{2}(|s|-k)\left(\frac{w'}{w}\right)'-\omega ^2\right]|\uppsi _{(k)}|^2\nonumber \\&\quad +h\operatorname {Re}\left(\mathfrak {G}_{(k)}\overline{\uppsi _{(k)}}\right)\nonumber \\&\quad -\frac{1}{2} \left(h''+(|s|-k)\frac{w'}{w} h'\right)|\uppsi _{(k)}|^2+ wh\sum _{j=0}^{k-1}\operatorname {Re}\left[\uppsi _{(j)}\left(ac_{s,k,j}^\textrm{id}\right.\right.\nonumber \\&\quad \left.\left.+imac_{s,k,j}^{\Phi }\right)\overline{\uppsi _{(k)}}\right]\,, \end{aligned}$$

5.5 $$\begin{aligned} \left(Q^y\right)'[\uppsi _{(k)}]&=y'|\uppsi _{(k)}'|^2+y'\omega ^2|\uppsi _{(k)}|^2-(y{\mathcal {V}}_{(k)})'|\uppsi _{(k)}|^2 +2y\operatorname {Re}\left(\mathfrak {G}_{(k)}\overline{\uppsi _{(k)}}'\right)\nonumber \\&\qquad +2y(|s|-k)w'\left\rbrace w|\uppsi _{(k+1)}|^2-\left(\omega -\frac{am}{r^2+a^2}\right)\operatorname {Re}\left[\uppsi _{(k+1)}\overline{\uppsi _{(k)}}\right]\right\lbrace \nonumber \\&\qquad + 2y(|s|-k)\frac{4amrw}{(r^2+a^2)}\left\rbrace w\operatorname {Im}\left[\uppsi _{(k+1)}\overline{\uppsi }_{(k)}\right]+ \left(\omega -\frac{am}{r^2+a^2}\right)|\uppsi _{(k)}|^2\right\lbrace \nonumber \\&\qquad + 2wy\sum _{j=0}^{k-1}\operatorname {Re}\left[\uppsi _{(j)}\left(ac_{s,k,j}^\textrm{id}+imac_{s,k,j}^{\Phi }\right)\overline{\uppsi _{(k)}'}\right]\,. \end{aligned}$$

We can also rewrite the last identity by noting that

$$\begin{aligned}&wy\operatorname {Re}\left[\uppsi _{(j)}\left(ac_{s,k,j}^\textrm{id}+imac_{s,k,j}^{\Phi }\right)\overline{\uppsi _{(k)}'}\right]\\&\quad =\Big (wy\operatorname {Re}\left[\uppsi _{(j)}\left(ac_{s,k,j}^\textrm{id}+imac_{s,k,j}^{\Phi }\right)\overline{\uppsi _{(k)}'}\right]\Big )' \nonumber \\&\qquad - wy_{(k)}\left(\omega -\frac{am}{r^2+a^2}\right)\operatorname {Im}[(ac_{s,k,j}^\textrm{id}+imac_{s,k,j}^\Phi )\uppsi _{(j)}\overline{\uppsi _{(k)}}]\\&\qquad +w^2y_{(k)}\operatorname {Re}[(ac_{s,k,j}^\textrm{id}+imac_{s,k,j}^\Phi )\uppsi _{(j+1)}\overline{\uppsi _{(k)}}]\\&\qquad -\operatorname {Re}[(wy_{(k)} (ac_{s,k,j}^\textrm{id}+imac_{s,k,j}^\Phi ))'\uppsi _{(j)}\overline{\uppsi _{(k)}}]\,. \end{aligned}$$

##### Proof

Note that, for $U=\uppsi _{(k)}$, we have

$$\begin{aligned} W=-(|s|-k)\frac{w'}{w}\,. \end{aligned}$$

For the *h* current, the result follows directly from (5.3). For the *y* current, given (4.17), we derive

$$\begin{aligned}&|\uppsi _{(k)}'|^2 =w\operatorname {Re}\left[\uppsi _{(k+1)}\left(-{{\,\textrm{sign}\,}}s\overline{\uppsi _{(k)}}'\right)\right]+{{\,\textrm{sign}\,}}s \left(\omega -\frac{am}{r^2+a^2}\right)\operatorname {Im}\left[\uppsi _{(k)}\overline{\uppsi _{(k)}}'\right]\\&\qquad \quad \,\,\,=w^2|\uppsi _{(k+1)}|^2-w\left(\omega -\frac{am}{r^2+a^2}\right)\operatorname {Im}\left[\uppsi _{(k+1)}\overline{\uppsi _{(k)}}\right]\\&\qquad \qquad \quad +{{\,\textrm{sign}\,}}s \left(\omega -\frac{am}{r^2+a^2}\right)\operatorname {Im}\left[\uppsi _{(k)}\overline{\uppsi _{(k)}}'\right]\,, \operatorname {Im}\left[\uppsi _{(k)}\overline{\uppsi _{(k)}}'\right]\\&\qquad \quad \,\,\,= {{\,\textrm{sign}\,}}s\left[w\operatorname {Im}\left[\uppsi _{(k+1)}\overline{\uppsi }_{(k)}\right]+\left(\omega -\frac{am}{r^2+a^2}\right)|\uppsi _{(k)}|^2\right]\,. \end{aligned}$$

Hence, adding $-W|U^{\prime }|$ and $$-\operatorname {Im}V\operatorname {Im}(U\overline{U}')$$ and using (4.20), we can conclude the proof of (5.4). For the alternative version of the j<k coupling errors, we integrate by parts in $$r^*$$ and use (4.17) again. □

##### Remark 5.2

*(Use of virial currents)*

As Lemma 5.1 and identities (5.3) show, applying virial currents to (4.19) will allow one to exploit the sign (and size) of $\mathcal {V}'_{(k)}$ and $\omega ^2-\mathcal {V}_{(k)}$ to produce $L^2$-in-*r* estimates for $\uppsi _{(k)}$, at the cost of producing (in the case of *y* and *f* currents) boundary term errors.

#### The Killing energy currents

Let $U(r^*)$ be a sufficiently regular function. We define the frequency-localized energy currents

5.6 $$\begin{aligned} Q^T[U]&=-\omega \operatorname {Im}(U'\overline{U})\,, \end{aligned}$$

5.7 $$\begin{aligned} Q^K[U]&=-(\omega -m\upomega _+)\operatorname {Im}(U'\overline{U})\,, \end{aligned}$$

which, if *U* satisfies the ODE (5.1), have derivatives

5.8 $$\begin{aligned} \begin{aligned} (Q^T)'[U] =&-\omega \operatorname {Im}(H\overline{U})+\omega \operatorname {Im}V |U|^2-\omega W\operatorname {Im}(\overline{U}U')\,, \\ (Q^K)'[U] =&-(\omega -m\upomega _+) \operatorname {Im}(H\overline{U})+(\omega -m\upomega _+) \operatorname {Im}V |U|^2\\&-(\omega -m\upomega _+) W\operatorname {Im}(\overline{U}U')\,. \end{aligned} \end{aligned}$$

These identities are obtained multiplying (5.1) by $i\omega \overline{U}$ and $i(\omega -m\upomega _+)\overline{U}$, respectively, which are the frequency space analogues of $T\overline{U}$ and $K\overline{U}$ (see definitions in Section 2.2), and taking the real part.

For our system of transformed equations, the above identities can be rewritten:

##### Lemma 5.3

Fix $s\in \mathbb {Z}$ and $0\leqq k\leqq |s|$. Define $\uppsi _{(k)}$ by (4.17) and recall the definitions of virial current templates in (5.2). Replacing *U* by $\uppsi _{(k)}$, we have from (5.8)

$$\begin{aligned} (Q^T)'[\uppsi _{(k)}] =&-\omega \operatorname {Im}[\mathfrak {G}_{(k)}\overline{\uppsi _{(k)}}]-\omega \sum _{j=0}^{k-1}\operatorname {Im}[(ac_{s,k,j}^\textrm{id}+imc_{s,k,j}^\Phi )\uppsi _{(j)}\overline{\uppsi _{(k)}}]\\&-{{\,\textrm{sign}\,}}s (|s|-k)\omega \left[w'\operatorname {Im}[\uppsi _{(k+1)}\overline{\uppsi _{(k)}}]+\frac{4amrw}{r^2+a^2}|\uppsi _{(k)}|^2\right]\,, \\ (Q^K)'[\uppsi _{(k)}] =&-(\omega -m\upomega _+) \operatorname {Im}[\mathfrak {G}_{(k)}\overline{\uppsi _{(k)}}]\\&-(\omega -m\upomega _+)\sum _{j=0}^{k-1}\operatorname {Im}[(ac_{s,k,j}^\textrm{id}+imc_{s,k,j}^\Phi )\uppsi _{(j)}\overline{\uppsi _{(k)}}]\\&-{{\,\textrm{sign}\,}}s (|s|-k)(\omega -m\upomega _+) \left[w'\operatorname {Im}[\uppsi _{(k+1)}\overline{\uppsi _{(k)}}]+\frac{4amrw}{r^2+a^2}|\uppsi _{(k)}|^2\right]\,. \end{aligned}$$

#### The Teukolsky–Starobinsky energy current

In this section, we will introduce another type of energy currents adapted to the radial ODEs of Section 4.3. For simplicity we drop all sub and superscripts other than *s* in what follows. Our definitions are motivated by two observations. First, for any two solutions of the homogeneous radial ODE (4.9) of spin ±s and $\omega \in \mathbb {R}$, $u^{[{+s}]}$ and $u^{[{-s}]}$, respectively, the Wronskian

5.9 $$\begin{aligned} W\left(u^{[{+s}]},\overline{u^{[{-s}]}}\right)=\left(u^{[{+s}]}\right)'\cdot \overline{u^{[{-s}]}}-u^{[{+s}]}\cdot \left(\overline{u^{[{-s}]}}\right)' \end{aligned}$$

is conserved in $r^*$, since $V^{[{s}]}=\overline{V^{[{+s}]}}$. Second, the radial Teukolsky–Starobinsky identities of Proposition 4.6 provide a way of generating solutions of spin +s from solutions of spin -s and vice-versa.

We write our conservation law in terms of $\uppsi _{(0)}$. With $\mathcal {D}^+=-\frac{r^2+a^2}{\Delta }\underline{L}$ and $\mathcal {D}^-=\frac{r^2+a^2}{\Delta }L$, let us set

5.10 $$\begin{aligned} \begin{aligned} q^W\left[\uppsi ^{[{\pm s}]}_{(0)}\right]&:=\left(w^{-s/2}\uppsi ^{[{\pm s}]}_{(0)}\right)'\cdot \overline{(r^2+a^2)^{s+1/2}(\mathcal {D}^\pm )^{2s}\left((r^2+a^2)^{s-1/2}\uppsi ^{[{\pm s}]}_{(0)}\right)}\\&\qquad \left.-w^{-s/2}\uppsi ^{[{\pm s}]}_{(0)}\cdot \left[w^{s}(r^2+a^2)^{s+1/2}\overline{(\mathcal {D}^\pm )^{2s}\left((r^2+a^2)^{s-1/2}\uppsi ^{[{\pm s}]}_{(0)}\right)}\right]'\right\lbrace \,, \end{aligned} \end{aligned}$$

so that we have

$$\begin{aligned} \left(q^W\left[\uppsi ^{[{\pm s}]}_{(0)}\right]\right)'&=\frac{\mathfrak {G}^{[{\pm s}]}_{(0)}}{w}w(r^2+a^2)^{s+1/2}\overline{(\mathcal {D}^\pm )^{2s}\left[(r^2+a^2)^{s-1/2}\uppsi ^{[{\pm s}]}_{(0)}\right]}\\&\qquad - \uppsi ^{[{\pm s}]}_{(0)}w(r^2+a^2)^{s+1/2}\overline{(\mathcal {D}^\pm )^{2s}\left[(r^2+a^2)^{s-1/2}\frac{\mathfrak {G}^{[{\pm s}]}_{(0)}}{w}\right]} \,. \end{aligned}$$

Define the *T*-type and *K*-type Teukolsky–Starobinsky energy currents, respectively, by

$$\begin{aligned} Q^{W,\,T}_\pm \left[\uppsi ^{[{\pm s}]}_{(0)}\right]&:=\mp \frac{1}{2} \omega \operatorname {Im}\left(i^{2s}q^W_\pm \left[\uppsi ^{[{\pm s}]}_{(0)}\right]\right)\,,\\ Q^{W,\,K}_\pm \left[\uppsi ^{[{\pm s}]}_{(0)}\right]&:=\mp \frac{1}{2}(\omega -m\upomega _+)\operatorname {Im}\left( i^{2s}q^W_\pm \left[\uppsi ^{[{\pm s}]}_{(0)}\right]\right)\,. \end{aligned}$$

##### Lemma 5.4

(Teukolsky–Starobinsky energy currents)

Fix M>0, |a|≦M and $s\in \mathbb {Z}$. Let $(\omega ,m,\Lambda )$ be an admissible frequency triple with respect to *s* and *a* with $\omega \in \mathbb {R}\backslash \{0,m\upomega _+\}$. Assume that, for all k=0,⋯,|s|, $\mathfrak {G}^{[{ s}]}_{(k)}=O(w^{1+2(|s|-k)})$ as $r^*\rightarrow \infty$ and $\uppsi ^{[{s}]}_{(k)}$ have outgoing boundary conditions. Then, we have

$$\begin{aligned} Q^{W,\,T}_{{{\,\textrm{sign}\,}}s} (+\infty )&=- \omega ^2\left\rbrace \begin{array}{lr} 1, & s<0\\ \frac{\mathfrak {C}_s}{\mathfrak {D}_{s}^{\mathcal {I}}}, & s>0 \end{array}\right\lbrace \left|A^{[{s}]}_{\mathcal {I}^+}\right|^2=\frac{\omega }{\omega -\upomega _+}Q^{W,\,K}_{{{\,\textrm{sign}\,}}s} (+\infty )\,,\\ Q^{W,\,K}_{{{\,\textrm{sign}\,}}s} (-\infty )&=- (\omega -m\upomega _+)^2\left\rbrace \begin{array}{lr} \frac{\mathfrak {C}_s}{\mathfrak {D}_{s}^{\mathcal {H}}}, & s<0\\ 1, & s>0 \end{array}\right\lbrace \left|A^{[{s}]}_{\mathcal {I}^+}\right|^2= \frac{\omega -\upomega _+}{\omega }Q^{W,\,T}_{{{\,\textrm{sign}\,}}s} (-\infty )\,, \end{aligned}$$

Moreover, for a smooth χ which is compactly supported in $r\in [r_+,\infty )$, we have

$$\begin{aligned}&\int _{-\infty }^\infty \chi (Q^{W,K}_{{{\,\textrm{sign}\,}}s})'\textrm{d}r^*\\&\quad ={{\,\textrm{sign}\,}}s \frac{1}{2}(-1)^{|s|}\int _{-\infty }^\infty (\omega -m\upomega _+)\chi \operatorname {Im}\left[\Big (\sum _{k=0}^{|s|}p_{s,|s|,k}(r){\mathfrak {G}}_{(k)}\Big )\Big (\sum _{k=0}^{|s|}p_{s,|s|,k}(r)\overline{\uppsi _{(k)}}\Big )\right]\textrm{d}r^*\\&\quad \qquad - \frac{1}{2}(-1)^{|s|}\sum _{j=1}^{|s|}\int _{-\infty }^\infty \chi '\Big \{ \Big ((r^2+a^2)^{|s|-j+1/2}\sum _{k=0}^{j-1}p_{s,j-1,k}(r)\frac{{\mathfrak {G}}_{(k)}}{w}\Big )\\&\quad \qquad \qquad \qquad \qquad \qquad \times \overline{\left(\frac{\mathcal {L}}{w(r^2+a^2)}\right)^{|s|-j}\Big ((r^2+a^2)^{-1/2}\sum _{k=0}^{|s|}p_{s,|s|,k}(r)\uppsi _{(k)}\Big )}\Big \}\textrm{d}r^*\,, \end{aligned}$$

where $p_{s,k,j}(r)$ are smooth polynomials of degree k-j which are regular at $r=r_+$ and can be made explicit.

##### Proof

Notice that, by Definition 4.5,

$$\begin{aligned} W\left(u^{[{+s}]},\overline{u^{[{-s}]}}\right)&= a^{[{+s}]}_{\mathcal {H}^+}\overline{a^{[{-s}]}_{\mathcal {H}^+}}\cdot W\left((r^2+a^2)^{1/2}\Delta ^{-s/2}\Delta ^s\upalpha ^{[{+s}]}_{\mathcal {H}^+},(r^2+a^2)^{1/2}\Delta ^{-s/2}\overline{\upalpha ^{[{-s}]}_{\mathcal {H}^+}}\right)\\&\quad +O\left(r-r_+\right)\\&= a^{[{+s}]}_{\mathcal {H}^+}\overline{a^{[{-s}]}_{\mathcal {H}^+}}\left[\Delta \frac{\textrm{d}}{\textrm{d}r}(\Delta ^s\upalpha ^{[{+s}]}_{\mathcal {H}^+})\Delta ^{-s}\overline{\upalpha ^{[{-s}]}_{\mathcal {H}^+}}-\Delta \frac{\textrm{d}}{\textrm{d}r}\left(\overline{\upalpha ^{[{-s}]}_{\mathcal {H}^+}}\right)\upalpha ^{[{+s}]}_{\mathcal {H}^+}\right]\\&\quad +O\left(r-r_+\right)\\&=\left\rbrace \begin{array}{lr} -(-2\xi +s)(r_+-r_-) & \text { if } |a|<M\\ -2\beta & \text { if } |a|=M \end{array}\right\lbrace \left(a^{[{+s}]}_{\mathcal {H}^+}\overline{a^{[{-s}]}_{\mathcal {H}^+}}-a^{[{+s}]}_{\mathcal {H}^-}\overline{a^{[{-s}]}_{\mathcal {H}^-}}\right)\\&\quad +O(r-r_+)\,, \end{aligned}$$

and, as $$r\rightarrow \infty $$,

$$\begin{aligned} W\left(u^{[{+s}]},\overline{u^{[{-s}]}}\right)&=2i\omega a^{[{+s}]}_{\mathcal {I}^+}\overline{a^{[{-s}]}_{\mathcal {I}^+}}+O(r^{-1})\,. \end{aligned}$$

To transform the expressions above into expression in terms of one sign of spin only, we apply the correspondence, given by the radial Teukolsky–Starobinsky identities (4.12) in Proposition 4.6, between $a^{[{+s}]}_{\mathcal {H}^\pm }$ and $\upalpha ^{[{+s }]}_{\mathcal {I}^\pm }$ and their spin -s counterparts. For instance, if we are looking to obtain the expression of the boundary term at r=∞ in terms of spin -s, we assume $\upalpha ^{[{+s}]}$ is obtained from $\upalpha ^{[{-s}]}$ by (4.12) (see also [155, Proposition 2.21]). Thus, as $r\rightarrow \infty$,

$$\begin{aligned} W(+\infty )&= 2i\omega a^{[{+s}]}_{\mathcal {I}^+}\overline{a^{[{-s}]}_{\mathcal {I}^+}} = 2i\omega \frac{\mathfrak {C}_s}{(2i\omega )^{2s}}\left|a^{[{- s}]}_{\mathcal {I}^+ }\right|^2 = \pm 2i\omega \frac{\mathfrak {C}_s}{\mathfrak {D}_s^{\mathcal {I}}}\left|A^{[{- s}]}_{\mathcal {I}^+ }\right|^2\,, \end{aligned}$$

where we have concluded using Lemma 4.10. The remaining boundary terms are similar.

Finally, we turn to the errors due to the inhomogeneity. We first note that

$$\begin{aligned} \left(\frac{\mathcal {L}}{w(r^2+a^2)}\right)^{|s|}\left((r^2+a^2)^{|s|-1/2}\uppsi _{(0)}\right)&=(r^2+a^2)^{-1/2}\sum _{k=0}^{|s|}p_{s,|s|,k}(r){\uppsi }_{(k)}\,,\\ \left(\frac{\mathcal {L}}{w(r^2+a^2)}\right)^{|s|}\Big ((r^2+a^2)^{|s|-1/2}\frac{\mathfrak {G}_{(0)}}{w}\Big )&=\frac{(r^2+a^2)^{-1/2}}{w}\sum _{k=0}^{|s|}p_{s,|s|,k}(r)\mathfrak {G}_{(k)}\,. \end{aligned}$$

where $p_{s,k,j}(r)$ are polynomials of degree k-j in *r* which can be explicitly computed: for instance,

$$\begin{aligned} p_{s,|s|,|s|}=1,\quad p_{s,1,0}= &   -(2|s|-1)r{{\,\textrm{sign}\,}}s,\quad p_{s,2,1}=- 4(|s|-1)r{{\,\textrm{sign}\,}}s,\quad p_{s,2,0}\\= &   (2|s|-1)(a^2+2|s|r^2). \end{aligned}$$

By integrating in the $r^*$ direction |*s*| times, we see that

5.11 $$\begin{aligned}&(r^2+a^2)^{|s|+1/2}{\mathfrak {G}}_{(0)}\overline{\left(\frac{\mathcal {L}}{w(r^2+a^2)}\right)^{2|s|}\left((r^2+a^2)^{|s|-1/2}\uppsi _{(0)}\right)}\nonumber \\&\quad ={{\,\textrm{sign}\,}}s\sum _{j=1}^{|s|} \frac{\textrm{d}}{\textrm{d}r^*}\Big \{ \Big ((r^2+a^2)^{|s|-j+1/2}\sum _{k=0}^{j-1}p_{s,j-1,k}(r)\frac{{\mathfrak {G}}_{(k)}}{w}\Big )\nonumber \\&\qquad \times \overline{\left(\frac{\mathcal {L}}{w(r^2+a^2)}\right)^{|s|-j}\Big ((r^2+a^2)^{-1/2}\sum _{k=0}^{|s|}p_{s,|s|,k}(r)\uppsi _{(k)}\Big )} \Big \}\nonumber \\&\qquad +\Big (\sum _{k=0}^{|s|}p_{s,|s|,k}(r){\mathfrak {G}}_{(k)}\Big )\Big (\sum _{k=0}^{|s|}p_{s,|s|,k}(r)\overline{\uppsi _{(k)}}\Big )\,. \end{aligned}$$

The boundary terms at $r^*=-\infty$ vanish by the boundary conditions on $\uppsi _{(k)}$ and decay assumptions on $\mathfrak {G}_{(k)}$; the same is true at $r^*=+\infty$ for s>0. □

### Basic estimates from the transport equations

In this section, we derive some basic estimates on the transformed variables which follow from the transport equations (4.17).

#### Lemma 5.5

*(Basic transport estimate 1)* Let $$c(r^*):\mathbb {R}\rightarrow \mathbb {R}$$ be a bounded, continuous and piecewise continuously differentiable function, and assume $c^{\prime }\neq 0$ almost everywhere. For some $$R_-^*,R_+^*\in \mathbb {R}\cup \{\pm \infty \}$$, we have

5.12 $$\begin{aligned} \int _{R_-^*}^{R_+^*} c'\mathbb {1}_{\{c'>0\}}\left|\uppsi _{(k)}\right|^2\textrm{d}r^*&\leqq \int _{R_-^*}^{R_+^*} \left(\frac{8w^2c^2}{|c'|}\left|\uppsi _{(k+1)}\right|^2+3|c'|\mathbb {1}_{\{c'<0\}}\left|\uppsi _{(k)}\right|^2\right)\textrm{d}r^* \nonumber \\&\qquad +2c(R_+^*)\left|\uppsi _{(k)}\right|^2(R_+^*)-2c(R_-^*)\left|\uppsi _{(k)}\right|^2(R_-^*)\,. \end{aligned}$$

5.13 $$\begin{aligned} \int _{R_-^*}^{R_+^*} c'\mathbb {1}_{\{c'>0\}}\left|\uppsi _{(k)}'\right|^2\textrm{d}r^*&\leqq 12\int _{R_-^*}^{R_+^*} \left(\frac{w^2c^2}{|c'|}\left|\uppsi _{(k+1)}'\right|^2+ |c'|\mathbb {1}_{\{c'<0\}}\left|\uppsi _{(k)}'\right|^2\right)\textrm{d}r^* \nonumber \\&\qquad + 12\int _{R_-^*}^{R_+^*}\left(\frac{(w')^2c^2}{|c'|}\left|\uppsi _{(k+1)}\right|^2\right.\nonumber \\&\qquad \left.+\frac{16w^2c^2r^2}{|c'|(r^2+a^2)^2}a^2m^2\left|\uppsi _{(k)}\right|^2\right)\textrm{d}r^*\nonumber \\&\qquad +2c(R_+^*)\left|\uppsi _{(k)}\right|^2(R_+^*)-2c(R_-^*)\left|\uppsi _{(k)}\right|^2(R_-^*)\,. \end{aligned}$$

#### Proof

Estimate (5.12) follows from Cauchy–Schwarz applied to the multiplier identity one may derive by multiplying (4.17) by $c(r^*)\overline{\uppsi _{(k)}}$, take the real part and integrating in $r^*$. Estimate (5.12) follows in the same way, after commuting (4.17) with $\partial _{r^*}$. □

## Frequency Space Estimates

In this section, we prove the main result of the paper, Theorem 1. The result concerns solutions of the transformed system of radial ODEs (4.19). Concretely, in this section, for k=0,⋯,|s|, the main objects are the solutions $\uppsi _{({k}),\,m\Lambda }^{[{s}],\,a,\,\omega }$, defined via (4.15) and (4.17), from a smooth solution $\upalpha _{ml}^{[{s}],\,a,\,\omega }$ to the inhomogeneous radial ODE (4.7) with outgoing boundary conditions as in Definition 4.5; thus, $\uppsi _{({k}),\,m\Lambda }^{[{s}],\,a,\,\omega }$ are themselves solutions to the inhomogeneous radial ODEs (4.19) with outgoing boundary conditions as in Definition 4.9.

A supporting role will be played by the inhomogeneities $\mathfrak G_{({k}),\,m\Lambda }^{[{s}],\,a,\,\omega }$ defined from (4.18), and by a series of auxiliary functions,

$$\begin{aligned} y_{(k)}, \hat{y}_{(k)}, f, h_{(k)}, \chi _1 \text { and } \chi _2, \end{aligned}$$

which are used to produce the final estimates (and in the first four cases take their name from the templates of Section 5.1). These interact to produce error terms which we denote by

6.1 $$\begin{aligned}&\sum _{k=0}^{|s|}\mathfrak {G}_k\cdot \left(f,y,h,\chi \right)\cdot \left(\uppsi _{(k)},\uppsi _{(k)}'\right) \nonumber \\&\quad := -2(y+\hat{y}+f)\operatorname {Re}\left[\mathfrak {G}\overline{\Psi }'\right]-(h+f')\operatorname {Re}\left[\mathfrak {G}\overline{\Psi }\right]\nonumber \\&\qquad +E\left(\chi _2\omega +\chi _1(\omega -m\upomega _+)\right)\operatorname {Im}\left[\mathfrak {G}\overline{\Psi }\right]\nonumber \\&\qquad -{{\,\textrm{sign}\,}}s \frac{1}{2}(-1)^{|s|} E_W(\omega -m\upomega _+)\chi _1 \operatorname {Im}\nonumber \\&\qquad \times \left[\Big (\sum _{k=0}^{|s|}p_{s,|s|,k}(r){\mathfrak {G}}_{(k)}\Big )\Big (\sum _{k=0}^{|s|}p_{s,|s|,k}(r)\overline{\uppsi _{(k)}}\Big )\right]\nonumber \\&\qquad + \frac{1}{2}(-1)^{|s|}\sum _{j=1}^{|s|} E_W\chi _1'\Big \{ \Big ((r^2+a^2)^{|s|-j+1/2}\sum _{k=0}^{j-1}p_{s,j-1,k}(r)\frac{{\mathfrak {G}}_{(k)}}{w}\Big )\nonumber \\&\qquad \times \overline{\left(\frac{\mathcal {L}}{w(r^2+a^2)}\right)^{|s|-j}\Big ((r^2+a^2)^{-1/2}\sum _{k=0}^{|s|}p_{s,|s|,k}(r)\uppsi _{(k)}\Big )}\Big \}\nonumber \\&\qquad +B\mathbb {1}_{\mathcal {F}_\textrm{unbdd}}\sum _{k=0}^{|s|-1}\left|\operatorname {Re}\left[\mathfrak {G}_{(k)}\overline{\uppsi _{(k)}}\right]\right|\nonumber \\&\qquad +B(\tilde{a}_0)\mathbb {1}_{\mathcal {F}_\textrm{bdd}}\sum _{k=0}^{|s|-1}E\left|\left(\chi _2\omega +\chi _1(\omega -m\upomega _+)\right)\operatorname {Im}\left[\mathfrak {G}_{(k)}\overline{\uppsi }_{(k)}\right]\right|\nonumber \\&\qquad +B\gamma (\tilde{a}_0)\sum _{k=0}^{|s|-1}\left|\left\rbrace (y_{(k)}+\hat{y}_{(k)})\operatorname {Re}\left[\mathfrak {G}_{(k)}\overline{\uppsi _{(k)}}'\right]+h_{(k)}\operatorname {Re}\left[\mathfrak {G}_{(k)}\overline{\uppsi _{(k)}}\right]\right\lbrace \right|\,, \end{aligned}$$

where $\tilde{a}_0\in (0,M)$ and γ is a real-valued function, $E,E_W>0$ are parameters, $\mathcal {F}_\textrm{bdd}\cup \mathcal {F}_\textrm{unbdd}\subset \mathcal {F}_\textrm{admiss}$ correspond to a frequency partition we will make precise in Section 6.2.3, and $p_{s,k,j}(r)$ are smooth polynomials of degree k-j which are regular at $r=r_+$, see Lemma 5.4. Here, and in the remainder of this paper, it will be useful to keep in mind the following notation and conventions.

### Notation 6.1

*(Sub- and superscripts)* For k=|s|, we have the following simplified notations:

$$\begin{aligned} \uppsi _{({|s|}),\,m\Lambda }^{[{s}],\,a,\,\omega }&=\Psi _{m\Lambda }^{[{s}],\,a,\,\omega }, \quad \mathcal {V}_{({|s|}),\,m\Lambda }^{[{s}],\,a,\,\omega }=\mathcal {V}_{m\Lambda }^{[{s}],\,a,\,\omega }, \quad \mathfrak G_{({|s|}),\,m\Lambda }^{[{s}],\,a,\,\omega }=\mathfrak G_{m\Lambda }^{[{s}],\,a,\,\omega }, \\&y_{(|s|)}= y, \quad \hat{y}_{(|s|)}= \hat{y}, \quad h_{(|s|)}= h, \end{aligned}$$

to that is we drop the subscript (|*s*|). We also routinely drop the sub- and superscripts $a,\omega , m, \Lambda$, as well as the superscript [*s*], unless doing so would cause an ambiguity.

### Notation 6.2

*(Constants in estimates)* We use *B* to denote possibly large positive constants and *b* to denote possibly small positive constants depending only on M>0 and $s\in \mathbb {Z}$. Whenever the constant depends, additionally, on another parameter that has not yet been fixed, say *x*, we write *B*(*x*) or *b*(*x*); we revert to *B* and *b* once it has been fixed. We also note the algebra of constants: Bb=B for upper bounds and Bb=b for lower bounds,

$$\begin{aligned} B+B=BB=B\,, \qquad b+b=bb=b\,,\qquad B+b=B\,, \qquad b^{-1}=B\,,\qquad \text {etc.} \end{aligned}$$

We conclude this preamble with an outline of the remainder of the paper:

- **Section** 6.1. We state the precise version of Theorem 1 as Theorem 6.3, as well as a refinement for bounded *m* solutions of the transformed system (for other refinements, we direct the reader to our preprint [151] and the second author’s PhD thesis [156]). We comment on the restrictions on *s*, and the frequency restrictions as |a|→M.
- **Section** 6.2. We provide an overview of the proof. We explain the rationale we apply in partitioning frequency space $\mathcal {F}_\textrm{admiss}$ into high frequencies ($\mathcal {F}_\textrm{unbdd}$) and bounded frequencies ($\mathcal {F}_\textrm{bdd}$), as well as further subdivisions, and the key (high-level) ideas for what properties of the ODEs to exploit in each regime and how. A complete list of the frequency subregimes we will consider separately is given in Section 6.2.3; the partition will involve many different parameters which will eventually be fixed.
- **Sections** 6.3 and 6.4. The estimates leading to Theorem 1 are obtained, respectively, for high frequencies and bounded frequencies. For each of the frequency subregimes we will consider within these sections, ODE estimates are made precise by a proposition whose proof is preceded by a detailed and technical explanation and a sketch of the relevant $y_{(k)}$, $\hat{y}_{(k)}$, *f*, $h_{(k)}$, $\chi _1$ and $\chi _2$ functions.
- **Section** 6.5. We combine the aforementioned propositions to produce the precise Theorem 6.3. It is in this section that we finally fix the parameters involved in the frequency partition of Section 6.2.3.

### Precise version of the main theorem

#### Theorem 6.3

(Radial ODE analysis I) Fix $s\in \{0,\pm 1,\pm 2\}$, M>0, $a_0\in (0,M)$, and $\delta _I\in (0,1]$ and $\delta _H\in (0,1)$. There are parameters $\omega _\textrm{high}>0$, $\varepsilon _\textrm{width}>0$, $\tilde{\omega }_\textrm{low}>0$, E>0 and $E_W>0$, such that for all $\tilde{\omega }_\textrm{low}>0$ and for all $\tilde{a}_0>0$ sufficiently small, letting $\omega _\textrm{low}(\tilde{a}_0)>0$, $\gamma (\tilde{a}_0) >0$ and $R^*_\textrm{large}(\tilde{a}_0,\tilde{\omega }_\textrm{low})>0$ denote smooth functions with the properties that $\omega _\textrm{low}(\tilde{a}_0),\gamma (\tilde{a}_0)\rightarrow 0$ and $R^*_\textrm{large}(\tilde{a}_0,\tilde{\omega }_\textrm{low})\rightarrow \infty$ as $\tilde{a}_0\rightarrow 0$, for any admissible

6.2 $$\begin{aligned}&(a,\omega ,m,\Lambda )\notin (a_0,M]\nonumber \times \left(\left\rbrace (\omega ,m,\Lambda ):{\Lambda }\leqq \varepsilon _\textrm{width}^{-1}\omega _\textrm{high}^2,\, \omega _\textrm{low}{<}|\omega |{<}\omega _\textrm{high},\, |\omega -m\upomega _{+}|\leqq \tilde{\omega }_\textrm{low},\, m\ne 0\right\lbrace \right.\nonumber \\&\quad \left.\cup \left\rbrace \Lambda \geqq \varepsilon _\textrm{width}\omega ^2,\,\, |\omega |\geqq \omega _\textrm{high},\,\,|m(\omega -m\upomega _+)|\sqrt{\varepsilon _\textrm{width}}\Lambda ,\,\,m\ne 0 \right\lbrace \right), \end{aligned}$$

there is a parameter $r_\textrm{trap}$ and a choice of functions $y_{(k)}$, $\hat{y}_{(k)}$, *f*, $h_{(k)}$, $\chi _1$ and $\chi _2$, for $0\leqq k\leqq |s|$, which depend on the frequency triple but satisfy the uniform bounds

6.3 $$\begin{aligned}  &   |r_\textrm{trap}|+|r_\textrm{trap}-r_+|^{-1}\leqq B(a_0,\tilde{\omega }_\textrm{low})\nonumber \\  &   \quad |y_{(k)}|+|\hat{y}_{(k)}|+|f|+|f'|+|h_{(k)}|+|\chi _1|+|\chi _2|\leqq B(\tilde{a}_0,\tilde{\omega }_\textrm{low}), \nonumber \\  &   \quad (|y'_{(k)}|+|\hat{y}_{(k)}'|+|f'|+|h_{(k)}|+|\chi _1'|+|\chi _2'|)(\mathbb {1}_{\{r^*\geqq 0\}}r^{1+\delta _I}\nonumber \\  &   \qquad +\mathbb {1}_{\{r^*\leqq 0\}}|r^*|^{1+\delta _H})\leqq B(\tilde{a}_0, \tilde{\omega }_\textrm{low}), \nonumber \\  &   \quad (|y+\hat{y}+f-1|+|\chi _2-1|)\mathbb {1}_{\{r^*\geqq 0\}}r^{\delta _I}\leqq B(\tilde{a}_0, \tilde{\omega }_\textrm{low}), \end{aligned}$$

such that the following estimate

6.4 $$\begin{aligned}&\left[\left(|\Psi '|^2+\omega ^2\left|\Psi \right|^2\right)_{r=\infty }+\left(|\Psi '|^2+(\omega -m\upomega _+)^2\left|\Psi \right|^2\right)_{r=r_+}\right]\nonumber \\&\qquad +b(\tilde{a}_0,\tilde{\omega }_\textrm{low}) \int _{r_+}^\infty \frac{\left(1-Mr^{-1}\right)^{\delta _H-1}}{r^{1+\delta _I}}|\Psi '|^2\textrm{d}r\nonumber \\&\qquad +b(\tilde{a}_0,\tilde{\omega }_\textrm{low}) \int _{r_+}^\infty \frac{1}{r^2}\left[\left(\frac{(r-M)}{r^2}\Lambda +\frac{\left(1-Mr^{-1}\right)^{\delta _H-1}}{r^{1+\delta _I}}\omega ^2\right)\right.\nonumber \\&\qquad \times \left.\left(1-\frac{r_\textrm{trap}(\omega ,m,\Lambda )}{r}\right)^2+\frac{(r-M)^2}{r^{4}}\right]|\Psi |^2\textrm{d}r\nonumber \\&\quad \leqq \int _{-\infty }^\infty \sum _{k=0}^{|s|}\mathfrak {G}_k\cdot \left(f,y,h,\chi \right)\cdot \left(\uppsi _{(k)},\uppsi _{(k)}'\right)\textrm{d}r^* \nonumber \\&\quad +\gamma (\tilde{a}_0)\mathbb {1}_{\mathcal {F}_\textrm{bdd}}\Big [|\uppsi _{(0)}|^2_{r=r_+} + \sum _{k=0}^{|s|-1}\left|w^{-1}\mathfrak G_{(k)}\right|^2_{r=r_+}\Big ]\nonumber \\&\qquad + B\mathbb {1}_{\mathcal {F}_\textrm{int,2}(\tilde{\omega }_\textrm{low})\cup \mathcal {F}_\textrm{low,3}(\tilde{\omega }_\textrm{low})}\sum _{k=0}^{|s|}\int _{-R^*_\textrm{large}(\tilde{a}_0,\tilde{\omega }_\textrm{low})}^{R^*_\textrm{large}(\tilde{a}_0,\tilde{\omega }_\textrm{low})}\left(|\uppsi _{(k)}'|^2+|\uppsi _{(k)}|^2\right)\textrm{d}r^*\,, \end{aligned}$$

where $\mathcal {F}_\textrm{int,2}$ and $\mathcal {F}_\textrm{low,3}$ are defined in Section 6.2.3 and the integrand on the first term of the right hand side is defined in (6.1). If $\mathfrak G_{(k)}=O(\Delta ^{|s|-k+1})$ as $r\rightarrow r_+$, we take $E_W>0$ and drop the penultimate term in (6.4); if $\uppsi _{(k)}$ are compactly supported in $r^*\in (-\infty ,\infty )$, we drop the last two terms in (6.4).

Analogously to the comparable result for s=0 in [57], we may also consider a version of Theorem 6.3 which holds for fixed *m*. The main advantage is that, in this case, the structure of trapping is simpler and, as we will show later in Lemma 6.10 (see also Remark 6.11) it is entirely outside the ergorregion. We thus have.

#### Theorem 6.4

(Radial ODE analysis II) Consider the setup of Theorem 6.3 once again. There is a choice of $\omega _\textrm{high}=\omega _\textrm{high}(m)$ such that (6.3), (6.4) and (6.1) hold but with the constants denoted by *b* and *B* (6.4) depending additionally on *m* and with either

$$\begin{aligned} r_\textrm{trap}=0 \text { or } (1+\sqrt{2})M<r_\textrm{trap}\leqq B(M,|s|)\,. \end{aligned}$$

Some remarks are now in order.

#### Remark 6.5

*(Lower level transformed variables)* In proving Theorems 6.3 and 6.4, we will not only control bulk terms of $\Psi ^{[{s}]}$ but, by the very nature of the proof, in fact control bulk terms of $\uppsi ^{[{s}]}_{(k)}$ for every k=0,⋯,|s|. For k<|s|, these do not experience any degeneration at trapping. We can furthermore control bulk terms in $(\uppsi ^{[{s}]}_{(k)})'$, without degenerations, at the cost of replacing $\mathfrak {G}^{[{s}]}_{(k)}$ by $(\mathfrak {G}^{[{s}]}_{(k)})'$ in the above. For brevity, we have opted not to include this more general statement here, but it will be useful to keep it in mind for the physical space analysis of [152].

#### Remark 6.6

*(Excluded frequency ranges)* Notice that, for all $|a|\leqq a_0$, the restrictions (6.2) in the statements of Theorems 6.3 and 6.4 do not apply. In particular, for any choice of *subextremal* $a_0< M$, Theorem 6.3 yields (uniform) estimates for all admissible frequencies with respect to $0\leqq |a|\leqq a_0$. However, it does not yield uniform estimates for all admissible frequencies with respect to any black hole parameter $0\leqq |a|\leqq M$: the restrictions (6.2) imply that we do not control frequencies close to the superradiant threshold uniformly as |a|→M. We should distinguish between several regimes:

- Bounded frequencies, to that is the first line in (6.2). In the case s=0, estimates for this regime were obtained by Gajic [70] after the research reported in this paper was concluded. The proof relies, among several other ingredients, on the bounds in [155] on the Wronskian associated to radial ODE (1.2). Such bounds were obtained for all $s\in \frac{1}{2}\mathbb {Z}$, and thus we expect one can handle the frequency range in the first line of (6.2) by a simple (if tedious) generalizations of the arguments of [70]. Doing so is, however, out of the scope of the present paper.
- High frequencies with bounded *m*, i.e the second line of (6.2) subject to the additional condition that $m^2\leqq c \Lambda$ for some appropriately small *c*. For s=0, estimates for this regime were similarly obtained by Gajic [70] after the research reported in this paper was concluded. The argument there relies on the use of a separated redshift-type current template. We expect this proof to be easily extendable to the s≠0 case, but do not include it here as we have chosen not to invoke any redshift-type currents in the present work.
- High frequencies with unbounded *m*, i.e the second line of (6.2) with $m^2> c\Lambda$. This regime remains unexplored in the literature, even in the s≠0 case.

#### Remark 6.7

*(Excluded integer spin parameters)* We will prove Theorems 6.3 and 6.4 by applying a combination of the virial and energy currents introduced in Section 5.1 to the radial (4.19). These equations are defined for any $s\in \mathbb {Z}$ and, indeed, the properties of the radial ODEs (4.19) that we appeal to in the construction of the virial currents and classical energy currents are valid for any integer *s*. However, in controlling the boundary terms in estimates (6.4), we rely on the specific properties of $\mathfrak {D}_{s,k}^{\mathcal {H}}$ and $\mathfrak {C}_s$ in a bounded frequency regime where $|\omega |\ll 1$, namely Lemma 6.36(iv). We may drop the requirement that |s|≦2 in Theorems 6.3 and 6.4 provided that these properties hold for general $s\in \mathbb {Z}$. We expect this to be the case: by the analytic dependence of $\mathfrak {D}_{s,k}^{\mathcal {H}}$ and $\mathfrak {C}_s$ on ω, it is enough to look at the case ω=0 where the Teukolsky ODE (4.7) becomes a hypergeometric equation and hence computing such constants is easier.

Let us note, however, that if we were to consider more general boundary conditions in our Theorems 6.3 and 6.4, we would likely require information about $\mathfrak {D}_{s,k}^{\mathcal {I}}$, $\mathfrak {D}_{s,k}^{\mathcal {H}}$ and $\mathfrak {C}_s$ in a regime $|\omega |\gg 1$; indeed, see the scattering theory in [156, Part I, Chapter 6] which first appeared in our preprint [151]. For |s|>2, one would encounter the important difficulty that, for the non-empty (see [43]) frequency regime where $\mathfrak C_s<0$, the Teukolsky–Starobinsky energy current of Section 5.1.3 is not coercive. For this reason, we do not expect that a scattering theory for the Teukolsky equation for |s|>2, if it exists, can be similar to that for |s|≦2.

The properties of the ODEs introduced in Section 4 allow us to, without loss of generality, take a≧0 in the remainder of the paper:

#### Lemma 6.8

(Reduction to a≧0) It is enough to prove Theorems 6.3 and 6.4 assuming a≧0.

#### Proof

Recall that $(\omega ,m,\Lambda )$ is an admissible frequency triple with respect to *s* and *a* if it satisfies the requirements in Definition 4.1. The constraints on Λ do not depend on the sign of either *a* or *m*, but only possibly on the sign of *am*. Thus, for k=0,...,|s|, all terms in the radial ODEs (4.19) are invariant under the a↦-a, m↦-m. The conclusion follows. □

### Overview of the proof and frequency space partition

In this section, we give an overview of our proof of Theorem 6.3. As is clear from the statement, the proof follows from applying suitable multiplier current identities to the radial ODEs (4.19). For the most part, these multipliers will have nontrivial dependence on $(\omega ,m,\Lambda )$; nevertheless there is a common theme to their construction: for each k=0,⋯,|s|, they are informed

- on the one hand by the behavior of the potentials $\mathcal {V}_{(k)}$ relative to the energy level $\omega ^2$;
- on the other hand, if s≠0, by the strength of coupling to $\uppsi _{(k+1)}$ (k<|s|) and/or $\uppsi _{(j)}$ (j<k, k>0).

To explain how we take into account (a), it is convenient to start by partitioning the space of admissible frequencies into two large ranges, that of bounded frequencies and that of unbounded, or high, frequencies. To be precise, let $\varepsilon _\textrm{width}>0$, which we will think of as being very small, and $\omega _\textrm{high}>0$, which we will think of as being very large, and define that

$$\begin{aligned} \mathcal {F}_\textrm{bdd}&:=\left\rbrace (\omega ,m,\Lambda ) \text { admissible }:\Lambda \leqq \varepsilon _\textrm{width}^{-1}\omega _\textrm{high}^2\,,\,\, |\omega |< \omega _\textrm{high}\right\lbrace \,,\\ \mathcal {F}_\textrm{unbdd}&:=\left\rbrace (\omega ,m,\Lambda ) \text {admissible}\right\lbrace \backslash \mathcal {F}_\textrm{bdd}\,. \end{aligned}$$

We describe our approach to these two regimes in the next two subsections. We start with $\mathcal {F}_\textrm{unbdd}$, as it is the one where our proof will be more familiar to the reader acquainted with the treatment of the s=0 case in [57].

#### The high frequencies

In this section, we will describe our proof of Theorem 6.3 in the the high frequency regime $\mathcal {F}_\textrm{unbdd}$, which is given in Section 6.3. We begin by noticing that, for k=|s|, the homogeneous radial ODE (4.21) in this regime takes the schematic form

6.5 $$\begin{aligned}  &   \Psi ''+\left(\omega ^2-\mathcal {V}_0\right)\Psi =aw\sum _{k=0}^{|s|-1}(1+im)\uppsi _{(k)}, \end{aligned}$$

6.6 $$\begin{aligned}  &   \mathcal {V}_0(r)=\frac{\Delta (\Lambda +s^2)+4Mram\omega -a^2m^2}{(r^2+a^2)^2}. \end{aligned}$$

It is natural to conjecture that, as the left hand side of (6.5) involves higher order quantities than the right hand side, the coupling on the right hand side should, in $\mathcal {F}_\textrm{unbdd}$, be a small perturbation. If this were the case, then one could reduce the study of (6.5) to that of the toy problem

6.7 $$\begin{aligned} \Psi ''+\left(\omega ^2-\mathcal {V}_0\right)\Psi =0. \end{aligned}$$

Let us begin by discussing this toy problem (6.7). The reader familiar with the s=0 proof of the first author with Dafermos and Rodnianski in [57] may recognize (6.7) as a minor modification of the radial ODEs studied there.^12^ However, as our approach to (6.7), even for s=0, is slightly different from that of [57], the reader familiar with that paper may nevertheless find our discussion below useful to understand the structure of Section 6.3.

In the toy problem (6.7), and in the more rigorous approximation (6.5), it is important to notice that the high frequency potential $\mathcal {V}_0$ has a much simpler expression than the full potential $\mathcal {V}$ from (4.21). Yet, for general |a|≦M, $\mathcal {V}_0$ is still reasonably involved: for example it has nontrivial dependence on all the frequency parameters, including the parameter ω which determines the energy level $\omega ^2$ with which $\mathcal {V}_0$ competes. This fact makes the energy picture for the Schrödinger toy problem (6.7) hard to sketch in general. Nevertheless, for such a problem we can clearly distinguish three regimes.

**Fig. 1:**
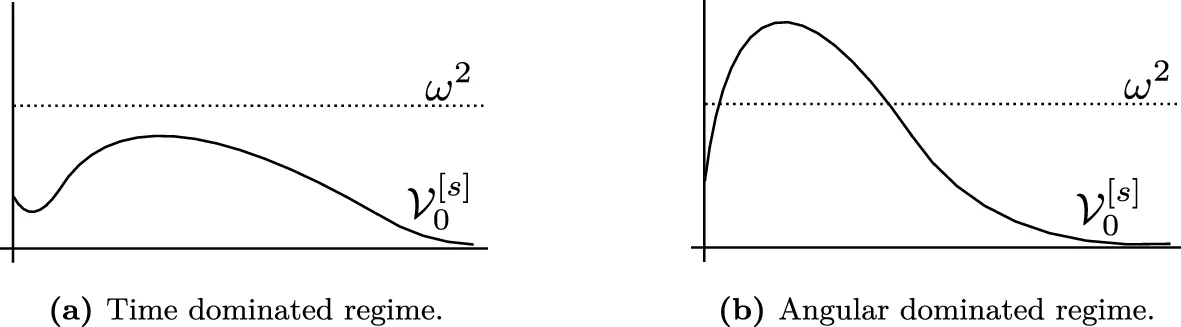
Sketch of $\mathcal {V}_0$ when one of $\omega ^2$ or Λ dominate

$\underline{{\mathrm{Time\,\, dominated\,\, regime}} (\omega ^2\gg \Lambda ).}$ In the Schrödinger toy problem (6.7) the potential is always dominated by the energy level, see Fig. 1a. This fact can captured with a suitable choice of *y* virial current, which leads to the desired integrated estimate for Ψ with $r_\textrm{trap}=0$, at the cost of boundary terms with a bad sign. From the admissibility conditions on (m,Λ), it is also manifest that the regime is non-superradiant, so a global energy current can be employed to control the boundary terms of Ψ.

Formally, we will define the time dominated regime by$\underline{{\mathrm{Angular\,\, dominated\,\, regime}}\,\, (\omega ^2\ll \Lambda ).}$ In this case, the maximum of the potential rises well above the energy level as in Fig. 1b, though asymptotically as $r^*\rightarrow \pm \infty$ the positivity of $\omega ^2-\mathcal {V}_0$ breaks down. This fact can be captured by an *h* current supported around the maximum. As we cannot rely on a good sign of $\omega ^2-\mathcal {V}_0$ away from the maximum, to absorb the resulting bulk error terms we are forced to employ an *f* virial current, which requires information on the sign and size of $\mathcal {V}_0$. In Section 6.3.1, a critical point analysis of $\mathcal {V}_0$ will produce the necessary input to construct *f* and conclude the proof of the desired integrated estimate for Ψ with $r_\textrm{trap}=0$, up to boundary terms with a bad sign.

In this frequency regime, superradiance is allowed. Therefore, to control the boundary terms, we employ localized versions of the energy currents associated to the Killing fields *T* and *K*. We will show that the strong positivity of $\omega ^2-\mathcal {V}_0$ allows the localization errors to be absorbed by the bulk estimates.

Formally, we will define the angular dominated regime by

$$\begin{aligned} \mathcal {F}_{\measuredangle }:=\left\rbrace (\omega ,m,\Lambda ) \text { admissible}:\Lambda \geqq \varepsilon _\textrm{width}^{-1}\omega ^2\,,\,\, \Lambda \geqq \varepsilon _\textrm{width}^{-1}\omega _\textrm{high}^2\right\lbrace . \end{aligned}$$

For some $\beta _1, \beta _2>0$ and $a_0\in (0,M)$, it will also be useful to consider the subregimes

$$\begin{aligned} \mathcal {F}_{\measuredangle ,1}&:=\left\rbrace a\in [0,a_0], \,(\omega ,m,\Lambda ) \text { admissible }:\Lambda \geqq \varepsilon _\textrm{width}^{-1}\omega ^2\,,\,\right. \nonumber \\&\left. \quad \Lambda \geqq \varepsilon _\textrm{width}^{-1}\omega _\textrm{high}^2\,\,\, m\omega \leqq m^2\upomega _++\beta _1\Lambda \right\lbrace ,\\ \mathcal {F}_{\measuredangle ,2}&:=\left\rbrace a\in (a_0,M], \, (\omega ,m,\Lambda ) \text { admissible }:\Lambda \geqq \varepsilon _\textrm{width}^{-1}\omega ^2\,,\right.\nonumber \\&\left. \quad \, \Lambda \geqq \varepsilon _\textrm{width}^{-1}\omega _\textrm{high}^2\,,\,\, m\omega \leqq m^2\upomega _+-\beta _2\Lambda \right\lbrace ,\\ \mathcal {F}_{\measuredangle ,3}&:=\left\rbrace a\in (a_0,M], \,(\omega ,m,\Lambda ) \text { admissible }:\Lambda \geqq \varepsilon _\textrm{width}^{-1}\omega ^2\, , \right. \nonumber \\&\left. \quad \, \Lambda \geqq \varepsilon _\textrm{width}^{-1}\omega _\textrm{high}^2\,,\,\, m\omega \geqq m^2\upomega _+-\beta _2\Lambda \right\lbrace , \end{aligned}$$

for reasons which will hopefully become clearer in the ensuing discussion.

$\underline{{\mathrm{Comparable\,\, regime}}~(\omega ^2\sim \Lambda ).}$ This regime is the complement, in $\mathcal {F}_\textrm{unbdd}$, of the previous two and thus can be defined by

$$\begin{aligned} \mathcal {F}_\textrm{comp}:=\left\rbrace (\omega ,m,\Lambda ) \text { admissible}:\varepsilon _\textrm{width}\omega ^2\leqq \Lambda \leqq \varepsilon _\textrm{width}^{-1}\omega ^2\,,\,\, |\omega |\geqq \omega _\textrm{high}\right\lbrace . \end{aligned}$$

In this regime, it is not *a priori* clear for what, if any, $r\in [r_+,\infty )$ we have $\omega ^2$ dominating $\mathcal {V}_0$ or vice-versa. For that reason, we will find it convenient to subdivide the regime further. To this end, we will introduce new parameters $r_0'\in [r_+,\infty )$, and $\beta _1,\beta _2>0$.

**Fig. 2:**
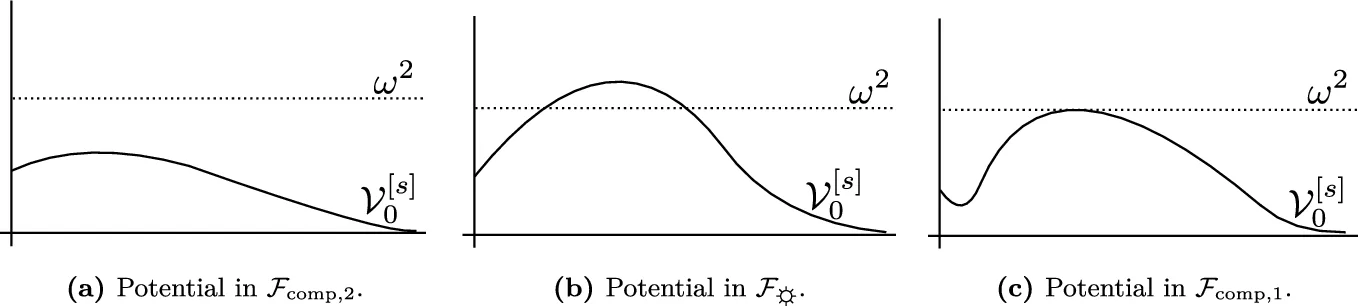
Sketch of $\mathcal {V}_0$ when $\omega ^2$ and Λ are comparable

- *Non-trapped regime*: this is a setting where there the critical points of the potential $\mathcal {V}_0$ are quantitatively away from (to that is either above or below) the energy level.
  - Let us first consider the case where all the critical points of $\mathcal {V}_0$ are below the energy level (denoted below by $r_0'=\infty$). In Section 6.3.1, we will show that a sufficiently condition on $(\omega ,m,\Lambda )$ for this condition on $\mathcal {V}_0$ to be satisfied is that they are sufficiently (a parameter $\beta _3>0$ is meant to measure this) non-superradiant frequencies. We therefore set:
    $$\begin{aligned} \mathcal {F}_\textrm{comp,2}&:=\{(\omega ,m,\Lambda ) \text { admissible}:\\ \varepsilon _\textrm{width}\omega ^2\leqq \Lambda&\leqq \varepsilon _\textrm{width}^{-1}\omega ^2\,,\,\, |\omega |\geqq \omega _\textrm{high}\,,\,\, r_0'=\infty \,,\\&\qquad \qquad m\omega \notin (0, m^2\upomega _++\beta _3\Lambda )\}. \end{aligned}$$
    Since the behavior of $\mathcal {V}_0$ in $\mathcal {F}_\textrm{comp,2}$ is similar to what we observed in  above, see Fig. 2asimilar and we will likewise set $r_\textrm{trap}=0$ in $\mathcal {F}_\textrm{comp,2}$. Due to their similarity, we have chosen to present the proofs of Theorem 6.3 in $\mathcal {F}_\textrm{comp,2}$ and  together in Section 6.3.5 below.
  - In Section 6.3.1, we will show that for sufficiently (as measured by some $\beta _1,\beta _2$) superradiant frequencies, the critical points of $\mathcal {V}_0$ are above the energy level. We denote such frequencies by , where  Clearly, the Schrödinger problem (6.7) looks very similar in  as it does in $\mathcal {F}_{\measuredangle }$, see Fig. 2bTheorem 6.3 in  and $\mathcal {F}_{\measuredangle }$ together in Section 6.3.3 below. In particular, we will choose $r_\textrm{trap}=0$ in both cases.
- *Trapped regime*: this is a setting where there are critical points of the potential which are arbitrarily close to the energy level. We thus expect to have $r_\textrm{trap}\ne 0$ in general. We can subdivide even further:
  - Trapped and sufficiently (as measured by some $\beta _1>0$) non-superradiant regime, denoted by $\mathcal {F}_\textrm{comp,1}$, and defined via
    $$\begin{aligned} \mathcal {F}_\textrm{comp,1}&:=\{(\omega ,m,\Lambda ) \text { admissible}:\varepsilon _\textrm{width}\omega ^2\leqq \Lambda \leqq \varepsilon _\textrm{width}^{-1}\omega ^2\,,\,\, \\&\qquad \qquad |\omega | \geqq \omega _\textrm{high}\,,\,\, r_0'<\infty \,,m\omega \notin (0, m^2\upomega _++\beta _3\Lambda )\}. \end{aligned}$$
    In Section 6.3.1, a critical point analysis of $\mathcal {V}_0$ will show that there is a unique critical point of $\mathcal {V}_0$ which can be arbitrarily close to $\omega ^2$, and it is a maximum at $r=r_\textrm{max}^0$, see also Fig. 2c. For *r* such that $\omega ^2-\mathcal {V}_0(r)$ is an unsigned quantity, Section 6.3.1 provides quantitative estimates for $\mathcal {V}'_0$ that can be exploited through an *f* multiplier current. This multiplier will lead to the desired integrated estimates for Ψ with $r_\textrm{trap}=r_\textrm{max}^0$, at the cost of producing badly signed boundary terms. Notice that, since $\mathcal {V}_0(r_\textrm{max}^0)\sim \omega ^2$, we cannot expect to absorb bulk errors arising from localization of energy currents in the manner described above in $\mathcal {F}_{\measuredangle }$. This is where the non-superradiant assumption comes in: in the absence of superradiance, a global *T*-based energy current does not, of course, generate localization errors, and thus will conclude the proof.
  - Trapped and superradiant regime. The preceding item makes the difficulties of this regime apparent: a weak bulk term might not be enough to control localization errors from the energy currents needed to control the boundary terms for superradiant frequencies. In Section 6.3.1, we will show that superradiant frequencies cannot be trapped unless |a|≈M and $\omega \approx m\upomega _+$, see Lemma 6.14 and Remark 6.15. In particular, for any choice of $a_0\in [0,M)$ subextremal, if we have $|a|\leqq a_0$, then we can tune the rigorous definitions of $\mathcal {F}_\textrm{comp,1}$ and , to that is the choices of $\beta _1,\beta _2, \beta _3$ and thus the definition of *sufficiently* (non-)superradiant, with respect to $a_0$ to show that . This fundamental insight, that superradiant frequencies are nontrapped for subextremal Kerr, is originally due to [55, 57] (see also [47]). If, however, we allow $a_0 <|a|\leqq M$, then  may be non-empty and, in that case, contains frequencies very close to the superradiant threshold $\omega =m\upomega _+$. Indeed, we group such frequencies in $\mathcal {F}_\textrm{comp,3}$ defined via
    $$\begin{aligned} \mathcal {F}_\textrm{comp,3}&:{=}\{(\omega ,m,\Lambda ) \text { admissible}:\varepsilon _\textrm{width}\omega ^2\leqq \Lambda \leqq \varepsilon _\textrm{width}^{-1}\omega ^2\,,\, |\omega |\geqq \omega _\textrm{high},\\&\qquad \qquad m^2\upomega _+-\beta _2\Lambda< m\omega <m^2\upomega _++\beta _1\Lambda ) \}. \end{aligned}$$
    Notice that, in $\mathcal {F}_\textrm{comp,3}$, $m^2\sim \omega ^2\gg 1$ is unbounded. This means that the $\mathcal {F}_\textrm{comp,3}$ regime is *excluded* from the fixed *m* statements of Theorem 6.4. Furthermore, as current work on extremal Kerr has focused on the case of bounded *m*, see [19, 70, 155], obtaining estimates in $\mathcal {F}_\textrm{comp,3}$ is, at this point, an open problem which we do not tackle here.

Let us consider what happens when we repeat the approach outlined above for the toy problem (6.7) to the true approximate problem (6.5) with s≠0. Repeating the multiplier currents we described, we will obtain estimates of the form of Theorem 6.3, but with errors arising from the interaction of the multiplier currents with the coupling terms in the right hand side of (6.5); see Section 5.1. In particular, application of virial currents will lead to errors schematically of the form

6.8 $$\begin{aligned}&(1+m)(1-r^{-1}r_\textrm{trap})\operatorname {Re}[\uppsi _{(k)}\overline{\Psi }]+ (1+m)\operatorname {Re}[\uppsi _{(k)}\overline{\Psi }'] \nonumber \\&\quad \lesssim \varepsilon (|\Psi |^2+|\Psi '|^2)+\varepsilon ^{-1}(1+m^2)|\uppsi _{(k)}|^2 \end{aligned}$$

for some $\varepsilon \in (0,1)$. In turn, application of the Killing energy currents leads to errors which, upon applying the transport estimates of Lemma 6.20, are controlled by

6.9 $$\begin{aligned} \varepsilon |\Psi '|^2+\varepsilon [1+(m^2+\omega ^2)]\left(1-\frac{r_\textrm{trap}}{r}\right)^2|\Psi |^2 +\varepsilon ^{-1}\sum _{k=0}^{|s|-1} (1+m^2)|\uppsi _{(k)}|^2 \end{aligned}$$

for some $\varepsilon \in (0,1)$. By the discussion above, the terms in (6.8) and (6.9) which involve Ψ can be absorbed by the current multipliers’ good bulk terms if ε is sufficiently small. Thus, the only terms we have yet to control are bulk errors

6.10 $$\begin{aligned} \sum _{k=0}^{|s|-1} (1+m^2)|\uppsi _{(k)}|^2. \end{aligned}$$

Here and in the schematic (6.8) and (6.9) we have suppressed the *r* weights as $r^*\rightarrow \pm \infty$ for simplicity.

In Section 5.2, we saw that basic transport estimates for (4.17) yield $|\uppsi _{(k)}|^2\lesssim |\Psi |^2$ (suppressing any *r* weights in the region $r^*\rightarrow \pm \infty$ and the *r* integration); see Lemma 5.5. Thus, from (4.17) the transport equations alone, we can expect the errors in (6.10) for general^13^|a|≦M to be

6.11 $$\begin{aligned} (4.17) \implies \sum _{k=0}^{|s|-1} (1+m^2)|\uppsi _{(k)}|^2\lesssim (1+m^2)|\Psi |^2, \end{aligned}$$

with suitable *r*-weights as $r^*\rightarrow \pm \infty$, and after integrating in *r*. Note that there is no degeneration at trapping for the *m*-derivatives, which means this expression *cannot* be absorbed by the positive bulk terms generated by our multiplier currents.

In Section 6.3.2, we will show that we can often improve the conclusion of (6.11) by estimating the radial ODEs for $\uppsi _{(k)}$, (4.19), in lieu of the transport equation (4.17). More accurately, we show that the constraint equation (4.23) can be viewed as an elliptic equation for $\uppsi _{(k)}$, k<|s|, in terms of $\uppsi _{(k+1)}$ and $\uppsi _{(j)}$, j<k, allowing us to show that

6.12 $$\begin{aligned} (4.23) + \text {frequency restrictions} \implies \sum _{k=0}^{|s|-1} (1+m^2)|\uppsi _{(k)}|^2\lesssim |\Psi |^2, \end{aligned}$$

schematically, with suitable *r*-weights as $r^*\rightarrow \pm \infty$, and after integrating in *r*. Crucially, the frequency restrictions needed in (6.12) are met whenever $r_\textrm{trap}\ne 0$; see Lemma 6.26, to that is in $\mathcal {F}_\textrm{comp,1}$ in particular. An immediate corollary is that $\uppsi _{(k)}$ with k<|s| do not experience trapping. The frequency restrictions are also met in $\mathcal {F}_{\measuredangle }$ and , see already Lemma 6.23, but they are *not* met in the frequency ranges .

To conclude our overview, therefore, we need only explain how to tackle (6.10) in the frequency regimes  (time dominated) and $\mathcal {F}_\textrm{comp,2}$ (comparable, non-superradiant, non-trapped). Note that, in the former, $\omega ^2\gg \Lambda$ and thus we can show in particular that $m^2\ll \omega ^2$. Consequently, the errors (6.10) are, in fact, much smaller than the positive bulk terms generated by the *y* multiplier current described above. This argument cannot be adapted to the $\mathcal {F}_\textrm{comp,2}$ regime: there, $\omega ^2\sim \Lambda$ and so the errors (6.11), though not too high in frequency weights (since $r_\textrm{trap}=0$ on $\mathcal {F}_\textrm{comp,2}$), do not have the necessary smallness parameter for us to close the estimates. In $\mathcal {F}_\textrm{comp,2}$, our strategy will rely on an application of an identical analysis, *y* multiplier currents, to each of the radial ODEs (4.19) in the transformed system; see 6.3.5 for more details.

We emphasize that our approach to $\mathcal {F}_\textrm{comp,2}$ is philosophically very different to the other high frequency regimes. Indeed, as we explained, in $\mathcal {F}_\textrm{comp,1}$, $\mathcal {F}_{\measuredangle }$,  and , we can treat the radial ODEs (4.19) very differently depending on *k*: for k=|s|, we deploy the full arsenal of microlocal current estimates, in the manner explained for the toy problem (6.7) above; for k<|s|, without the invoking multiplier currents and by a very easy argument, we show an elliptic estimate in some frequency regimes. In turn, in $\mathcal {F}_\textrm{comp,2}$, our treatment is more uniform across k=0,⋯,|s|, in that we will apply microlocal current estimates, in the manner explained for the toy problem (6.7) above, to all of (4.19). This makes the $\mathcal {F}_\textrm{comp,2}$ regime^14^ the closest in spirit (and, consequently, in our presentation) to the bounded frequencies regime which we will describe next.

#### The bounded frequencies

In this section, we give a brief overview of our approach to the bounded frequency regimes covered by Theorems 6.3 and 6.4, in particular contrasting with the case of high frequencies. In the latter, our starting point was the radial ODE

6.13 $$\begin{aligned} \Psi ''+\left(\omega ^2-\mathcal {V}\right)\Psi =\mathfrak {G}+aw\sum _{k=0}^{|s|-1}\left(im c_{s,\,k}^{\Phi }+ c_{s,\,k}^{\textrm{id}}\right)\uppsi _{(k)}, \end{aligned}$$

which we attempted to treat using the scalar wave techniques for s=0 introduced in [57]. To show that these could be employed, we had to show that the coupling on the right hand side of (6.13) was lower order; intuitively, we could hope that this was the case at least outside of trapping, when $\omega ^2-\mathcal {V}$ is quadratic in the (large) frequencies and the coupling is at most linear. In contrast, if $(\omega ,m,\Lambda )$ are bounded, both the left and right hand sides of the (6.13) are of the same order in frequency. Thus, at least in a bounded region of $|r^*|$, the coupling terms on the right hand side of (6.13) are *not* lower order in $\mathcal {F}_\textrm{bdd}$ and so there is no reason to expect that scalar wave techniques are sufficient for (6.13).

For general |a|≦M, our strategy for bounded frequencies will be to replace (6.13) by the entire system of radial ODEs given by (4.19). For each k=0,⋯,|s|, scalar wave techniques will produce estimates for $\uppsi _{(k)}$ in terms of bulk terms in j<k, if k>0, and k+1, if k<|s|. The coupling to j<k, as we have seen, in general does not allow one to advice smallness in a bounded $|r^*|$ region. The exception to this rule will be the case where *a* can be taken to be a smallness parameter, as (6.13) can, and will, be the starting point in our proofs in that case.

$\underline{{\textrm{Low}}\,\, T\,\, {\mathrm{frequency\, regime}}(\omega ^2\ll 1).}$ For such frequencies, we will show in Section 6.4.1 that $\mathcal {V}_{(k)}$ is sufficiently positive, and dominates $\omega ^2$ in an interval $r^*\in [R_-^*,R_+^*]$ where $R_-^*<R_+^*$ and $R^*_+-R_-^*$ is large enough depending on the smallness of $\omega ^2$. We can exploit this positivity with a compactly supported *h* current. As seen in Lemma 5.1, the *h* current does not produce coupling terms with k+1, but only with j<k which, as expected, do not come with any smallness. It also produces bulk errors due to its compact support.

By taking $\omega ^2$ small enough, and thus $R_+ ^*-R_- ^*$ large enough, we will show in Section 6.4.1 that $\mathcal {V}_{(k)}'$ has a well defined sign for $r^*\geqq R_+^*$. For $r^*\leqq R_-^*$, either it has a well defined (opposite) sign or we have $(\omega -m\upomega _+)^2\sim 1$. To exploit either one of these characteristics, we rely on *y* currents. The *y* currents produce several types of errors, according to the formulas obtained in Lemma 5.1 above:

- Boundary terms, which we must control through energy currents.
- Bulk errors due to coupling terms, and note these also arise from the energy currents if they are modeled on the Killing energies of Lemma 5.3. Coupling terms to j<k do not in general come with any smallness. Thus, in order to close our estimates, we must produce some smallness in the coupling to k+1.

The energy currents, in addition to possible coupling terms mentioned above, generate localization errors if, due to superradiance, we must add them in a localized fashion.

Let us first consider the case where $(\omega -\upomega _+)^2\ll 1$, which we will refer to as $\mathcal {F}_\textrm{low,1}$. Concretely, since, in a regime where we already have $\omega ^2\ll 1$, the condition $(\omega -m\upomega _+)^2\ll 1$ can be satisfied only if a≪1 or m=0 (recall that $m\in \mathbb {Z}$ is discrete), $\mathcal {F}_\textrm{low,1}$ is defined as the union of the frequency regimes

$$\begin{aligned} \mathcal {F}_\textrm{low,1a}&:=\left\rbrace a\in [0,\tilde{a}_0]\,,\,\, (\omega ,m,\Lambda ) \text { admissible }:\right.\\&\qquad \left. \Lambda \leqq \varepsilon _\textrm{width}^{-1}\omega _\textrm{high}^2\,,\,\, |\omega |\leqq \omega _\textrm{low}\right\lbrace ,\\ \mathcal {F}_\textrm{low,1b}&:=\left\rbrace a\in [0,M-\tilde{a}_1)\,,\,\, (\omega ,m,\Lambda ) \text { admissible }:\right.\\&\qquad \left. \Lambda \leqq \varepsilon _\textrm{width}^{-1}\omega _\textrm{high}^2\,,\,\, |\omega |\leqq \omega _\textrm{low}\,,\,\, m=0\right\lbrace ,\\ \mathcal {F}_\textrm{low,1c}&:=\left\rbrace a\in [M-\tilde{a}_1,M]\,,\,\, (\omega ,m,\Lambda ) \text { admissible }:\right.\\&\qquad \left.\Lambda \leqq \varepsilon _\textrm{width}^{-1}\omega _\textrm{high}^2\,,\,\, |\omega |\leqq \omega _\textrm{low}\,,\,\, m=0\right\lbrace , \end{aligned}$$

where $\varepsilon _\textrm{width},\omega _\textrm{high}>0$ are thought of as fixed parameters, and $\omega _\textrm{low}>0$, $\tilde{a}_0, \tilde{a}_1>0$ are thought of as small parameters to be fixed. In $\mathcal {F}_\textrm{low,1}$, as superradiance is small, the both any coupling errors as well as any localization errors arising from the energy currents are small themselves. Furthermore, $\mathcal {V}_{(k)}'$ has a well defined sign for $r^*\geqq R_+^*>0$ and $r^*\leqq R_-^*<0$, as we will show in Section 6.4.1; for such large $|r^*|$, due to the *r*-weights, we will show that there is smallness in the coupling to k+1.

To conclude the proof in $\mathcal {F}_\textrm{low,1}$, we need to justify that we can connect the *h* and *y* currents in such a way as to eliminate the localization errors due to *h*; we will show that this can be done as $r^*\rightarrow \infty$ by the same approach as in the s=0 case in [57, Section 8.7], see already Lemma 6.39 in Section 6.4.2 below, but not quite so for $r^*\rightarrow -\infty$ unless a≈M ($\mathcal {F}_\textrm{low, 1c}$) or k=|s|. Nevertheless, for |a|<M and k<|s| ($\mathcal {F}_\textrm{low,1b}$ and possibly $\mathcal {F}_\textrm{low, 1a}$), we can overcome this additional difficulty using the expoentially-strong decay of Δ as $r\rightarrow r_+$; we defer to the comments Section 6.4.3 for more precise details. Finally, note that if *a* is very small ($\mathcal {F}_\textrm{low, 1a}$), though the strategy outlined for |a|<M also works, an easier approach is to consider only the k=|s| equation (6.13) and use the smallness of *a* and transport estimates (rather than complicated virial currents) to control the errors due to coupling to j<k=|s|.

Next, we turn to the case $(\omega -\upomega _+)^2\sim 1$ or, more precisely, to

$$\begin{aligned}\mathcal {F}_\textrm{low,2}&:=\left\rbrace a\in (\tilde{a}_0,M]\,,\,\,(\omega ,m,\Lambda ) \text { admissible }:\right.\\  &\qquad \left. \Lambda \leqq \varepsilon _\textrm{width}^{-1}\omega _\textrm{high}^2\,,\,\, |\omega |\leqq \omega _\textrm{low}\,,\,\, m\ne 0\right\lbrace ,\end{aligned}$$

where $\varepsilon _\textrm{width},\omega _\textrm{high}, \tilde{a}_0>0$ are thought of as fixed parameters, and $\omega _\textrm{low}>0$ is thought of as a small parameter to be fixed. In the region $r^*\rightarrow \infty$, the potential in $\mathcal {F}_\textrm{low,2}$ behaves in exactly the same way as in $\mathcal {F}_\textrm{low,1}$. It is for this reason that we introduce our template for the *h* and *y* currents for large $r^*$ in $\mathcal {F}_\textrm{low,1}\cup \mathcal {F}_\textrm{low,2}$ already in Section 6.4.2 below, Lemma 6.39. For the remaining $r^*$ values, we can exploit the non-vanishing of $(\omega -\upomega _+)^2$; this is very similar to what happens in the frequency regimes  (though there it is $\omega ^2$ which does not vanish), and so likewise we can exploit this property by constructing an exponential-type *y* multiplier. The *h* current rises from 0 so that it is initially anchored to the exponential *y* multiplier and thus its compact support does not introduce too-large errors.

Finally, let us discuss the errors arising from the energy currents in $\mathcal {F}_\textrm{low,2}$. While any *T*-based energy current will still enjoy the smallness of ω in this regime, the *K*-based energy currents do not, as $|\omega -\upomega _+|\sim 1$. Thus, the coupling errors arising from application of a *K*-type Killing energy current cannot be controlled, and we rely instead on a *K*-type Teukolsky–Starobinsky energy current, which is exactly conserved if $\mathfrak G_{(k)}\equiv 0$ and thus does not generate any coupling errors, see Lemma 5.4. This is the only point of the proof where we appeal to this result and, therefore, appeal to any statements on the Teukolsky–Starobinsky constants, though see our preprint [151] and the thesis [156] for a comparison to the scattering problem.

$\underline{{\textrm{Nonzero}}\,\, T\,\, {\mathrm{frequency\,\, regime}}\,\, (\omega ^2\sim 1).}$ In this case, we do not attempt to completely close integrated-in-$r^*$ estimates for the system: we only seek to obtain closed estimates for large $|r^*|$ (including the boundary terms) but are content with producing errors in a compact $r^*$-region. A *y* current near r=∞ will take advantage of the fact that ω is nonzero, and a localized *T* energy current will control the boundary terms produced, at the cost of a bounded $|r^*|$ error. The same strategy is used for $r\rightarrow r_+$ as long as $|\omega -m\upomega _+|\sim 1$. In the case of $|\omega -m\upomega _+|\ll 1$, where a *y* current near $r=r_+$ can lose control over the zeroth order bulk term, we obtain estimates near $r=r_+$ in the subextremal case by exploiting (a version of) the redshift effect through a Hardy estimate. Thus, we can show our result for $(\omega ,m,\Lambda )$ in one of

$$\begin{aligned} \mathcal {F}_\textrm{int,1}&:=\left\rbrace a\in [0,M-\tilde{a}_1)\,, (\omega ,m,\Lambda ) \text { admissible }:\right.\\&\qquad \left.\Lambda \leqq \varepsilon _\textrm{width}^{-1}\omega _\textrm{high}^2\,,\,\, \omega _\textrm{low}<|\omega |< \omega _\textrm{high}\right\lbrace ,\\ \mathcal {F}_\textrm{int,2}&:=\left\rbrace a\in [M-\tilde{a}_1,M]\,, (\omega ,m,\Lambda ) \text { admissible }:\right.\\  &\qquad \left. \Lambda \leqq \varepsilon _\textrm{width}^{-1}\omega _\textrm{high}^2\,,\,\, \omega _\textrm{low}<|\omega |< \omega _\textrm{high}\,,\right.\\&\qquad \left. |\omega -m\upomega _+|> \tilde{\omega }_\textrm{low}\right\lbrace . \end{aligned}$$

where $\varepsilon _\textrm{width},\omega _\textrm{high}, \omega _\textrm{low}, \tilde{\omega }_\textrm{low}>0$ are thought of as fixed parameters, and $\tilde{a}_1$ is thought of as a small parameter to be fixed. We do not obtain estimates here in the complementary range, where a→M and $|\omega -m\upomega _+|\rightarrow 0$; more precisely, for the frequency range

$$\begin{aligned} \mathcal {F}_\textrm{low,3}&:=\left\rbrace a\in [M-\tilde{a}_1,M]\,,\,\, (\omega ,m,\Lambda ) \text { admissible}:\right.\\&\qquad \left. \Lambda \leqq \varepsilon _\textrm{width}^{-1}\omega _\textrm{high}^2\,,\,\, \omega _\textrm{low}<|\omega |< \omega _\textrm{high}\,,\,\,\right.\\&\qquad \qquad \left. |\omega -m\upomega _+|\leqq \tilde{\omega }_\textrm{low}\,,\,\, m\ne 0\right\lbrace . \end{aligned}$$

After the present work was completed, the regime $\mathcal {F}_\textrm{low,3}\cap \{|a|=M\}$ was addressed by Gajic in [70].

In $\mathcal {F}_\textrm{int,1}$ and $\mathcal {F}_\textrm{int,2}$, integrated-in-$r^*$ estimates for bounded $|r^*|$ are obtained for some choices of inhomogeneities $\mathfrak {G}_{(k)}$ in our companion paper [152], using the quantiative mode stability results of the two authors in [150, 155]. However, it is instructive to note that an alternative proof is available for |a|≪M: in that case, as $|\omega -m\upomega _+|\ll 1$, the strength of superradiance is very small and $\mathcal {V}_{(k)}$ agrees with the the (good) a=0 potential to leading order. We can extend the *y* current we just described for $\mathcal {F}_\textrm{int, 1}$ near r=∞ to the entire $r\in (r_+,\infty )$ range by making it decay exponentially fast as $r\rightarrow r_+$. Just like in $\mathcal {F}_\textrm{low,1a}$, due to the smallness of |*a*| we can implement these ideas at the level k=|s| only, to that is to (6.13), and rely on transport estimates to control the coupling errors, thus concluding an easy adaptation of [54, Section 9.3].

#### The complete list of frequency regimes

**Fig. 3:**
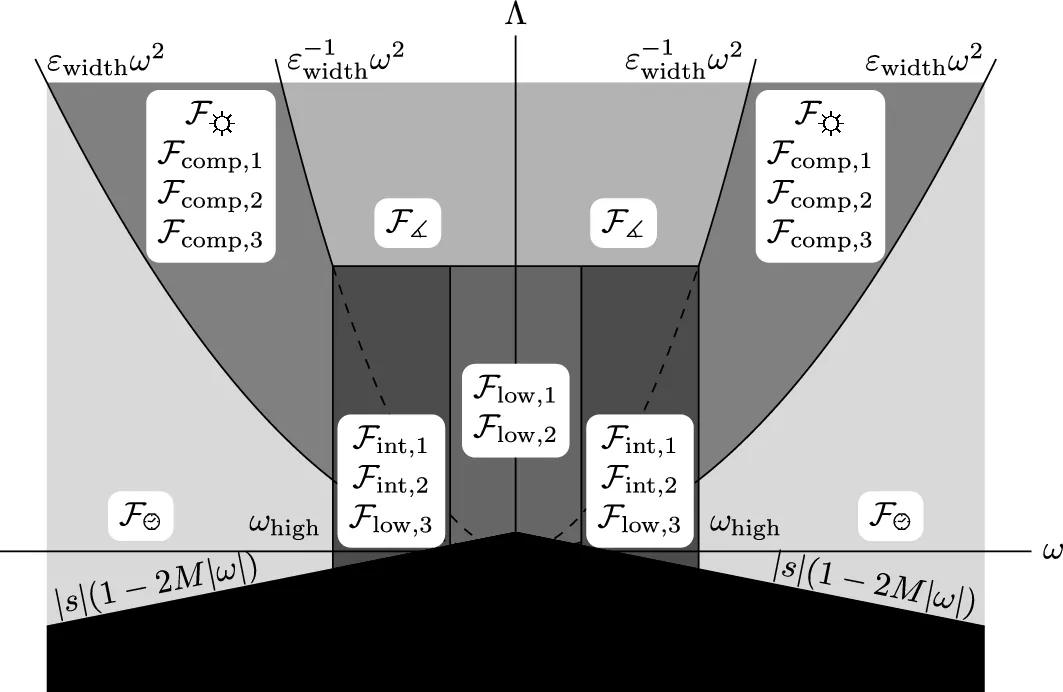
Frequency ranges in the proof of Theorems 6.3 and 6.4

For the convenience of the reader, in this section we collect the definitions of the various frequency regimes we will consider (making the dependence on *a* explicit), and explain why they form a partition of $\mathcal {F}_\textrm{admiss}$, see Fig. 3. Fix $s\in \mathbb {Z}$ and M>0. For some parameters $a_0,\tilde{a}_0,\tilde{a}_1\in (0,M)$, $\varepsilon _\textrm{width},\omega _\textrm{high}>0$, $r_0'\in [r_+,\infty )$ and $\beta _1,\beta _2,\beta _3\beta _4>0$, we define tha following

- High frequencies $$\mathcal {F}_\textrm{unbdd}:=\left\rbrace a\in [0,M],\,\, (\omega ,m,\Lambda ) \text { admissible}\right\lbrace \backslash \mathcal {F}_\textrm{bdd}$$
  - Time dominated frequencies:
  - Angular dominated frequencies:
    $$\begin{aligned} \mathcal {F}_{\measuredangle }:=\left\rbrace a\in [0,M],\,\,(\omega ,m,\Lambda ) \text { admissible}:\Lambda \geqq \varepsilon _\textrm{width}^{-1}\omega ^2,\,\, \Lambda \geqq \varepsilon _\textrm{width}^{-1}\omega _\textrm{high}^2\right\lbrace ,\end{aligned}$$
    comprising
    $$\begin{aligned} \mathcal {F}_{\measuredangle ,1}&:=\left\rbrace a\in [0,a_0]\,,(\omega ,m,\Lambda ) \text { admissible }:\Lambda \geqq \varepsilon _\textrm{width}^{-1}\omega ^2\,,\,\, \Lambda \geqq \varepsilon _\textrm{width}^{-1}\omega _\textrm{high}^2\,,\right.\\&\qquad \qquad \qquad \qquad \qquad \qquad \qquad \qquad \qquad \left. m\omega \leqq m^2\upomega _++\beta _1\Lambda \right\lbrace ,\\ \mathcal {F}_{\measuredangle ,2}&:=\left\rbrace a\in (a_0,M]\,,(\omega ,m,\Lambda ) \text { admissible }:\Lambda \geqq \varepsilon _\textrm{width}^{-1}\omega ^2\,,\,\, \Lambda \geqq \varepsilon _\textrm{width}^{-1}\omega _\textrm{high}^2\,,\right.\\&\qquad \qquad \qquad \qquad \qquad \qquad \qquad \qquad \qquad \left. m\omega \leqq m^2\upomega _+-\beta _2\Lambda \right\lbrace ,\\ \mathcal {F}_{\measuredangle ,3}&:=\left\rbrace a\in (a_0,M], \,(\omega ,m,\Lambda ) \text { admissible }:\Lambda \geqq \varepsilon _\textrm{width}^{-1}\omega ^2\,,\,\, \Lambda \geqq \varepsilon _\textrm{width}^{-1}\omega _\textrm{high}^2\,,\right.\\&\qquad \qquad \qquad \qquad \qquad \qquad \qquad \qquad \qquad \left. m\omega \geqq m^2\upomega _+-\beta _2\Lambda \right\lbrace \,; \end{aligned}$$
  - Comparable frequencies:
    $$\begin{aligned}  &   \mathcal {F}_\textrm{comp}:=\left\rbrace a\in [0,M],\,\,(\omega ,m,\Lambda ) \text { admissible}:\right. \\  &   \left.\quad \varepsilon _\textrm{width}\omega ^2\leqq \Lambda \leqq \varepsilon _\textrm{width}^{-1}\omega ^2,\,\, |\omega |\geqq \omega _\textrm{high}\right\lbrace , \end{aligned}$$
    comprising
    - non-trapped and non-superradiant
      $$\begin{aligned} \mathcal {F}_\textrm{comp,2}&:=\{a\in [0,M]\,,\,\, (\omega ,m,\Lambda ) \text { admissible}:\\&\quad \varepsilon _\textrm{width}\omega ^2 \leqq \Lambda \leqq \varepsilon _\textrm{width}^{-1}\omega ^2\,,\,\, |\omega |\geqq \omega _\textrm{high}\,,\,\, r_0'=\infty \,,\\&\quad \qquad m\omega \notin (0, m^2\upomega _++\beta _1\Lambda )\}, \end{aligned}$$
      with $\mathcal {F}_\textrm{comp,2a}=\mathcal {F}_\textrm{comp,2}\cap \left\rbrace \Lambda -2am\omega \geqq \beta _4\Lambda \right\lbrace$ and with $\mathcal {F}_\textrm{comp,2b}=\mathcal {F}_\textrm{comp,2}\backslash \mathcal {F}_\textrm{comp,2a}$,
    - non-trapped superradiant regime, , where we have
    - trapped non-superradiant regime
      $$\begin{aligned} \mathcal {F}_\textrm{comp,1}&:=\{a\in [0,M]\,,\,\,(\omega ,m,\Lambda ) \text { admissible}:\\&\varepsilon _\textrm{width}\omega ^2 \leqq \Lambda \leqq \varepsilon _\textrm{width}^{-1}\omega ^2\,,\,\, |\omega |\geqq \omega _\textrm{high}\,,\,\, r_0'<\infty \,,\\&\qquad m\omega \notin (0, m^2\upomega _++\beta _3\Lambda )\}, \end{aligned}$$
    - trapped and marginally superradiant regime
      $$\begin{aligned} \mathcal {F}_\textrm{comp,3}&:=\{a\in (a_0,M]\,,\,\,(\omega ,m,\Lambda ) \text { admissible}:\\&\varepsilon _\textrm{width}\omega ^2 \leqq \Lambda \leqq \varepsilon _\textrm{width}^{-1}\omega ^2\,,\,\, |\omega |\geqq \omega _\textrm{high}\,,\\&\qquad \qquad m^2\upomega _+-\beta _2\Lambda< m\omega <m^2\upomega _++\beta _3\Lambda ) \}. \end{aligned}$$
- Bounded frequencies
  $$\mathcal {F}_\textrm{bdd}:=\left\rbrace a\in [0,M], (\omega ,m,\Lambda ) \text { admissible }:\Lambda \leqq \varepsilon _\textrm{width}^{-1}\omega _\textrm{high}^2,\,\, |\omega |< \omega _\textrm{high}\right\lbrace $$
  - Bounded frequencies with small “Killing frequency”: $\mathcal {F}_\textrm{low}:=\mathcal {F}_\textrm{low,1}\cup \mathcal {F}_\textrm{low,2}\cup \mathcal {F}_\textrm{low,3}$, where we have
    - small *T* frequency under axisymmetry or slowly rotating background $\mathcal {F}_\textrm{low,1}=\mathcal {F}_\textrm{low,1a}\cup \mathcal {F}_\textrm{low,1b}\cup \mathcal {F}_\textrm{low,1c}$, with
      $$\begin{aligned} \mathcal {F}_\textrm{low,1a}&:=\left\rbrace a\in [0,\tilde{a}_0]\,,\,\, (\omega ,m,\Lambda ) \text { admissible }:\right.\\&\left.\Lambda \leqq \varepsilon _\textrm{width}^{-1}\omega _\textrm{high}^2\,,\,\, |\omega |\leqq \omega _\textrm{low}\right\lbrace ,\\ \mathcal {F}_\textrm{low,1b}&:=\left\rbrace a\in [0,M-\tilde{a}_1)\,,\,\, (\omega ,m,\Lambda ) \text { admissible }:\right.\\&\left.\Lambda \leqq \varepsilon _\textrm{width}^{-1}\omega _\textrm{high}^2\,,\,\, |\omega |\leqq \omega _\textrm{low}\,,\,\, m=0\right\lbrace ,\\ \mathcal {F}_\textrm{low,1c}&:=\left\rbrace a\in [M-\tilde{a}_1,M]\,,\,\, (\omega ,m,\Lambda ) \text { admissible }:\right.\\&\left.\Lambda \leqq \varepsilon _\textrm{width}^{-1}\omega _\textrm{high}^2\,,\,\, |\omega |\leqq \omega _\textrm{low}\,,\,\, m=0\right\lbrace , \end{aligned}$$
    - small *T* frequency outside axisymmetry for fast rotating background
      $$\begin{aligned} \mathcal {F}_\textrm{low,2}&:=\left\rbrace a\in ({\tilde{a}}_0,M],\,\,(\omega ,m,\Lambda ) \text { admissible }:\right.\\&\left.\Lambda \leqq \varepsilon _\textrm{width}^{-1}\omega _\textrm{high}^2,\,\, |\omega |\leqq \omega _\textrm{low},\,\, m\ne 0\right\lbrace , \end{aligned}$$
    - small *K* frequency and nearly extremal
      $$\begin{aligned} \mathcal {F}_\textrm{low,3}&:=\left\rbrace a\in [M-{\tilde{a}}_1,M]\,,\,\, (\omega ,m,\Lambda ) \text { admissible}:\right.\\&\quad \left.\Lambda \leqq \varepsilon _\textrm{width}^{-1}\omega _\textrm{high}^2\,,\,\, \omega _\textrm{low}<|\omega |< \omega _\textrm{high}\,,\,\,\right.\\&\qquad \qquad \left. |\omega -m\upomega _+|\leqq \tilde{\omega }_\textrm{low}\,,\,\, m\ne 0\right\lbrace . \end{aligned}$$
  - Intermediate bounded frequencies, where we have
    $$\begin{aligned} \mathcal {F}_\textrm{int,1}&:=\left\rbrace a\in [0,M-{\tilde{a}}_1)\,, (\omega ,m,\Lambda ) \text { admissible }:\right.\\&\left. \Lambda \leqq \varepsilon _\textrm{width}^{-1}\omega _\textrm{high}^2\,,\,\, \omega _\textrm{low}<|\omega |< \omega _\textrm{high}\right\lbrace ,\\ \mathcal {F}_\textrm{int,2}&:=\left\rbrace a\in [M-{\tilde{a}}_1,M]\,, (\omega ,m,\Lambda ) \text { admissible }\right.\\&\qquad :\Lambda \leqq \varepsilon _\textrm{width}^{-1}\omega _\textrm{high}^2\,,\,\, \omega _\textrm{low}<|\omega |< \omega _\textrm{high}\,,\\&\qquad \qquad \left. |\omega -m\upomega _+|> \tilde{\omega }_\textrm{low}\right\lbrace . \end{aligned}$$

##### Lemma 6.9

(Frequency partition) Fix M>0, $s\in \mathbb {Z}$, $a_0\in (0,M)$. If $\varepsilon _\textrm{width}$ is sufficiently small depending on $a_0$ and $\beta _1$, if $\beta _2$ is sufficiently small depending on $a_0$ and if $\beta _3\leqq \beta _1$, we haveand so $\cup _{0\leqq a\leqq M}\mathcal {F}_{\textrm{ admiss}(a,M,s)} \subset \mathcal {F}_\textrm{unbdd} \cup \mathcal {F}_\textrm{unbdd}$.

##### Proof

From the definitions above, we see that the only assertion which is not obvious is the statement that $\mathcal {F}_{\measuredangle }\subseteq \mathcal {F}_{\measuredangle ,1}\cup \mathcal {F}_{\measuredangle ,2}\cup \mathcal {F}_{\measuredangle ,3}$, which we focus on now.

Consider $(\omega ,m,\Lambda )\in \mathcal {F}_{\measuredangle }$, to that is assume $\Lambda \geqq \varepsilon _\textrm{width}^{-1}\omega ^2$ and $\Lambda \geqq \varepsilon _\textrm{width}^{-1}\omega _\textrm{high}^2$. For sufficiently large $\omega _\textrm{high}$ and $\varepsilon _\textrm{width}^{-1}$, the angular dominated conditions together with the admissibility criteria in Definition 4.1 imply that $\Lambda \geqq m^2$; see Lemma 6.23(i), below.

We first notice that

$$\begin{aligned} m\omega \geqq m^2\upomega _+ +\beta _1\Lambda \geqq \beta _1 \varepsilon ^{-1}_\textrm{width} \omega ^2&\Rightarrow |m|\geqq \beta _1 \varepsilon ^{-1}_\textrm{width} |\omega | \text { and } |\omega |\geqq \beta _1 \Lambda |m|^{-1} \geqq \beta _1 |m|\\&\Rightarrow |m|\geqq \beta _1^2\varepsilon _\textrm{width}^{-1}|m|\,, \end{aligned}$$

cannot hold if $\varepsilon _\textrm{width}$ is taken small enough that $\varepsilon _\textrm{width}< \beta _1^2$. □

### The high frequencies

In this section, we will establish Theorem 6.3 for frequency triples $(\omega ,m,\Lambda )$ where at least one entry is bounded from below by a large constant. We begin, in Section 6.3.1 by stating some important properties of the potential for the transformed radial ODE (4.21) in these regimes. Next, we establish an easy, but very important, elliptic estimate for the k<|s| transformed variables in Section 6.3.2. Finally, Sections 6.3.3, 6.3.4 and 6.3.5 contain the statement and proofs of the main results for the high frequency regimes.

#### Properties of the frequency-dependent potential

For large frequency parameters, the behavior of the potential will be driven by $\mathcal {V}_0$ given in (4.22). This potential is essentially very similar to that in the case s=0, hence most of our statements here are easy modifications of [55, 57], which we include for completeness. We begin by discussing the critical point structure $\mathcal {V}_0$:

##### Lemma 6.10

(Critical points of $\mathcal {V}_0$) Fix $s\in \mathbb {Z}$, M>0 and $a_0\in (0, M)$. Assume $0\leqq a\leqq M$. Then, for all admissible frequency triples $(\omega ,m,\Lambda )$ such that $\Lambda \geqq \frac{2}{3} m^2$ and $\Lambda +s^2>0$, we have:

(i) As a function of $r\in (r_+,\infty )$, the potential $\mathcal {V}_0$, is either (a) strictly decreasing, (b) has a unique critical value $r^0_\textrm{max}$ which is a global maximum or (c) has exactly two critical values $r^0_\textrm{min}<r^0_\textrm{max}$ which are a local minimum and maximum respectively.
(ii) If it exists, $r^0_\textrm{min}\in (r_+,\infty )$ satisfies $\mathcal {V}_0(r_\textrm{min}^0) <\omega ^2$.
(iii) it:trappingspsupperspsbound Suppose $r_\textrm{max}^0$ exists. If mω>0, then $r^0_\textrm{max}\leqq B$ independently of the frequency parameters. If $m\omega \leqq 0$ and there is a constant c>0 such that $\Lambda \geqq c|am\omega |$, then $r^0_\textrm{max}\leqq B(c)$.

##### Remark 6.11

*(On trapping I)* It is worth noting that, in addition to its role in (4.21), $\mathcal {V}_0$ is also the potential associated with geodesic flow, see [57, Section 6.4]. Thus, Lemma 6.10 yields information regarding geodesic trapping. For instance, by combining (i) and (ii), we deduce that trapping can occurs at the single maximum of $\mathcal {V}_0$, if it exists: thus, for each $(\omega ,m,\Lambda )$, trapping is associated with a single *r*-value and it is an unstable feature, both of these aspects replicating the situation for a=0. By (iii), trapping occurs at finite distance from the event horizon, and not in the asymptotically flat region (as expected from the absence of trapping in flat space).

##### Proof of Lemma 6.10

We begin by identifying the critical point structure of $\mathcal {V}_0$. Define $\sigma =am\omega /(\Lambda +s^2)$. It is easy to check that

6.14 $$\begin{aligned} (r^2+a^2)^3\frac{\textrm{d}\mathcal {V}_0}{\textrm{d}r}&=-2(\Lambda +s^2)\left[ r^3 -3Mr^2(1-2\sigma )\right.\nonumber \\&\quad \left.+a^2r\left(1-\frac{2m^2}{\Lambda +s^2}\right)+Ma^2(1-2\sigma )\right]\,, \end{aligned}$$

6.15 $$\begin{aligned} \frac{\textrm{d}}{\textrm{d}r}\left[(r^2+a^2)^3\frac{\textrm{d}\mathcal {V}_0}{\textrm{d}r}\right]&=-12(\Lambda +s^2) \left[\frac{1}{2}r^2-M(1-2\sigma ) r +\frac{1}{6}a^2\left(1-\frac{2m^2}{\Lambda +s^2}\right)\right]\,, \end{aligned}$$

and the latter has roots

$$\begin{aligned} r_{1,\,2}=M(1-2\sigma )\pm \sqrt{M^2(1-2\sigma )^2-\frac{a^2}{3}\left(1-\frac{2m^2}{\Lambda +s^2}\right)}\,, \end{aligned}$$

where $r_1$ corresponds to the + sign and $r_2$ corresponds to − sign, so that $\operatorname {Re}r_2\leqq \operatorname {Re}r_1$.

If $m\omega> 0 \implies \sigma > 0$, we have $\operatorname {Re}r_2<M\leqq r_+$. Now suppose that $m\omega \leqq 0$ and rewrite $r_2$ as

$$\begin{aligned} r_{2}=M(1+2|\sigma |)\left(1- \sqrt{1-x}\right)\,,\quad x=\frac{a^2}{3M^2(1+2|\sigma |)^2}\left(1-\frac{2m^2}{\Lambda +s^2}\right)\,. \end{aligned}$$

Assuming that $$\Lambda \geqq \frac{2}{3}m^2$$,

$$\begin{aligned} 0\leqq |x|&=\frac{a^2}{3M^2(1+2|\sigma |)^2}\left|1-\frac{2m^2}{\Lambda +s^2}\right|\\&\leqq \frac{a^2}{3M^2}\left|1-\frac{2m^2}{\Lambda +s^2}\right|\leqq \frac{1}{3}\left|1-\frac{2m^2}{\Lambda +s^2}\right|\leqq \frac{2}{3}\,, \end{aligned}$$

and, since $0\leqq |x| \leqq 2/3$, we have the inequality $\sqrt{1-x}\geqq 1-2|x|/3 \Rightarrow 1-\sqrt{1-x}\leqq 2|x|/3$, we obtain

$$\begin{aligned} \operatorname {Re}r_2< \frac{2M(1+2|\sigma |)a^2}{9M^2(1+2|\sigma |)^2}\left|1-\frac{2m^2}{\Lambda +s^2}\right| \leqq \frac{2M}{9}\left|1-\frac{2m^2}{\Lambda +s^2}\right|\leqq \frac{4}{9}M <M\leqq r_+\,. \end{aligned}$$

We can now conclude that there is at most one root in $[r_+,\infty )$ for $\frac{\textrm{d}}{{\textrm{d}}r}\left[(r^2+a^2)^3\frac{\textrm{d}}{{\textrm{d}}r}\mathcal {V}_0\right]$, at $r=r_1$.

By Rolle’s theorem, there are at most two zeros of $\frac{\textrm{d}}{{\textrm{d}}r}\mathcal {V}_0$. As the leading order term of (6.14) in *r* is negative, since $\Lambda +s^2> 0$, we conclude that $\frac{\textrm{d}}{{\textrm{d}}r}\mathcal {V}_0$ either vanishes nowhere, vanishes at a unique point $r^0_\textrm{max}$, or vanishes at points $r^0_{\min }<r^0_\textrm{max}$. Hence, in $[r_+,\infty )$, $\mathcal {V}_0$ is either (a) strictly decreasing, (b) has a unique critical value $r^0_\textrm{max}$ which is a global maximum or (c) has exactly two critical values $r^0_\textrm{min}<r^0_\textrm{max}$ which are a local minimum and maximum respectively. This concludes the proof of statement (i).

We now move on to proving some general points about the minimum and maximum of $\mathcal {V}_0$, if they exist. For statement (ii), note that

6.16 $$\begin{aligned} \omega ^2-\mathcal {V}_0(r_+)=(\omega -m\upomega _+)^2\geqq 0\,, \end{aligned}$$

so $\mathcal {V}_0(r_\textrm{min}^0)< \omega ^2$ if $r_\textrm{min}^0\in (r_+,\infty )$ exists. For statement (iii), we observe that, as $r\rightarrow \infty$

$$\begin{aligned} \frac{(r^2+a^2)^3}{2\Lambda }\frac{\textrm{d}\mathcal {V}_0}{\textrm{d}r}(r)&=-r^3+3M\left(1-\frac{2am\omega }{\Lambda }\right)r^2\\&\quad +\left[O\left(\frac{am\omega }{\Lambda }\right)+O\left(\frac{m^2}{\Lambda }\right)\right]O(r)\,. \end{aligned}$$

Thus, $r^0_{\max }$ must bounded from above if $m\omega \geqq 0$ (in this case, independently of the frequency parameters) or if $\Lambda +s^2\geqq c |am\omega |$ (in this case, depending on *c*). This shows statement (iii). □

We warm-up the more in-depth analysis by looking at the easy case when *m* is arbitrarily small compared to the frequency parameter Λ:

##### Lemma 6.12

($\mathcal {V}_0$ for fixed *m*) Fix $s\in \mathbb {Z}$, M>0 and $a_0\in [0,M)$. There is a sufficiently small c>0 such that for $\Lambda \geqq c^{-1}$, $\Lambda \geqq c^{-1}|am\omega |$ and $m^2\leqq c\Lambda$, the potential $\mathcal {V}_0$ has a maximum at $r=r_\textrm{max}^0$, where

$$\begin{aligned} b\leqq r_\textrm{max}^0-(1+\tfrac{3}{4}\sqrt{2})M\leqq B, \end{aligned}$$

and may additionally have a minimum at $r=r_\textrm{min}^0$, where then $0\leqq r_\textrm{min}-r_+\leqq Bc$.

If we moreover have $\omega ^2\leqq c^2\Lambda$, then

$$\begin{aligned} \mathcal {V}_0(r_\textrm{max}^0)-\omega ^2\geqq b\Lambda ,\qquad |\mathcal {V}_0(r_\textrm{min}^0)|\leqq B c \Lambda . \end{aligned}$$

##### Remark 6.13

*(On high frequency fixed m)*

Continuing from Remark 6.11, we note that Lemma 6.12 implies that for fixed *m* but large Λ (and possibly ω), as $1+\frac{3}{4}\sqrt{2}>2.06>2$, trapping can only occur outside the ergorregion. One can check that $r=(1+\sqrt{2})M$ corresponds to the location of trapping in extremal Kerr, |a|=M, under axisymmetry, see also [19].

##### Proof of Lemma 6.12

We start by estimating $r_\textrm{max}^0$. Since $|am\omega |\leqq c\Lambda$ and $m^2\leqq c\Lambda$ for sufficiently small *c*, we obtain

$$\begin{aligned} \frac{1}{\Lambda }(r^2+a^2)^3\frac{\textrm{d}\mathcal {V}_0}{\textrm{d}r}\Big |_{r=(1+\tfrac{3}{4}\sqrt{2})M}&= O(c) -2\left[r^3-3Mr^2+a^2(r+M)\right]\big |_{r=(1+\tfrac{3}{4}\sqrt{2})M}\\&= O(c)+\frac{8+3\sqrt{2}}{2}M(M^2-a^2)+\frac{21M^3}{8\sqrt{2}}\,, \end{aligned}$$

as c→0. Since $\mathcal {V}_0'<0$ for sufficiently large *r*, we deduce that $r_\textrm{max}^0$ exists and lies quantitatively above $(1+\tfrac{3}{4}\sqrt{2})M>2M$; Lemma 6.10(iii) yields an upper bound.

We now turn to the last statement. As we have no information on the sign of amω, we might have either sign for $\frac{\textrm{d}}{\textrm{d}r}\mathcal {V}_0(r_+)$. Thus, by Lemma 6.10, $\mathcal {V}_0$ may have a minimum at $r=r_\textrm{min}^0$ satisfying $r_\textrm{min}^0<r_\textrm{max}^0\leqq B$. We can obtain a sharper upper bound: since

$$\begin{aligned}&\frac{\textrm{d}}{\textrm{d}r}\mathcal {V}_0 =-\frac{\textrm{d}}{\textrm{d}r}(\omega ^2-\mathcal {V}_0)= -\frac{dw}{\textrm{d}r}(\Lambda +s^2-2am\omega )\\&\quad -\frac{4am}{r^2+a^2}\left(\omega -\frac{am}{r^2+a^2}\right) \geqq \left(-r^3\frac{dw}{\textrm{d}r}-bc\right)\frac{\Lambda +s^2}{r^3}, \end{aligned}$$

we deduce that $r_\textrm{min}^0-r_+ \leqq B c$ if it exists.

If one additionally has $\omega ^2\leqq c^2\Lambda$, then estimates for $\mathcal {V}_0(r_\textrm{max}^0)\geqq \mathcal {V}_0((1+\tfrac{3}{4}\sqrt{2})M)$ and $\mathcal {V}_0(r_\textrm{min}^0)$ follow. □

Next, we look at the critical point structure in the case of (sufficiently) superradiant frequencies and where $m\omega \leqq 0$:

##### Lemma 6.14

($\mathcal {V}_0$ at superradiance and below) Fix $s\in \mathbb {Z}$, M>0 and $a_0\in (0,M)$. Let $\tilde{\epsilon }>0$ satisfy $\tilde{\epsilon }\leqq M^2-a_0^2$, $\beta _1=\beta _1(a_0)$ be such that $a_0\beta _1<(M^2-a_0^2)/(12M^2)$, and $\beta _2=\beta _2(a_0,\tilde{\epsilon })$ be such that $0<\beta _2<\frac{a_0}{2\,M^2}$ and $\tilde{\epsilon }< M^3 a_0\beta _2$. If $(\omega ,m,\Lambda )$ be an admissible frequency triple satisfying one of the following three requirements

$$\begin{aligned} (i)&~ 0<m\omega \leqq m^2\upomega _+ +\beta _1\Lambda \,,\,\, 0\leqq a\leqq a_0\,,\,\,\Lambda \geqq \max \left\rbrace \frac{a_0^2}{M^2},\frac{2}{3}\right\lbrace m^2\,,\\ (ii)&~0<m\omega \leqq m^2\upomega _+ -\beta _2\Lambda \,,\,\, a_0< a\leqq M\,, \,\,\Lambda \geqq m^2\,,\\ (iii)&~m\omega \leqq 0\,, \,\,\Lambda \geqq \max \left\rbrace \frac{a_0^2}{M^2},\frac{2}{3}\right\lbrace m^2\,,\,\,\Lambda \geqq \tilde{\epsilon }^{-1}\omega ^2\,, \end{aligned}$$

and $\tilde{\epsilon }>0$ is sufficiently small, then the following hold.

1. (Derivatives of $\mathcal {V}_0$ at $r_+$.) In cases (i)–(ii) we have
  $$\begin{aligned} \frac{\textrm{d}}{\textrm{d}r} \mathcal {V}_0(r_+)\geqq b\Lambda \geqq 0\,, \end{aligned}$$
  where *b* is a possibly small constant which, in addition to *M* and *s*, depends on $a_0$ in case (i) and on $a_0\beta _2$ in case (ii). In case (iii), we have
  $$\begin{aligned} \frac{\textrm{d}}{\textrm{d}r}\left((r^2+a^2) \frac{\textrm{d}}{\textrm{d}r}\mathcal {V}_0\right)(r_+)\geqq b a^2\Lambda \geqq 0\,,\qquad \frac{\textrm{d}}{\textrm{d}r} \mathcal {V}_0(r)\geqq b(r-M)\Lambda \geqq 0\,, \end{aligned}$$
  for *r* sufficiently close to $r_+$, independently of $(a_0,\beta _1,\beta _2)$. Thus, the potential $\mathcal {V}_0$ has a unique critical point, which is a maximum, located at $r=r_\textrm{max}^0$.
2. (Sign of $\mathcal {V}_0-\omega ^2$.) In cases (i)–(ii), we have the estimate
  6.17 $$\begin{aligned} \mathcal {V}_0(r^0_\textrm{max})-\omega ^2\geqq b \Lambda \,, \end{aligned}$$
  where $b=b(a_0)$ in case (i), $b=b(a_0\beta _2)$ in case (ii); in case (iii) the same estimate holds with *b* independent of $(a_0,\beta _1,\beta _2)$. If additionally we have that $|am\omega |\leqq B\Lambda$, then $r_\textrm{max}^0$ satisfies
  $$\begin{aligned} |r_\textrm{max}^0|\leqq B\,,\quad |r_\textrm{max}^0-r_+|\geqq b\,, \end{aligned}$$
  where *b* depends on $a_0$ and $\beta _1$ in case (i) and on $a_0\beta _2$ in case (ii). Furthermore, we can find δ, with the same dependencies, such that
  $$\begin{aligned} \mathcal {V}_0(r)-\omega ^2\geqq b \Lambda \qquad \forall \,r\in [r_\textrm{max}^0-\delta ,r_\textrm{max}^0+\delta ]\,. \end{aligned}$$
3. (Sign of $\mathcal {V}_0'$.) Assume that $|am\omega |\leqq B\Lambda$. Then, in (i)–(ii) we have
  $$\begin{aligned} -(r-r_\textrm{max}^0)\frac{\textrm{d}\mathcal {V}_0}{\textrm{d}r} \geqq b\Lambda \frac{(r-r_\textrm{max}^0)^2}{r^4}&\qquad \forall \, r\in [r_+,\infty )\,, \end{aligned}$$
  where *b* is a possibly small constant which, in addition to *M* and *s*, depends on $a_0$ in case (i) and on $a_0\beta _2$ in case (ii). In case (iii), we have
  $$\begin{aligned} -(r-r_\textrm{max}^0)\frac{\textrm{d}\mathcal {V}_0}{\textrm{d}r} \geqq b\Lambda \frac{(r-M)(r-r_\textrm{max}^0)^2}{r^5}&\qquad \forall \, r\in [r_+,\infty )\,. \end{aligned}$$

##### Remark 6.15

*(On high frequency superradiance)* Lemma 6.14 characterizes $\mathcal {V}_0$ for most (for extremal or nearly extremal Kerr) or all (for subextremal Kerr) superradiant frequencies. By statement (1), combined with Lemma 6.10, we conclude that $\mathcal {V}_0$ has a unique critical point, located at $r^0_\textrm{max}$, which is a maximum. By statement (2), the maximum of $\mathcal {V}_0$ is always quantitatively above the energy level of trapped geodesics in the subextremal case $0\leqq a \leqq a_0$. Thus, for subextremal Kerr, superradiant frequencies are quantitatively non-trapped. This property degenerates as a→M: for $a_0<a\leqq M$, from Lemma 6.14 we see that it only holds if the frequencies are quantitatively away from the superradiant threshold $\omega =m\upomega _+$.

##### Proof of Lemma 6.14

We will prove each statement separately.

*Statement * (1). At $$r=r_+$$,

$$\begin{aligned}&(r_+^2+a^2)^3\frac{\textrm{d}\mathcal {V}_0}{\textrm{d}r}(r_+)\\&= -4Mam(\omega -m\upomega _+)(3r_+^2-a^2)+4(r_+-M)[(\Lambda +s^2)Mr_+-a^2m^2]\,,\\&\quad \frac{\textrm{d}}{\textrm{d}r}\left((r^2+a^2)^3\frac{\textrm{d}\mathcal {V}_0}{\textrm{d}r}\right)(r_+) =4\left( a^2(\Lambda +s^2+m^2) -6am\omega Mr_+\right)\,. \end{aligned}$$

Clearly, if $m\omega \leqq 0$ and $a\in [0,a_0]$, then

$$\begin{aligned} \frac{\textrm{d}\mathcal {V}_0}{\textrm{d}r}(r_+)&\geqq \frac{(r_+-M)}{2M^2r_+^2}\Lambda \,. \end{aligned}$$

If $m\omega \leqq 0$ and $a\in (a_0,M]$, then either $m^2\leqq \frac{1}{4} \Lambda$, in which case $\Lambda -2\,m^2\geqq \frac{1}{2}\Lambda$, or $m^2\geqq \frac{1}{4}\Lambda$, in which case $64\,M^2\,m^2\upomega _+^2\leqq \Lambda$; in both instances, $\frac{\textrm{d}\mathcal {V}_0}{\textrm{d}r}$ is positive and scales, at worst, like Λ(r-M) in the limit $r\rightarrow r_+$. Thus, the conclusion holds in case (iii).

Now we turn to cases (i) and (ii), starting with the former. Assuming $a\in [0,a_0]$ and using the bound $\Lambda \geqq \max \left\rbrace \frac{2}{3},\frac{a_0^2}{M^2}\right\lbrace m^2$, we find

$$\begin{aligned} (r_+^2+a^2)^3\frac{\textrm{d}}{\textrm{d}r}\mathcal {V}_0(r_+)&\geqq 4\Lambda \left[-6M^2a_0\beta _1+(M^2-a_0^2)\right]\geqq 2(M^2-a_0^2)\Lambda \,, \end{aligned}$$

by the assumption on $\beta _1$. On the other hand, if $a\in [a_0,M]$, using the bound $\Lambda \geqq m^2$, we have

$$\begin{aligned} (r_+^2+a^2)^3\frac{\textrm{d}}{\textrm{d}r}\mathcal {V}_0(r_+)&\geqq 8M^3a_0\beta _2\Lambda \,. \end{aligned}$$

*Statement *(2). By Lemma 6.10 and statement (1), we deduce that $\mathcal {V}_0$ has a maximum at $r=r_\textrm{max}^0$ and no other critical points. We will now prove (6.17).

Let ϵ>0 be a sufficiently small constant to be fixed, and assume $\tilde{\epsilon }$ is even smaller. Then, the frequency ranges (i), (ii) and (iii) are are contained in one of the five ranges

$$\begin{aligned} (a)&~\omega ^2\leqq \epsilon \Lambda \,,\,\, \Lambda \geqq \max \left\rbrace \frac{2}{3},\frac{a_0^2}{M^2}\right\lbrace m^2\,,\\ (b)&~m^2\upomega _+-\epsilon |m|\sqrt{\Lambda }\leqq m\omega \leqq m^2\upomega _++\beta _1\Lambda \,,\,\, \omega ^2>\epsilon \Lambda \,,\,\, 0\leqq a \leqq a_0\,, \\ (c)&~m^2\upomega _+-\epsilon |m|\sqrt{\Lambda }\leqq m\omega \leqq m^2\upomega _+-\beta _2\Lambda \,,\,\, \omega ^2>\epsilon \Lambda \,,\,\, a>a_0\,, \\ (d)&~0<m\omega<m^2\upomega _+-\epsilon |m|\sqrt{\Lambda }\,,\,\, \omega ^2>\epsilon \Lambda \,,\,\,0\leqq a\leqq a_0\,,\,\,\\&\qquad \Lambda \geqq \max \left\rbrace \frac{2}{3},\frac{a_0^2}{M^2}\right\lbrace m^2\,, \\ (e)&~0<m\omega <m^2\upomega _+-\epsilon |m|\sqrt{\Lambda }\,,\,\, \omega ^2>\epsilon \Lambda \,,\,\,a>a_0\,,\,\, \Lambda \geqq m^2\,. \end{aligned}$$

In each of these five ranges, we will find some *r*-value for which the potential $\mathcal {V}_0$ is quantitatively above $\omega ^2$, implying that so is the maximum of $\mathcal {V}_0$.

In range (a), if we have the bound $\Lambda \geqq \frac{2}{3} m^2$, then $2am\omega \geqq -M\sqrt{6\epsilon }\Lambda$, $-a^2\,m^2 \geqq -2\,M^2 \Lambda$, hence

$$\begin{aligned} \mathcal {V}_0(r)-\omega ^2\geqq - \epsilon \Lambda + \frac{\Lambda +s^2}{r^2}+O\left(\frac{\Lambda }{r^3}\right) \end{aligned}$$

as $r\rightarrow \infty$. Thus, for $\tilde{r}$ sufficiently large and ϵ sufficiently small depending on $\tilde{r}$, we have $\mathcal {V}_0(\tilde{r})-\omega ^2 \geqq b(\epsilon )\Lambda$.

In ranges (b) and (c), $|m\upomega _+-\omega |\leqq \max \{\epsilon \sqrt{\Lambda },|\omega |\}$, hence

$$\begin{aligned} \omega ^2-\mathcal {V}_0(r_+)=(\omega -m\upomega _+)^2\leqq \epsilon ^2\Lambda \,. \end{aligned}$$

Combining with statement (1), we have, for sufficiently small δ>0 and smaller ϵ,

$$\begin{aligned} \mathcal {V}_0(r_++\delta )-\omega ^2 =-\epsilon ^2\Lambda +\frac{\textrm{d}\mathcal {V}_0}{\textrm{d}r}(r_+)\delta +O(\delta ^2)\geqq b\Lambda \,, \end{aligned}$$

for $b=b(a_0)$ in case (i) and $b=b(a_0\beta _2)$ in case (ii). We can now fix ϵ.

Finally, in the last two frequency ranges, (d) and (e), define

$$\begin{aligned} r_0:=\frac{m \upomega _+}{\omega }r_+ \in \left(\frac{\upomega _+}{\upomega _+-\epsilon }r_+,\frac{\upomega _{+}}{\sqrt{\epsilon }}r_+\right)\,, \end{aligned}$$

where the bounds are computed using the definition of the frequency range. Letting $\Delta _{r_0}=r_0^2-2Mr_0+a^2$, we compute

$$\begin{aligned} \frac{(r_0^2+a^2)^2}{\Delta _{r_0}}\left(\mathcal {V}_0(r_0)-\omega ^2\right)&=\left[\Lambda +s^2-\frac{a^2}{4M^2}\left(1+\frac{2M}{r_0}+\frac{a^2}{r_0^2}\right)m^2\right]\\&\geqq \left[\Lambda -\frac{a^2}{M^2}(1+M^2\epsilon ^2-M\epsilon )m^2\right]\,. \end{aligned}$$

Using $\Lambda \geqq a_0^2m^2/M^2$ if $a\in [0,a_0]$ and $\Lambda \geqq m^2$ if $a\in (a_0,M]$, we obtain

$$\begin{aligned} \mathcal {V}_0(r_0)-\omega ^2 \geqq \frac{\Delta _{r_0}}{(r_0^2+a^2)^2} M\epsilon (1-M\epsilon )\Lambda \geqq b(\epsilon )\Lambda \,; \end{aligned}$$

this concludes the proof of (6.17).

Next, we assume that $\sigma :=am\omega /(\Lambda +s^2)$ is bounded independently of the frequencies. In this case,

6.18 $$\begin{aligned} \left|\frac{\textrm{d}\mathcal {V}_0}{\textrm{d}r}\right|+r\left|\frac{\textrm{d}^2\mathcal {V}_0}{\textrm{d}r^2}\right| \leqq \frac{B\Lambda }{r^3}\,, \qquad \left|\frac{\textrm{d}}{\textrm{d}r}\left((r^2+a^2)^3\frac{\textrm{d}\mathcal {V}_0}{\textrm{d}r}\right)\right|\leqq B\Lambda r^2\,. \end{aligned}$$

As we have seen in Lemma 6.10(2), this implies that $r_\textrm{max}^0\leqq B$ independently of the frequency parameters. Combining with statement (1), it also implies that $r^0_\textrm{max}-r_+\geqq b$ with *b* depending only on the same parameters as cited in statement (1). Finally, combining with (6.17), by continuity we can conclude the proof of statement (2).

*Statement (3).* To obtain the desired global estimates on $\mathcal {V}_0'$, we want to strengthen the above estimates for derivatives of $\mathcal {V}_0'$. Concretely, we will show that there exists an $r_1'\in (r_+,r_\textrm{max}^0)$ such that

6.19 $$\begin{aligned} \begin{aligned} \frac{\textrm{d}\mathcal {V}_0}{\textrm{d}r}(r) \geqq \hat{c}\frac{(r_+^2+a^2)^3}{(r_1^2+a^2)^3} \Lambda \,, \quad \forall \, r\in [r_+,r_1']\,, \\ \frac{\textrm{d}}{\textrm{d}r}\left((r^2+a^2)^3\frac{\textrm{d}\mathcal {V}_0}{\textrm{d}r}\right)\leqq -\tilde{c}\Lambda r^2\,, \quad \forall \, r\in [r_1',\infty )\,, \end{aligned} \end{aligned}$$

for some $\hat{c}>0$ possibly depending on $a_0,\beta _1,\beta _2$ as in the statement and $\tilde{c}$ independent of the frequency parameters. Recall that, in Lemma 6.10, it was seen that $\frac{\textrm{d}}{\textrm{d}r}\left((r^2+a^2)^3\frac{\textrm{d}\mathcal {V}_0}{\textrm{d}r}\right)$ is either non-positive on $[r_+,\infty )$ or there exists a unique point $r_1\in [r_+,r_\textrm{max})$ at which it switches from positive to negative. We have two cases:

- $r_1$ *exists.* In this case, $(r^2+a^2)^3\frac{\textrm{d}\mathcal {V}_0}{\textrm{d}r}$ is increasing up to $r_1$, hence
  $$\begin{aligned} \frac{\textrm{d}\mathcal {V}_0}{\textrm{d}r}(r) \geqq \frac{(r_+^2+a^2)^3}{(r_1^2+a^2)^3} \frac{\textrm{d}\mathcal {V}_0}{\textrm{d}r}(r_+) \geqq \frac{\hat{c}}{2}\frac{(r_+^2+a^3)^3}{(r_1^2+a^2)^3} \Lambda \,, \quad \forall \, r\in [r_+,r_1]\,, \end{aligned}$$
  where the final bound comes from 6.14(1) so $\hat{c}>0$ inherits the dependence on other parameters from it. Thus, by the bound on $\frac{\textrm{d}}{\textrm{d}r}\left((r^2+a^2)^3\frac{\textrm{d}\mathcal {V}_0}{\textrm{d}r}\right)$ from before and the fact that this is negative in $(r_1,\infty )$ by definition of $r_1$, there must be some $r_1'\in (r_1,r_\textrm{max}^0)$ such that (6.19) holds.
- $r_1$ *does not exist.* In this case, the second line of (6.19) holds for all *r* by Lemma 6.10 and the bounds (6.18). Moreover, $(r^2+a^2)^3\frac{\textrm{d}\mathcal {V}_0}{\textrm{d}r}$ is decreasing until it attains the value 0 at $r^0_\textrm{max}$, so there must be $r_1'$ such that the first line of (6.19) holds.

From (6.19), we can now conclude. □

Finally, we look at the critical point structure in the case of nonsuperradiant frequencies where $\omega ^2\sim \Lambda$:

##### Lemma 6.16

(Trapped frequencies) Fix M>0 and suppose a∈[0,M]. For any $\beta _3>0$, all sufficiently small $\varepsilon _\textrm{width}$ depending on $\beta _3$ that $\beta _3\geqq \sqrt{\varepsilon _\textrm{width}}$, all sufficiently large $\omega _\textrm{high}$ depending on $\varepsilon _\textrm{width}$, and all $(\omega ,m,\Lambda )$ admissible satisfying $\Lambda \geqq \frac{2}{3} m^2$ and

$$\begin{aligned} \varepsilon _\textrm{width}\omega ^2\leqq \Lambda \leqq \varepsilon _\textrm{width}^{-1}\omega ^2\,, \quad |\omega |\geqq \omega _\textrm{high}\,, \quad m\omega \notin (0, m^2\upomega _++\beta _3\Lambda ]\,, \end{aligned}$$

there is a $$c=c(\varepsilon _\textrm{width})$$ such that $$\omega ^2-\mathcal {V}_0(r_+)\geqq c\Lambda $$. Thus, for some $r^{\prime }$ depending on the frequency triple but satisfying $$|r'-r_+|\geqq b(\varepsilon _\textrm{width})$$, one has

6.20 $$\begin{aligned} \mathcal {V}_0-\omega ^2\leqq -\frac{1}{4} c\Lambda \,, \quad \forall \,r\in [r_+,r_0']\,. \end{aligned}$$

Let $$r_0'\in (r_+,\infty ]$$ be the supremum over such $r^{\prime }$. If $$r_0'<\infty $$, it satisfies $$r_0'\leqq B(\varepsilon _\textrm{width})$$, and the potential has a unique maximum $$r_\textrm{max}^0\in [r_0',\infty )$$ which satisfies $$|r_\textrm{max}^0-r_+|\leqq b(\varepsilon _\textrm{width})$$ and $$r_\textrm{max}^0\leqq B(\varepsilon _\textrm{width})$$.

##### Remark 6.17

*(On trapping II)*

In Lemma 6.16, we find that for quantitatively nonsuperradiant frequencies where $\omega ^2\sim \Lambda$, $\mathcal {V}_0$ is always below the energy level $\omega ^2$ close to $r=r_+$. If $r_0'=\infty$, this is the case for every *r*; hence only frequency triples such that $r_0'(\omega ,m,\Lambda )<\infty$ can be trapped.

##### Proof of Lemma 6.16

As $\sigma :=2am\omega /\Lambda \leqq 3M\varepsilon _\textrm{width}^{1/2}\leqq 1$ for sufficiently small $\varepsilon _\textrm{width}$, we can obtain the uniform bounds

6.21 $$\begin{aligned} \left|\frac{\textrm{d}\mathcal {V}_0}{\textrm{d}r}\right|+r\left|\frac{\textrm{d}^2\mathcal {V}_0}{\textrm{d}r^2}\right| \leqq B(\varepsilon _\textrm{width})\frac{\Lambda }{r^3}\,, \qquad \left|\frac{\textrm{d}}{\textrm{d}r}\left((r^2+a^2)^3\frac{\textrm{d}\mathcal {V}_0}{\textrm{d}r}\right)\right|\leqq B(\varepsilon _\textrm{width})\Lambda r^2\,. \end{aligned}$$

Moreover, we have

$$\begin{aligned} \omega ^2-\mathcal {V}(r_+)=(\omega -m\upomega _+)^2\geqq \min \left\rbrace \left(\sqrt{\varepsilon _\textrm{width}}+\frac{\sqrt{3/2}}{2M}\right)^2, \frac{2}{3} \beta _3^2\right\lbrace \Lambda \geqq \frac{2}{3}\varepsilon _\textrm{width}\Lambda \,, \end{aligned}$$

and hence we can fix $c(\varepsilon _\textrm{width})$ to be simply $\varepsilon _\textrm{width}$.

Let $r_0\in (r_+,\infty ]$ be the largest value such that

$$\begin{aligned} \mathcal {V}_0(r)\leqq \mathcal {V}_0(r_+)+\frac{c}{2}\Lambda \,, \qquad \forall r\in [r_+,r_0]\,. \end{aligned}$$

Clearly $r_0-r_+\geqq b(\varepsilon _\textrm{width})$. If $r_0=\infty$, then the potential is always below $\mathcal {V}_0(r_+)+\frac{c}{2}\Lambda$, hence any $r_0'>r_+$ satisfies (6.20) for b≦c/2; thus we can take $r_0'=\infty$.

We now turn to the case $r_0<\infty$. As $\frac{\textrm{d}\mathcal {V}_0}{\textrm{d}r}(r_0)\geqq 0$, by Lemma 6.10, $\mathcal {V}_0$ has a maximum at $r_\textrm{max}^0\geqq r_0$ and can also have a minimum at $r_\textrm{min}^0$ satisfying $r_\textrm{min}^0<r_0\leqq r_\textrm{max}^0<B(\varepsilon _\textrm{width})$. We can consider the following two cases:

- If $\mathcal {V}_0(r_\textrm{max}^0)\leqq \mathcal {V}_0(r_+)+\frac{3c}{4}\Lambda$, the inequality holds in $[r_+,\infty )$, so the bound (6.20) holds for any $r'\geqq r_0$. Thus, $r_0'=\infty$.
- If $\mathcal {V}_0(r_\textrm{max}^0)>\mathcal {V}_0(r_+)+\frac{3c}{4}\Lambda$, from the bound for the first derivative in (6.21), one can show that $r_\textrm{max}^0-r_0\geqq b(\varepsilon _\textrm{width})$. Since $\frac{\textrm{d}\mathcal {V}_0}{\textrm{d}r}(r_0)\geqq 0$, by continuity, there is a $r'\in [r_0,r_\textrm{max}^0]$ such that
  $$\begin{aligned} \mathcal {V}_0(r) \leqq \mathcal {V}_0(r_+)+\frac{3\Lambda }{4}\leqq \omega ^2 -\frac{1}{4}c\Lambda \,, \quad \forall r\in [r_+,r']\,. \end{aligned}$$
  Thus, as any such $r^{\prime }$ lies below $$r_\textrm{max}^0\leqq B(\varepsilon _\textrm{width})$$, so does $$r_0'$$.

This concludes the proof. □

#### An estimate for the k<|s| transformed variables

In this section we introduce two estimates which relates bulk terms in $\uppsi _{(k)}$ to bulk terms in $\uppsi _{(k+1)}$. The first makes use of the radial ODE for k<|s| or, more precisely, the constraint equation (4.23).

##### Lemma 6.18

(An elliptic estimate) Fix $s\in \mathbb {Z}\backslash \{0\}$, and k=0,⋯,|s|-1. Let $(\omega ,m,\Lambda )$ be an admissible frequency triple such that $\Lambda \geqq \Lambda _\textrm{high}>0$. The following hold.

1. Assume that there are $\varepsilon _1,\varepsilon _3\in (0,1)$ such that
  6.22 $$\begin{aligned} \Lambda -2am\omega -\varepsilon _1\left(\omega ^2+\frac{a^2}{Mr_+}m^2\right)-1\geqq \varepsilon _3\Lambda \,, \quad (\varepsilon _1\varepsilon _{3}^2 \Lambda _\textrm{high}^2)^{-1}\ll 1\,; \end{aligned}$$
  then we have
  6.23 $$\begin{aligned} \int _{-\infty }^\infty \varepsilon _3 w\Lambda \left|\uppsi _{(k)}\right|^2\textrm{d}r^*&\leqq \int _{-\infty }^\infty Bw\varepsilon _1^{-1}\left|\uppsi _{(k+1)}\right|^2\textrm{d}r^*\nonumber \\&\quad +B\int _{-\infty }^\infty \sum _{j=0}^{k}|\operatorname {Re}\left[\mathfrak {G}_{(j)}\overline{\uppsi _{(j)}}\right]|\textrm{d}r^*\,. \end{aligned}$$
  6.24 $$\begin{aligned} \int _{-\infty }^\infty \varepsilon _3w\Lambda \left|\uppsi _{(k)}'\right|^2\textrm{d}r^*&\leqq \int _{-\infty }^\infty \frac{Bw\Lambda }{\varepsilon _1\Lambda _\textrm{high}} \left(\left|\uppsi _{(k+1)}'\right|^2+\left|\uppsi _{(k+1)}\right|^2\right)\textrm{d}r^* \nonumber \\&\quad +B\int _{-\infty }^\infty k\sum _{j=0}^{k}\left(|\operatorname {Re}\left[\mathfrak {G}_{(j)}\overline{\uppsi _{(j)}}\right]|\right.\nonumber \\&\quad \left.+|\operatorname {Re}\left[\mathfrak {G}_{(j)}'\overline{\uppsi _{(j)}}'\right]|\right)\textrm{d}r^*\,. \end{aligned}$$
2. Assume that there are $\varepsilon _1, \varepsilon _2,\varepsilon _3\in (0,1)$ such that
  6.25 $$\begin{aligned}&\Lambda -2am\omega -\varepsilon _1\frac{a^2}{Mr_+}m^2-1-\varepsilon _2\Lambda _\textrm{high}\geqq \varepsilon _3\Lambda \,,\quad (\varepsilon _1\varepsilon _{3}^2 \Lambda _\textrm{high}^2)^{-1}\ll 1\,,\nonumber \\&\quad \frac{\omega ^2}{\Lambda }\ll \varepsilon _2 \varepsilon _3\Lambda _\textrm{high}^2\,; \end{aligned}$$
  then we have
  6.26 $$\begin{aligned}&\int _{-\infty }^\infty \varepsilon _3 w\Lambda \left|\uppsi _{(k)}\right|^2\textrm{d}r^*\nonumber \\&\quad \leqq \int _{-\infty }^\infty Bw\left(\frac{\omega ^2}{\varepsilon _2\Lambda _\textrm{high}}+\frac{\Lambda }{\Lambda _\textrm{high} \varepsilon _1 r}\right)\left|\uppsi _{(k+1)}\right|^2\textrm{d}r^*\nonumber \\&\qquad +B\int _{-\infty }^\infty \sum _{j=0}^{k}|\operatorname {Re}\left[\mathfrak {G}_{(j)}\overline{\uppsi _{(j)}}\right]|\textrm{d}r^*\,. \end{aligned}$$
  6.27 $$\begin{aligned}&\int _{-\infty }^\infty \varepsilon _3 w\Lambda \left|\uppsi _{(k)}'\right|^2\textrm{d}r^*\nonumber \\&\quad \leqq \int _{-\infty }^\infty Bw\left(\frac{\omega ^2}{\varepsilon _2\Lambda _\textrm{high}}+\frac{\Lambda }{\Lambda _\textrm{high} \varepsilon _1 r}\right)\left(\left|\uppsi _{(k+1)}\right|^2+\left|\uppsi _{(k+1)}'\right|^2\right)\textrm{d}r^*\nonumber \\&\qquad +B\int _{-\infty }^\infty \sum _{j=0}^{k} \left(|\operatorname {Re}\left[\mathfrak {G}_{(j)}'\overline{\uppsi _{(j)}}'\right]|+|\operatorname {Re}\left[\mathfrak {G}_{(j)}\overline{\uppsi _{(j)}}\right]|\right)\textrm{d}r^*\,. \end{aligned}$$

##### Remark 6.19

*(On an elliptic estimate for* k<|s|*)* Lemma 6.18 above should be compared to Lemma 5.5. While the latter provides an unconditional hierarchy of estimates for the transformed system, to that is converts estimates in $\uppsi _{(k)}$ into estimates on $\uppsi _{(k+1)}$, because it is based on the transport equations (4.17) the procedure necessarily loses derivatives. In contrast, Lemma 6.18 is an elliptic estimate for the wave equations (4.19) so it does not lose derivatives, but its range of applicability is smaller.

##### Proof of Lemma 6.18

Consider the constraint equation (4.23), and recall that $\underline{\mathcal {L}}=\frac{\textrm{d}}{\textrm{d}r^*}-i\omega +i\frac{am}{r^2+a^2}$ in frequency space. Multiplying (4.23) by $\overline{\uppsi _{(k)}}$ and taking real parts, we obtain the identity

$$\begin{aligned} w(\Lambda -2am\omega )\left|\uppsi _{(k)}\right|^2&= -w^2\left|\uppsi _{(k+1)}\right|^2\\&\quad -2w\left(\omega -\frac{am}{r^2+a^2}\right)\operatorname {Im}\left[\uppsi _{(k+1)}\overline{\uppsi _{(k)}}\right]-U_{(k)}\left|\uppsi _{(k)}\right|^2\\&\quad +{{\,\textrm{sign}\,}}s (|s|-k)w'\operatorname {Re}\left[\uppsi _{(k+1)}\overline{\uppsi _{(k)}}\right]\\&\quad +\frac{1}{2} \left(-{{\,\textrm{sign}\,}}s w\operatorname {Re}\left[\overline{\uppsi _{(k)}}\uppsi _{(k+1)}\right]\right)' -\operatorname {Re}\left[\mathfrak {G}_k\overline{\uppsi _{(k)}}\right]\\&\quad -w\sum _{j=0}^{k-1}\operatorname {Re}\left[\overline{\uppsi _{(k)}}\left(aim c_{s,\,k,\,j}^\Phi +ac^\textrm{id}_{s,\,k,\,j}\right)\uppsi _{(j)}\right]\,, \end{aligned}$$

where we have introduced

$$\begin{aligned} U_{(k)}(r)&{:}{=}w\left\{ |s|+k(2|s|-k-1)+a^2w[1-2s-2k(2|s|-k-1)]\right. \\&\qquad \left. +\frac{2Mr(r^2-a^2)}{(r^2+a^2)^2}[1-3|s|+2s^2-3k(2|s|-k-1)]\right\} \,. \end{aligned}$$

One can check that $U_{(k)}\geqq \frac{1}{8} w$, see for instance the proof of Lemma 6.36(i) below. After integration in $r^*$, the boundary terms produced by the term $\left(w\operatorname {Re}\left[\overline{\uppsi _{(k)}}\uppsi _{(k+1)}\right]\right)'$ vanish by the assumptions on $\uppsi _{(k)}$. Thus, we have

$$\begin{aligned}&\int _{-\infty }^\infty w(\Lambda -2am\omega )\left|\uppsi _{(k)}\right|^2\textrm{d}r^*\\&\leqq \int _{-\infty }^\infty w\left[\varepsilon _1\left(\omega ^2+\frac{a^2m^2}{Mr_+}\right)+1\right]\left|\uppsi _{(k)}\right|^2\textrm{d}r^*\\&\quad +B\int _{-\infty }^\infty w\left(\varepsilon _1^{-1}+1\right)|\uppsi _{(k+1)}|^2\\&\quad +\sum _{j=0}^k\int _{-\infty }^\infty w\varepsilon _1^{-1}\left|\uppsi _{(j)}\right|^2\textrm{d}r^*-\int _{-\infty }^\infty \operatorname {Re}[\mathfrak G_{(k)}\overline{\uppsi _{(k)}}]\textrm{d}r^* \end{aligned}$$

or, alternatively,

$$\begin{aligned}&\int _{-\infty }^\infty w(\Lambda -2am\omega )\left|\uppsi _{(k)}\right|^2\textrm{d}r^* \\&\quad \leqq \int _{-\infty }^\infty w\left(\varepsilon _2\Lambda _\textrm{high}+\varepsilon _1\frac{a^2m^2}{Mr_+}+1\right)\left|\uppsi _{(k)}\right|^2\textrm{d}r^*\\&\qquad +B\int _{-\infty }^\infty w\left(\frac{(\varepsilon _1^{-1}+1)}{r^2+a^2}+\frac{\omega ^2}{\varepsilon _2\Lambda _\textrm{high}}\right)|\uppsi _{(k+1)}|^2\\&\qquad +\sum _{j=0}^k\int _{-\infty }^\infty w\varepsilon _1^{-1}\left|\uppsi _{(j)}\right|^2\textrm{d}r^*-\int _{-\infty }^\infty \operatorname {Re}[\mathfrak G_{(k)}\overline{\uppsi _{(k)}}]\textrm{d}r^*\,. \end{aligned}$$

If the stated conditions on $\varepsilon _1,\varepsilon _2,\varepsilon _3$, $\frac{\omega ^2}{\Lambda }$ and $\Lambda _\textrm{high}$ hold, we can iterate the above estimates for k=0,⋯,|s|-1. This concludes the proof of (6.23) and (6.26).

For (6.24) and (6.27), we start by commuting (4.23) with $\frac{\textrm{d}}{\textrm{d}r^*}$, and then repeat the above procedure. □

The next estimate allows us to trade ω by *m* factors in the error terms generated by the applying Killing energy currents, see (5.8), to our transformed system. This is a generalization of the trick introduced in [47, Proposition 5.3.1] to address the former type of error, and only requires the transport identity (4.17).

##### Lemma 6.20

(Basic transport estimate 2) Let $$c(r^*)$$ be a continuously differentiable function such that *c* and $c^{\prime }$ are bounded in $$r^*\in \mathbb {R}$$; let $$0\leqq \chi \leqq 1$$ be a bump function compactly supported in $$(r_+,\infty )$$. For any ε>0, we have

6.28 $$\begin{aligned}&\int _{-\infty }^\infty wc(r)\operatorname {Im}\left[i\omega \overline{\Psi }m\uppsi _{(k)}\right]\textrm{d}r^* \nonumber \\&\quad \leqq B\int _{-\infty }^{\infty }\left\rbrace \varepsilon w(1-\chi )^2\omega ^2|\Psi |^2+ w m^2\left|\uppsi _{(k+1)}\right|^2\right\lbrace \textrm{d}r^*\,,\nonumber \\&\quad \qquad + B\int _{-\infty }^{\infty }\left\rbrace (|\chi '|+w)\omega ^2 \left|\uppsi _{(|s|-1)}\right|^2+\varepsilon ^{-1}(|\chi '|+w)m^2\left|\uppsi _{(k)}\right|^2\right\lbrace \textrm{d}r^*\,, \end{aligned}$$

if k=0,⋯|s|-2, and if k=|s|-1,

6.29 $$\begin{aligned}&\int _{-\infty }^\infty wc(r)\operatorname {Im}\left[i\omega \overline{\Psi }m\uppsi _{(k)}\right]\textrm{d}r^* \nonumber \\&\quad \leqq B\int _{-\infty }^{\infty }\left\rbrace \varepsilon w |\Psi '|^2+\varepsilon w\left[1+(1-\chi )^2m^2\right]|\Psi |^2\right.\nonumber \\&\qquad \left.+\varepsilon ^{-1}(w+|\chi '|) m^2\left|\uppsi _{(k)}\right|^2\right\lbrace \textrm{d}r^*\,. \end{aligned}$$

##### Proof

Using the identity (4.17) for the term $i\omega \uppsi _{(k)}$, we obtain

6.30 $$\begin{aligned} wc\operatorname {Im}\left[i\omega \overline{\Psi }m\uppsi _{(k)}\right]&= wcm\operatorname {Im}\left[\overline{\Psi }\left(w\uppsi _{(k+1)}+{{\,\textrm{sign}\,}}s \uppsi _{(k)}'-i\frac{am}{r^2+a^2}\uppsi _{(k)}\right)\right]\nonumber \\&= cw^2 m \operatorname {Im}\left[\overline{\Psi }\uppsi _{(k+1)}\right]\nonumber \\&\quad -\operatorname {Im}\left[\left(cw\frac{iam^2}{r^2+a^2}+(wc)'m{{\,\textrm{sign}\,}}s\right)\overline{\Psi }\uppsi _{(k)}\right] \nonumber \\&\quad -cwm {{\,\textrm{sign}\,}}s \operatorname {Im}\left[\overline{\Psi }'\uppsi _{(k)}\right]+\left(cwm{{\,\textrm{sign}\,}}s \operatorname {Im}\left[\overline{\Psi }\uppsi _{(k)}\right]\right)'\,. \end{aligned}$$

After integration in $r^*$, the boundary terms vanish due to the asymptotics of $\uppsi _{(k)}$. Now suppose k=|s|-1; we have

$$\begin{aligned} \begin{aligned} \operatorname {Im}\left[\overline{\Psi }\uppsi _{(k+1)}\right]&= 0\,,\\ wc\operatorname {Im}\left[i\overline{\Psi }\uppsi _{(k)}\right]&= -\frac{1}{2}c{{\,\textrm{sign}\,}}s \left(\left|\uppsi _{(k)}\right|^2\right)'\\&=-\left(\frac{1}{2}c{{\,\textrm{sign}\,}}s \left|\uppsi _{(k)}\right|^2\right)'+ \frac{1}{2}c'{{\,\textrm{sign}\,}}s \left|\uppsi _{(k)}\right|^2\,. \end{aligned} \end{aligned}$$

For the boundary term produced here to vanish, *c* must itself vanish as $r^*\rightarrow \pm \infty$ at a suitable rate; we use a cutoff function χ so that this is the case: since $acw(r^2+a^2)^{-1}=acw(r^2+a^2)^{-1}\left[\chi +(1-\chi )\right]$, we have

$$\begin{aligned} \begin{aligned}&-{{\,\textrm{sign}\,}}s\int _{-\infty }^\infty wc(r)\operatorname {Im}\left[i\omega \overline{\Psi }m\uppsi _{(k)}\right]\textrm{d}r^* \\&\quad = \int _{-\infty }^\infty \left\rbrace wcm\operatorname {Im}\left[\overline{\Psi }'\uppsi _{(k)}\right]+(wc)'m\operatorname {Im}\left[\overline{\Psi }\uppsi _{(k)}\right]\right\lbrace \textrm{d}r^*\\&\qquad +\int _{-\infty }^\infty \left\rbrace {{\,\textrm{sign}\,}}s \frac{a}{r^2+a^2}cw(1-\chi )m^2\operatorname {Re}\left[\overline{\Psi }\uppsi _{(k)}\right]\right.\\&\qquad \left.+\frac{1}{2} \left(c\frac{a}{r^2+a^2}\chi \right)'m^2\left|\uppsi _{(k)}\right|^2\right\lbrace \textrm{d}r^*\,, \end{aligned} \end{aligned}$$

Then, estimate (6.29) follows by Cauchy–Schwarz.

Alternatively, the identity (4.17) can be applied to the Ψ term to lower it: letting K=|s|-1,

6.31 $$\begin{aligned} wc\operatorname {Im}\left[i\omega \overline{\Psi }m\uppsi _{(k)}\right]&= c\omega \operatorname {Im}\left[\left(-{{\,\textrm{sign}\,}}s \overline{\uppsi _{(K}}'+i\omega \overline{\uppsi _{(K)}} -\frac{iam}{r^2+a^2}\overline{\uppsi _{(K)}}\right)im\uppsi _{(k)}\right] \nonumber \\&=\left(-{{\,\textrm{sign}\,}}s c\omega \operatorname {Im}\left[\overline{\uppsi _{(K)}}im\uppsi _{(k)}\right]\right)'+{{\,\textrm{sign}\,}}s c'\omega \operatorname {Im}\left[\overline{\uppsi _{(K)}}im\uppsi _{(k)}\right]\nonumber \\&\qquad -c\omega \operatorname {Im}\left[\overline{\uppsi _{(K)}}\left(-{{\,\textrm{sign}\,}}s \uppsi _{(k)}'-i\omega \uppsi _{(k)}+\frac{iam}{r^2+a^2}\uppsi _{(k)}\right)\right]\nonumber \\&=\left(-{{\,\textrm{sign}\,}}s c\omega \operatorname {Im}\left[\overline{\uppsi _{(K)}}im\uppsi _{(k)}\right]\right)'+{{\,\textrm{sign}\,}}s c'\omega \operatorname {Im}\left[\overline{\uppsi _{(K)}}im\uppsi _{(k)}\right]\nonumber \\&\qquad -cw\omega \operatorname {Im}\left[\overline{\uppsi _{(K)}}im\uppsi _{(k+1)}\right]\,. \end{aligned}$$

In this identity, upon integration, the boundary term only vanishes if *c* itself vanishes; we use a bump function χ with compact support in $(r_+,\infty )$ so that this is the case. Thus, when k<|s|, we consider

$$\begin{aligned} wc\operatorname {Im}\left[i\omega \overline{\Psi }m\uppsi _{(k)}\right]&= wc(1-\chi )\operatorname {Im}\left[i\omega \overline{\Psi }m\uppsi _{(k)}\right] + (c\chi )'\omega \operatorname {Im}\left[\overline{\uppsi _{(|s|-1)}}im\uppsi _{(k)}\right]\\&\qquad +c\chi w\omega \operatorname {Im}\left[\overline{\uppsi _{(|s|-1)}}im\uppsi _{(k+1)}\right]\\&\qquad +\left(-{{\,\textrm{sign}\,}}s c\chi \omega \operatorname {Im}\left[\overline{\uppsi _{(K)}}im\uppsi _{(k)}\right]\right)'\,, \end{aligned}$$

to which an application of Cauchy–Schwarz yields (6.28). □

#### Angular dominated and non-trapped superradiant regimes

This section concerns the frequency ranges  and $\mathcal {F}_{\measuredangle }$. We will prove the following two results:

##### Proposition 6.21

(Estimates in $\mathcal {F}_{\measuredangle }\backslash \mathcal {F}_{\measuredangle ,3}$) Fix $s\in \mathbb {Z}$, M>0 and $a_0\in (0,M)$. Then, for all $\delta _I,\delta _H\in (0,1]$, for all $a_0\in (0,M)$, for all E>0 sufficiently large, for all $\beta _1>0$ sufficiently small depending on $a_0$, for all $\varepsilon ^{-1}_\textrm{width}$ sufficiently large depending on $a_0$, for all $\beta _2$ sufficiently small depending on $a_0$ and $\varepsilon _\textrm{width}$ that in particular $\beta _2\leqq \sqrt{\varepsilon _\textrm{width}}$, for all $\omega _\textrm{high}$ sufficiently large depending on *E*, and for all $(a,\omega ,m,\Lambda ) \in \mathcal {F}_{\measuredangle ,1}(\omega _\textrm{high},\varepsilon _\textrm{width},a_0,\beta _1)\cup \mathcal {F}_{\measuredangle ,2}(\omega _\textrm{high},\varepsilon _\textrm{width},a_0,\beta _2)$, there exist functions f≧0, y≧0, $\hat{y}\geqq 0$, h≧0, $0\leqq \chi _1\leqq 1$ and $0\leqq \chi _2\leqq 1$ satisfying the bounds (6.3) such that

$$\begin{aligned}&\omega ^2\left|\Psi (\infty )\right|^2+(\omega -m\upomega _+)^2\left|\Psi (-\infty )\right|^2 \\&\qquad + b(\delta _I,\delta _H,a_0,\beta _1,\beta _2)\int _{-\infty }^\infty \frac{\Delta }{r^{2}}\Big [\frac{(1-Mr^{-1})^{\delta _H-1}}{r^{1+\delta _I}}|\Psi '|^2\\&\qquad +\left(\frac{(1-Mr^{-1})^{\delta _H-1}}{r^{1+\delta _I}}\omega ^2+\frac{1-Mr^{-1}}{r^3}\Lambda \right)|\Psi |^2\Big ]\textrm{d}r^*\\&\quad \leqq -\int _{-\infty }^\infty \left\rbrace 2(y+\hat{y}+f)\operatorname {Re}\left[\mathfrak {G}\overline{\Psi }'\right]+(h+f')\operatorname {Re}\left[\mathfrak {G}\overline{\Psi }\right]\right.\\&\qquad \qquad \qquad \left.-E\left[\omega \chi _2+(\omega -m\upomega _+)\chi _1\right]\operatorname {Im}\left[\mathfrak {G}\overline{\Psi }\right]\right\lbrace \textrm{d}r^*\\&\qquad +B(E,\omega _\textrm{high})\sum _{k=0}^{|s|-1}\int _{-\infty }^\infty \left|\operatorname {Re}\left[\mathfrak {G}_{(k)}\uppsi _{(k)}\right]\right|\textrm{d}r^*\,. \end{aligned}$$

The same result holds for all $(\omega ,m,\Lambda )\in \mathcal {F}_{\measuredangle ,3}\cap \{m=0\}$.

##### Proposition 6.22

(Estimates in ) Fix $s\in \mathbb {Z}$ and M>0. Then, for all $\delta _I,\delta _H\in (0,1]$, for all $a_0\in (0,M)$, for all E>0 sufficiently large, for all $\beta _1>0$ sufficiently small depending on $a_0$, for all $\varepsilon ^{-1}_\textrm{width}$ sufficiently large depending on $a_0$, for all $\beta _2$ sufficiently small depending on $a_0$ and $\varepsilon _\textrm{width}$ that in particular $\beta _2\leqq \sqrt{\varepsilon _\textrm{width}}$, for all $\omega _\textrm{high}$ sufficiently large depending on *E*, $\varepsilon _\textrm{width}$ and $\beta _2$, and for all , there exist functions f≧0, y≧0, $\hat{y}\geqq 0$, h≧0, $0\leqq \chi _1\leqq 1$ and $0\leqq \chi _2\leqq 1$ satisfying the bounds (6.3) such that

$$\begin{aligned}&\omega ^2\left|\Psi (\infty )\right|^2+(\omega -m\upomega _+)^2\left|\Psi (-\infty )\right|^2 + b(\delta _I,\delta _H,a_0,\beta _1,\beta _2)\\&\int _{-\infty }^\infty \frac{\Delta }{r^{3+\delta _I}}\left(1-\frac{M}{r}\right)^{\delta _H-1}\Big [|\Psi '|^2+\omega ^2|\Psi |^2\Big ]\textrm{d}r^*\\&\quad \leqq -\int _{-\infty }^\infty \left\rbrace 2(y+\hat{y}+f)\operatorname {Re}\left[\mathfrak {G}\overline{\Psi }'\right]+(h+f')\operatorname {Re}\left[\mathfrak {G}\overline{\Psi }\right]\right.\\&\qquad \qquad \qquad \left.-E\left[\omega \chi _2+(\omega -m\upomega _+)\chi _1\right] \operatorname {Im}\left[\mathfrak {G}\overline{\Psi }\right]\right\lbrace \textrm{d}r^*\\&\qquad +B(E,\varepsilon _\textrm{width},\omega _\textrm{high})\sum _{k=0}^{|s|-1}\int _{-\infty }^\infty \left|\operatorname {Re}\left[\mathfrak G_{(k)}\uppsi _{(k)}\right]\right|\textrm{d}r^*\,. \end{aligned}$$

We begin by deriving important properties of the frequency parameters in the relevant frequency regimes:

##### Lemma 6.23

(Properties of the frequency parameters in  and $\mathcal {F}_{\measuredangle }$)

Fix $s\in \mathbb {Z}$, M>0 and $a_0\in (0,M)$. Let  be an admissible frequency triple with respect to a∈[0,M] such that $a\in [0,a_0]$ if  and $a\in (a_0,M]$ if . Then, for all $\beta _1>0$ sufficiently small depending on $a_0$, for all $\varepsilon ^{-1}_\textrm{width}$ sufficiently large depending on $a_0$, for all $\beta _2$ sufficiently small depending on $a_0$ and $\varepsilon _\textrm{width}$, for all $\omega _\textrm{high}$ sufficiently large depending on $\varepsilon _\textrm{width}$, the following hold:

(i) $\Lambda \geqq m^2$ in  and $\mathcal {F}_{\measuredangle }$, with an improved bound $\Lambda \geqq 8M^2a_0^{-1}\varepsilon _\textrm{width}^{1/2}m^2$ in $\mathcal {F}_{\measuredangle , 3}$, but $\Lambda \geqq \max \left\rbrace \frac{2}{3},\frac{a_0^2}{M^2}\right\lbrace m^2$ in ;
(ii) the conditions for Lemma 6.18 are satisfied, namely
  in $\mathcal {F}_{\measuredangle }$, we have $\Lambda \geqq \Lambda _\textrm{high}:=\varepsilon _\textrm{width}^{-1}\omega _\textrm{high}^2\gg 1$ and
    $$\begin{aligned} \Lambda -2am\omega -\varepsilon _1\frac{a^2}{2Mr_+}m^2-\varepsilon _2\Lambda _\textrm{high}\geqq \varepsilon _3 \Lambda \,, \quad \varepsilon _1=\varepsilon _2=\frac{1}{8}, \,\, {\varepsilon }_3=\frac{1}{2}\,, \end{aligned}$$
    where we note $(\sqrt{\varepsilon }_1\varepsilon _3\Lambda _\textrm{high})^{-2}\leqq B \Lambda _\textrm{high}^{-2}\ll 1$ and $\frac{\omega ^2}{\Lambda }\leqq \varepsilon _\textrm{width}^{-1}\ll \Lambda _\textrm{high}$ as long as $\omega _\textrm{high}$ is sufficiently large;
  in , we have $\Lambda \geqq \Lambda _\textrm{high}:=\varepsilon _\textrm{width}\omega _\textrm{high}^2\gg 1$ and
    $$\begin{aligned}&\Lambda -2am\omega -\varepsilon _1\left(\omega ^2+m^2\right)\geqq \varepsilon _3 \Lambda \,,\\&\quad \varepsilon _1=\frac{\sqrt{M^2-a_0^2}}{3M}\varepsilon _\textrm{width},\,\, {\varepsilon }_3= \frac{\sqrt{M^2-a_0^2}}{12M}\,, \end{aligned}$$
    where we note $(\sqrt{\varepsilon }_1\varepsilon _3\Lambda _\textrm{high})^{-2}\leqq B(M^2-a_0^2)^{-3/2}\varepsilon _\textrm{width}^{-3}\omega _\textrm{high}^{-4}\ll 1$ as long as $\omega _\textrm{high}$ is sufficiently large depending on $\varepsilon _\textrm{width}$ and $a_0$;
  in , we have $\Lambda \geqq \Lambda _\textrm{high}:=\varepsilon _\textrm{width}\omega _\textrm{high}^2\gg 1$ and
    $$\begin{aligned} \Lambda -2am\omega -\varepsilon _1\left(\omega ^2+\frac{a^2}{Mr_+}m^2\right)\geqq \varepsilon _3 \Lambda \,, \quad \varepsilon _1=\frac{1}{4} a_0\beta _2\varepsilon _\textrm{width}, \,\, \varepsilon _3=a_0\beta _2\,, \end{aligned}$$
    $(\sqrt{\varepsilon }_1\varepsilon _3\Lambda _\textrm{high})^{-2}\leqq Ba_0^{-3}\beta _2^{-3}\varepsilon _\textrm{width}^{-3}\omega _\textrm{high}^{-4}\ll 1$ as long as $\omega _\textrm{high}$ is sufficiently large depending on $\varepsilon _\textrm{width}$, $\beta _2$ and $a_0$.
(iii) writing $\sigma :=am\omega /(\Lambda +s^2)$, we have $|\sigma |\leqq \frac{1}{2}$; in $\mathcal {F}_{\measuredangle , 3}$ we have the stronger bound $|\sigma |\leqq 3a_0^{-1/2}M\varepsilon _\textrm{width}^{3/4}$.

##### Proof

We begin by showing that the bound $\Lambda \geqq \max \left\rbrace \frac{2}{3},\frac{a_0^2}{M^2}\right\lbrace$ holds more generally when $\Lambda \geqq \varepsilon _\textrm{width}\omega ^2$ and $|\omega |\geqq \omega _\textrm{high}$. If $m^2\leqq \varepsilon _\textrm{width}\omega ^2$, of course $\Lambda \geqq m^2$. On the other hand, if $m^2>\varepsilon _\textrm{width}\omega ^2$ then in particular |m|≫|s|, so it will be convenient to recall condition IV(b) of Definition 4.1:

$$\begin{aligned} \Lambda \geqq l(l+1)-s^2-2|s||a\omega | \,. \end{aligned}$$

If $\omega _\textrm{high}$ is sufficiently large and either $0\leqq |a|< \tilde{a}_0\ll M$ sufficiently small or $m^2\geqq \varepsilon _\textrm{high}^{-1}\omega ^2$, for instance, clearly $\Lambda \geqq m^2$. Thus, we only have to check the case $|a|\geqq \tilde{a}_0$ and $\varepsilon _\textrm{width}\omega ^2< m^2<\varepsilon _\textrm{width}^{-1}\omega ^2$. Then,

$$\begin{aligned} \Lambda&\geqq l(l+1)-s^2-2|s||a\omega |\\&\geqq (1-\epsilon )m^2+(\epsilon \omega _\textrm{high}\varepsilon _\textrm{width}+\sqrt{\varepsilon _\textrm{width}}-2|s|M-s^2\omega _\textrm{high}^{-1})|\omega | \geqq (1-\epsilon )m^2\,, \end{aligned}$$

for any $\epsilon \leqq 4\,M|s|\omega _\textrm{high}^{-1}\varepsilon _\textrm{width}^{-1}$ as long as $\omega _\textrm{high}\gg \varepsilon _\textrm{width}^{-1}$ is sufficiently large. In particular, for sufficiently small $\varepsilon _\textrm{width}$ and $\omega _\textrm{high}^{-1}$, we can take $\epsilon =\min \left\rbrace \frac{1}{3},\frac{M-a_0}{M}\right\lbrace$.

The above paragraph shows (i) holds for , and moreover establishes the improvement that $\Lambda \geqq m^2$ if $m^2\leqq \varepsilon _\textrm{width}\omega ^2$ or if $m^2\geqq \varepsilon _\textrm{high}^{-1}\omega ^2$. In the subrange , since

$$\begin{aligned} a_0\sqrt{\varepsilon _\textrm{width}}<q:=\frac{a\omega }{m}=a\upomega _+-\frac{a\beta _2\Lambda }{m^2}\leqq \frac{a^2}{2Mr_+}\leqq \frac{1}{2}, \\ |m|q^2\geqq (a_0^2\varepsilon _\textrm{width})(\varepsilon _\textrm{width}\omega _\textrm{high})= a_0^2\varepsilon _\textrm{width}^2\omega _\textrm{high}, \end{aligned}$$

if $\omega _\textrm{high}$ is chosen sufficiently large depending on $\varepsilon _\textrm{width}$ and $a_0$, by item VI(d) of the admissibility conditions on Λ laid out in Definition 4.1, we conclude that $\Lambda \geqq m^2$.

We now prove (i) for $\mathcal {F}_{\measuredangle }$, to that is when $\Lambda \geqq \max \{\varepsilon _\textrm{width}^{-1}\omega ^2, \varepsilon _\textrm{width}^{-1}\omega _\textrm{high}^2\}$. Clearly, the conclusion follows if $m^2\leqq \varepsilon _\textrm{width}^{-1}\omega ^2_\textrm{high}$ or if $m^2\leqq \varepsilon _\textrm{width}^{-1}\omega ^2$. The remaining cases can be dealt with using condition IV(b) from Definition 4.1: as long as $\varepsilon _\textrm{width}^{-1}$ and $\omega _\textrm{high}$ sufficiently large, if $m^2>\varepsilon _\textrm{width}^{-1}\omega ^2$ and $|\omega |\geqq \omega _\textrm{high}$,

$$\begin{aligned} \Lambda -m^2\geqq \max \{|m|,|s|\}-s^2-2|s||a\omega |\geqq (\varepsilon _\textrm{width}^{-1/2}-2|s|M)|\omega |-s^2\geqq 0\,; \end{aligned}$$

if $$m^2>\varepsilon _\textrm{width}^{-1}\omega _\textrm{high}^2$$ and $$|\omega |< \omega _\textrm{high}$$,

$$\begin{aligned} \Lambda -m^2\geqq \max \{|m|,|s|\}-s^2-2|s||a\omega |\geqq (\varepsilon _\textrm{width}^{-1/2}-2|s|M)\omega _\textrm{high}-s^2\geqq 0\,. \end{aligned}$$

In the subrange $\mathcal {F}_{\measuredangle , 3}$, using that $\Lambda \geqq m^2$ we deduce

$$\begin{aligned} \frac{|m\omega |}{\Lambda }\leqq \frac{\varepsilon _\textrm{width}^{1/2}\Lambda }{\Lambda }=\varepsilon _\textrm{width}^{1/2}, \end{aligned}$$

from where we obtain that

$$\begin{aligned} \frac{a_0}{4\,M^2}m^2 \leqq m^2\upomega _+\leqq m\omega +\beta _2\Lambda \leqq (\varepsilon _\textrm{width}^{1/2}+\beta _2)\Lambda \leqq 2\varepsilon _\textrm{width}^{1/2}\Lambda , \end{aligned}$$

as long as $\beta _2\leqq \varepsilon _\textrm{width}^{1/2}$.

For (ii), first consider $\mathcal {F}_\measuredangle$, in which

$$\begin{aligned} \Lambda -2am\omega -\varepsilon _1m^2-\varepsilon _2\Lambda _\textrm{high}&\geqq \Lambda \left(1-2M\varepsilon _\textrm{width}^{1/2}\Lambda -\varepsilon _1-\varepsilon _2\right)\Lambda \geqq \frac{1}{2} \Lambda \,, \end{aligned}$$

for $\varepsilon _1,\varepsilon _2\in (0,1/4]$ and $\varepsilon _\textrm{width}\leqq 1$. In ,

$$\begin{aligned}&\Lambda -2am\omega -\varepsilon _1\left(\omega ^2+\frac{a^2}{Mr_+} m^2\right) \geqq \left(1-(1+\varepsilon _1)\frac{a_0^2}{Mr_+}\frac{m^2}{\Lambda }-2a_0\beta _1-\varepsilon _1\varepsilon _\textrm{width}^{-1}\right)\Lambda \\&\quad \geqq \left(\frac{\sqrt{M^2-a_0^2}}{M}-\frac{M^2-a_0^2}{3M^2}-\varepsilon _1(\varepsilon _\textrm{width}^{-1}+1)\right)\Lambda \geqq \frac{\sqrt{M^2-a_0^2}}{12M}\Lambda \,, \end{aligned}$$

where we have used $a_0\beta _1<(M^2-a_0^2)/(6M^2)$, as long as $\varepsilon _1\leqq \frac{\sqrt{M^2-a_0^2}}{3\,M}\varepsilon _\textrm{width}$ and we have that $\varepsilon _\textrm{width}$ is sufficiently small. Finally, in , if $a_0>0$

$$\begin{aligned} \Lambda -2am\omega -\varepsilon _1(\omega ^2+m^2)\geqq \Lambda \left[1-\frac{a^2}{Mr_+}+2a\beta _2-\varepsilon _1(\varepsilon _\textrm{width}^{-1}+1)\right]\geqq a_0\beta _2\Lambda \,, \end{aligned}$$

as long as $\varepsilon _1\leqq a_0\beta _2\varepsilon _\textrm{width}/4$. Finally, (iii) follows similarly. □

Next, we obtain a description of $\mathcal {V}$ in the relevant frequency ranges:

##### Lemma 6.24

(Behavior of the potential in  and $\mathcal {F}_{\measuredangle }$) Fix $s\in \mathbb {Z}$, M>0 and $a_0\in (0,M)$. For all $\beta _1$ and $\varepsilon _\textrm{width}$ sufficiently small depending on $a_0$, for all $\beta _2$ depending on $a_0$ and $\varepsilon _\textrm{width}$, for all sufficiently large $\omega _\textrm{high}$ depending on $\varepsilon _\textrm{width}$, and for all , the potential $\mathcal {V}$ has a critical point $r_\textrm{max}$ that satisfies $|r_\textrm{max}-r_\textrm{max}^0|\leqq B\Lambda ^{-1}$ and

$$\begin{aligned} \mathcal {V}(r)-\omega ^2 \geqq b\Lambda \,,&\quad \forall \, r\in (r_\textrm{max}-\tilde{\delta }, r_\textrm{max}+\tilde{\delta })\,, \end{aligned}$$

for some $\tilde{\delta }$ and *b* which, (a) if $m\omega \leqq 0$ or $(\omega ,m,\Lambda )\in \mathcal {F}_{\mathrm {\measuredangle },3}$, are independent of the frequency parameters, and (b) otherwise depend on $a_0$ if  or $a_0\beta _2$ if . Moreover, in the case (b) there is a constant *b* with the same dependencies such that

$$\begin{aligned} -(r-r_\textrm{max})\frac{\textrm{d}\mathcal {V}}{\textrm{d}r} \geqq b\Lambda \frac{(r-r_\textrm{max})^2}{r^4}\,,&\quad \forall \, r\in [r_+,\infty )\,; \end{aligned}$$

in the case $m\omega \leqq 0$, independently of the frequency parameters, we have

$$\begin{aligned} -(r-r_\textrm{max})\frac{\textrm{d}\mathcal {V}}{\textrm{d}r} \geqq b\Lambda \frac{(r-M)(r-r_\textrm{max})^2}{r^5}\,,&\quad \forall \, r\in [r_+,\infty )\,. \end{aligned}$$

##### Proof

It is clear that we can choose $\beta _1=\beta _1(a_0)>0$ sufficiently small for Lemma 6.14 to hold, and that we can choose $\beta _2=\beta _2(a_0, \varepsilon _\textrm{width})$ sufficiently small that both Lemma 6.14 with $\tilde{\epsilon }=\varepsilon _\textrm{width}$ and Lemma 6.23(i) hold, as long as $\varepsilon _\textrm{width}=\varepsilon _\textrm{width}(a_0)$ is sufficiently small.

Let us first consider . By Lemma 6.23 and direct inspection, we find that all of the conditions of Lemma 6.14 are met, and hence the claim here holds for $\mathcal {V}$ replaced by $\mathcal {V}_0$. Similarly, if $(\omega ,m,\Lambda )\in \mathcal {F}_{\mathrm {\measuredangle },3}$, then in view of Lemma 6.23(i), as long as $\varepsilon _\textrm{width}$ is sufficiently small we can apply Lemma 6.12 to obtain the desired conclusions with $\mathcal {V}$ replaced by $\mathcal {V}_0$. To conclude, we just note that in all the relevant frequency regimes, the frequency-independent potential $\mathcal {V}_1$ satisfies

$$\begin{aligned} \left|\mathcal {V}_1\right| \leqq Br^{-3}\,, \quad \left|\frac{\textrm{d}\mathcal {V}_1}{\textrm{d}r}\right|\leqq Br^{-4}\,,\quad \left|\frac{\textrm{d}}{\textrm{d}r}\left((r^2+a^2)^3\frac{\textrm{d}\mathcal {V}_1}{\textrm{d}r}\right)\right|\leqq Br\,; \end{aligned}$$

hence if $\omega _\textrm{high}$ is sufficiently large, $\mathcal {V}_0$ dominates. □

In Lemma 6.24, we do not provide global estimates for $\mathcal {V}'$ for $(\omega ,m,\Lambda )\in \mathcal {F}_{\measuredangle ,3}$ if m≠0, as one cannot rule out the possibility of a ($\sqrt{\varepsilon _\textrm{width}}$-)small minimum ($\sqrt{\varepsilon _\textrm{width}}$-)close to $r=r_+$, see Lemma 6.12. At least under the stronger bound $m^2\leqq \omega ^{-2}_\textrm{high}\Lambda$ and for s=0, it has been shown in [70], after the conclusion of the research reported here, that the procedure for the proof of Proposition 6.21 for $\mathcal {F}_{\measuredangle ,1}\cup \mathcal {F}_{\measuredangle ,2}$ can be repeated in $\mathcal {F}_{\measuredangle ,3}$ if one additionally invokes redshift-type multiplier currents to control the errors created by the possible minimum. We do not pursue this here.

**Fig. 4:**
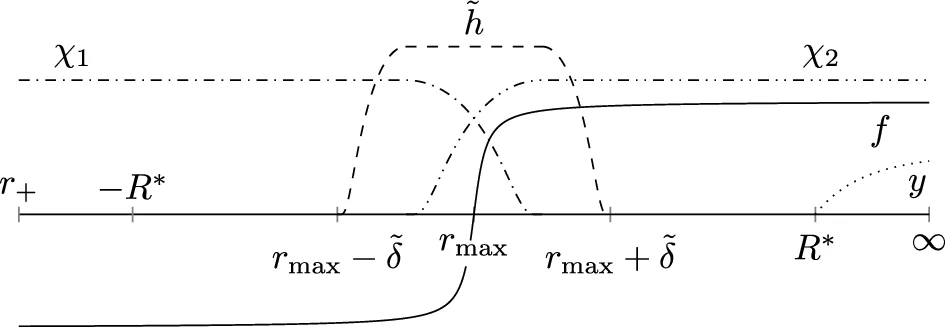
Sketch of currents in the proofs of Propositions 6.21 and 6.22

Let us turn to a brief description of our strategy to prove Proposition 6.21 for $\mathcal {F}_{\measuredangle ,1}\cup \mathcal {F}_{\measuredangle ,2}$ and Proposition 6.22, which is best illustrated by Fig. 4. Lemma 6.24 has shown that $\mathcal {V}'$ is positive as $r\rightarrow r_+$ and negative as $r\rightarrow \infty$, with the single zero being at $r=r_\textrm{max}(\omega ,m,\Lambda )$. To exploit this detailed knowledge of $\mathcal {V}'$, we can employ *y*-type virial currents localized to the large $|r^*|$ regions, as well as an *f*-type virial current in the bounded $|r^*|$ region. Unlike the former, the latter does not give us the full control we expect in Proposition 6.21: because the *f* current interacts with the potential through $\mathcal {V}'$, see (5.3), it loses control at $r=r_\textrm{max}$. Using the fact that $\mathcal {V}(r)-\omega ^2$ is quantitatively positive close to $r=r_\textrm{max}$, we eliminate the degeneration by introducing an *h*-type current. As the *f* and *y* currents produce bad boundary terms, and the frequency regimes we consider may be superradiant, we add *T* and *K* Killing energy currents which are localized to the region close to $r=r_\textrm{max}$.

For s≠0, these separated currents produce by interacting with the coupling terms in (4.21). To control them, we appeal to the elliptic estimate of Lemma 6.18, exploiting the strong positivity of $\Lambda -2am\omega$ obtained in Lemma 4.3(ii).

##### Proof of Propositions 6.21 and 6.22

Suppose $(\omega ,m,\Lambda )$ is an admissible frequency triple with respect to a∈[0,M] with  or $(a,\omega ,m,\Lambda )\in \mathcal {F}_{\measuredangle ,3}(\varepsilon _\textrm{width},\omega _\textrm{high},a_0,\beta _2)\cap \{m=0\}$. We assume $\beta _1=\beta _1(a_0)>0$ is sufficiently small for Lemma 6.14 to hold, $\varepsilon _\textrm{width}=\varepsilon _\textrm{width}(a_0)$ and $\beta _2=\beta _2(a_0, \varepsilon _\textrm{width})$ are sufficiently small that Lemma 6.14 and Lemma 6.23 hold, $\omega _\textrm{high}= \omega _\textrm{high}(\varepsilon _\textrm{width})$ is sufficiently large that Lemma 6.23 and Lemma 6.24 hold. We employ *h*, *y* and *f* type virial currents, as well as *T* and *K* type Killing energy currents.

*The main terms in *(4.21). Recall the definitions of $r_\textrm{max}$ and $\tilde{\delta }$ from Lemma 6.24. Let $0\leqq \tilde{h}\leqq 1$ be a smooth function supported for $r\in [r_\textrm{max}-\tilde{\delta },r_\textrm{max}+\tilde{\delta }]$ such that $\tilde{h}=1$ in $r\in [r_\textrm{max}-\tilde{\delta }/2,r_\textrm{max}+\tilde{\delta }/2]$. For $\delta _H\in (0,1]$ and some $R^*>1$ and A>0 to be fixed, set

$$\begin{aligned}  &   y(r)=\left(1-\frac{(R^*)^{\delta _I}}{(r^*)^{\delta _I}}\right)\mathbb {1}_{[R^*,\infty )},\\  &   \hat{y}(r)=\left(1-\frac{1}{2\pi }\arctan (r_\textrm{max}-r_+)\right)\left(1-\frac{(R^*)^{\delta _H}}{|r^*|^{\delta _H}}\right)\mathbb {1}_{(-\infty ,-R^*]}, \\  &   f(r)=\frac{1}{\pi }\arctan (r-r_\textrm{max}),\qquad h(r)=A\tilde{h}(r). \end{aligned}$$

By Lemma 6.24, $\mathcal {V}'$ is, up to degeneration, positive as $r\rightarrow r_+$. Thus, we have

$$\begin{aligned} \hat{y}'(\omega -m\upomega _+)^2-(\hat{y}\hat{\mathcal {V}})'&\geqq \hat{y}'(\omega -m\upomega _+)^2-\hat{y}' |\omega -m\upomega _+|(r-M) -\frac{3}{4}\hat{y} \hat{\mathcal {V}}'\\&\geqq \frac{1}{2} \hat{y}'(\omega -m\upomega _+)^2 - \frac{1}{2}\hat{y} \hat{\mathcal {V}}'\,, \end{aligned}$$

where $\hat{\mathcal {V}}:=\mathcal {V}-\mathcal {V}(r_+)$, as long as $R^*$ is chosen to be sufficiently large depending on $\delta _H\in (0,1]$. Similarly,

$$\begin{aligned} y'\omega ^2-(y\mathcal {V})'\geqq y'\omega ^2\,, \end{aligned}$$

if $R^*$ is chosen to be sufficiently large depending on $\delta _I$; we fix it so that the two lower bounds hold. Also from Lemma 6.24, we deduce that

$$\begin{aligned}&-f\mathcal {V}'\geqq b\frac{(r-M)\Delta (r-r_\textrm{max})^2}{r^8}\Lambda \text { if }m\omega \leqq 0\,; \\&-f\mathcal {V}'\geqq b(a_0,\beta _1,\beta _2)\frac{\Delta (r-r_\textrm{max})^2}{r^7}\Lambda \text { if }m\omega > 0\,. \end{aligned}$$

For $m\omega \leqq 0$, it must be the case that $(\omega ,m,\Lambda )\in \mathcal {F}_{\measuredangle }$, so by taking $\omega _\textrm{high}$ and/or $\varepsilon _\textrm{width}^{-1}$ sufficiently large,

$$\begin{aligned} -f\mathcal {V}'-\frac{1}{2}f'''\geqq -\frac{3}{4}f\mathcal {V}'-B\mathbb {1}_{\{|r-r_\textrm{max}|\leqq \tilde{\delta }/2\}}\,; \end{aligned}$$

if mω>0, then as long as $\varepsilon _\textrm{width}\omega _\textrm{high}^2$ is sufficiently large depending on $a_0$ () or $a_0\beta _2$ (), the same conclusion holds. Thus, we can choose *A* so as to have

$$\begin{aligned}&-f\mathcal {V}'+h(\mathcal {V}-\omega ^2)-\frac{1}{2}f'''-\frac{1}{2}h'' \geqq \left[h(\mathcal {V}-\omega ^2)-B\mathbb {1}_{\{|r-r_\textrm{max}|\leqq \tilde{\delta }/2\}}\right]\\&\quad + \left[-\frac{3}{4}f\mathcal {V}' -\frac{1}{2}A\tilde{\delta }^2\mathbb {1}_{\{\tilde{\delta }/2\leqq |r-r_\textrm{max}|\leqq \tilde{\delta }\}}\right]\\&\quad \geqq -\frac{1}{2} f\mathcal {V}'+\frac{1}{2} h(\mathcal {V}-\omega ^2)\,, \end{aligned}$$

because the first term enclosed in square brackets is quantitative positive by Lemma 6.24 for $\omega _\textrm{high}$ sufficiently large, and the second term enclosed in square brackets can also be made positive provided that $A\leqq \mathcal {V}'\mathbb {1}_{\{\tilde{\delta }/2\leqq |r-r_\textrm{max}|\leqq \tilde{\delta }\}}$. The last condition is satisfied if $A\leqq b\varepsilon _\textrm{width}^{-1}\omega _\textrm{high}^2$ in $\mathcal {F}_\mathrm{\mathrm {\measuredangle }}$, if $A\leqq b(a_0)\varepsilon _\textrm{width}\omega _\textrm{high}^2$ in  and if $A\leqq b(a_0\beta _2)\varepsilon _\textrm{width}\omega _\textrm{high}^2$ in .

The above remarks show that $Q^{\hat{y}}+Q^f+Q^{h}$ applied to (4.21) produce a good bulk term. However, these currents also produce boundary terms. Let $\chi _1\leqq 1$ be a smooth function which is 1 in $[r_+,r_\textrm{max}-\tilde{\delta }/2]$ and 0 for $r\geqq r_\textrm{max}+\tilde{\delta }/2$ and $\chi _2\leqq 1$ be a smooth function which is 1 in $[r_\textrm{max}+\tilde{\delta }/2,\infty )$ and 0 for $r\in [r_+,r_\textrm{max}-\tilde{\delta }/2]$ and such that $\chi _2'=-\chi '$ in $[r_\textrm{max}-\tilde{\delta }/2, r_\textrm{max}+\tilde{\delta }/2]$. By applying $E\chi _1Q^T+E\chi _2Q^K$ currents with E≧3, we can control the boundary terms. The energy currents produce localization errors

$$\begin{aligned}&\int _{-\infty }^\infty [E\chi _1'(\omega -m\upomega _+)+E\chi _2'\omega ]\operatorname {Im}\left[\Psi '\overline{\Psi }\right]\textrm{d}r^* \\&\quad \leqq \int _{-\infty }^\infty \left[\frac{1}{2} f'|\Psi '|^2+ B(E\tilde{\delta }^{-1})^2 m^2|\Psi |^2\right]\mathbb {1}_{|r-r_\textrm{max}|\leqq \tilde{\delta }/2]}\textrm{d}r^*\\&\quad \ll \int _{-\infty }^\infty \left\rbrace \frac{1}{2} f'|\Psi '|^2+\frac{1}{4}\left[- f\mathcal {V}' +h\left(\mathcal {V}-\omega ^2\right)\right]|\Psi |^2\right\lbrace \textrm{d}r^*\,, \end{aligned}$$

as long as $\varepsilon _\textrm{width}\omega _\textrm{high}^2$ is large enough depending on *E* (and, if mω>0, depending on $\varepsilon _\textrm{width}$, $a_0$ and possibly $\beta _2$) that we can, and do, fix *A* satisfying the constraints above and still have $A\gg E^2 \tilde{\delta }^{-2}$.

Combining the previous results, we finally have the estimate

6.32 $$\begin{aligned}&(\omega -m\upomega _+)^2|\Psi (-\infty )|^2+\omega ^2|\Psi (+\infty )|^2 +\int _{-\infty }^\infty \left(\frac{1}{2}f'+h+\hat{y}'+ y'\right)|\Psi '|^2\textrm{d}r^*\nonumber \\&\quad +\int _{-\infty }^\infty \left[y'\omega ^2+\hat{y}' (\omega -m\upomega _+)^2-\frac{1}{2}\hat{y} \hat{\mathcal {V}}'- \frac{1}{4}f\mathcal {V}' +\frac{1}{4} h\left(\mathcal {V}-\omega ^2\right)\right]|\Psi |^2\textrm{d}r^*\nonumber \\&\quad \leqq -\sum _{k=0}^{|s|-1}\int _{-\infty }^\infty \left\rbrace 2w(y+\hat{y}+f) \operatorname {Re}[(ac_{s,k}^\textrm{id}+im ac_{s,k}^{\Phi })\uppsi _{(k)}\overline{\Psi }']\right.\nonumber \\&\qquad \qquad \qquad \qquad \qquad \left.+(h+f')w\operatorname {Re}[(ac_{s,k}^\textrm{id}+im ac_{s,k}^{\Phi })\uppsi _{(k)}\overline{\Psi }]\right\lbrace \textrm{d}r^*\nonumber \\&\qquad + \sum _{k=0}^{|s|-1}\int _{-\infty }^\infty Ew\left[(\omega -m\upomega _+)\chi _1+\omega \chi _2\right]\operatorname {Im}[(ac_{s,k}^\textrm{id}+im ac_{s,k}^{\Phi })\uppsi _{(k)}\overline{\Psi }]\textrm{d}r^*\nonumber \\&\qquad -\int _{-\infty }^\infty \left\rbrace 2(y+\hat{y}+f)\operatorname {Re}[\mathfrak G\overline{\Psi }']+(h+f')\operatorname {Re}[\mathfrak G\overline{\Psi }]\right.\nonumber \\&\qquad \left.\qquad \qquad -E\left[(\omega -m\upomega _+)\chi _1+\omega \chi _2\right]\operatorname {Im}[\mathfrak G\overline{\Psi }]\right\lbrace \textrm{d}r^*\,. \end{aligned}$$

If s=0, the first two lines on the right hand side of (6.32) are absent and so the proof is complete.

*The coupling terms in* (4.21). Let us now assume s≠0. We turn to the right hand side of (6.32). For the first and second lines this is controlled by

$$\begin{aligned}&\int _{-\infty }^\infty \epsilon \left[\left(h+\frac{1}{2} f'\right)|\Psi '|^2+\frac{1}{4} h(\mathcal {V}-\omega ^2)|\Psi |^2+B(A)E^2\epsilon ^{-1}(1+m^2)w|\uppsi _{(k)}|^2 \right] \textrm{d}r^*\,, \end{aligned}$$

for some ϵ>0. Thus, fixing ϵ to be sufficiently small, we can absorb the errors involving Ψ into the left hand side of (6.32). We are left with errors in $|\uppsi _{(k)}|^2$. Making use of the elliptic estimates of Lemma 6.18, respectively (6.23) in  and (6.26) in $\mathcal {F}_{\measuredangle }$, (see Lemma 6.23), we deduce that, if $\omega _\textrm{high}$ is sufficiently large depending on *E*, and also $a_0$, $\beta _1$, $\beta _2$ and $\varepsilon _\textrm{width}$ in the case of , we have

$$\begin{aligned}&\int _{-\infty }^\infty BE^2(1+m^2)w|\uppsi _{(k)}|^2 \textrm{d}r^*\\&\quad \leqq \{\varepsilon _\textrm{width}^{-2},1\}\frac{B(A)E^2}{\omega _\textrm{high}^2}\int _{-\infty }^{\infty }\left[f'|\Psi |^2+\left(y'\omega ^2-f\mathcal {V}'+h(\mathcal {V}-\omega ^2)\right)|\Psi |^2\right]\textrm{d}r^*\\&\qquad \quad +\int _{-\infty }^\infty B(A)E^2\sum _{k=0}^{|s|-1}|\operatorname {Re}[\mathfrak {G}_{(k)}\overline{\uppsi }_{(k)}]|\,, \end{aligned}$$

where the alternatives given are for $\mathcal {F}_{\measuredangle }$ and , respectively. Assuming $\omega _\textrm{high}$ is sufficiently large depending on *E* and, in , also on $\varepsilon _\textrm{width}$, $a_0$, $\beta _1$ and $\beta _2$, the first term above can be absorbed by the left hand side of (6.32). We remark that having introduced the current *y* has allowed us to improve the *r*-weights in the $\omega ^2$ bulk term (this term is controlled by the *f* current with faster decaying weights), so that (6.26) can indeed be applied in $\mathcal {F}_{\measuredangle }$. □

#### Nonsuperradiant trapping regime

This section concerns the frequency range $\mathcal {F}_\textrm{comp,1}$. We will show

##### Proposition 6.25

(Estimates in $\mathcal {F}_\textrm{comp,1}$) Fix $s\in \mathbb {Z}$ and M>0. Then, for all $\delta _I,\delta _H\in (0,1]$, for all $\beta _3>0$, for all $\varepsilon _\textrm{width}>0$ sufficiently small depending on $\beta _3$ that in particular $\beta _3\geqq \varepsilon _\textrm{width}$, for all E>0 sufficiently large depending on $\varepsilon _\textrm{width}$, for all $\omega _\textrm{high}$ sufficiently large depending $\varepsilon _\textrm{width}$ and *E*, and for all $(a,\omega ,m,\Lambda ) \in \mathcal {F}_\textrm{comp,1}(\omega _\textrm{high},\varepsilon _\textrm{width},r_0',\beta _3)$, there exist exists a value $r_\textrm{trap}\in (r_+,\infty )$ satisfying $b(\varepsilon _\textrm{width})\leqq r_\textrm{trap}-r_+\leqq B(\varepsilon _\textrm{width})$ and functions f≧0, y≧0 and $\hat{y}\geqq 0$ satisfying the bounds (6.3) such that

$$\begin{aligned}&\omega ^2\left|\Psi (\infty )\right|^2+(\omega -m\upomega _+)^2\left|\Psi (-\infty )\right|^2\\&+ b(\delta _H,\delta _I,\varepsilon _\textrm{width})\int _{-\infty }^\infty \frac{\Delta }{r^{3+\delta _I}}\\&\qquad \times \left(1-\frac{M}{r}\right)^{\delta _H-1}\Big [|\Psi '|^2+\left(1+\omega ^2(1-r^{-1}r_\textrm{trap})\right)|\Psi |^2\Big ]\textrm{d}r^*\\&\quad \leqq -\int _{-\infty }^\infty \left\rbrace 2(y+\hat{y}+f)\operatorname {Re}\left[\mathfrak {G}\overline{\Psi }'\right]+f\operatorname {Re}\left[\mathfrak {G}\overline{\Psi }\right]-E\omega \operatorname {Im}\left[\mathfrak {G}\overline{\Psi }\right]\right\lbrace \textrm{d}r^*\\&\qquad +B(\varepsilon _\textrm{width},E)\sum _{k=0}^{|s|-1}\int _{-\infty }^\infty \left|\operatorname {Re}\left[\mathfrak {G}_{(k)}\uppsi _{(k)}\right]\right| \textrm{d}r^*\,. \end{aligned}$$

We begin by deriving important properties of the frequency parameters:

##### Lemma 6.26

(Properties of the frequency parameters in $\mathcal {F}_\textrm{comp,1}$) Fix $s\in \mathbb {Z}$ and M>0. Let a∈[0,M] and $(\omega ,m,\Lambda )\in \mathcal {F}_\textrm{comp,1}(\varepsilon _\textrm{width}, \omega _\textrm{high},r_0',\beta _3)$. Then, for sufficiently small $\varepsilon _\textrm{width}$ depending on $\beta _3$ and for sufficiently large $\omega _\textrm{high}$ depending on $\varepsilon _\textrm{width}$, fixing $r_0'$ by Lemma 6.16, we have

(i) $$\varepsilon _\textrm{width}\leqq \omega /(\omega -m\upomega _+) \leqq \varepsilon _\textrm{width}^{-1}$$;
(ii) $$\Lambda \geqq \frac{2}{3} m^2$$;
(iii) the conditions for Lemma 6.20 are satisfied; in fact, there is a $\beta _0=\beta _0(\varepsilon _\textrm{width})$ such that $\Lambda -2am\omega \geqq \beta _0\Lambda$ and hence
  $$\begin{aligned} \Lambda -2am\omega -\varepsilon _1(m^2+\omega ^2)\geqq \varepsilon _3\Lambda \,,\qquad \varepsilon _1=\beta _0\varepsilon _\textrm{width}/6, \quad \varepsilon _3= \frac{1}{2} \beta _0\,, \end{aligned}$$
  and $\Lambda \geqq \Lambda _\textrm{high}:=\varepsilon _\textrm{width}\omega _\textrm{high}^2$, where we note $(\varepsilon _1\varepsilon _3^2\Lambda _\textrm{high}^2)^{-1}\leqq B \beta _0^{-3}\varepsilon _\textrm{width}^{-3}\omega _\textrm{high}^{-4}\ll 1$ as long as $\omega _\textrm{high}$ is sufficiently large depending on $\varepsilon _\textrm{width}$.

##### Proof

For (ii), we direct the reader to the the first paragraph of the proof of Lemma 6.23.

Let us now focus on (i). Either $m\omega \leqq 0$, in which case

$$\begin{aligned} 1\geqq \frac{\omega }{\omega -m\upomega _+}=\frac{|\omega |}{|\omega |+|m|\upomega _+} \geqq \frac{1}{1+\sqrt{\frac{3}{2}}\varepsilon _\textrm{width}^{-1/2}\upomega _+}\geqq \frac{2}{M}\varepsilon _\textrm{width}^{1/2}\,, \end{aligned}$$

or $m(\omega -m\upomega _+)\geqq \beta _3\Lambda$, in which case

$$\begin{aligned} 1< \frac{\omega }{\omega -m\upomega _+}=\frac{|m\omega |}{|m(\omega -m\upomega _+)|} \leqq \frac{|m\omega |}{\beta _3\Lambda }\leqq \sqrt{\frac{3}{2}}\frac{\varepsilon _\textrm{width}^{-1/2}}{\beta _3}\leqq \varepsilon _\textrm{width}^{-1}\,, \end{aligned}$$

using the fact that we can choose $\varepsilon _\textrm{width}<\frac{2}{3} \beta _3^2$ for sufficiently small $\varepsilon _\textrm{width}$.

Turning to (iii), we note this follows easily if $m^2<(2\,M)^{-2}\varepsilon _\textrm{width}\Lambda$ or if $a\leqq \tilde{a}_0:=\varepsilon _\textrm{width}^{1/2}/2$ (see also the proof of Lemma 6.31(iii)). Hence, suppose $(2\,M)^{-2}\varepsilon _\textrm{width}\Lambda \leqq m^2\leqq \Lambda$ and $a\geqq \tilde{a}_0$. If $\Lambda -2am\omega =o(\Lambda )$, then

$$\begin{aligned} \omega ^2-\mathcal {V}_0&=\left(\omega -\frac{am}{r^2+a^2}\right)^2-\frac{\Delta (\Lambda -2am\omega )}{(r^2+a^2)^2}\geqq (\omega -m\upomega _+)^2-\frac{\Delta (\Lambda -2am\omega )}{(r^2+a^2)^2} \\&\geqq \varepsilon _\textrm{width}\Lambda -\frac{\Delta (\Lambda -2am\omega )}{(r^2+a^2)^2}\geqq \frac{1}{2} \varepsilon _\textrm{width}\Lambda \,,\quad \forall r\in [r_+,\infty )\,, \end{aligned}$$

using (i), which contradicts the assumption that $r_0'<\infty$ for $\mathcal {F}_\textrm{comp,1}$. Thus, there must exist a $\beta _0>0$ such that $\Lambda -2am\omega \geqq \beta _0\Lambda$; this constant depends on $r_0'$ and hence on $\varepsilon _\textrm{width}$. That the conditions of Lemma 6.20 are satisfied then follows from (ii) and the remaining restrictions on the frequencies in $\mathcal {F}_\textrm{comp,1}$. □

Next, we obtain a description of $\mathcal {V}$ in $\mathcal {F}_\textrm{comp,1}$:

##### Lemma 6.27

(Behavior of the potential in $\mathcal {F}_\textrm{comp,1}$) Fix $s\in \mathbb {Z}$, M>0 and $r_0'$ by Lemma 6.16. For all $\beta _3>0$, all sufficiently small $\varepsilon _\textrm{width}$ depending on $\beta _3$ and all sufficiently large $\omega _\textrm{high}$ depending on $\varepsilon _\textrm{width}$, fixing $r_0'$ by Lemma 6.16, if $(\omega ,m,\Lambda )\in \mathcal {F}_\textrm{comp,1}(\varepsilon _\textrm{width},\omega _\textrm{high},\beta _3,r_0')$, then there exists an $r_3\in (r_+,\infty )$ satisfying $|r_3-r_+|\geqq B(\varepsilon _\textrm{width})$ and $r_3\leqq B(\varepsilon _\textrm{width})$ such that

$$\begin{aligned} \mathcal {V}-\omega ^2\leqq -b(\varepsilon _\textrm{width})\Lambda \,, \quad \forall \,r\in [r_+,r_3]\,. \end{aligned}$$

and the potential $\mathcal {V}$ has a unique maximum $r_\textrm{max}\in [r_3,\infty )$ which satisfies $|r_\textrm{max}-r_+|\leqq b(\varepsilon _\textrm{width})$ and $r_\textrm{max}\leqq B(\varepsilon _\textrm{width})$.

##### Proof

By Lemma 6.16, the above conditions hold for $\mathcal {V}_0$ with $r_3$ replaced by $r_0'$ and $r_\textrm{max}$ replaced by $r_\textrm{max}^0$ To extend these conclusions to the full potential $\mathcal {V}$, we appeal to the bounds

$$\begin{aligned} \left|\mathcal {V}_1\right| \leqq Br^{-3}\,, \quad \left|\frac{\textrm{d}\mathcal {V}_1}{\textrm{d}r}\right|\leqq Br^{-4}\,,\quad \left|\frac{\textrm{d}}{\textrm{d}r}\left((r^2+a^2)^3\frac{\textrm{d}\mathcal {V}_1}{\textrm{d}r}\right)\right|\leqq Br\,, \end{aligned}$$

hence if $\omega _\textrm{high}$ is very large, Λ is as well, so $\mathcal {V}_0$ dominates. □

**Fig. 5:**
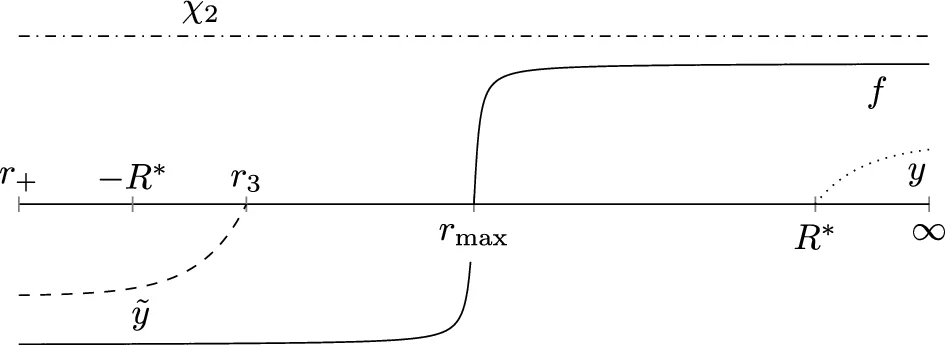
Sketch of currents in the proofs of Proposition 6.25

We are finally ready to make use of the above information to prove Proposition 6.25. We briefly describe the strategy, which is best exemplified by Fig. 5. By Lemma 6.26, $\mathcal {V}-\omega ^2$ is quantitatively negative in $[r_+,r_3]$ and $\mathcal {V}$ has a unique maximum in $[r_3,\infty )$. To exploit the former property, we can employ a *y* current in $[r_+,r_3]$ which is fast growing. To capture the latter, we construct an *f* that changes sign with $\mathcal {V}'$ as in the proof of Propositions 6.21 and 6.22. We may also add another *y* current supported near $r\rightarrow \infty$ to improve the large *r* weights. By combining these virial currents, we obtain a bulk estimate with a degeneration at $r=r_\textrm{max}$; unlike what occurs in the $\mathcal {F}_{\measuredangle }$ and , the degeneration cannot be eliminated though it can, and is, improved through a Hardy estimate. The virial currents also produce boundary terms which we must control by means of an energy estimate: since frequencies in $\mathcal {F}_\textrm{comp,1}$ are not superradiant, see also Lemma 6.26(i), we choose a global Killing *T* energy current.

For s≠0, these virial and energy currents also produce errors due to the coupling terms on the right hand side of (4.21). To control the errors from the virial currents we can simply appeal to Lemma 6.18; indeed, as we show in Lemma 6.26(iii), the elliptic estimate (6.23) can be applied. In other words, frequencies which are trapped for Ψ are *not* trapped for $\uppsi _{(k)}$ with k<|s|. Unlike in the $\mathcal {F}_{\measuredangle }$ and  regimes, however, the elliptic estimates of Lemma 6.18 alone are not enough to control the errors arising from the energy currents, due to the degeneration of our bulk estimate for Ψ at $r=r_\textrm{max}$. Instead, we perform a more delicate analysis: we first appeal to transport identities in Lemma 6.20, to reduce the order (in frequency parameters) of the energy current errors; after doing so, Lemma 6.18 provides the necessary smallness of the errors. In this last step, our Hardy estimate improvement at $r=r_\textrm{max}$ is essential.

##### Proof

Suppose $(\omega ,m,\Lambda )\in \mathcal {F}_\textrm{comp,1}$, to that is ω is large, of the same size as Λ and all frequency triples are quantitatively (depending on $\beta _3$) nonsuperradiant. We set $r_\textrm{trap}:=r_\textrm{max}$ to be the maximum of the potential, whose existence and uniqueness was established in Lemma 6.27. We employ virial currents of *f* and *y* type, as well as a *T* type Killing energy current.

*Main terms in *(4.21). For some $R^*\geqq 1$, C>0 and $\delta _H\in (0,1)$ to be fixed, set

$$\begin{aligned} f(r)=\frac{1}{\pi }\arctan (r-r_\textrm{max}),\qquad y(r)=\frac{1}{2}\left(1-\frac{(R^*)^{\delta _I}}{(r^*)^{\delta _I}}\right)\mathbb {1}_{[R^*,\infty )},\\ \hat{y}(r)=-\frac{e^{C(r_3-r)}-1}{4(e^{C(r_3-r(-R*))}-1)}\mathbb {1}_{[-R^*,r_3]}+\frac{1}{2}\left(1-\frac{(R^*)^{\delta _H}}{2|r^*|^{\delta _H}}\right)\mathbb {1}_{(-\infty ,-R^*]}, \end{aligned}$$

and note that

$$\begin{aligned} f'''(r_\textrm{max})<0\,,\qquad \frac{w}{\hat{y}'}\leqq \frac{B}{C}\mathbb {1}_{[-R^*,r_3]}+\frac{B\delta _H}{(R^*)^{\delta _H}}\mathbb {1}_{(-\infty ,-R^*]}\,. \end{aligned}$$

As in this regime $\omega \ne 0$, for sufficiently large $R^*$ depending on $\varepsilon _\textrm{width}$, $\delta _H$ and $\delta _I$,

$$\begin{aligned}  &   y'\omega ^2-(y\mathcal {V})'\geqq y'\omega ^2\left[1-\frac{\varepsilon _\textrm{width}^{-1}}{r}\right]\geqq \frac{1}{2} y'\omega ^2, \\  &   \quad \left(\hat{y}'(\omega ^2-\mathcal {V})-\hat{y}\mathcal {V}'\right) \mathbb {1}_{(-\infty ,-R^*]}\\  &   \quad \geqq \hat{y}'(\omega ^2-\mathcal {V})\left(1+\frac{B(\varepsilon _\textrm{width})}{(R^*)^{\delta _H}(R^*)^{1-\delta _H}}\right)\mathbb {1}_{(-\infty ,-R^*]} \geqq \frac{1}{2} \hat{y}'(\omega ^2-\mathcal {V})\mathbb {1}_{(-\infty ,-R^*]}; \end{aligned}$$

we fix $R^*$ so that the above hold. Next, by Lemma 6.27, we have

6.33 $$\begin{aligned} -f\mathcal {V}'\mathbb {1}_{[r_3,\infty )} \geqq b(\varepsilon _\textrm{width})\Lambda \frac{(r-r_\textrm{max})^2\Delta }{r^7}\mathbb {1}_{[r_3,\infty )}\,, \end{aligned}$$

and thus, for sufficiently large $\omega _\textrm{high}$ depending on $\varepsilon _\textrm{width}$,

$$\begin{aligned} -f\mathcal {V}' -\frac{1}{2} f'''\geqq \left(-f\mathcal {V}'-\frac{1}{2} f'''\right)\mathbb {1}_{[r_+,r_3]}-\frac{1}{2}f\mathcal {V}'\,. \end{aligned}$$

At the same time, we have

$$\begin{aligned} \hat{y}' (\omega ^2-\mathcal {V})\mathbb {1}_{[-R^*,r_3]}\geqq C b(\varepsilon _\textrm{width})\Lambda \frac{\Delta }{r^2} \mathbb {1}_{[-R^*,r_3]}\,, \end{aligned}$$

and so for sufficiently large *C* depending on $\varepsilon _\textrm{width}$, we obtain

$$\begin{aligned} \left(\hat{y}' (\omega ^2-\mathcal {V}) -\hat{y} \mathcal {V}' \right)\mathbb {1}_{[-R^*,r_3]} \geqq \frac{3}{4}\hat{y}' (\omega ^2-\mathcal {V})\mathbb {1}_{[-R^*,r_3]}\,, \end{aligned}$$

Combining with the previous estimates, we deduce that for even larger *C* depending on $\varepsilon _\textrm{width}$,

$$\begin{aligned} -f\mathcal {V}' -\frac{1}{2} f'''+ \left(\frac{1}{2}+\frac{1}{4}\mathbb {1}_{[-R^*,r_3]}\right)\hat{y}' (\omega ^2-\mathcal {V}) \geqq \frac{1}{2}\hat{y}' (\omega ^2-\mathcal {V}) -\frac{1}{2}f\mathcal {V}'\,. \end{aligned}$$

The estimates above and (5.3) imply that $Q^y+Q^{\hat{y}}+Q^f$ produce a good bulk term from the point of view of the left hand side of (4.21); however, they also produce boundary terms with the wrong sign. To control these, in view of the absence of superradiance from Lemma 6.26(i), we apply a global current $E Q^T$ for some $E\geqq 2\varepsilon _\textrm{width}^{-1}+1$. Thus, we have

6.34 $$\begin{aligned}&(\omega -m\upomega _+)^2|\Psi (-\infty )|^2+\omega ^2|\Psi (+\infty )|^2\nonumber \\&\qquad +\int _{-\infty }^\infty \left[(2f'+\hat{y}' + y')|\Psi '|^2+\left(y'\omega ^2+\frac{1}{2}\hat{y}' (\omega ^2-\mathcal {V}) -\frac{1}{2}f\mathcal {V}'\right)|\Psi |^2\right]\textrm{d}r^*\nonumber \\&\quad \leqq \sum _{k=0}^{|s|-1}\int _{-\infty }^\infty \left\rbrace 2w(\hat{y}+f+y) \operatorname {Re}[(ac_{s,k}^\textrm{id}+im ac_{s,k}^{\Phi })\overline{\Psi }']\right.\nonumber \\&\qquad \qquad \qquad \qquad \left.+wf'\operatorname {Re}[(ac_{s,k}^\textrm{id}+im ac_{s,k}^{\Phi })\overline{\Psi }]\right\lbrace \textrm{d}r^* \nonumber \\&\qquad - \sum _{k=0}^{|s|-1}\int _{-\infty }^\infty Ew\omega \operatorname {Im}[(ac_{s,k}^\textrm{id}+im ac_{s,k}^{\Phi })\overline{\Psi }]\textrm{d}r^*\nonumber \\&\qquad + \int _{-\infty }^\infty \left\rbrace 2(\hat{y}+y+f)\operatorname {Re}[\mathfrak {G}\overline{\Psi }']-E\omega \operatorname {Im}[\mathfrak {G}\overline{\Psi }]\right\lbrace \textrm{d}r^*\,. \end{aligned}$$

In (6.34), the degeneration at $r=r_\textrm{max}$ in (6.33) can be improved. For instance,

$$\begin{aligned}&\int _{-\infty }^\infty \frac{w(r-M)}{r^{3/2}}\sqrt{\Lambda }|\Psi |\textrm{d}r^* \\&\quad \leqq B\left| \int _{-\infty }^\infty \frac{\textrm{d}}{\textrm{d}r^*}\left(\frac{(r-r_\textrm{max})\Delta }{r^{11/2}}\sqrt{\Lambda }\right)|\Psi |^2\textrm{d}r^*\right|\\&\quad = B\int _{-\infty }^\infty \frac{2(r-r_\textrm{max})\Delta }{r^{11/2}}\sqrt{\Lambda }\operatorname {Re}[\Psi '\overline{\Psi }]\textrm{d}r^*\\&\quad \leqq B(\varepsilon _\textrm{width})\int _{-\infty }^\infty \left[\frac{1}{2}(2f'+\hat{y}' + y')|\Psi '|^2-f\mathcal {V}'|\Psi |\right]\textrm{d}r^* \end{aligned}$$

and the fact that $r_\textrm{max}-r_+\geqq b(\varepsilon _\textrm{width})$ shows that in fact we can replace (6.34) by

6.35 $$\begin{aligned}&(\omega -m\upomega _+)^2|\Psi (-\infty )|^2+\omega ^2|\Psi (+\infty )|^2 \nonumber \\&\qquad +\int _{-\infty }^\infty \left[\frac{1}{2}(2f'+\hat{y}' + y')|\Psi '|^2\right.\nonumber \\&\left.\quad +\left(y'\omega ^2+\frac{1}{2}\hat{y}' (\omega ^2-\mathcal {V}) +b(\varepsilon _\textrm{width})\frac{w}{r^{1/2}}(\sqrt{\Lambda }+|\omega |) -\frac{1}{4}f\mathcal {V}'\right)|\Psi |^2\right]\textrm{d}r^* \nonumber \\&\quad \leqq -\sum _{k=0}^{|s|-1}\int _{-\infty }^\infty \Bigg \{2w(\hat{y}+f+y) \operatorname {Re}[(ac_{s,k}^\textrm{id}+im ac_{s,k}^{\Phi })\uppsi _{(k)}\overline{\Psi }']\nonumber \\&\quad \qquad \qquad \qquad \qquad \qquad +wf'\operatorname {Re}[(ac_{s,k}^\textrm{id}+im ac_{s,k}^{\Phi })\uppsi _{(k)}\overline{\Psi }]\Bigg \}\textrm{d}r^* \nonumber \\&\quad \qquad + \sum _{k=0}^{|s|-1}\int _{-\infty }^\infty Ew\omega \operatorname {Im}[(ac_{s,k}^\textrm{id}+im ac_{s,k}^{\Phi })\uppsi _{(k)}\overline{\Psi }]\textrm{d}r^* \nonumber \\&\quad - \int _{-\infty }^\infty \left\rbrace 2(\hat{y}+y+f)\operatorname {Re}[\mathfrak {G}\overline{\Psi }']-E\omega \operatorname {Im}[\mathfrak {G}\overline{\Psi }]\right\lbrace \textrm{d}r^*\,. \end{aligned}$$

If s=0, the first two integrals on the right hand side of (6.35) are absent and so the proof is finished.

*The coupling terms in* (4.21). Let us now assume s≠0. For the second integral on the right hand side of (6.35), we appeal to the transport estimate of Lemma 6.20, in which we let $\varepsilon \ll E^{-1}$ depending on $\varepsilon _\textrm{width}$ and 1-χ to be a smoothing of $(r-r_\textrm{max})$. We deduce that, for some ϵ>0, the first two integrals on the right hand side of (6.35) can be controlled by

$$\begin{aligned}&\int _{-\infty }^\infty \left\rbrace \epsilon (2f'+y'+\hat{y}')|\Psi '|^2+\epsilon \left(\frac{1}{2}\hat{y}' (\omega ^2-\mathcal {V})-\frac{1}{4} f\mathcal {V}'\right)|\Psi |^2\right\lbrace \textrm{d}r^*\\&\quad + E \epsilon ^{-1} B(\varepsilon _\textrm{width})\sum _{k=0}^{|s|-1}\int _{-\infty }^\infty w\Lambda |\uppsi _{(k)}|^2\textrm{d}r^*\,, \end{aligned}$$

as long as $\omega _\textrm{high}^2, \varepsilon _\textrm{width}\omega ^2_\textrm{high}\geqq 1$. We now fix this ϵ to be sufficiently small so that the first term can be absorbed by the left hand side of (6.35). For the second term, we appeal to our improved estimate (6.23) in Lemma 6.18:

$$\begin{aligned}&E B(\varepsilon _\textrm{width})\sum _{k=0}^{|s|-1}\int _{-\infty }^\infty w\Lambda |\uppsi _{(k)}|^2\textrm{d}r^*\\&\quad \leqq \int _{-\infty }^\infty \frac{B(E,\varepsilon _\textrm{width})}{\omega _\textrm{high}}w|\omega ||\Psi |^2 + B(E,\varepsilon _\textrm{with})\sum _{k=0}^{|s|-1}\int _{-\infty }^\infty |\operatorname {Re}[\mathfrak {G}_{(k)}\overline{\uppsi _{(k)}}]|\textrm{d}r^*\,. \end{aligned}$$

Here, the first term can also be absorbed into the left hand side of (6.35) as long as $\omega _\textrm{high}$ is sufficiently large depending on *E* and $\varepsilon _\textrm{width}$. □

#### Time dominated regime and comparable, non-superradiant and non-trapped regime

This section concerns the frequency ranges  and $\mathcal {F}_\textrm{comp,2}$. We will prove the following two propositions:

##### Proposition 6.28

(Estimates in ) Fix $s\in \mathbb {Z}$ and M>0. Then, for all $\delta _I,\delta _H\in (0,1]$, for all E>0 sufficiently large, for all $\varepsilon _\textrm{width}$ sufficiently small, for all $\omega _\textrm{high}$, and for all , there exist functions y≧0 and $\hat{y}\geqq 0$ satisfying the bounds (6.3)

$$\begin{aligned}&\omega ^2\left|\Psi (\infty )\right|^2+(\omega -m\upomega _+)^2\left|\Psi (-\infty )\right|^2 \\&\qquad + b(\delta _H,\delta _I)\int _{-\infty }^\infty \frac{\Delta }{r^{3+\delta _I}}\left(1-\frac{M}{r}\right)^{\delta _H-1}\Big [|\Psi '|^2+\omega ^2|\Psi |^2\Big ]\textrm{d}r^*\\&\quad \leqq -\int _{-\infty }^\infty \left\rbrace 2(y+\hat{y})\operatorname {Re}\left[\mathfrak {G}\overline{\Psi }'\right]-E\omega \operatorname {Im}\left[\mathfrak {G}\overline{\Psi }\right]\right\lbrace \textrm{d}r^*\\&\qquad -\int _{-\infty }^\infty \sum _{k=0}^{|s|-1}2(y\mathbb {1}_{\{s>0\}}+\hat{y}\mathbb {1}_{\{s<0\}})\operatorname {Re}\left[\mathfrak {G}_{(k)}\overline{\uppsi _{(k)}}'\right]\textrm{d}r^*\,. \end{aligned}$$

We may drop the terms on the right hand side with k≠|s| if $|a|\leqq \tilde{a}_0$ is sufficiently small depending on $\varepsilon _\textrm{width}$ or, adding by a dependence on *m* in the parameters and constants in the estimate, if *m* is fixed.

##### Proposition 6.29

(Estimates in $\mathcal {F}_\textrm{comp,2}$) Fix $s\in \mathbb {Z}$ and M>0. Then, for all $\beta _3>0$, for all $\varepsilon _\textrm{width}$ sufficiently small depending on $\beta _3$ that in particular $\beta _3\geqq \sqrt{\varepsilon _\textrm{width}}$, for all E>0 sufficiently large depending on $\varepsilon _\textrm{width}$, for all $\omega _\textrm{high}$ sufficiently large depending on $\varepsilon _\textrm{width}$, and for all $(a,\omega ,m,\Lambda ) \in \mathcal {F}_\textrm{comp,2}(\omega _\textrm{high},\varepsilon _\textrm{width},r_0',\beta _3)$ where $r_0'$ is fixed by Lemma 6.16, there exist functions y≧0 and $\hat{y}\geqq 0$ satisfying the bounds (6.3) such that

$$\begin{aligned}&\omega ^2\left|\Psi (\infty )\right|^2+(\omega -m\upomega _+)^2\left|\Psi (-\infty )\right|^2 + b(\delta _H,\delta _I,\varepsilon _\textrm{width})\\&\int _{-\infty }^\infty \frac{\Delta }{r^{3+\delta _I}}\left(1-\frac{M}{r}\right)^{\delta _H-1}\Big [|\Psi '|^2+\omega ^2|\Psi |^2\Big ]\textrm{d}r^*\\&\quad \leqq -\int _{-\infty }^\infty \left\rbrace 2(y+\hat{y})\operatorname {Re}\left[\mathfrak {G}\overline{\Psi }'\right]-E\omega \operatorname {Im}\left[\mathfrak {G}\overline{\Psi }\right]\right\lbrace \textrm{d}r^*\\&\qquad -\int _{-\infty }^\infty \sum _{k=0}^{|s|-1}2(y\mathbb {1}_{\{s>0\}}+\hat{y}\mathbb {1}_{\{s<0\}})\operatorname {Re}\left[\mathfrak {G}_{(k)}\overline{\uppsi _{(k)}}'\right]\textrm{d}r^*\,. \end{aligned}$$

We may drop the terms on the right hand side with k≠|s| if $|a|\leqq \tilde{a}_0$ is sufficiently small depending on $\varepsilon _\textrm{width}$ or, adding by a dependence on *m* in the parameters and constants in the estimate, if *m* is fixed.

We begin, in the next two lemmas, by deriving important properties of the frequency parameters in the relevant frequency regimes.

##### Lemma 6.30

(Properties of ) Fix $s\in \mathbb {Z}$ and M>0. Let  be an admissible frequency triple with respect to a∈[0,M]. Then, for sufficiently small $\varepsilon _\textrm{width}$ and sufficiently large $\omega _\textrm{high}$, we have

(i) $$m^2,|\Lambda |\leqq 2\varepsilon _\textrm{width}\omega ^2$$;
(ii) $$2\omega ^2\geqq \omega (\omega -m\upomega _+)\geqq \frac{1}{2} \omega ^2$$.

##### Proof

By property IV(b) in Definition 4.1, we obtain statement (i):

$$\begin{aligned} m^2&\leqq \Lambda +2|s||a\omega |+s^2 \leqq 2\varepsilon _\textrm{width}\omega ^2\,,\\ |\Lambda |&\leqq \max \{-\Lambda ,\Lambda \} \leqq \max \{|s|(2M|\omega |-1),\varepsilon _\textrm{width}\omega ^2\}\leqq 2\varepsilon _\textrm{width}\omega ^2\,, \end{aligned}$$

for sufficiently small $\varepsilon _\textrm{width}$ and sufficiently large $\omega _\textrm{high}$. From these bounds, we obtain (ii). □

##### Lemma 6.31

(Properties of $\mathcal {F}_\textrm{comp,2}$) Fix $s\in \mathbb {Z}$, M>0 and $\beta _3>0$. Let $r_0'$ be defined by Lemma 6.16 and $\beta _3>0$. Let $(\omega ,m,\Lambda )\in \mathcal {F}_\textrm{comp,2}(\varepsilon _\textrm{width}, \omega _\textrm{high},r_0',\beta _3)$ be an admissible frequency triple with respect to a∈[0,M]. Then, for sufficiently small $\varepsilon _\textrm{width}$ depending on $\beta _3$, and sufficiently large $\omega _\textrm{high}$, in the case of $\mathcal {F}_\textrm{comp,2}$ depending on $\varepsilon _\textrm{width}$,

(i) $$\Lambda \geqq \max \{\frac{a_0^2}{M^2},\frac{2}{3}\}m^2$$;
(ii) $$\Lambda \geqq \varepsilon _\textrm{width}^{1/2}|m\omega |$$;
(iii) in $\mathcal {F}_\mathrm{comp,\,2a}(\varepsilon _\textrm{width}, \omega _\textrm{high},r_0',\beta _3,\beta _4)$, if $\omega _\textrm{high}$ is sufficiently large depending also on $\beta _4$, the conditions for Lemma 6.18 hold, namely, we have
  $$\begin{aligned} \Lambda -2am\omega -\varepsilon _1(m^2+\omega ^2)\geqq \varepsilon _3\Lambda \,, \qquad \varepsilon _1 =\frac{1}{3} \varepsilon _\textrm{width}\beta _4, \quad \varepsilon _1=\frac{1}{8}\,, \end{aligned}$$
  where we note that $\beta _4\varepsilon _\textrm{width}^3\omega _\textrm{high}^4\ll 1$ as long as $\omega _\textrm{high}^2$ is sufficiently large depending on $\varepsilon _\textrm{width}$ and $\beta _4$;
iv $$2\varepsilon _\textrm{width}^{1/2}\omega ^2/M\leqq \omega (\omega -m\upomega _+)\leqq \varepsilon _\textrm{width}^{-1} \omega ^2$$ and $$|m/(\omega -m\upomega _+)|\leqq \frac{2}{3} \varepsilon _\textrm{width}^{-1}$$.

##### Proof

For (i), we direct the reader to the the first paragraph of the proof of Lemma 6.23. The proof of (ii) and (iii) then follows easily. For (iv), one repeats the proof of Lemma 6.26(i). □

Next, we obtain a description of $\mathcal {V}$ in the relevant frequency ranges:

##### Lemma 6.32

(Properties of $\mathcal {V}$ in  and $\mathcal {F}_\textrm{comp,2}$) Let  or $(\omega ,m,\Lambda )\in \mathcal {F}_\textrm{comp,2}(\omega _\textrm{high},\varepsilon _\textrm{width},r_0',\beta _3)$ be an admissible frequency triple. Then, for sufficiently small $\varepsilon _\textrm{width}$ depending on $\beta _3$ and sufficiently large $\omega _\textrm{high}$, in the case of $\mathcal {F}_\textrm{comp,2}$ depending on $\varepsilon _\textrm{width}$, we have6.36

6.37 $$\begin{aligned}&\omega ^2-\mathcal {V}\geqq b(\varepsilon _\textrm{width})\omega ^2\,,\quad \mathcal {V}\leqq b(\varepsilon _\textrm{width})\frac{\omega ^2}{r^2}\,,\quad  &   |\mathcal {V}'|\leqq b(\varepsilon _\textrm{width})\frac{\omega ^2\Delta }{r^5}, \text {in} \mathcal {F}_\textrm{comp,2}. \end{aligned}$$

##### Proof

In both cases, if $\omega _\textrm{high}$ is sufficiently large, for $\mathcal {F}_\textrm{comp,2}$ depending on $\varepsilon _\textrm{width}$, the contribution from $\mathcal {V}_1$ can be neglected, so one needs only study $\mathcal {V}_0$. In , the results follow from direct computations with (4.22), (6.14) and Lemma 6.30. In $\mathcal {F}_\textrm{comp,2}$, we invoke the latter together with Lemma 6.16. □

**Fig. 6:**
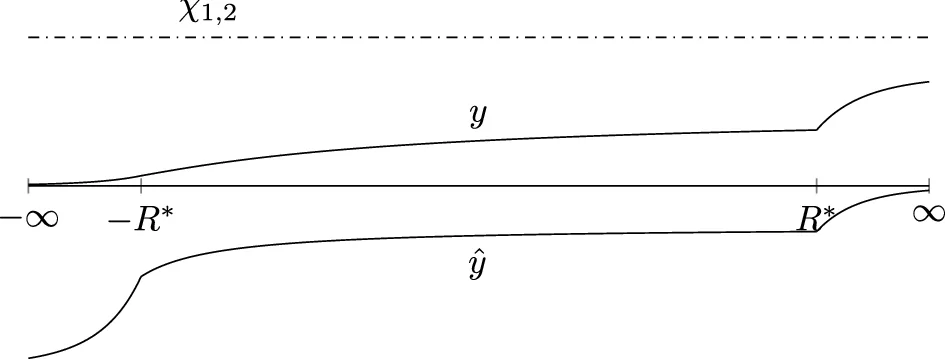
Sketch of currents in the proofs of Propositions 6.28 and 6.29

We briefly describe our strategy to prove Propositions 6.28 and 6.29, which is best illustrated by Fig. 6. Lemma 6.32 has shown that, in both the time dominated and the large, comparable non-trapped frequencies, $$\omega ^2-\mathcal {V}$$ is globally positive and large. Virial currents of *y*-type can exploit this, but they also generate a term $$y\mathcal {V}'|\Psi |^2$$ of indeterminate sign, see (5.3). To show that $$|y\mathcal {V}'|\ll \omega ^2-\mathcal {V}$$, in  we appeal to the fact that $$\Lambda \ll \omega ^2$$, but in $$\mathcal {F}_\textrm{comp,2}$$ we instead design *y* so that $|y/y^{\prime }|$ on a compact region is sufficiently small. Since *y*-type currents produce bad boundary terms, we need to add energy currents to control them; by Lemmas 6.30(ii) and 6.31(iv) these frequency regimes are non-superradiant, so we add a global *T*-type Killing current to control the boundary terms.

For s≠0, the separated currents produce errors due to the coupling terms in (4.21), the most worrying being those which arise due to $m\uppsi _{(k)}$ in (4.21). If the elliptic estimates of Lemma 6.18 were available, as is the case for instance in $\mathcal {F}_\textrm{comp,2a}$ and was the case in the previously considered high frequency regimes, we would have $m^2|\uppsi _{(k)}|^2\sim |\Psi |^2 \ll \omega ^2| \Psi |^2$ so that the strength of our bulk estimate for Ψ would be sufficient to conclude. In general in $\mathcal {F}_\textrm{comp,2}$ and , as we are not guaranteed the conditions of the improved estimates of Lemma 6.18, our proof is more complicated. We notice that, for all k=0,⋯,|s|, the potential $\mathcal {V}_{(k)}$ enjoys the properties of Lemma 6.32 so we may apply *y* currents (which we can choose so as not to produce boundary terms, if we so wish) to the k<|s| radial ODEs (4.19) as well. The resulting bulk terms for k<|s| couple to the k=|s| bulk term only through $|\Psi |^2\ll (\omega ^2-\mathcal {V})|\Psi |^2$, which gives us the missing smallness in our analysis.

##### Proof of Propositions 6.28 and 6.29

Let $(\omega ,m,\Lambda )$ be any admissible frequency triple with respect to *s* and a∈[0,M] such that . In both frequency ranges, we will employ *y*-type virial and *T*-type Killing currents.

*The main terms in *(4.21). For $R^*\geqq 1$ and C≧1 to be fixed, set

$$\begin{aligned} y(r^*)&:=\exp \left(-C\int _{r(r^*)}^\infty \frac{\textrm{d}r}{\Delta } \right)\mathbb {1}_{[-R^*,R^*]}+\frac{y(-R^*)(R^*)^{\delta _H}}{(-r^*)^{\delta _H}}\mathbb {1}_{(-\infty ,-R^*)}\\&\quad +y(R^*)\left(2-\frac{R^*}{r^*}\right)\mathbb {1}_{(R^*,\infty )}\,,\\ \hat{y}(r^*)&:=-\exp \left(-C(r-r_+)\right)\mathbb {1}_{[-R^*,R^*]}+\hat{y}(-R^*)\left(2-\frac{(R^*)^{\delta _H}}{(-r^*)^{\delta _H}}\right)\mathbb {1}_{(-\infty ,-R^*)}\\&\quad +\frac{\hat{y}(R^*)R^*}{r^*}\mathbb {1}_{(R^*,\infty )}\,. \end{aligned}$$

With these definitions, for sufficiently large $R^*$,

6.38 $$\begin{aligned} \left\rbrace \left|\frac{wy}{y'}\right|,\left|\frac{w\hat{y}}{\hat{y}'}\right|\right\lbrace&\leqq \frac{B}{\min \{R^*, C\}}\,,\qquad \left\rbrace \left|\frac{w}{y'}\right|,\left|\frac{w}{\hat{y}'}\right|\right\lbrace \leqq \frac{B}{\min \{\hat{y} (R^*),y(-R^*)\}}\,. \end{aligned}$$

In the time dominated frequencies, , taking C=1 we have

$$\begin{aligned}&y'\omega ^2-(y\mathcal {V})'\\&\quad \geqq y'\omega ^2\left[1-\frac{B\varepsilon _\textrm{width}^{1/2}}{r^2}-\frac{B\varepsilon _\textrm{width}^{1/2}\Delta }{r^3(r^2+a^2)}\left( |r^*|\mathbb {1}_{(-\infty ,-R^*]}+(r^2+a^2)\mathbb {1}_{[-R^*,\infty )}\right)\right]\\&\quad \geqq \frac{1}{2} y'\omega ^2\,,\\&\hat{y}'\omega ^2-(\hat{y}\mathcal {V})'\geqq \frac{1}{2} \hat{y}'\omega ^2\,, \end{aligned}$$

by Lemma 6.32, for sufficiently small $\varepsilon _\textrm{width}$ and sufficiently large $\omega _\textrm{high}$. In the non-superradiant and non-trapped frequencies $\mathcal {F}_\textrm{comp, 2}$, again using Lemma 6.32, we have

$$\begin{aligned} y'(\omega ^2-\mathcal {V})-y\mathcal {V}'&\geqq y'(\omega ^2-\mathcal {V})\left(1-\frac{B(\varepsilon _\textrm{width})\Delta }{C r^3}\mathbb {1}_{[-R^*,R^*]}-\frac{B(\varepsilon _\textrm{width})}{R^*}\mathbb {1}_{[-R^*,R^*]^c}\right)\\&\geqq \frac{1}{2} y'(\omega ^2-\mathcal {V})\,,\\ \hat{y}'(\omega ^2-\mathcal {V})-\hat{y}\mathcal {V}'&\geqq \frac{1}{2} \hat{y}'(\omega ^2-\mathcal {V})\,, \end{aligned}$$

as long as *C* and $R^*$ are sufficiently large depending on $\varepsilon _\textrm{width}$ and $R^*$ is sufficiently large depending on $\delta _H$. Thus, noting (5.3), we deduce that both $Q^y$ and $Q^{\hat{y}}$ generate good bulk terms for Ψ when applied to the left hand side of (4.21).

On the other hand, since $y(\infty )=2y(R^*)>0$, y(-∞)=0, $\hat{y}(\infty )=0$ and $\hat{y}(-\infty )=2\hat{y}(-R^*)<0$, these currents generate bad boundary terms. By Lemmas 6.30 and 6.31, we can apply a global $EQ^T$ current. Choosing $E\geqq \left\rbrace 2y(\infty )+1,4\hat{y}(-\infty )+2\right\lbrace$ in  and $E\geqq \left\rbrace 2y(\infty )+1,M\varepsilon _\textrm{width}^{-1/2}(\hat{y}(-\infty )+1/2)\right\lbrace$ in $\mathcal {F}_\textrm{comp,2}$, with the two alternatives corresponding to applications of $Q^y$ alone and $Q^{\hat{y}}$ alone, respectively, we have

$$\begin{aligned}&\left(2\hat{y}(-\infty )(\omega -m\upomega _+)^2-E\omega (\omega -m\upomega _+)\right)|\Psi (-\infty )|^2\\&\qquad + \left(-2 y(\infty )\omega ^2-E\omega ^2\right)|\Psi (+\infty )|^2 \\&\quad \leqq -(\omega -m\upomega _+)^2|\Psi (-\infty )|^2-\omega ^2|\Psi (+\infty )|^2\,, \end{aligned}$$

thus obtaining good boundary terms. We end up with the estimate

6.39 $$\begin{aligned}&(\omega -m\upomega _+)^2|\Psi (-\infty )|^2+\omega ^2|\Psi (+\infty )|^2\nonumber \\&\qquad + \int _{-\infty }^\infty \{y',\hat{y}'\}\left(|\Psi '|^2+\frac{1}{2}(\omega ^2-\mathcal {V})|\Psi |^2\right)\textrm{d}r^*\nonumber \\&\quad \leqq -\sum _{k=0}^{|s|-1}\int _{-\infty }^\infty \left\rbrace 2aw\{y,\hat{y}\}\operatorname {Re}[\Psi '(c_{s,j}^\textrm{id}+imc_{s,k}^{\Phi })\overline{\uppsi }_{(k)}]\right.\nonumber \\&\qquad \qquad \qquad \left.\qquad \qquad -Ea\omega w\operatorname {Im}[\Psi (c_{s,j}^\textrm{id}+imc_{s,k}^{\Phi })\overline{\uppsi }_{(k)}]\right\lbrace \textrm{d}r^*\nonumber \\&\qquad -\int _{-\infty }^\infty \left(2\{y,\hat{y}\}\operatorname {Re}[\mathfrak G\overline{\Psi }']-E\operatorname {Im}[\mathfrak {G}\overline{\Psi }]\right)\textrm{d}r^*\,, \end{aligned}$$

for sufficiently large $R^*$ and, in $\mathcal {F}_\textrm{comp, 2}$, sufficiently large *C*. Now, we fix $R^*$ and *C* so that the above holds, and rescale *y* and $\hat{y}$ so that y(∞)=1 and $-\hat{y}(-\infty )=1$. If s=0, adding the two alternative estimates given by the curly brackets concludes the proof.

*The coupling terms in* (4.21). From now on, assume s≠0, and let *B* denote a constant depending not just on *M* but possibly also on $\delta _{H}$. We want to estimate the first term on the right hand side of (6.39): for some ϵ>0, using (6.38),

6.40 $$\begin{aligned}&\int _{-\infty }^\infty \left\rbrace 2aw\{y,\hat{y}\}\operatorname {Re}[\overline{\Psi }'(c_{s,j}^\textrm{id}+imc_{s,k}^{\Phi })\uppsi _{(k)}]-Ea\omega w\operatorname {Im}[\overline{\Psi }(c_{s,j}^\textrm{id}+imc_{s,k}^{\Phi })\uppsi _{(k)}]\right\lbrace \textrm{d}r^*\nonumber \\&\quad \leqq B\int _{-\infty }^\infty \{y',\hat{y}'\}\left(\epsilon |\Psi '|^2+\epsilon (\omega ^2-\mathcal {V})|\Psi |^2\right.\nonumber \\&\qquad \qquad \qquad \left.+\frac{E^2a^2\epsilon ^{-1}\omega ^2(1+m^2)}{(\omega ^2-\mathcal {V})(\omega ^2-\mathcal {V}_{(k)})}(\omega ^2-\mathcal {V}_{(k)})|\uppsi _{(k)}|^2\right)\textrm{d}r^*\,. \end{aligned}$$

The first term in the right hand side of (6.40) can be absorbed into the left hand side of (6.39) if ϵ is sufficiently small; we fix it so that this holds. We are left with the second term in the right hand side of (6.40). Note that, by Lemma 6.32,Moreover, by the basic transport estimate of Lemma 5.5,

6.41 $$\begin{aligned} \int _{-\infty }^{\infty }\{y',\hat{y}'\}|\uppsi _{(k)}|^2\textrm{d}r^*&\leqq B\{|\uppsi _{(k)}(\infty )|^2,|\uppsi _{(k)}(-\infty )|^2\}\nonumber \\&\quad + B\int _{-\infty }^\infty \{y',\hat{y}'\}|\uppsi _{(k+1)}|^2\textrm{d}r^*\,. \end{aligned}$$

In the case $\mathcal {F}_\textrm{comp,2a}$, we can make use of the improved estimate of Lemma 6.18 to obtain

$$\begin{aligned}&\sum _{k=0}^{|s|-1}\int _{-\infty }^\infty \frac{E^2a^2\omega ^2}{(\omega ^2-\mathcal {V})}\{y',\hat{y}'\}(1+m^2)|\uppsi _{(k)}|^2\textrm{d}r^* \\&\quad \leqq B(\varepsilon _\textrm{width},\beta _4)E^2\int _{-\infty }^\infty \{y',\hat{y}'\}\omega _\textrm{high}^2(\omega ^2-\mathcal {V})|\Psi |^2\textrm{d}r^*\\&\qquad + BE^2\int _{-\infty }^\infty \sum _{k=0}^{|s|}\operatorname {Re}[\mathfrak {G}_{(k)}\overline{\uppsi _{(k)}}]\textrm{d}r^*\,. \end{aligned}$$

Thus, if $\omega _\textrm{high}$ is sufficiently large depending on *E*, $\varepsilon _\textrm{width}$ and $\beta _4$, we can absorb the last term into the left hand side of (6.39). Alternatively, if $a\leqq a_0$, we can obtain from (6.41) the estimate

$$\begin{aligned}&\sum _{k=0}^{|s|-1}\int _{-\infty }^\infty \frac{E^2a^2\omega ^2}{(\omega ^2-\mathcal {V})}\{y',\hat{y}'\}(1+m^2)|\uppsi _{(k)}|^2\textrm{d}r^* \\&\quad \leqq a_0^2B(\varepsilon _\textrm{width})\sum _{k=0}^{|s|-1}E^2(1+m^2)\{y(\infty )|\uppsi _{(k)}(\infty )|^2,-\hat{y}(-\infty )|\uppsi _{(k)}(-\infty )|^2\} \\&\quad \qquad + a_0^2B(\varepsilon _\textrm{width})E^2\int _{-\infty }^\infty \{y',\hat{y}'\}(\omega ^2-\mathcal {V})|\Psi |^2\textrm{d}r^*\,. \end{aligned}$$

Making $a_0$ sufficiently small depending on $\varepsilon _\textrm{width}$ implies that, after using (6.41), we can absorb the resulting errors into the left hand side of (6.39) as well. Assuming outgoing boundary conditions, the boundary terms vanish if we choose to work solely with $Q^y$ for s>0 and with $Q^{\hat{y}}$ for s<0.

For general a≦M and , neither does Lemma 6.18 hold nor do we have smallness for *a*. Therefore, we find it necessary to add an extra step: noting that $\mathcal {V}_{(k)}$ share the properties of $\mathcal {V}$ outlined in Lemma 6.32, we may apply $Q^{y}$, if s>0, and $Q^{\hat{y}}$, if s<0, currents to each of the radial ODEs (4.19), to arrive at

$$\begin{aligned}&\int _{-\infty }^\infty (y'\mathbb {1}_{\{s>0\}}+\hat{y}'\mathbb {1}_{\{s<0\}})\left(|\uppsi _{(k)}'|^2+\frac{1}{2}(\omega ^2-\mathcal {V}_{(k)})|\uppsi _{(k)}|^2\right)\textrm{d}r^*\\&\quad \leqq B(\varepsilon _\textrm{width})\int _{-\infty }^\infty (y'\mathbb {1}_{\{s>0\}}+\hat{y}'\mathbb {1}_{\{s<0\}})\Big (\sum _{j=0}^{k-1}(\omega ^2-\mathcal {V}_{(j)})|\uppsi _{(j)}|^2+\epsilon (\omega ^2-\mathcal {V}_{(k)})|\uppsi _{(k)}|^2\Big )\textrm{d}r^*\\&\quad \qquad +B(\varepsilon _\textrm{width})\int _{-\infty }^\infty (y'\mathbb {1}_{\{s>0\}}+\hat{y}'\mathbb {1}_{\{s<0\}})\epsilon ^{-1} \varepsilon _\textrm{width}^{-1}\omega _\textrm{high}^{-2}(\omega ^2-\mathcal {V}_{(k+1)})|\uppsi _{(k+1)}|^2 \textrm{d}r^*\\&\quad \qquad -\int _{-\infty }^\infty 2 (y\mathbb {1}_{\{s>0\}}+\hat{y}\mathbb {1}_{\{s<0\}})\operatorname {Re}[\mathfrak G_{(k)}\overline{\uppsi }_{(k)}']\textrm{d}r^*\, \end{aligned}$$

for k<|s|, where we have used Lemma 5.1, the strategy of (6.40), the fact that $\uppsi _{(k)}$ have outgoing boundary conditions and Lemma 6.31. We fix ϵ depending on $\varepsilon _\textrm{width}$ so that the term in $|\uppsi _{(k)}|^2$ can be absorbed into the left hand side. Thus, if $\omega _\textrm{high}$ is sufficiently large depending on $\varepsilon _\textrm{width}$, we can iterate the estimate as,

$$\begin{aligned}&\int _{-\infty }^\infty (y'\mathbb {1}_{\{s>0\}}+\hat{y}'\mathbb {1}_{\{s<0\}})\left(|\uppsi _{(k)}'|^2+\frac{1}{4}(\omega ^2-\mathcal {V}_{(k)})|\uppsi _{(k)}|^2\right)\textrm{d}r^*\\&\quad \leqq B(\varepsilon _\textrm{width})\int _{-\infty }^\infty (y'\mathbb {1}_{\{s>0\}}+\hat{y}'\mathbb {1}_{\{s<0\}})\nonumber \\&\quad \Big (\sum _{j=0}^{k-1}(\omega ^2-\mathcal {V}_{(j)})|\uppsi _{(j)}|^2+ \varepsilon _\textrm{width}^{-1}\omega _\textrm{high}^{-2}(\omega ^2-\mathcal {V}_{(k+1)})|\uppsi _{(k+1)}|^2 \Big ) \textrm{d}r^*\\&\quad \qquad -\int _{-\infty }^\infty 2 (y\mathbb {1}_{\{s>0\}}+\hat{y}\mathbb {1}_{\{s<0\}})\operatorname {Re}[\mathfrak G_{(k)}\overline{\uppsi }_{(k)}']\textrm{d}r^*\\&\quad \leqq \frac{B(\varepsilon _\textrm{width})}{\varepsilon _\textrm{width}\omega _\textrm{high}^{2}}\int _{-\infty }^\infty (y'\mathbb {1}_{\{s>0\}}+\hat{y}'\mathbb {1}_{\{s<0\}})(\omega ^2-\mathcal {V})|\Psi |^2 \textrm{d}r^*\\&\quad -\int _{-\infty }^\infty 2 (y\mathbb {1}_{\{s>0\}}+\hat{y}\mathbb {1}_{\{s<0\}})\operatorname {Re}[\mathfrak G_{(k)}\overline{\uppsi }_{(k)}']\textrm{d}r^*\,, \end{aligned}$$

where the first term on the right hand side can be absorbed by the left hand side of (6.39) if $\omega _\textrm{high}$ is sufficiently large depending on $\varepsilon _\textrm{width}$. It is not hard to see that in the case of , the above instances of $B(\varepsilon _\textrm{width})$ can be replaced by *B* and $\varepsilon _\textrm{width}\omega _\textrm{high}^{2}$ can be replaced by $\varepsilon _\textrm{width}^{-1}\omega _\textrm{high}^{2}$, and so $\omega _\textrm{high}$ can be chosen to be large independently of $\varepsilon _\textrm{width}$. □

### The bounded frequencies

In this section, we will establish Theorem 6.3 for frequency triples $(\omega ,m,\Lambda )$ which are bounded. We begin, in Section 6.4.1 by stating some important properties of the frequency triple and of the transformed system potentials in these regimes. Next, in Section 6.4.2, we establish two estimates which will be important to our analysis. We obtain our main results in Section 6.4.3, for frequencies where |ω| and $|\omega -m\upomega _+|$ are both small, Section 6.4.4, for the case where |ω| is small and $|\omega -m\upomega _+|$ is bounded away from zero, and Section 6.4.5, in the case where |ω| is bounded away from zero.

For convenience, we use $\omega _0$,  and $\hat{\mathcal {V}}_{(k)}$ to denote that

#### Properties of the frequency parameters and potential

We begin by introducing the key properties of the potentials $\mathcal {V}_{(k)}$, k=0,...,|s|, in (4.20) which are essential to the construction of suitable currents in the bounded frequency regime $\mathcal {F}_\textrm{bdd}(\omega _\textrm{high},\varepsilon _\textrm{width})$.

We start with some very general bounds.

##### Lemma 6.33

(Properties of the frequency parameters in $\mathcal {F}_\textrm{bdd}$) Let $(\omega ,m,\Lambda )\in \mathcal {F}_\textrm{bdd}(\varepsilon _\textrm{width},\omega _\textrm{high})$ be an admissible frequency triple. Then, we have $m^2, |\Lambda | \leqq B(\varepsilon _\textrm{width},\omega _\textrm{high})$. If, additionally, $|a|\leqq \tilde{a}_0$ with $\tilde{a}_0$ sufficiently small depending on $\varepsilon _\textrm{width}$ and $\omega _\textrm{high}$, then we have $\Lambda \geqq \min \{m^2,\frac{1}{2}|s|\}\geqq 0.$

##### Proof

Follows from the admissibility conditions in Definition 4.1. □

##### Lemma 6.34

(Properties of the potential for frequencies in $\mathcal {F}_\textrm{bdd}$) Let $(\omega ,m,\Lambda )\in \mathcal {F}_\textrm{bdd}(\varepsilon _\textrm{width},\omega _\textrm{high})$ be an admissible frequency triple. For each k=0,⋯,|s|, the potential $\mathcal {V}_{(k)}$ introduced in (4.20) has the following properties:

(i) For any $$\in (r_+,\infty )$$,
  $$\begin{aligned} |\mathcal {V}_{(k)}|+r|\mathcal {V}_{(k)}'|\leqq B(\omega _\textrm{high},\varepsilon _\textrm{width})\frac{1}{r^2}\,; \end{aligned}$$
(ii) For *r* sufficiently close to $r=r_+$, we have
  $$\begin{aligned} \hat{\mathcal {V}}_{(k)}' +\frac{2(3r_+^2-a^2)}{r_+}m(\omega -m\upomega _+)\Delta&\geqq -B(\omega _\textrm{high},\varepsilon _\textrm{width})(r-M)\Delta ; \end{aligned}$$
(iii) If $a\leqq \tilde{a}_0$ with $\tilde{a}_0$ sufficiently small, then for sufficiently large *r*, we have that, for s≠0
  $$\begin{aligned} \mathcal {V}_{(k)}\geqq \frac{2|s|}{r^2}-\frac{a_0 B(\omega _\textrm{high},\varepsilon _\textrm{width})}{r^3}\,,\quad - \mathcal {V}'_{(k)}\geqq \frac{4|s|}{r^3}-\frac{a_0 B(\omega _\textrm{high},\varepsilon _\textrm{width})}{r^4}\,, \end{aligned}$$
  and for |s|=0,
  $$\begin{aligned} \mathcal {V}\geqq \frac{\Lambda }{r^2}+\frac{2M}{r^3}-\frac{a_0 B(\omega _\textrm{high},\varepsilon _\textrm{width})}{r^3}\,,\quad -\mathcal {V}'\geqq \frac{2\Lambda }{r^3}+\frac{6M}{r^4}-\frac{a_0 B(\omega _\textrm{high},\varepsilon _\textrm{width})}{r^4}\,. \end{aligned}$$

##### Proof

The behavior at $r\rightarrow \infty$ follows easily by inspection of (4.20). For the $r\rightarrow r_+$ end, we can compute that

$$\begin{aligned} (r_+^2+a^2)^3\frac{\textrm{d}}{\textrm{d}r}\hat{\mathcal {V}}_{(k)}(r_+)&= 2(r_+-r_-)\left(Mr_+\left(\Lambda +|s|+k(2|s|-k-1)\right)-a^2m^2\right)\\&\qquad -4M am (\omega -m\upomega _+)(3r_+^2-a^2)\,. \end{aligned}$$

Using the bound on Λ from Lemma 6.33, we estimate the first term by $B(\omega _\textrm{high},\varepsilon _\textrm{width})(r_+-r_-)$; using the bound on |*m*|, we estimate the second term similarly. □

Next, we look at the case where $|\omega |\ll 1$.

##### Lemma 6.35

(Properties of the frequency parameters in $\mathcal {F}_\textrm{low}$) Let $(\omega ,m,\Lambda )\in \mathcal {F}_\textrm{low}(\varepsilon _\textrm{width},\omega _\textrm{high},\omega _\textrm{low})$. Then, for sufficiently small $\omega _\textrm{low}$ depending on *M*, $\varepsilon _\textrm{width}$ and $\omega _\textrm{high}$, we have

(i) $$\Lambda +s^2\geqq m^2$$;
(ii) $$\Lambda - 2am\omega \geqq \frac{7}{8}\max \{|m|,|s|\}\geqq 0$$ and $$\Lambda \geqq \frac{3}{4}\max \{|s|,|m|\}\geqq 0$$;
(iii) in $\mathcal {F}_\textrm{low,1a}$, $(\omega -m\upomega _+)^2\leqq 4\omega _\textrm{low}^2$; in $\mathcal {F}_\textrm{low,2}$, $(\omega -m\upomega _+)^2\geqq b(\tilde{a}_0)$;
(iv) for |s|=0,1,2, we have
  $$\begin{aligned} b\leqq \frac{\mathfrak C_s}{\mathfrak D_s^{\mathcal {H}}}\leqq B\,, \qquad b(\varepsilon _\textrm{width},\omega _\textrm{high})\leqq \frac{\mathfrak D_{s,k}^{\mathcal {H}}}{\mathfrak D_s^{\mathcal {H}}}\leqq B\,. \end{aligned}$$

##### Proof

From property IV(b) of the admissibility conditions in Definition 4.1, we have

$$\begin{aligned} \Lambda +s^2-m^2&\geqq \max \{|m|,|s|\}-2|s||a\omega |\geqq \max \{|m|,|s|\}(1-2M\omega _\textrm{low})\geqq 0\,,\\ \Lambda -2am\omega&\geqq \max \{m^2-s^2+|m|,|s|\}-2(|s|+|m|)|a\omega |\geqq \frac{7}{8}\max \{|m|,|s|\}\,,\\ \Lambda&\geqq \frac{7}{8}\max \{|m|,|s|\}-2|a||m|\omega |\geqq \frac{3}{4}\max \{|m|,|s|\} \,, \end{aligned}$$

as long as $\omega _\textrm{low}\leqq (16M)^{-1}$, which proves (i) and (ii). Property (iii) follows from

$$\begin{aligned} (\omega -m\upomega _+)^2\leqq (|\omega |+|m|\upomega _+)^2 \leqq \left(\omega _\textrm{low}+\frac{\tilde{a}_0}{4M^2}\sqrt{\varepsilon _\textrm{width}^{-1}\omega _\textrm{high}^2+s^2}\right)^2\leqq 4\omega _\textrm{low}^2\,, \end{aligned}$$

as long as $\tilde{a}_0\leqq 4\,M^2\omega _\textrm{low}(\varepsilon _\textrm{width}^{-1}\omega _\textrm{high}^2+s^2)^{-1/2}$. Finally, (iv) follows by inspection of the formulas in Proposition 4.6 and Lemma 4.10 for ω=0, and their analyticity in ω. □

##### Lemma 6.36

(Properties of the potential for frequencies in $\mathcal {F}_\textrm{low}$) Let $(\omega ,m,\Lambda )\in \mathcal {F}_\textrm{low}(\varepsilon _\textrm{width},\omega _\textrm{high},\omega _\textrm{low})$ be an admissible frequency triple. For each k=0,...,|s|, the potentials $\mathcal {V}_{(k)}$ introduced in (4.20), respectively, has the following properties if $\omega _\textrm{low}$ is sufficiently small depending on $\varepsilon _\textrm{width}$ and $\omega _\textrm{high}$.

(i) $\mathcal {V}_{(k)}$ is positive in a large compact $r^*$ range. Concretely, if s≠0 or if s=0 and $m,\Lambda \ne 0$, then there are $-\infty<R^*_{-}\leqq R_+^*<\infty$ so that
  $$\begin{aligned}\frac{1}{8} w&\leqq \mathcal {V}_{(k)}-\sigma (|s|-k)\left(\frac{w'}{w}\right)'-\omega ^2\\&\leqq (\Lambda +2+3\,s^2)w \quad \forall r^*\in [R_-^*, R_+^*],\,\, \forall \sigma \in \left[0,\frac{1}{2}\right],\end{aligned}$$
  as long as,
  - if $|\omega |\leqq \omega _\textrm{low}$ and $a\leqq \tilde{a}_0$ are small enough depending on $R_+^*-R_-^*$;
  - if m=0 and $|\omega |\leqq \omega _\textrm{low}$ is small enough depending on $R_+^*-R_-^*$;
  - if m≠0 and $a\geqq \tilde{a}_0>0$, $R_-^*>0$ is sufficiently large and $|\omega |\leqq \omega _\textrm{low}$ is small enough depending on $R_+^*$.
  If s=m=Λ=0, then
  $$\begin{aligned} M\Delta r^{-5} \leqq \mathcal {V}-\omega ^2 \leqq 2\,M\Delta r^{-5} \,. \end{aligned}$$
(ii) $\mathcal {V}_{(k)}'<0$ for sufficiently large positive $r^*$. Concretely, if s≠0 or if s=0 and $m,\Lambda \ne 0$, then there is a c>0 satisfying $7|s|/2 \leqq c \leqq 2\Lambda +s^2$ so that
  $$\begin{aligned} \mathcal {V}_{(k)}'=-cr^{-3} +O(r^{-4})\text { as }r\rightarrow \infty \,; \end{aligned}$$
  if s=m=Λ=0, then
  $$\begin{aligned} \mathcal {V}_{(k)}'=-6 Mr^{-4} +O(r^{-5})\text { as }r\rightarrow \infty \,. \end{aligned}$$
(iii) If $(\omega ,m,\Lambda )\in \mathcal {F}_\textrm{low,1}$ and $\tilde{a}_0$ is sufficiently small depending on $\varepsilon _\textrm{width}$ and $\omega _\textrm{high}$, we have $\mathcal {V}_{(k)}'>0$ for sufficiently negative $r^*$. Concretely, if m=0, there is a c>0 satisfying $3|s|/4\leqq c-1\leqq \Lambda +2|s|(|s|-1)$ such that
  $$\begin{aligned} \mathcal {V}_{(k)}' = \frac{c}{2M^2r_+^2}\Delta (r-M)+ O(\Delta ^2)\,; \end{aligned}$$
  if $|a|\leqq \tilde{a}_0$ and $\tilde{a}_0$ is sufficiently small, then there is a *c* satisfying $3|s|/4\leqq c\leqq \Lambda +2|s|(|s|-1)+2$ such that
  $$\begin{aligned} \mathcal {V}_{(k)}' = \frac{c}{2M^2r_+^2}\Delta + O(\Delta ^2)\,. \end{aligned}$$
(iv) If $(\omega ,m,\Lambda )\in \mathcal {F}_\textrm{low,2}$ then $\omega ^2-\mathcal {V}_{(k)}(r_+)=(\omega -m\upomega _+)^2\geqq b(\tilde{a}_0)>0$.

##### Proof

The lemma follows by direct inspection of (4.20). We remark that one can only have Λ=0 if m=0 and s=0 (by properties (i) and (ii) in Lemma 6.35).

For statement (i), let us first consider the case s=0. We have

$$\begin{aligned} \mathcal {V}_{(k)}&\geqq w\left\rbrace \Lambda +a^2w+\frac{2Mr(r^2-a^2)}{(r^2+a^2)^2}\right\lbrace + \frac{4Mram\omega -a^2m^2}{(r^2+a^2)^2}\\&\geqq w\Lambda +\frac{2Mr(r^2-a^2)w+4Mram\omega -a^2m^2}{(r^2+a^2)^2}\,. \end{aligned}$$

If s≠0, the proof follows by analyzing the frequency independent part of $\mathcal {V}_{(k)}-\sigma (|s|-k)(w'/w)'$ for $\sigma \in [0,5/4]$. By Lemma 6.35(ii),

$$\begin{aligned} U_{(k)}&:=\frac{1}{w}\left(\mathcal {V}_{(k)}-\frac{4Mram\omega -a^2m^2}{(r^2+a^2)^2}-\sigma (|s|-k)\left(\frac{w'}{w}\right)' \right)\\&\geqq 3\frac{|s|}{4}+|s| + k (2 |s|-k-1)-2\sigma (|s|-k)\\&\qquad +a^2w[1-2|s|-k (2 |s|-k-1)+4\sigma (|s|-k)]\\&\qquad +\frac{2Mr(r^2-a^2)}{(r^2+a^2)^2}[1-3|s| + 2 s^2 - 3 k (2 |s| - k - 1) + 6\sigma (|s| - k)]\,. \end{aligned}$$

In the above, we have

$$\begin{aligned}&\frac{2Mr(r^2-a^2)}{(r^2+a^2)^2}\leqq {\left\{ \begin{array}{ll} \sqrt{1-\frac{a^2}{M^2}}, &  \left|\frac{a}{M}\right|\in \left[0,\frac{1}{\sqrt{2}}\right]\\ \frac{1}{\sqrt{2}}, &  \left|\frac{a}{M}\right|\in \left[\frac{1}{\sqrt{2}},1\right] \end{array}\right. },\\&\qquad a^2 w\leqq \frac{3-2\sqrt{2}}{4} \frac{a^2}{M^2}\leqq {\left\{ \begin{array}{ll} \frac{a^2/M^2}{20}, &  \left|\frac{a}{M}\right|\in \left[0,\frac{1}{\sqrt{2}}\right]\\ \frac{1}{20}, &  \left|\frac{a}{M}\right|\in \left[\frac{1}{\sqrt{2}},1\right] \end{array}\right. }, \end{aligned}$$

and thus, by analyzing different regimes of (*k*, *s*), one can obtain the desired bounds; see for instance our preprint [151] or the thesis [156] for further details.

For statements (ii) and (iii), it is enough to consider the expansions

$$\begin{aligned} \mathcal {V}_{(k)}'&=-\frac{2[\Lambda +|s|-k+k(2|s|-k)]}{r^3}\\&\quad -\frac{6M\left[1-2s^2-4k(2|s|-k)-4(|s|-k)-(\Lambda -2am\omega )\right]}{r^4}\\&\quad +O\left(r^{-5}\right)\,,\quad \text { as }r\rightarrow \infty \,,\\ \frac{\textrm{d}}{\textrm{d}r}\mathcal {V}_{(k)}&=\frac{2m\upomega _+(m\upomega _+-\omega )}{M}+\frac{2m(r_+-M)(m-a\omega )}{2M^2r_+^2}\\&\quad +(r-M)\frac{\Lambda +|s|+k(2|s|-k-1)-2m^2}{2M^2r_+^2}\\&\quad +(r-M)(r_+-M)\frac{\left(1-3 |s| + 2 s^2- 3 k (2 |s|- k- 1)\right)}{2M^3r_+^2}\\&\quad +O(\Delta )\,,\quad \text { as }r\rightarrow r_+\,, \end{aligned}$$

where we note that, if |a|<M,

$$\begin{aligned}&\frac{2m(r_+-M)(m-a\omega )}{2M^2r_+^2}+(r-M)\frac{\Lambda +|s|+k(2|s|-k-1)-2m^2}{2M^2r_+^2} \\&\quad = (r_+-M)\frac{\Lambda +|s|+k(2|s|-k-1)-2am\omega }{2M^2r_+^2} +O(r-r_+)\,,\quad \text { as }r\rightarrow r_+\,. \end{aligned}$$

Coupled with Lemma 6.35(ii), these identities give the results. □

#### Estimates for the k<|s| transformed variables

In this section, we derive estimates that we will use throughout the section to prove estimates for $(\omega ,m,\Lambda )\in \mathcal {F}_\textrm{bdd}$ or subsets thereof.

We begin with a very general lemma concerning localization errors for the Teukolsky–Starobinsky energy currents introduced in Lemma 5.4.

##### Lemma 6.37

Fix $s\in \mathbb {Z}$. Let $(\omega ,m,\Lambda )$ be an admissible frequency triple. For a smooth $\chi (r^*)$ of compact support for $L\leqq r^*\leqq 2\,L$ with L≫1 such that $\left(\frac{\textrm{d}}{\textrm{d}r^*}\right)^N\chi =O(L^{-N})$, one has that

6.42 $$\begin{aligned}&\int _{-\infty }^\infty \chi ' Q^{W,\, \{T,K\}}_{\pm }-\int _{-\infty }^\infty \chi ' \{\omega ,\omega -m\upomega _+\} \operatorname {Im}\left[\Psi '\overline{\Psi }\right] \\&\quad \leqq \frac{B\{|\omega |,|\omega -m\upomega _{+}|\}}{L}\left(1{+}\frac{|am|}{L}\right)\sum _{k=0}^{|s|}\sum _{j=0}^{|s|-1}L^{(|s|-k){+}(|s|-j)}\nonumber \\&\quad \times \int _{\textrm{supp}(\chi ')}\left[\left|\uppsi _{(k)}'\right|\left|\uppsi _{(j)}\right|{+}L^{-1}\left|\uppsi _{(k)}\right|\left|\uppsi _{(j)}\right|\right], \nonumber \end{aligned}$$

where the alternatives in braces correspond to the *T*-type and *K*-type Teukolsky–Starobinsky currents as defined in Section 5.1.

##### Proof

Recall (5.10). The goal is to integrate by parts the left hand side of (6.42), allowing the $\hat{\mathcal {D}}^\pm$ operators in $q_W$ to be equally distributed between $\uppsi _{(0)}$ and its image under the Teukolsky–Starobinsky maps, and using the relation (4.19) to make $\uppsi _{(k)}$ appear. Indeed, taking s>0 without loss of generality, we have

6.43 $$\begin{aligned} \chi ' q_W&= \left[2w^{s/2}\left(w^{-s/2}\uppsi ^{[{\pm s}]}_{(0)}\right)'\chi ' +\chi ''\uppsi ^{[{\pm s}]}_{(0)}\right](r^2+a^2)^{s+1/2}\nonumber \\&\quad \overline{\left((r^2+a^2)^{-1}\frac{{\mathcal {L}}}{w}\right)^{2s}\left[(r^2+a^2)^{s-1/2}\uppsi ^{[{\pm s}]}_{(0)}\right]}\nonumber \\&= (-1)^s w(r^2+a^2)\left[(r^2+a^2)^{-1}\frac{\mathcal {L}}{w}\right]^s\nonumber \\&\quad \left\rbrace \left[2w^{s/2}\left(w^{-s/2}\uppsi ^{[{\pm s}]}_{(0)}\right)'\chi ' +\chi ''\uppsi ^{[{\pm s}]}_{(0)}\right](r^2+a^2)^{s-1/2}w^{-1}\right\lbrace \nonumber \\&\qquad \times \overline{\left((r^2+a^2)^{-1}\frac{{\mathcal {L}}}{w}\right)^{s}\left[(r^2+a^2)^{s-1/2}\uppsi ^{[{\pm s}]}_{(0)}\right]}\,, \end{aligned}$$

where we have ignored full derivatives due to the compact support of $\chi^{\prime }$ (upon integration, they generate boundary terms that vanish). By this procedure, one obtains, for s=1,

$$\begin{aligned}&\operatorname {Im}\left(\chi 'q_W\right) \\&\quad = -2\chi '\operatorname {Im}\left[\Psi '\overline{\Psi }\right] +2r\chi '\operatorname {Im}\left[\Psi ' \overline{\uppsi _{(0)}}\right]-2r\left(\frac{\chi ''}{rw}+\chi '\right)\operatorname {Im}\left[\Psi \overline{\uppsi _{(0)}}'\right]\\&\qquad -\operatorname {Im}\left\rbrace \left[\frac{\chi '''}{w}-\frac{2\chi '' w'}{w^2}-\left(\frac{w'}{w^2}\right)'\chi '-4iam\frac{r}{r^2+a^2}\chi '\right]\left[\Psi \overline{\uppsi _{(0)}}\right]\right\lbrace \\&\qquad -2r^2\left(\frac{\chi ''}{rw}+\chi '\right)\operatorname {Im}\left[\uppsi _{(0)}'\overline{\uppsi _{(0)}}\right]\,, \end{aligned}$$

again ignoring full derivatives. More generally, if $\chi ^{(N)}\sim r^{-N}\sim L^{-N}$, we obtain the final result. □

Next, we derive another identity which will be useful to control boundary terms. The next result states that, while boundary terms at $r^*=(-{{\,\textrm{sign}\,}}s)\infty$ throughout the hierarchy of transformed variables may not be related by the second part of Lemma 4.10 unless $\mathfrak {G}_{(k)}$ have strong decay, we can still obtain general estimates relating *k* boundary terms with j<k boundary terms.

##### Lemma 6.38

(Relating boundary terms) Let $(\omega ,m,\Lambda )\in \mathcal {F}_\textrm{bdd}(\varepsilon _\textrm{width},\omega _\textrm{high})$ be an admissible frequency triple. For k=0,⋯,|s|, assume $\uppsi _{k}$ are solutions to the radial ODEs (4.19) with outgoing boundary conditions. Then, we have the following boundary term estimates for k<|s|. For s>0,

$$\begin{aligned}&\left[(|s|-k-1)^2\left(\frac{r_+-M}{2Mr_+}\right)^2 +(\omega -m\upomega _+)^2\right]|\uppsi _{(k+1)}(-\infty )|^2\\&\quad \leqq B\left|\frac{\mathfrak {G}_{(k)}}{w}\right|^2(-\infty )+B(\omega _\textrm{width},\varepsilon _\textrm{width})|\uppsi _{(k)}(-\infty )|^2\\&\qquad +B(\omega _\textrm{width},\varepsilon _\textrm{width})\sum _{j=0}^{k-1}|\uppsi _{(j)}(-\infty )|^2\,. \end{aligned}$$

For s<0, we have instead that

$$\begin{aligned} \omega ^2|\uppsi _{(k+1)}(+\infty )|^2&\leqq B\left|\frac{\mathfrak {G}_{(k)}}{w}\right|^2(+\infty )\\&\quad +B(\omega _\textrm{width},\varepsilon _\textrm{width})|\uppsi _{(k+1)}(+\infty )|^2\\&\quad +B(\omega _\textrm{high},\varepsilon _\textrm{width})\sum _{j=0}^{k-1}|\uppsi _{(j)}(+\infty )|^2\,. \end{aligned}$$

##### Proof

Recall the radial ODE for s>0 in the constraint form of (4.23). Dropping subscripts, using the definition of $\uppsi _{(k+1)}$ (4.17), and dividing through by *w*, we find that (4.19) at $r^*=-\infty$ can be recast as

$$\begin{aligned}&\left(\frac{\textrm{d}}{\textrm{d}r^*}-i(\omega -m\upomega _+)\right)\uppsi _{(k+1)}\Big |_{r=r_+}- 2(|s|-k-1)\kappa \uppsi _{(k+1)}(-\infty )\\&\quad \qquad +\left(|s|+k(2|s|-k-1)+\frac{r_+-M}{M}(1-3|s|+2s^2-3k(2|s|-k-1))\right)\uppsi _{(k)}(-\infty )\\&\quad \qquad +\left(\Lambda -2am\omega -\frac{a}{M}{{\,\textrm{sign}\,}}s(2|s|-2k-1) im\right)\uppsi _{(k)}(-\infty )\\&\quad = \frac{\mathfrak {G}_{(k)}}{w}\Big |_{r=r_+}+\sum _{i=0}^{k-1}\left(a c_{s,\,k,\,i}^\Phi im+ac^\textrm{id}_{s,\,k,\,i}\right)\Big |_{r=r_+}\uppsi _{(i)}(-\infty )\,, \end{aligned}$$

where we have used the notation $\kappa =\frac{r_+-M}{2Mr_+}$. Assume outgoing boundary conditions, see Definition 4.9, and multiply by $\left[ (|s|-k-1)\kappa +i(\omega -m\upomega _+) \right]\overline{\uppsi _{(k+1)}(-\infty )}$ to obtain the desired estimate. Similarly, if s<0, we can rewrite (4.19) as

$$\begin{aligned}&\left(-\frac{\textrm{d}}{\textrm{d}r^*}-i\omega \right)\uppsi _{(k+1)}\Big |_{r=\infty }+\left(\Lambda +|s|+k(2|s|-k-1)\right)\uppsi _{(k)}\Big |_{r=\infty }\\&\quad = \frac{\mathfrak {G}_{(k)}}{w}\Big |_{r=\infty }+\sum _{i=0}^{k-1}\left(a c_{s,\,k,\,i}^\Phi im+ac^\textrm{id}_{s,\,k,\,i}\right)\Big |_{r=\infty }\uppsi _{(i)}\Big |_{r=\infty }\,, \end{aligned}$$

thus arguing as before concludes the proof. □

The final estimates concern only the regime $\mathcal {F}_\textrm{low}$, where ω is small. A first result encodes the observation that, if $|\omega |\ll 1$ and all frequency parameters are bounded, the large *r* behavior of $\mathcal {V}_{(k)}$ is independent of *am*.

##### Lemma 6.39

(An estimate for large $r^*$) Fix M>0 and $s\in \mathbb {Z}$. For each $R_3^*>0$, choose parameters $C_{i,(k)}^+=C_{i,(k)}^+(R_3^*)>0$, i=0,1,2 and k=0,⋯,|s|, satisfying $C_{i,(k)}\leqq BC_{i,(j)}$ for j<k. Let $\chi _2$ be a smooth function which is identically 1 for $r\geqq R_3^*$. Let $h_{(k)}$ be a $C^1$ function and $y_{(k)}$ be a $C^0$ function with

$$\begin{aligned} h_{(k)}(R_3^*)= C_{0,(k)}^+\,, \quad h_{(k)}'(R_3^*)=C_{1,(k)}^+\,, \qquad y_{(k)}=C_{2,(k)}^+, \end{aligned}$$

and with $h_{(k)}=h_{(k)}^\textrm{right}$ and $y_{(k)}=y_{(k)}^\textrm{right}$ for $r^*\geqq R_3^*$, where

6.44 $$\begin{aligned} h_{(k)}^\textrm{right}\quad&\!\!=[C_{0,(k)}^+(R_3^*)+R_3^*C_{1,(k)}^+(R_3^*)]\left[1+\frac{p\left(1-\frac{|R_3^*|}{|r^*|}+\log \left(\frac{|R_3^*|}{|r^*|}\right)\right)}{(1-2p(1-\log 2))}\right]\mathbb {1}_{[R_3^*,e^{p^{-1}}R_3^*]}\nonumber \\&\qquad -C_{1,(k)}^+(R_3^*)R_3^*\frac{|R_3^*|}{|r^*|}\mathbb {1}_{[R_3^*,e^{p^{-1}}R_3^*]}\nonumber \\&\qquad + \frac{p[C_{0,(k)}^+(R_3^*)+R_3^*C_{1,(k)}^+(R_3^*)]}{(1-2p(1-\log 2))}\times \nonumber \\&\qquad \left[2\left(\frac{|r^*|}{2e^{p^{-1}}|R_3^*|}-1\right)-2\log \left(\frac{|r^*|}{2e^{p^{-1}}|R_3^*|}\right)\right]\mathbb {1}_{[e^{p^{-1}}R_3^*,2e^{p^{-1}}R_3^*]}\nonumber \\&\qquad + \frac{2e^{-1/p}}{(\log 8-2)}\left(\frac{p[C_{0,(k)}^+(R_3^*)+R_3^*C_{1,(k)}^+(R_3^*)]}{(1-2p(1-\log 2))}+R_3^*C_{1,(k)}^+(R_3^*)\right)\mathbb {1}_{[e^{p^{-1}}R_3^*,2e^{p^{-1}}R_3^*]}\nonumber \\&\qquad \times \left[(1-\log 2)\left(\frac{2e^{p^{-1}}R_3^*}{|r^*|}-1\right)-\log 2\left(\frac{|r^*|}{2e^{p^{-1}}|R_3^*|}-1\right)\right.\left.+\log \left(\frac{|r^*|}{2e^{p^{-1}}|R_3^*|}\right)\right]\,, \end{aligned}$$

6.45 $$\begin{aligned} \qquad \qquad \qquad y_{(k)}^\textrm{right}&=C_{2,(k)}^+(R_3^*)\left[1+\epsilon \left(\frac{1}{|r^*|\mathcal {V}}-\frac{1}{R_3^*\mathcal {V}(R_3^*)}\right)\right]\mathbb {1}_{[R_3^*,2e^{p^{-1}}R_3^*]} \nonumber \\&\qquad +y_{(k)}^\textrm{right}(2e^{p^{-1}}R_3^*)\left[1+\frac{1}{|2e^{p^{-1}}R_3^*|^{\delta _I}}-\frac{1}{|r^*|^{\delta _I}}\right]\mathbb {1}_{[2e^{p^{-1}}R_3^*,\infty )}\,, \end{aligned}$$

for some positive parameters *p*, ϵ, $\delta _H$ and $\delta _I$. Then, for all $(\omega ,m,\Lambda )\in \mathcal {F}_\textrm{low,1}(\varepsilon _\textrm{width},\omega _\textrm{high},\omega _\textrm{low})\cup \mathcal {F}_\textrm{low,2}(\varepsilon _\textrm{width},\omega _\textrm{high},\omega _\textrm{low},\tilde{a}_0)$, for all $\delta _I\in (0,1]$, $\varepsilon _\textrm{width}>0$, $\omega _\textrm{high}>0$ and $\tilde{a}_0$, for all $E\geqq 2\max _k y_{(k)}(\infty )$, for all $R_3^*$ sufficiently large depending on $\varepsilon _\textrm{width}$ and $\omega _\textrm{high}$, for all ϵ sufficiently small depending on $R_3^*$, for all p∈(0,1) sufficiently small depending on $\delta _I$, $C_{i,(k)}^+(R_3^*)$ for i=0,1,2 and k=0,⋯|s|, and ϵ, for $\omega _\textrm{low}$ sufficiently small depending on *E*, $R_3^*$, $C_{i,(k)}^+(R_3^*)$ for i=0,1,2 and k=0,⋯|s|, $\varepsilon _\textrm{width}$ and $\omega _\textrm{high}$, the following estimate holds:6.46

##### Proof

The lemma follows from applying $Q^{y_{(k)}}$, $Q^{h_{(k)}}$ and $E\chi _2 Q^T$ to (4.19). Our construction of these currents is very similar to those of the s=0 case in [57, Section 8.7]. Indeed, in view of Lemmas 5.1 and 5.3 and Lemma 6.36, among the terms generated by these currents, we can distinguish between “main terms” which behave like those one obtains for s=0, and “additional terms” which we treat as errors.

*Main terms.* We take $R_3^*$ to be large enough, depending on $\varepsilon _\textrm{width}$ and $\omega _\textrm{high}$, that $\mathcal {V}_{(k)}'<0$ for $r^*\geqq R_3^*$ for all k=0,⋯|s|, as per Lemma 6.36(ii). We take $\epsilon =\epsilon (R_3^*)$ to be sufficiently small to ensure that, in the formula

$$\begin{aligned}&-(y_{(k)}\mathcal {V}_{(k)})' \mathbb {1}_{[R_3^*, 2e^{p^{-1}}R_3^*]} = C_{2,(k)}^+\left(1-\frac{\epsilon }{(R_3^*)\mathcal {V}_{(k)}(R_3^*)}\right)\\&\quad \left(-\mathcal {V}_{(k)}\right)' \mathbb {1}_{[R_3^*, 2e^{p^{-1}}R_3^*]} +\frac{C_{2,(k)}^+\epsilon }{(r^*)^2} \mathbb {1}_{[R_3^*, 2e^{p^{-1}}R_3^*]}\,, \end{aligned}$$

the first term is positive. Then, noting that

$$\begin{aligned} h_{(k)}''+(|s|-k)\frac{w'}{w}h_{(k)}'\leqq \frac{Bp [C_{0,(k)}^+(R_3^*)+R_3^*C_{1,(k)}^+(R_3^*)]}{(r^*)^2}\mathbb {1}_{[R_3^*, 2e^{p^{-1}}R_3^*]}\,, \end{aligned}$$

we see that if *p* is made to be sufficiently small depending on $C_{i,(k)}^+$, with $i\in \{1,2,3\}$, and $\epsilon =\epsilon (R_3^*)$, the error introduced by derivatives of $h_{(k)}$ in $[R_3^*,2e^{p^{-1}}R_3^*]$ can controlled by the $y_{(k)}$ current. Moreover, for $r^*\geqq 2e^{p^{-1}}R_3^*$,

$$\begin{aligned} -(y_{(k)}\mathcal {V}_{(k)})'&= -y_{(k)}\mathcal {V}_{(k)}'\left(1+\frac{y_{(k)}'}{y_{(k)}}\frac{\mathcal {V}_{(k)}}{\mathcal {V}_{(k)}'}\right)\\&\geqq -y_{(k)}\mathcal {V}_{(k)}'\left(1-\frac{B\delta _I}{(2e^{p^{-1}}R_3^*)^{\delta _I}}\right)\geqq -\frac{1}{2} y_{(k)}\mathcal {V}_{(k)}'\,, \end{aligned}$$

making *p* sufficiently small depending on $\delta _I$.

Finally, it is easy to check that $h_{(k)}\geqq 0$ and $y'_{(k)}> 0$ for $r^*\geqq R_3^*$. Thus, the choices of $y_{(k)}$ and $h_{(k)}$ here ensure that a good bulk term in $\uppsi _{(k)}$ is produced for $r^*\geqq R_3^*$, at the cost of boundary terms, which are controlled by $E\chi _2Q^T$ if $E\geqq 2y_{(k)}(\infty )$, and additional terms that we will examine next.

*Additional terms.* We turn to the formulas in Lemmas 5.1 and 5.3 to control the additional terms generated by our multiplier currents for $r^*\geqq R_3^*$.

From $E\chi _2Q^T$ we have

$$\begin{aligned}&E(s-k)\omega \left[w'\operatorname {Im}[\uppsi _{(k+1)}\overline{\uppsi }_{(k)}]+\frac{4amrw}{r^2+a^2}|\uppsi _{(k)}|^2\right]\\&\quad \leqq - \left[\frac{1}{4} (y_{(k)}\mathcal {V}_{(k)})'|\uppsi _{(k)}|^2+B(m)\frac{E^2(|s|-k)^2\omega _\textrm{low}^2}{y_{(k)}(\infty )y_{(k+1)}(R_3^*)}\left(y_{(k+1)}\mathcal {V}_{(k+1)}\right)'|\uppsi _{(k+1)}|^2\right] \,, \end{aligned}$$

if $r^*\geqq R_3^*$; and from $Q^{y_{(k)}}$ we have, for the same $r^*$ range,

$$\begin{aligned}&2y_{(k)}(|s|-k)\left[w'w|\uppsi _{(k+1)}|^2-w'\omega \operatorname {Re}[\uppsi _{(k+1)}\overline{\uppsi _{(k)}}]\right]\\&\qquad +2y_{(k)}(|s|-k)\frac{4amrw}{r^2+a^2}\left[w\operatorname {Im}[\uppsi _{(k+1)}\overline{\uppsi _{(k)}}]-\left(\omega -\frac{am}{r^2+a^2}\right)|\uppsi _{(k)}|^2\right]\\&\quad \leqq -B(m)(|s|-k)^2\left(\omega _\textrm{low}^2+(R_3^*)^{-2}\right)\frac{y_{(k)}}{y_{(k+1)}}(y_{(k+1)}\mathcal {V}_{(k+1)})'|\uppsi _{(k+1)}|^2\\&\qquad -\frac{1}{4}(y_{(k)}\mathcal {V}_{(k)})'|\uppsi _{(k)}|^2\,. \end{aligned}$$

Furthermore, we have terms arising from the coupling to j<k: if $r^*\geqq R_3^*$, these are6.47where, to estimate terms of the form $(E+y_{\mathcal {(}k)})|w\uppsi _{(k)}\uppsi _{(j)}|$ by the last term, we have use the fact that, if s≠0,

$$\begin{aligned} y_{(k)}'\omega ^2- (y_{(k)}\mathcal {V}_{(k)})'|\geqq b\frac{y_{(k)}|\omega |}{r^{5/2}}. \end{aligned}$$

For the last term of (6.47), we invoke a basic estimate from Lemma 5.5: we set

$$\begin{aligned} c=-\frac{1}{r^{1/2}}\mathbb {1}_{[R_3,\infty )}-\frac{2r-R_3}{R_3^{3/2}}\mathbb {1}_{[R_3/2,R_3]}\,, \end{aligned}$$

and obtain

6.48 $$\begin{aligned}&\int _{-\infty }^\infty \frac{\Delta }{r^{2+3/2}}|\uppsi _{(j)}|^2 \mathbb {1}_{[R_3,\infty )} \textrm{d}r^*\leqq B\int \left(c'|\uppsi _{(j)}|^2 +\frac{1}{R_3^{3/2}}\mathbb {1}_{[R_3/2,R_3]}\right) \textrm{d}r^*\nonumber \\&\quad \leqq B\int _{-\infty }^\infty \left[\frac{w}{r^{3/2}}|\uppsi _{(j+1)}|^2 \mathbb {1}_{[R_3,\infty )}\right.\nonumber \\&\qquad \left. + w\left(\frac{1}{R_3^{3/2}}|\uppsi _{(j+1)}|^2+\left(1+\frac{r^2}{R_3^{3/2}}\right)|\uppsi _{(j)}|^2\right)\mathbb {1}_{[R_3/2,R_3]}\right] \textrm{d}r^*\nonumber \\&\quad \leqq B\int _{-\infty }^\infty \left(\frac{w}{r}|\uppsi _{(j+1)}|^2 \mathbb {1}_{[R_3/2,\infty )} +w R_3^{1/2}|\uppsi _{(j)}|^2\mathbb {1}_{[R_3/2,R_3]}\right) \textrm{d}r^*\,, \end{aligned}$$

where the last line follows from the fact that $r^2\sim R_3^2$ in $[R_3/2,R_3]$. The upshot is that we have pushed the errors to a compact *r* range where the $h_{(j)}$ current produces a good bulk term.

*Conclusion.* Assuming $E\geqq 2y_{(k)}(\infty )$, we obtain6.49Under the assumption that

$$\begin{aligned} \frac{C_{0,(k)}^+(R_3^*)}{C_{0,(j)}^+(R_3^*)} \leqq B\,, \quad \frac{C_{2,(k)}^+(R_3^*)}{C_{2,(j)}^+(R_3^*)} \leqq B \,,\qquad j<k \end{aligned}$$

with a constant which is independent of $R_3^*$, we see that if $R_3^*$ is sufficiently large, and if $\omega _\textrm{low}$ is chosen to be sufficiently small, in particular depending on *E* and $R_3^*$ in such a way that

$$\begin{aligned} \frac{E^2}{C_{2,(k)}^+(R_3^*)C_{0,(k)}^+(R_3^*)}\omega _\textrm{low}(R_3^*)^{1/2} \leqq B\,, \qquad k=0,\dots ,|s|, \end{aligned}$$

then the estimate follows by using Lemmas 5.1 and 5.3 to write out the additional terms in the last line of (6.49). □

#### Low time frequency and axisymmetry or small black hole angular momentum

This section concerns the frequency range $\mathcal {F}_\textrm{low,1}$. We will prove the following three propositions:

##### Proposition 6.40

(Estimates in $\mathcal {F}_\textrm{low,1a}$) Fix $s\in \mathbb {Z}$ and M>0. Then, for all $\delta _I\in (0,1]$, for all $\omega _\textrm{high}>0$, for all $\varepsilon _\textrm{width}>0$, for all E>0 sufficiently large, for all $\tilde{a}_0$ sufficiently small depending on $\delta _I$, $\varepsilon _\textrm{width}$, $\omega _\textrm{high}$ and *E*, for all $\omega _\textrm{low}$ sufficiently small depending on $\delta _I$, $\varepsilon _\textrm{width}$ and $\omega _\textrm{high}$, for all $(a,\omega ,m,\Lambda ) \in \mathcal {F}_\textrm{low,1a}(\omega _\textrm{high},\varepsilon _\textrm{width},\omega _\textrm{low},\tilde{a}_0)$, there exist functions y≧0, $\hat{y}\geqq 0$, h≧0, $0\leqq \chi _1\leqq 1$ and $0\leqq \chi _2\leqq 1$ satisfying the bounds (6.3) and a smooth function $\gamma _{1a}$ with the property that $\gamma _{1a}(x)\rightarrow 0$ as x→0 such that

$$\begin{aligned}&\omega ^2\left|\Psi (\infty )\right|^2+(\omega -m\upomega _+)^2\left|\Psi (-\infty )\right|^2\\&\qquad + b(\delta _I)\int _{-\infty }^\infty \frac{\Delta }{r^2}\Big [r^{-1-\delta _I}|\Psi '|^2+\frac{1}{r^3}\left(\Lambda +r^{-1}\right)|\Psi |^2\Big ]\textrm{d}r^*\\&\quad \leqq -\int _{-\infty }^\infty \left\rbrace 2(y+\hat{y})\operatorname {Re}\left[\mathfrak {G}\overline{\Psi }'\right]+h\operatorname {Re}\left[\mathfrak {G}\overline{\Psi }\right]\right.\\&\qquad \left.-E\left(\chi _2\omega +\chi _1(\omega -m\upomega _+)\right)\operatorname {Im}\left[\mathfrak {G}\overline{\Psi }\right]\right\lbrace \textrm{d}r^*\\&\quad \qquad +\gamma _{1a}(\tilde{a}_0)\Big (|\uppsi _{(0)}(-\infty )|^2+\sum _{k=0}^{|s|-1}\left|\frac{\mathfrak {G}_{(k)}}{w}\right|^2(-\infty )\Big )\mathbb {1}_{\{s> 0\}}\,. \end{aligned}$$

If $\mathfrak {G}_{(k)}=O(\Delta ^{|s|-k+1})$ as $r\rightarrow r_+$ for all k=0,⋯,|s|, we can drop the last term from the right hand side.

##### Proposition 6.41

(Estimates in $\mathcal {F}_\textrm{low,1b}$) Fix $s\in \mathbb {Z}$ and M>0. Then, for all $\delta _I\in (0,1]$, for all $\omega _\textrm{high}>0$, for all $\varepsilon _\textrm{width}>0$, for all E>0 sufficiently large, for all $\tilde{a}_1>0$, for all $\omega _\textrm{low}$ sufficiently small depending on $\delta _I$, $\omega _\textrm{high}$, $\varepsilon _\textrm{width}$, *E*, $\tilde{a}_1$, and for all $(a,\omega ,m,\Lambda ) \in \mathcal {F}_\textrm{low,1b}(\varepsilon _\textrm{width},\omega _\textrm{high},\tilde{a}_1)$, functions $y_{(k)}\geqq 0$, $\hat{y}_{(k)}\geqq 0$ and $h_{(k)}\geqq 0$ satisfying the bounds (6.3) and a smooth function $\gamma _{1b}$ with the property that $\gamma _{1b}(x)\rightarrow 0$ as x→0 such that

$$\begin{aligned}&\omega ^2\left|\Psi (\infty )\right|^2+(\omega -m\upomega _+)^2\left|\Psi (-\infty )\right|^2\\&\qquad + b(\delta _I, \tilde{a}_1)\int _{-\infty }^\infty \frac{\Delta }{r^2}\Big [r^{-1-\delta _I}|\Psi '|^2+\frac{1}{r^3}(\Lambda +r^{-\delta _I})|\Psi |^2\Big ]\textrm{d}r^*\\&\quad \leqq -\int _{-\infty }^\infty \left\rbrace 2(y+\hat{y})\operatorname {Re}\left[\mathfrak {G}\overline{\Psi }'\right]+h\operatorname {Re}\left[\mathfrak {G}\overline{\Psi }\right]-E\omega \operatorname {Im}\left[\mathfrak {G}\overline{\Psi }\right]\right\lbrace \textrm{d}r^*\\&\qquad +B\sum _{k=0}^{|s|-1}\left|\int _{-\infty }^\infty \left\rbrace 2(y_{(k)}+\hat{y}_{(k)})\operatorname {Re}\left[\mathfrak {G}_{(k)}\overline{\uppsi _{(k)}}'\right]\right.\right.\\&\qquad \qquad \quad \qquad \quad \qquad \left.\left.+h_{(k)}\operatorname {Re}\left[\mathfrak {G}_{(k)}\overline{\uppsi _{(k)}}\right]-E\omega \operatorname {Im}\left[\mathfrak {G}_{(k)}\overline{\uppsi _{(k)}}\right]\right\lbrace \textrm{d}r^*\right|\\&\quad \qquad \qquad \quad \quad \qquad \qquad +\gamma _{1b}(\omega _\textrm{low})\Big (|\uppsi _{(0)}(-\infty )|^2+\sum _{k=0}^{|s|-1}\left|\frac{\mathfrak {G}_{(k)}}{w}\right|^2(-\infty )\Big )\mathbb {1}_{\{s> 0\}}\,. \end{aligned}$$

If $\mathfrak {G}_{(k)}=O(\Delta ^{|s|-k+1})$ as $r\rightarrow r_+$ for all k=0,⋯,|s|, we can drop the last term from the right hand side.

##### Proposition 6.42

(Estimates in $\mathcal {F}_\textrm{low,1c}$) Fix $s\in \mathbb {Z}$ and M>0. Then, for all $\delta _H,\delta _I\in (0,1]$, for all $\omega _\textrm{high}>0$, for all $\varepsilon _\textrm{width}>0$, for all E>0 sufficiently large, for all $\tilde{a}_1\in (0,M)$ sufficiently small, for all $\omega _\textrm{low}$ sufficiently small depending on $\delta _I$, $\delta _H$, $\omega _\textrm{high}$, $\varepsilon _\textrm{width}$ and *E*, and for all $(a,\omega ,m,\Lambda ) \in \mathcal {F}_\textrm{low,1c}(\varepsilon _\textrm{width},\omega _\textrm{high},\tilde{a}_1)$, there exist and functions $y_{(k)}\geqq 0$, $\hat{y}_{(k)}\geqq 0$ and $h_{(k)}\geqq 0$ satisfying the bounds (6.3) such that

$$\begin{aligned}&\omega ^2\left|\Psi (\infty )\right|^2+(\omega -m\upomega _+)^2\left|\Psi (-\infty )\right|^2 \\&\qquad + b(\delta _H,\delta _I)\int _{-\infty }^\infty \frac{\Delta }{r^2}\Big [\frac{(1-Mr^{-1})^{\delta _H-1}}{r^{1+\delta _I}}|\Psi '|^2+\frac{(r-M)}{r^4}(\Lambda +r^{-\delta _I})|\Psi |^2\Big ]\textrm{d}r^*\\&\quad \leqq -\int _{-\infty }^\infty \left\rbrace 2(y+\hat{y})\operatorname {Re}\left[\mathfrak {G}\overline{\Psi }'\right]+h\operatorname {Re}\left[\mathfrak {G}\overline{\Psi }\right]-E\omega \operatorname {Im}\left[\mathfrak {G}\overline{\Psi }\right]\right\lbrace \textrm{d}r^*\\&\qquad +B\sum _{k=0}^{|s|-1}\left|\int _{-\infty }^\infty \left\rbrace 2(y_{(k)}+\hat{y}_{(k)})\operatorname {Re}\left[\mathfrak {G}_{(k)}\overline{\uppsi _{(k)}}'\right]\right.\right.\\&\qquad \qquad \qquad \qquad \quad \quad \left.\left.+h_{(k)}\operatorname {Re}\left[\mathfrak {G}_{(k)}\overline{\uppsi _{(k)}}\right]-E\omega \operatorname {Im}\left[\mathfrak {G}_{(k)}\overline{\uppsi _{(k)}}\right]\right\lbrace \textrm{d}r^*\right|\,. \end{aligned}$$

**Fig. 7:**
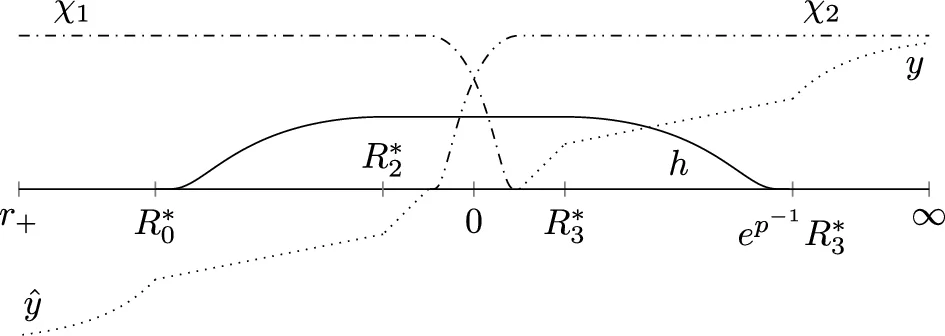
Sketch of currents in the proofs of Propositions 6.40, 6.41 and 6.42

We briefly review the strategy to prove Propositions 6.40, 6.41 and 6.42, which is best illustrated by Fig. 7. In $\mathcal {F}_\textrm{low,1}$, Lemma 6.36 has shown that $\mathcal {V}_{(k)}'$ have a definite sign as $r^*\rightarrow \pm \infty$, which can be exploited with *y* currents. To control the boundary terms they introduce, we rely on energy currents which, due to fact that both |ω| and $|\omega -m\upomega _+|$ are small, produce small (if any) localization errors. Though small, such localization errors must be controlled; for this, we use an *h* current which captures the fact that, by Lemma 6.36, $\mathcal {V}_{(k)}-\omega ^2>0$ in a large compact range of $r^*$. The *h* currents, which are themselves compactly supported, produce errors due to their derivatives. We have already shown in Lemma 6.39 how *h* and *y* currents for large *r* can be nontrivially connected so as to produce good bulk terms; repeating this scheme for $r\approx r_+$ will be our goal. Let us now distinguish between the three propositions:

- In $\mathcal {F}_\textrm{low,1a}$, we apply currents only to (4.21). This proof is entirely self-contained, but the logic is very similar to that of Lemma 6.39: we connect an *h* at bounded $|r^*|$ with *y* currents at large $|r^*|$ by the same procedure as in Lemma 6.39, that is, the currents for $r^*\leqq 0$ and $r^*\geqq 0$ are completely analogous. Up to this point, the proof is very similar to that of the case s=0 in [57, Proposition 8.7.1]. To conclude, we must show that the errors induced by the coupling terms on the right hand side of (4.21) are lower order. The smallness of *a* is essential to this goal, however it is not enough; since $\mathcal {V}'$ has stronger *r*-decay as $r\rightarrow \infty$ than the coupling terms, applying a suitable transport estimate (see Lemma 5.5) leads to boundary term errors at r=-∞ associated with k<|s| transformed variables. The boundary terms are weighted by $\tilde{a}_0$ but not by $(\omega -m\upomega _+)^2$.
- In $\mathcal {F}_\textrm{low,1b}$, we apply suitable currents to all of the transformed ODEs (4.19) with k=0,⋯,|s| to address errors arising from the interaction of the currents with the coupling terms on the right hand side of (4.19), instead of applying transport estimates. We use the current identities derived in Lemmas 5.1 and 5.3. In this case we find that our template *h* current in Lemma 6.39, when turned to the region of very negative $r^*$, does not produce an error
  $$\begin{aligned} h''+(|s|-k)\frac{w'}{w}h' \end{aligned}$$
  which is integrable, since $w^{\prime }=O\left(w\right)$ for subextremal *a*. The upshot is that we cannot connect the *h* and *y* current near $$r_+$$ as we do in the $$r\rightarrow \infty $$ setting; we can only do so up to the non-integrable term. To address the latter, we use transport estimates, which produce boundary terms weighted by a smallness parameter associated with derivatives of *h* but again not weighted by $$(\omega -m\upomega _+)^2$$. Notice that, unlike in the case of $$\mathcal {F}_\textrm{low,1a}$$, there can be no *a priori* smallness in the coupling with j<k in (4.19). However, for k<|s|, there is smallness in the coupling to k+1: in Lemmas 5.1 and 5.3, we see that the terms produced by this “forward” coupling either contain improved *r*-weights in a large $$|r^*|$$ region or a factor of $$|\omega |\ll 1$$. This smallness is what allows us to close our estimates.
- In $\mathcal {F}_\textrm{low,1c}$, we proceed as in $\mathcal {F}_\textrm{low,1b}$ but use the proximity to |a|=M, for which $w'=O(|r^*|^{-3})$ and there is a well-known correspondence between the $r^*\rightarrow \pm \infty$ ends to show that we can also connect the *h* and *y* currents near $r=r_+$ as in Lemma 6.39.

We start with the proof for $\mathcal {F}_\textrm{low,1a}$.

##### Proof of Proposition 6.40

Let $(\omega ,m,\Lambda )\in \mathcal {F}_\textrm{low,1a}$ be an admissible frequency triple, to that is assume all frequency parameters are bounded in terms of $\varepsilon _\textrm{width}$ and $\omega _\textrm{high}$, and $|\omega |\leqq \omega _\textrm{low}$, $a\leqq \tilde{a}_0$ are sufficiently small. We will employ virial currents of *h* and *y* type, as well as energy currents of *T* and *K* type.

*The main terms in *(4.21). Fix some $-R_2^*$ and $R_3^*$ to be large enough that $\mathcal {V}'<0$ for $r^*\geqq R_3^*/2$ and $\mathcal {V}'>0$ for $r^*\leqq R_2^*/2$, see Lemma 6.36. Assume that $\omega _\textrm{low}$ and $\tilde{a}_0$ are sufficiently small depending on some p∈(0,1) to be fixed that $\mathcal {V}-\omega ^2\geqq b w$ for $r^*\in [2e^{p^{-1}}R_2^*,2e^{p^{-1}}R_3^*]$. For $p,\tilde{p}, \epsilon \in (0,1)$ to be fixed, and for $\delta _I,\delta _H\in (0,1]$, we take *h* and *y* to be, respectively, $C^1$ and $C^0$ functions given by

$$\begin{aligned}  &   h=h^\textrm{left}+\mathbb {1}_{[R_2^*,R_3^*]}+h^\textrm{right}, \\  &   y= \frac{2\tilde{p}}{R_3^*} \left(|r^*|-\frac{R_3^*}{2}\right)\mathbb {1}_{[R_3^*/2,R_3^*]} +y^\textrm{right},\quad \hat{y}= -\frac{2\tilde{p}}{R_2^*} \left(r^*-\frac{R_2^*}{2}\right)\mathbb {1}_{[R_2^*,R_2^*/2]} +\hat{y}^\textrm{left}, \end{aligned}$$

where $h^\textrm{right}$ and $y^\textrm{right}$ take the formulas (6.44) and (6.45) in Lemma 6.39, $h^\textrm{left}$ is identical to $h^\textrm{right}(-r^*)$ but with $R_3^*$ replaced by $R_2^*$, $\hat{y}^\textrm{left}$ is identical to $-y^\textrm{right}(-r^*)$ but with $R_3^*$ replaced by $R_2^*$ and $\delta _I$ replaced by $\delta _H$. (Here and throughout the proof, we are using the convention in Notation 6.1.)

Though we can appeal to the case k=|s| of Lemma 6.39 here, we will instead choose to redo the computations in this simplified setting, as doing so is instructive for understanding the proof of that lemma and of the estimates remaining $\mathcal {F}_\textrm{low,1}$ subregimes. For $r^*\in [2e^{p^{-1}}R_2^*,R_2^*]\cup [R_3^*,2e^{p^{-1}}R_3^*]$,

$$\begin{aligned}&-\frac{(y\mathcal {V}+\hat{y} \hat{\mathcal {V}})'}{\tilde{p} } = \left(1-\frac{\epsilon }{|R_2^*|\hat{\mathcal {V}}(R_2^*)}\right)\mathcal {V}' \mathbb {1}_{[2e^{p^{-1}}R_2^*,R_2^*]} \\&\quad -\left(1-\frac{\epsilon }{(R_3^*)\mathcal {V}(R_3^*)}\right)\mathcal {V}' \mathbb {1}_{[R_3^*, 2e^{p^{-1}}R_3^*]} +\frac{\epsilon }{(r^*)^2}\,, \end{aligned}$$

so, given our choices of $R_2^*$ and $R_3^*$, we now fix ϵ to be sufficiently small so that the first two terms are positive. For $r^*\notin [2e^{p^{-1}}R_2^*, 2e^{p^{-1}}R_3^*]$,

$$\begin{aligned} -(y\mathcal {V})'&= -y\mathcal {V}'\left(1+\frac{y'}{y}\frac{\mathcal {V}}{\mathcal {V}'}\right) \geqq -y\mathcal {V}'\left(1-\frac{B\delta _I}{(2e^{p^{-1}}R_3^*)^{\delta _I}}\right)\geqq -\frac{1}{2}y\mathcal {V}'\,,\\ -(\hat{y}\hat{\mathcal {V}})'&= -\hat{y}\hat{\mathcal {V}}'\left(1+\frac{y'}{y}\frac{\hat{\mathcal {V}}}{\hat{\mathcal {V}}'}\right) \geqq -\hat{y}\hat{\mathcal {V}}'\left(1-\frac{B\delta _H}{|2e^{p^{-1}}R_2^*|^{\delta _H}}\right)\geqq -\frac{1}{2} \hat{y}\hat{\mathcal {V}}'\,, \end{aligned}$$

as long as $2e^{p^{-1}}R_2^*$ and $2e^{p^{-1}}R_3^*$ are sufficiently large, which can be attained my making *p* even smaller.

From Lemma 6.36, we see that, for $2e^{p^{-1}}R_2^* \leqq r^*\leqq 2e^{p^{-1}}R_3^*$, we have

$$\begin{aligned}&h(\mathcal {V}-\omega ^2)-\frac{1}{2}h''+ y'\omega ^2+\hat{y}'\omega _0^2-(y\mathcal {V}+\hat{y} \hat{\mathcal {V}})'\\&\quad \geqq \frac{3}{4} h(\mathcal {V}-\omega ^2)+ y'\omega ^2+\hat{y}'\omega _0^2+\frac{\epsilon -Bp}{(r^*)^2} \\&\quad \geqq \frac{3}{4} h(\mathcal {V}-\omega ^2)+ y'\omega ^2+\hat{y}'\omega _0^2\,, \end{aligned}$$

fixing some $\tilde{p}\in (0,1/4)$, as long as *p* is sufficiently small (depending on the already fixed ϵ). We fix *p* so that the two conditions above hold. Finally, now that all the constants we have introduced are fixed, we rescale our *h* and *y* currents so that y(∞)=1.

Adding the currents $Q^h$, $Q^y$ and $Q^{\hat{y}}$ thus produces a good bulk term, but it generates boundary terms with the wrong sign. As superradiance may occur in this frequency regime, we add localized Killing energy currents $E\chi _2 Q^T$ and $E\chi _1 Q^K$ for E≧3. Here, $\chi _1$ and $\chi _2$ are smooth functions taking values between 0 and 1 such that: $\chi _1=1$ for $r^*\leqq -1$, $\chi _1=0$ for $r^*\geqq 0$; $\chi _2=1$ for $r^*\geqq 1$, $\chi _2=0$ for $r^*\leqq 0$ and $\chi _2'=0$. Thus, we generate localization errors

$$\begin{aligned}&\int _{-\infty }^\infty E(\chi _1'\omega +\chi _2'\omega _0)\operatorname {Im}[\Psi '\overline{\Psi }]\textrm{d}r^*\\&\quad \leqq \int _{-\infty }^\infty \left(\frac{1}{2} h|\Psi |^2+B(\varepsilon _\textrm{width},\omega _\textrm{width})E^2\tilde{a}_0^2 h(\mathcal {V}-\omega ^2) |\Psi |^2\right)\textrm{d}r^*\\&\quad \leqq \frac{1}{2} \int _{-\infty }^\infty \left(h|\Psi |^2+ \frac{1}{2}h(\mathcal {V}-\omega ^2) |\Psi |^2\right)\textrm{d}r^*\,, \end{aligned}$$

if $\tilde{a}_0$ is sufficiently small depending on $\varepsilon _\textrm{width}$, $\omega _\textrm{width}$ and *E*.

By combining the above computations, we obtain

6.50 $$\begin{aligned}&(\omega -m\upomega _+)^2|\Psi (-\infty )|^2+\omega ^2|\Psi (+\infty )|^2+\nonumber \\&\qquad +\int _{-\infty }^{\infty } \left\rbrace \left(h+y'+\hat{y}'\right)|\Psi '|^2+\left[\frac{1}{2} h(\mathcal {V}-\omega ^2)+y'\omega ^2+\hat{y}' \omega _0^2-\frac{1}{2} y\mathcal {V}'-\frac{1}{2}\hat{y} \hat{\mathcal {V}}'\right]|\Psi |^2\right\lbrace \textrm{d}r^*\nonumber \\&\quad \leqq -\sum _{k=0}^{|s|-1}\int _{-\infty }^\infty \left\rbrace hw\operatorname {Re}[(ac_{s,k}^\textrm{id}+im ac_{s,k}^{\Phi })\uppsi _{(k)}\overline{\Psi }]\right.\nonumber \\&\left.\quad -2 \operatorname {Re}\left[\left(w(y+\hat{y})(ac_{s,k}^\textrm{id}+im ac_{s,k}^{\Phi })\right)'\uppsi _{(k)}\overline{\Psi }\right] \right\lbrace \textrm{d}r^* -\sum _{k=0}^{|s|-1}\int _{-\infty }^\infty 2 w (y+\hat{y}) {{\,\textrm{sign}\,}}s\nonumber \\&\qquad \operatorname {Re}\left[(ac_{s,k}^\textrm{id}+im ac_{s,k}^{\Phi })\overline{\Psi }\left(w\uppsi _{(k+1)}-i\omega \uppsi _{(k)}+\frac{iam}{r^2+a^2}\uppsi _{(k)}\right)\right]\textrm{d}r^*\nonumber \\&\qquad + \sum _{k=0}^{|s|-1}\int _{-\infty }^\infty Ew\left[(\omega -m\upomega _+)\chi _1+\omega \chi _2\right]\operatorname {Im}[(ac_{s,k}^\textrm{id}+im ac_{s,k}^{\Phi })\uppsi _{(k)}\overline{\Psi }]\textrm{d}r^*\nonumber \\&\qquad -\int _{-\infty }^\infty \left\rbrace 2(\hat{y}+y)\operatorname {Re}[\mathfrak G\overline{\Psi }']+h\operatorname {Re}[\mathfrak G\overline{\Psi }]-E\left[(\omega -m\upomega _+)\chi _1+\omega \chi _2\right]\operatorname {Im}[\mathfrak G\overline{\Psi }]\right\lbrace \textrm{d}r^*\,, \end{aligned}$$

where we have used Lemma 5.1. If s=0, the first two lines on the right hand side are absent and the proof is finished.

*The coupling terms in* (4.21). From now on, assume s≠0. We wish to estimate the first two lines on the right hand side of (6.50). By an application of Cauchy–Schwarz, see Lemma 6.39, we can control them by

$$\begin{aligned}&\tilde{\epsilon }\int _{-\infty }^{\infty } \left[\frac{1}{2} h(\mathcal {V}-\omega ^2)+y'\omega ^2+\hat{y}' \omega _0^2-\frac{1}{2} y\mathcal {V}'-\frac{1}{2}\hat{y} \hat{\mathcal {V}}'\right]|\Psi |^2\textrm{d}r^*\\&\qquad + \tilde{a}_0^2B(E,\varepsilon _\textrm{width},\omega _\textrm{width})\tilde{\epsilon }^{-1}\sum _{k=0}^{|s|}\int _{-\infty }^{\infty } w|\uppsi _{(k)}|^2 \textrm{d}r^*\,, \end{aligned}$$

where we fix $\tilde{\epsilon }$ to be small enough that we can absorb the first term into the left hand side of (6.50).

To control the outstanding terms, we appeal to Lemma 5.5. With c=-1/r if s<0 and $c=\frac{r-r_+}{r}$ if s>0, (5.12) becomes

$$\begin{aligned} \int _{-\infty }^\infty w|\uppsi _{(j)}|^2\textrm{d}r^*&\leqq \int _{-\infty }^\infty c'|\uppsi _{(k)}|^2\textrm{d}r^*\\&\leqq B\int _{-\infty }^\infty w|\uppsi _{(k+1)}|^2\textrm{d}r^*\leqq B\int _{-\infty }^\infty w|\uppsi _{(|s|-1)}|^2\textrm{d}r^*\,, \end{aligned}$$

for k<|s|-1. For k=|s|-1, with $c(r)=({{\,\textrm{sign}\,}}s) r^{-1}$, we obtain

$$\begin{aligned} \int _{-\infty }^\infty w|\uppsi _{j}|^2\textrm{d}r^*&\leqq B|\uppsi _{(|s|-1)}(-\infty )|^2+ B\int _{-\infty }^\infty \frac{w}{r^2+a^2}|\Psi |^2 \textrm{d}r^*\,. \end{aligned}$$

In the above, we are making essential use of the outgoing boundary conditions of $\uppsi _{(k)}$. Thus,

$$\begin{aligned}&\tilde{a}_0^2B(E,\varepsilon _\textrm{width},\omega _\textrm{width})\sum _{k=0}^{|s|}\int _{-\infty }^{\infty } w|\uppsi _{(k)}|^2 \textrm{d}r^*\\&\quad \leqq \tilde{a}_0^2B(E,\varepsilon _\textrm{width},\omega _\textrm{width})\\&\qquad \times \int _{-\infty }^{\infty }\left[\frac{1}{2} h(\mathcal {V}-\omega ^2)+y'\omega ^2+\hat{y}' \omega _0^2-\frac{1}{2} y\mathcal {V}'-\frac{1}{2}\hat{y} \hat{\mathcal {V}}'\right]|\Psi |^2\textrm{d}r^*\\&\quad \qquad +\tilde{a}_0^2B(E,\varepsilon _\textrm{width},\omega _\textrm{width}) |\uppsi _{(|s|-1)}(-\infty )|^2\,, \end{aligned}$$

where the first term on the right hand side may also be absorbed into the left hand side of (6.50) provided that $\tilde{a}_0$ is sufficiently small depending on all the parameters on which the constant *B* depends. Finally, for s>0, we can relate the boundary term with that of $\uppsi _{(0)}$ by Lemma 6.38. Alternatively, if for k=0,⋯,|s| the inhomogeneity $\mathfrak {G}_{(k)}$ enjoy sufficiently strong decay towards $r\rightarrow r_+$ that Lemma 4.10 applies, then

$$\begin{aligned} |\uppsi _{(|s|-1)}(-\infty )|^2\leqq (\omega -m\upomega _+)^2|\Psi (-\infty )|^2 \end{aligned}$$

so, by the smallness of $\tilde{a}_0$, we can also absorb the boundary term errors by the left hand side of (6.50).

To conclude the proof, we set $\gamma _{1a}=\tilde{a}_0$. □

The constructions of *h* and *y* currents in the above proof assume a certain measure of symmetry between the $r^*\rightarrow \pm \infty$ ends. In the case where *a* is not a small parameter and thus the coupling terms on the right hand side of (4.21) are not lower order, we find this symmetry in the case m=0 by restricting to nearly extremal Kerr spacetimes:

##### Proof of Proposition 6.42

Let $(\omega ,m,\Lambda )\in \mathcal {F}_\textrm{low,1c}$ be an admissible frequency triple, to that is assume all frequency parameters are bounded in terms of $\varepsilon _\textrm{width}$ and $\omega _\textrm{high}$, m=0, and $|\omega |\leqq \omega _\textrm{low}$, $0\leqq M-a<\tilde{a}_1$ are sufficiently small. We will employ virial currents of type *h* and *y*, as well as a global *T* Killing energy current, for each of the radial ODEs (4.19).

For some $R_2^*<0$, let p∈(0,1) be defined in terms of *M* and $\tilde{a}_1$ by

$$\begin{aligned} \sqrt{M^2-(M-\tilde{a}_1)^2}= pe^{-2/p}, \end{aligned}$$

so that choosing *p* is equivalent to choosing $\tilde{a}_1$. Define $R_1=r_++(R_2-r_+)e^{-1/p}$ and $R_{0}=r_++(R_1-r_+)/2$. Let $R_3^*>1$ be another constant to be fixed. We take $R_3^*$, $|R_2^*|$ and 1/*p* to be sufficiently large so that, if $\omega _\textrm{low}$ is sufficiently small: (a) $\mathcal {V}_{(k)}'<0$ for $r^*\geqq R_3^*/2$ and $\mathcal {V}_{(k)}'<0$ for $r^*\leqq R_2^*/2$, see Lemma 6.36(ii)–(iii); (b)  for $r^*\in [R_0^*,2e^{p^{-1}}R_3^*]$.

We set

$$\begin{aligned}  &   h_{(k)}=h_{(k)}^{\textrm{left}}+\mathbb {1}_{[R_2^*,R_3^*]}+h_{(k)}^{\textrm{right}}, \\  &   y_{(k)}=\frac{2\tilde{p}}{R_3^*}\left(r^*-\frac{R_3^*}{2}\right)\mathbb {1}_{[R_3^*/2,R_3^*]}+y_{(k)}^{\textrm{right}},\\  &   \hat{y}_{(k)}=-\frac{2\tilde{p}}{|R_2^*|}\left(|r^*|-\frac{|R_2^*|}{2}\right)\mathbb {1}_{[R_2^*,R_2^*/2]}+\hat{y}_{(k)}^{\textrm{left}}, \end{aligned}$$

for some $\tilde{p}>0$, where $h_{(k)}^{\textrm{right}}$ and $y_{(k)}^{\textrm{right}}$ are given by (6.44) and (6.45); we define $h_{(k)}^{\textrm{left}}$ and $y_{(k)}^{\textrm{left}}$ below.

*The region close to* r=∞. Possibly by making $R_3^*$ larger, we can take ϵ, *p* and $\omega _\textrm{low}$ to be sufficiently small, depending on $\tilde{p}$ and considering the constraints of Lemma 6.39, that the conclusion holds for $r\in [R_3^*,\infty )$ and with $\chi _2\equiv 1$.

*The region close to* $r=r_+$. Now, we wish to define $h_{(k)}^\textrm{left}$ and $y_{(k)}^\textrm{left}$ so that similar estimates to those of Lemma 6.39 hold for $r^*\leqq R_2^*$. Our choices of the dependence of $R_1$ and $R_0$ on *p* are critical to this endeavor: they ensure that, in practice, the present regime can be treated as if the Kerr black hole were exactly extremal, since

6.51 $$\begin{aligned} \Delta (r-M)^{-2} = 1+\left(\frac{\sqrt{M^2-a^2}}{r-M}\right)^2 =1+O(p)\,, \end{aligned}$$

for $r\geqq R_0$ as p→0, assuming $|R_2^*|$ is sufficiently large. Indeed, let us set

$$\begin{aligned} h_{(k)}^{\textrm{left}}&= \frac{1}{1-2p(1-\log 2)}\left[\frac{r-M}{R_2-M}+p\log \left(\frac{r-M}{R_2-M}\right)\right]\mathbb {1}_{[R_1,R_2]} \\&\qquad +\left[\frac{(1+p(\log 4-1))}{(R_2-r_++M-r_+)}\left(\frac{(R_2-M)(r-r_+)}{(r-M)}-\frac{(R_2-r_+)(r-M)}{(R2-M)}\right)\right]\mathbb {1}_{[R_1,R_2]}\\&\qquad + \left[C_{1}^-\frac{r-r_+}{R_1-r_+}\frac{R_1-M}{r-M}+C_{2}^-\frac{R_1-M}{r-M}+C_{3}^-\log \left(\frac{R_1-M}{r-M}\right)+C_{4}^-\frac{r-M}{R_1-M}\right]\mathbb {1}_{[R_{0},R_1]}\,, \end{aligned}$$

and the constants $C_{1}^-$ up to $C_{4}^-$ are chosen so that *h* is $C^1$: for sufficiently large $|R_2^*|$,

$$\begin{aligned}  &   C^-_{1}=\frac{(\log 8-1)h(R_1)-(5\log 2-3)(R_1-M)h'(R_1)}{\log 8 -2}+o(p),\\  &   C_{2}^-=\frac{h(R_1)-(1-\log 2)(R_1-M)h'(R_1)}{\log 8 -2}+o(p) , \\  &   C_{3}^-=-\frac{[3h(R_1)-(R_1-M)h'(R_1)]}{\log 8 -2},\\  &   C_{4}^-=-\frac{2[h(R_1)-(\log 4-1)(R_1-M)h'(R_1)]}{\log 8 -2}, \end{aligned}$$

where the first two lines are obtained in the limit p→0. One can verify that, for $r^*\leqq R_2^*$,

$$\begin{aligned} h_{(k)}''+(|s|-k)\frac{w'}{w}h_{(k)}' \leqq \frac{Bp}{(r^*)^2}\,, \end{aligned}$$

due to (6.51).

We then set $\hat{y}_{(k)}^{\textrm{left}}$ to be identical to $-y_{(k)}^{\textrm{right}}(-r^*)$ but with $R_3^*$ replaced by $R_2^*$, $-2e^{p^{-1}R_3^*}$ replaced by $R_0^*$, and $\delta _I$ replaced by $\delta _H$. We may now fix $R_2$ as a function of $R_3^*$ so as to ensure that $|y(\infty )|=|\hat{y}(-\infty )|$; note $R_2^*\rightarrow -\infty$ as $R_3^*\rightarrow \infty$. It is easy to see that, repeating the arguments of Lemma 6.39, we can deduce that, if we take $R_3^*$ even larger, then ϵ possibly smaller depending on $R_3^*$, then *p* possibly smaller depending on $\delta _H$, we can show that6.52We fix *p* and ϵ as functions of $R_3^*$, $\delta _H$, $\delta _I$ and $\tilde{p}$ so that this holds.

*A bounded* $|r^*|$ *region.* Finally, we take $r\in [R_2^*,R_3^*]$ and consider the last four lines in (6.52). We have6.53if we choose $\tilde{p}$ to be sufficiently small; we fix $\tilde{p}$ so that this is the case.

Let us now turn to errors associated with the all the currents we have introduced, see Lemmas 5.1 and 5.3 for the formulas. We start with the coupling to k+1: for $E\geqq 2\max \{y_{(k)}(\infty ),-\hat{y}_{(k)}(-\infty )\}$For the coupling to j<k, we haveMaking $\omega _\textrm{low}$ even smaller, we can take $\omega _\textrm{low}^2(|R_2^*|+R_3^*)^2\leqq B$.

By combining these estimates with (6.52), we obtain6.54*Conclusion.* In estimate (6.54) the hierarchical structure is clear: we can make the coupling to k+1 arbitrarily small while maintaining the coupling to j<k bounded. Indeed, for $E\geqq 2\max \{y_{(k)}(\infty ),-\hat{y}_{(k)}(\infty )\}+1$, $R_3^*$ (and thus $-R_2^*$) sufficiently large and $\omega _\textrm{low}$ sufficiently small, depending on $R_3^*$ and *E*, so that

$$\begin{aligned} \frac{1}{(R_3^*)^2}+(R_2-r_+)^2\ll 1, \quad \omega _\textrm{low}^2\left(1+\frac{E^2}{y_{(k)}(\infty )}\right)\ll 1, \end{aligned}$$

at repeating the estimate (6.54) for all levels of the transformed variables up to *k*, we obtainWe fix $\omega _\textrm{low}$ as a function of $R_3^*$ and *E* so that this holds. Thus, for k=|s|,We can now fix $R_3^*$ to be sufficiently large so that all the above estimates hold, and then rescale the *y* and $\hat{y}$ currents so that $y(\infty )=-\hat{y}(-\infty )=1$. □

For subextremal spacetimes, the symmetry between $r^*\rightarrow \pm \infty$ is absent even if m=0. Nevertheless, we will show that the subextremality will allow us to control the region near $r=r_+$.

##### Proof of Proposition 6.41

Let $(\omega ,m,\Lambda )\in \mathcal {F}_\textrm{low,1b}$ be an admissible frequency triple, to that is assume all frequency parameters are bounded in terms of $\varepsilon _\textrm{width}$ and $\omega _\textrm{high}$, m=0, $\tilde{a}_0\leqq a<M-\tilde{a}_1$, and $|\omega |\leqq \omega _\textrm{low}$ are sufficiently small. We will employ virial currents of type *h* and *y*, as well as a global *T* Killing energy current, for each of the radial ODEs (4.19).

Let $R_3^*>1$ and $R_2^*>1$ and 1/p>0 be sufficiently large so that, if $\omega _\textrm{low}$ is sufficiently small: (a) $\mathcal {V}_{(k)}'<0$ for $r^*\geqq R_3^*/2$ and $\mathcal {V}_{(k)}'<0$ for $r^*\leqq R_2^*/2$, see Lemma 6.36(ii)–(iii); (b) $\mathring{\mathcal {V}}_{(k)}-\omega ^2\geqq bw$ for $r^*\in [R_0^*,2e^{p^{-1}}R_3^3*]$. For $\tilde{p},\epsilon >0$, we set

$$\begin{aligned} h_{(k)}= &   h_{(k)}^{\textrm{left}}+\mathbb {1}_{[R_2^*,R_3^*]}+h_{(k)}^{\textrm{right}}, \\ y_{(k)}= &   \frac{2\tilde{p}}{R_3^*}\left(r^*-\frac{R_3^*}{2}\right)\mathbb {1}{[R_3^*/2,R_3^*]}+y_{(k)}^{\textrm{right}},\\ \hat{y}_{(k)}= &   -\frac{2\tilde{p}}{R_2^*}\left(r^*-\frac{R_2^*}{2}\right)\mathbb {1}{[R_2^*,R_2^*/2]}+\hat{y}_{(k)}^{\textrm{left}}, \end{aligned}$$

where $h_{(k)}^{\textrm{right}}$ and $y_{(k)}^{\textrm{right}}$ are given by (6.44) and (6.45), $h_{(k)}^\textrm{left}$ is identical to $h_{(k)}^\textrm{right}(-r^*)$ but with $R_3^*$ replaced by $R_2^*$ and $\hat{y}_{(k)}^\textrm{left}$ is identical to $-y_{(k)}^\textrm{right}(-r^*)$ but with $R_3^*$ replaced by $R_2^*$ and $\delta _I$ replaced by $\delta _H$.

*A bounded* $|r^*|$. In these definitions, we have parameter a parameter $\tilde{p} \in (0,1)$, which we fix, as a function of $R_3^*$, so thatfor $-R_2^*$ and $R_3^*$ sufficiently large. It is easy to see that the estimates for coupling error terms for $r^*\in [R_2^*,R_3^*]$ in the proof of Proposition 6.42 hold in this case too.

*The region close to* r=∞. Possibly by making $R_3^*$ larger, we can take ϵ, *p* and $\omega _\textrm{low}$ to be sufficiently small, considering the constraints of Lemma 6.39, that the conclusion holds for $r\in [R_3^*,\infty )$ and with $\chi _2\equiv 1$.

*The region close to* $$r=r_+$$. We would like the $$r\rightarrow r_+$$ end to be similar to that of $$r\rightarrow \infty $$ considered in Lemma 6.39, as in Proposition 6.42. The differences are related to the fact that $w^{\prime }=O\left(w\right)$ for a<M, with a constant depending on M-a. Indeed, this means that, by making (6.54)

6.55 $$\begin{aligned} \left(h_{(k)}''+(|s|-k)\frac{w'}{w}h_{(k)}'\right) \mathbb {1}_{(-\infty , R_2^*]}\leqq &   \frac{Bp }{(r^*)^2}\mathbb {1}_{[2e^{p^{-1}}R_2^*,R_2^*]}\nonumber \\  &   +\frac{Bp(|s|-k)}{|r^*|}\mathbb {1}_{[2e^{p^{-1}}R_2^*,R_2^*]}, \end{aligned}$$

where we note that the last term is not integrable. Nevertheless, it is not hard to see that one can repeat the arguments given in the proof of Proposition 6.42 in the $r^*\leqq R_2^*$ region: if $-R_2^*$ is sufficiently large, ϵ is made even smaller depending on $R_2^*$, *p* is made even smaller depending on $R_2^*$, ϵ and $\delta _H$, and $\omega _\textrm{low}$ made even smaller depending on $R_2^*$, then we can treat the first (6.55) and the coupling terms from the currents in essentially the same fashion as in the proof of Proposition 6.42. (A small difference is that, while in the latter proof it was necessary to apply a transport estimate to improve the *r*-weights as $r\rightarrow r_+$ on certain terms, analogously to the large $r^*$ region, see (6.48) in the proof of Lemma 6.39, in the setting of $a\leqq \tilde{a}_1$, this improvement is not needed, as $\mathcal {V}_{(k)}'=O(w)$ for a<M with a constant depending on M-a.) Thus, (6.54) holds with an additional bulk term coming from the last term in (6.55), to that is, we have6.56We fix ϵ as a function of $R_3^*$ and $R_2^*$ so that the above holds.

**Fig. 8:**
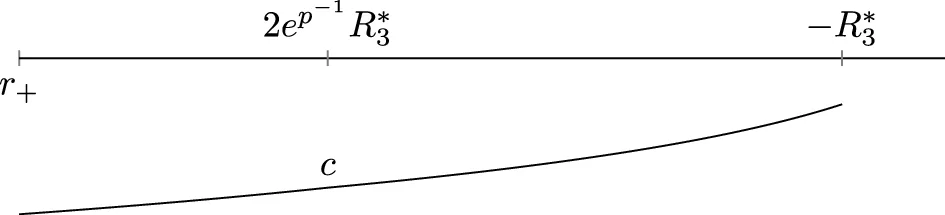
A weight *c*(*r*) for application of basic estimate (5.12) from Lemma 5.5 in the analysis of $\mathcal {F}_\textrm{low,1b}$

Let us turn to controlling the first term on the right hand side of (6.56), using the subextremality assumption that $a\leqq \tilde{a}_1<M$. Let us introduce a continuous, piecewise $C^1$ function given by

$$\begin{aligned} c&=-\log |r^*|\mathbb {1}_{[2e^{p^{-1/2}}R_2^*,R_2^*]}\\&\quad +\left(\frac{1}{|r^*|}-\frac{1}{2e^{p^{-1/2}}|R_2^*|}-\log (2e^{p^{-1/2}}|R_2^*|)\right)\mathbb {1}_{(-\infty ,2e^{p^{-1}/2}R_2^*]}\,,\\ c'&=\frac{1}{|r^*|}\mathbb {1}_{[-2e^{p^{-1/2}}R_2^*,R_2^*}+\frac{1}{(r^*)^2}\mathbb {1}_{(-\infty ,2e^{p^{-1/2}}R_2^*]}\,, \end{aligned}$$

see also Fig. 8. As long as $R_2^*$ is sufficiently large depending on $\tilde{a}_1$, $wc^2/c'$ is a decreasing function. If moreover $R_2^*$ is sufficiently large depending on *p*, the latter is bounded above independently of the parameters:

$$\begin{aligned} \frac{wc^2}{c'}\mathbb {1}_{(-\infty , -R_2^*]}&\leqq B\exp \left(-\frac{\sqrt{M^2-a^2}}{Mr_+}|r^*|\right)|r^*|^2\left(\log (2e^{p^{-1/2}}R_2^*)\right)^2\mathbb {1}_{(-\infty , R_2^*]}\\&\leqq B\exp \left(2\log (R_2^*)\left(1+\frac{\log 2}{\sqrt{p}}\right)-R_2^*\sqrt{\frac{\tilde{a}_1}{M}}\right)\mathbb {1}_{(-\infty , R_2^*]} \leqq B\sqrt{p}\,. \end{aligned}$$

We take $e^{b/\sqrt{p}}\leqq R_2^*\leqq e^{B/\sqrt{p}}$ so that the above holds for sufficiently small *p* depending on $\tilde{a}_1$ and, moreover, we have

$$\begin{aligned} p\log (2e^{p^{-1/2}}R_2^*) =\sqrt{p}+p\log (2R_2^*) \leqq B \sqrt{p} \,. \end{aligned}$$

Then, from Lemma 5.5, for 0≦j<|s|, we obtain

6.57 $$\begin{aligned} \int _{2e^{p^{-1}}R_2^*}^{R_2^*}\frac{p}{|r^*|}|\uppsi _{(k)}|^2\textrm{d}r^*&\leqq \int _{-\infty }^{R_2^*} p c'\left|\uppsi _{(k)}\right|^2\textrm{d}r^*\nonumber \\&\leqq Bp\log (2e^{p^{-1}}R_2^*)|\uppsi _{(k+1)}(-\infty )|^2\nonumber \\&\quad + Bp\int _{-\infty }^{R_2^*} w \frac{wc^2}{c'}\left|\uppsi _{(k+1)}\right|^2\textrm{d}r^*\nonumber \\&\leqq B\sqrt{p} |\uppsi _{(k+1)}(-\infty )|^2 + \int _{-\infty }^\infty pw\mathbb {1}_{(-\infty ,R_2^*]} \left|\uppsi _{(k+1)}\right|^2\textrm{d}r^*\,. \end{aligned}$$

Take $E\geqq 2\max \{y_{(k)}(\infty ),-\hat{y} _{(k)}(-\infty )\}$. We deduce that if $R_3^*$ and $|R_2^*|$ are sufficiently large, *p* is sufficiently small, and $\omega _\textrm{low}$ is sufficiently small depending on *E*, *p*, $R_3^*$ and $|R_2^*|$ that

$$\begin{aligned} \omega _\textrm{low}^2\left(1+\frac{E^2}{\max \{y_{(k)}(\infty ),-\hat{y}_{(k)}(\infty )\}}\right)\ll 1, \quad \omega _\textrm{low}^2(|R_3^*|^2+|R_3^*|^2)\ll 1, \end{aligned}$$

then by combining (6.56) and (6.57) for all levels of the transformed variables up to *k*, we have6.58*The boundary terms*. Let us now consider the case s>0, where the boundary terms

$$\begin{aligned} B\sqrt{p}\sum _{j=0}^{k}|\uppsi _{(j)}(-\infty )|^2 \end{aligned}$$

in (6.58) prevent us from concluding. Indeed, notice that, because the boundary term does not have a frequency weight $\omega ^2$ and we are in a regime where the latter is small, *a priori* we cannot absorb it by the left hand side of (6.58). However, by Lemma 6.38, we can control this by

$$\begin{aligned} B\sqrt{p}\left\rbrace \sum _{j=0}^{|s|}\left|\frac{\mathfrak {G}_{(j)}}{w}\right|^2(-\infty )+|\uppsi _{(0)}(-\infty )|^2\right\lbrace \,. \end{aligned}$$

or, if $\mathfrak {G}_{(k)}=O(\Delta ^{|s|-k})$ as $r\rightarrow r_+$ for all *k*, by

$$\begin{aligned} B\sqrt{p}\omega ^2|\Psi (-\infty )|^2\,. \end{aligned}$$

In the latter case, for $\sqrt{p}$ even smaller, we can absorb this term into the left hand side of (6.58) with k=|s|. In the former case, we set $\gamma _{1b}=\sqrt{p}$.

Finally, we can fix $R_2^*$ in terms of $R_3^*$ to be sufficiently small, and rescale all the currents, so that $y(\infty )=-\hat{y}(-\infty )=1$. □

#### Low time frequency outside of axisymmetry for nonzero black hole angular momentum

This section concerns the frequency range $\mathcal {F}_\textrm{low,2}$. We will prove the following result:

##### Proposition 6.43

(Estimates in $\mathcal {F}_\textrm{low,2}$) Fix $s\in \{0,\pm 1,\pm 2\}$ and M>0. Then, for all $\delta _H\in (0,1)$ and $\delta _I\in (0,1]$, for all $\omega _\textrm{high}>0$, for all $\varepsilon _\textrm{width}>0$, for all $\tilde{a}_0>0$, for all $E_W\geqq 0$, for all E>0 sufficiently large, for all $\omega _\textrm{low}$ sufficiently small depending on $\omega _\textrm{high}$, $\varepsilon _\textrm{width}$, $\tilde{a}_0$, $E_W$ and *E*, and for all $(a,\omega ,m,\Lambda ) \in \mathcal {F}_\textrm{low,2}(\omega _\textrm{high},\varepsilon _\textrm{width},\omega _\textrm{low},\tilde{a}_0)$, there exist functions $y_{(k)}$, $\hat{y}_{(k)}$, $h_{(k)}$, $\chi _1$ and $\chi _2$ satisfying the uniform bounds for all $(a,\omega ,m,\Lambda ) \in \mathcal {F}_\textrm{low,2}(\omega _\textrm{high},\varepsilon _\textrm{width},\omega _\textrm{low},\tilde{a}_0)$, there is exist functions y≧0, $\hat{y}\geqq 0$, h≧0, $0\leqq \chi _1\leqq 1$ and $0\leqq \chi _2\leqq 1$ satisfying the bounds (6.3) and a function $\gamma _2(x)$ with the property that $\gamma _2(x)\rightarrow 0$ as x→0 such that

$$\begin{aligned}&\omega ^2\left|\Psi (\infty )\right|^2+(\omega -m\upomega _+)^2\left|\Psi (-\infty )\right|^2 \\&\qquad + b(\tilde{a}_0,\delta _H,\delta _I)\int _{-\infty }^\infty \frac{\Delta }{r^2}\Big [\left(1-Mr^{-1}\right)^{\delta _H-1}r^{-1-\delta _I}|\Psi '|^2\\&\qquad \qquad \qquad \quad \qquad \quad \qquad \qquad +\left(\frac{(r-M)}{r^4}\Lambda +\frac{(1-Mr^{-1})^{\delta _H-1}}{r^{1+\delta _I}}\right)|\Psi |^2\Big ]\textrm{d}r^*\\&\quad \leqq -\int _{-\infty }^\infty \left\rbrace 2(y+\hat{y})\operatorname {Re}\left[\mathfrak {G}\overline{\Psi }'\right]+h\operatorname {Re}\left[\mathfrak {G}\overline{\Psi }\right]-E\chi _2\omega \operatorname {Im}\left[\mathfrak {G}\overline{\Psi }\right]\right\lbrace \textrm{d}r^* \\&\qquad -{{\,\textrm{sign}\,}}s \frac{1}{2}(-1)^{|s|}\int _{-\infty }^\infty E_W(\omega -m\upomega _+)\chi _1 \operatorname {Im}\left[\Big (\sum _{k=0}^{|s|}p_{s,|s|,k}(r){\mathfrak {G}}_{(k)}\Big )\Big (\sum _{k=0}^{|s|}p_{s,|s|,k}(r)\overline{\uppsi _{(k)}}\Big )\right]\textrm{d}r^*\\&\qquad + \frac{1}{2}(-1)^{|s|}\sum _{j=1}^{|s|}\int _{-\infty }^\infty E_W\chi _1'\Big \{ \Big ((r^2+a^2)^{|s|-j+1/2}\sum _{k=0}^{j-1}p_{s,j-1,k}(r)\frac{{\mathfrak {G}}_{(k)}}{w}\Big )\\&\qquad \qquad \quad \qquad \qquad \qquad \qquad \quad \times \overline{\left(\frac{\mathcal {L}}{w(r^2+a^2)}\right)^{|s|-j}\Big ((r^2+a^2)^{-1/2}\sum _{k=0}^{|s|}p_{s,|s|,k}(r)\uppsi _{(k)}\Big )}\Big \}\textrm{d}r^*\\&\qquad +B(\varepsilon _\textrm{width},\omega _\textrm{high}) \sum _{k=0}^{|s|-1}\left|\int _{-\infty }^\infty \left\rbrace 2(y_{(k)}+\hat{y}_{(k)})\operatorname {Re}\left[\mathfrak {G}_{(k)}\overline{\uppsi _{(k)}}'\right]+h_{(k)}\operatorname {Re}\left[\mathfrak {G}_{(k)}\overline{\uppsi _{(k)}}\right]\right\lbrace \textrm{d}r^*\right|\\&\qquad +B(\varepsilon _\textrm{width},\omega _\textrm{high}) \sum _{k=0}^{|s|-1}\left|\int _{-\infty }^\infty E\chi _2\omega \operatorname {Im}\left[\mathfrak {G}_{(k)}\overline{\uppsi _{(k)}}\right]\textrm{d}r^*\right| \\&\qquad +\gamma _2(\omega _\textrm{low})B(\varepsilon _\textrm{width},\omega _\textrm{high})\Big (|\uppsi _{(0)}(-\infty )|^2+\sum _{k=0}^{|s|-1}\left|\frac{\mathfrak {G}_{(k)}}{w}\right|^2(-\infty )\Big )\mathbb {1}_{\{s> 0\}}\,, \end{aligned}$$

where $p_{s,k,j}(r)$ are smooth polynomials of degree k-jwhich are regular at $r=r_+$. If $\mathfrak {G}_{(k)}=O(\Delta ^{|s|-k+1})$ as $r\rightarrow r_+$ for all k=0,⋯,|s|, we can drop the last term from the right hand side by assuming $E_W>0$. Note that we can include $\delta _H=1$ if we restrict the statement to $a\leqq a_0<M$.

**Fig. 9:**
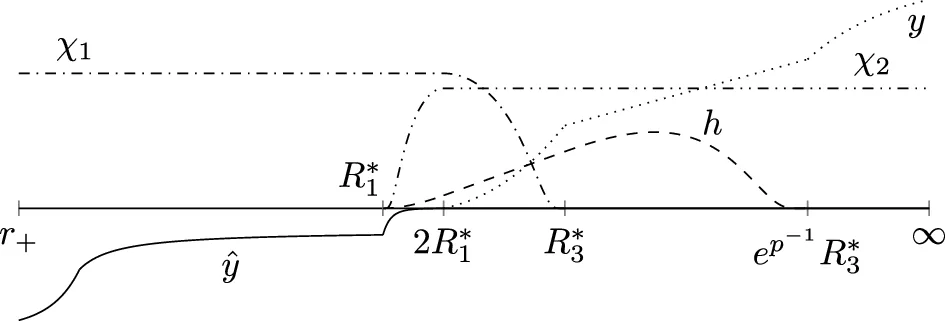
Sketch of currents in the proofs of Proposition 6.43

We start by briefly recalling the strategy to prove Proposition 6.43, which is best illustrated by Fig. 9. As the coupling terms on the right hand side of (4.21) are manifestly not small, our current constructions are applied to each of the radial ODEs (4.19). In $\mathcal {F}_\textrm{low,2}$, $\omega _0\geqq b(\tilde{a}_0)>0$ which, fimilarly to what we have seen in the time dominated regimes of Section 6.3.5, can be exploited by using a $\hat{y}$ multiplier which exponentially decays as $r\rightarrow \infty$. This current generates a boundary term at $r^*=-\infty$ that we must control with an energy current which is, as superradiance can occur in $\mathcal {F}_\textrm{low,2}$, localized to a bounded $|r^*|$ region. To absorb the localization errors, we make use of an *h* current, noting that $\mathcal {V}_{(k)}-\omega ^2$ is positive in a compact $|r^*|$ region by Lemma 6.36. The *h* current itself produces localization errors: to start if off, we make sure it does not grow too fast compared with the exponential $\hat{y}$; to take it down to 0, we connect it with another *y* current exploiting the sign of $\mathcal {V}_{(k)}'$ for large *r* as in Lemma 6.39.

In the above description, we have glossed over two subtle points. The first, which is already present when s=0, is that if *h* starts off being tied to $\hat{y}$, then taking it to be constant after that transitional regime will not allow us to absorb any energy localization errors. Indeed, the size of the errors (which are proportional to $\omega _0$ and not ω!) is comparable to the size of $\hat{y}(-\infty )$ which is must larger than one could attain by leveling off such *h* in a large *r* region (where $\hat{y}$ is very small). However, since the localization errors associated to *h* are proportional to

$$\begin{aligned} h''+(|s|-k)\frac{w'}{w}h'\,, \end{aligned}$$

we can allow *h* to grow as fast as $r^{2(|s|-k)+1}$ without any negative consequences for the bulk term generated by this current. This growth turns out to be *exactly* what is needed for the localization errors associated with energy currents. We are thus led to consider the second subtle point: what one means by energy current. We have seen in Lemma 6.39 that for boundary terms at r=∞ we can use a Killing energy current: even if this is not a conserved quantity, the errors it generates by interacting with coupling terms on the right hand side of (4.19) contain a factor of ω, which is small. Turning to the $r=r_+$ boundary terms, we find that the *K*-Killing coupling errors would come with a factor $\omega _0$, which is not small. This crucial difference leads us to use the *K* type Teukolsky–Starobinsky energy current, which is exactly conserved if $\mathfrak {G}_{(k)}\equiv 0$, instead.

##### Proof of Proposition 6.43

Let $(\omega ,m,\Lambda )$ be an admissible frequency triple such that $(\omega ,m,\Lambda )\in \mathcal {F}_\textrm{low,2}$, to that is assume all frequency parameters are bounded above in terms of $\varepsilon _\textrm{width}$ and $\omega _\textrm{high}$, |m|≧1 and $a>\tilde{a}_0$. Let $\omega _0:=|\omega -m\upomega _+|>0$. We will apply virial currents of *h* and *y* type, as well as energy currents of *T* and *K* type, to the radial ODEs (4.19).

In our construction of virial currents, we will introduce, and later fix, large positive constants *C* and $R_1^*,R_2^*,R_3^*$ as well as small positive parameters *p*, $\hat{p}$, $\tilde{p}$ and ϵ.

*The* $\hat{y}$ *current.* For some $R_0^*> 1$ and C>1 to be fixed, we set

$$\begin{aligned} \hat{y}(r^*)&=-\exp \left[-C(r-r_+)\right]\mathbb {1}_{[-R_0^*,R_0^*]}+\frac{\hat{y}(R_0^*)(R_0^*)^{\delta _I}}{(r^*)^{\delta _I}}\mathbb {1}_{(R_0^*,\infty )}\\&\qquad +\hat{y}(-R_0^*)\left[1+\frac{1}{(R_0^*)^{\delta _H}}-\frac{1}{(-r^*)^{\delta _H}}\right]\mathbb {1}_{(-\infty ,-R_0^*)}\,. \end{aligned}$$

With this choice, if $R_0$ is sufficiently large, we have

6.59 $$\begin{aligned} \left|\frac{w\hat{y}}{\hat{y}'}\right|&\leqq B\frac{\Delta |r^*|^{1+\delta _H}}{\delta _{H}}\mathbb {1}_{(-\infty ,-R_0^*]}+\frac{B}{C}\mathbb {1}_{[-R_0^*,R_0^*]}+\frac{B\delta _I}{(R_0^*)^3}\mathbb {1}_{[R_0^*,\infty )}\leqq \frac{B}{C}\,, \end{aligned}$$

where the conclusion follows from the following assumptions: if $a\leqq a_0<M$, for $\delta _H\in (0,1]$, we take $R_0^*$ to be sufficiently large depending on $\omega _0$, $\delta _I$, $\delta _H$, $a_0$ and *C*; otherwise if a≦M is more general, we restrict $\delta _H\in (0,1)$ and we take $R_0^*$ to be sufficiently large depending on $\omega _0$, $\delta _H$, $\delta _I$ and *C*.

From (6.59), we can immediately deduce three inequalities: first,

$$\begin{aligned} -\int _{-\infty }^\infty (\hat{y} \hat{\mathcal {V}}_{(k)})'|\uppsi _{(k)}|^2\textrm{d}r^*&= \int _{-\infty }^\infty 2 \hat{y} \hat{\mathcal {V}}_{(k)}\operatorname {Re}[\uppsi _{(k)}'\overline{\uppsi _{(k)}}]\textrm{d}r^*\\&\leqq \int _{-\infty }^\infty \frac{1}{16} \hat{y}'\left[|\uppsi _{(k)}'|+\omega _0^2|\uppsi _{(k)}|\right]\textrm{d}r^*\,; \end{aligned}$$

second, for j<k and assuming s≠0, we have

$$\begin{aligned} 2\hat{y}w\operatorname {Re}[ac_{s,k,j}^\textrm{id}\uppsi _{(j)}\overline{\uppsi _{(k)}'}]\leqq \frac{1}{32} \hat{y}'|\uppsi _{(k)}'|^2+\frac{B}{C}\hat{y}'\omega _0^2|\uppsi _{(j)}|^2,; \end{aligned}$$

third, if s≠0, then we also have

$$\begin{aligned}&2\hat{y} (|s|-k)w'\left[|\uppsi _{(k+1)}|^2-\left(\omega -\frac{am}{r^2+a^2}\right)\operatorname {Re}[\overline{\uppsi _{(k)}}\uppsi _{(k+1)}]\right]\\&\qquad +\int _{-\infty }^\infty 2\hat{y} (|s|-k)\frac{4am rw}{r^2+a^2}{{\,\textrm{sign}\,}}s \operatorname {Im}[\uppsi _{(k)}\overline{\uppsi _{(k)}'}]\textrm{d}r^*\\&\quad \leqq \frac{1}{32} \hat{y}'\left[|\uppsi _{(k)}'|^2+\omega _0^2|\uppsi _{(k)}|\right]\textrm{d}r^* + \frac{B(|s|-k)^2}{C} \hat{y}'\omega _0^2|\uppsi _{(k+1)}|\textrm{d}r^*\,; \end{aligned}$$

as long as *C* is sufficiently large depending on $\varepsilon _\textrm{width}$, $\omega _\textrm{high}$ and $\tilde{a}_0$.

From Lemma 5.1, we thus obtain

6.60 $$\begin{aligned}&\int _{-\infty }^\infty \frac{5}{8}\hat{y}'\left[|\uppsi _{(k)}'|^2+\omega _0^2|\uppsi _{(k)}|^2\right]\textrm{d}r^* \nonumber \\&\quad \leqq \sum _{j=0}^{k-1}\int _{-\infty }^\infty \hat{y}'\omega _0^2|\uppsi _{(j)}|^2 \textrm{d}r^*+B(|s|-k)\int _{-\infty }^\infty \frac{\hat{y}'}{C}\omega _0^2|\uppsi _{(k+1)}|^2 \textrm{d}r^*\nonumber \\&\qquad +2\omega _0^2|\uppsi _{(k)}(-\infty )|^2-\int _{-\infty }^\infty 2\hat{y}\operatorname {Re}[\mathfrak {G}_{(k)}\overline{\uppsi _{(k)}'}] \textrm{d}r^*\,. \end{aligned}$$

*A* *K-based energy current.* For some $1< 2R_2^*\leqq R_3^*$ to be fixed, let $0\leqq \chi _2\leqq 1$ be a smooth cutoff function which is identically 0 for $r^*\geqq R_3^*$ and identically 1 for $r^*\leqq R_2^*$. If s≠0, suppose that $\mathfrak {G}_{(k)}$ all benefit from improved decay as $r\rightarrow r_+$ so that the Teukolsky–Starobinsky *K* energy current satisfies the properties in Lemma 5.4. Then, application of currents $E_W\chi _1Q^K$ if s=0 and $E_W \chi _1 Q^{W,T}$ otherwise leads to localization errors:

$$\begin{aligned}&\int _{-\infty }^{\infty } E_W \chi _1'\omega _0\operatorname {Im}[\uppsi _{(k)}'\overline{\uppsi }_{(k)}]\textrm{d}r^*\\&\quad \leqq \frac{BE_1\omega _0}{R_3^*-R_2^*}\int _{-\infty }^\infty \left[r|\uppsi _{(k)}'|^2 +r^{-1}|\uppsi _{(k)}|^2\right]\mathbb {1}_{[R_2^*,R_3^*]}\textrm{d}r^*\,,\\&\int _{-\infty }^{\infty } E_W \chi _1'\omega _0 Q^{T,W} \textrm{d}r^* \\&\quad \leqq \frac{BE_W\omega _0}{R_3^*-R_2^*}\int _{-\infty }^\infty \sum _{k=0}^{|s|}\left[r^{2(|s|-k)+1}|\uppsi _{(k)}'|^2 +r^{2(|s|-k)-1}|\uppsi _{(k)}|^2\right]\mathbb {1}_{[R_2^*,R_3^*]}\textrm{d}r^*\,, \end{aligned}$$

see Lemma 5.4. Combining the above calculations with Lemma 4.10 and Lemma 6.36(iv), we obtain

6.61 $$\begin{aligned}&\sum _{k=0}^{|s|}E_W\omega _0^2|\uppsi _{(k)}(-\infty )|^2\nonumber \\&\quad \leqq \frac{B(\omega _0)E_W}{R_3^*-R_2^*}\int _{-\infty }^\infty \sum _{k=0}^{|s|}\left[r^{2(|s|-k)+1}|\uppsi _{(k)}'|^2 +r^{2(|s|-k)-1}|\uppsi _{(k)}|^2\right]\mathbb {1}_{[R_2^*,R_3^*]}\textrm{d}r^*\,. \end{aligned}$$

*The* *h*, *y* *and* *T* *currents.* Fix $$R_1^*>1$$ to be large enough that $w^{\prime }<0$ for $$r^*\geqq R_1^*$$ and Lemma 6.36(i) applies with $$R_-^*= R_1^*$$. For some $$1<R_2^*$$ and $$\hat{p}, p\in (0,1)$$ to be fixed, we set

$$\begin{aligned} h_{(k)}=h^\textrm{left}+h_{(k)}^{\textrm{int}}+h_{(k)}^{\textrm{right}}\,, \end{aligned}$$

to be a $C^1$ function where

$$\begin{aligned} h^\textrm{left}&= \hat{p}\omega _0^2 \left[|\hat{y}(R_1^*)|(r^*-R_1^*)+\int _{R_1^*}^{r^*}\hat{y}(r^*) \textrm{d}r^*\right]\mathbb {1}_{[R_1^*,R_2^*]}\,,\\ h_{(k)}^{\textrm{int}}&=h^\textrm{left}(R_2^*)+[w(R_2^*)]^{|s|-k}(h^\textrm{left})'(R_2^*) \int _{R_2^*}^{r^*}w^{-(|s|-k)}\textrm{d}r^*\mathbb {1}_{[R_2^*,R_3^*]}\,, \end{aligned}$$

and $h_{(k)}^\textrm{right}$ is given by (6.44) in Lemma 6.39. For $\tilde{p}\in (0,1)$ and ϵ,p>0 to be fixed, we set $y_{(k)}$

$$\begin{aligned} y_{(k)}&=\frac{\tilde{p}}{(R_3^*)^{2(|s|-k)+1}} \int _{R_3^*/2}^{r^*}h_{(k)}^{\textrm{int}}(x^*)dx^*\mathbb {1}_{[R_3^*/2,R_3^*]} + y_{(k)}^\textrm{right}\,, \end{aligned}$$

where $y_{(k)}^\textrm{right}$ as in (6.45) with $C_{2,(k)}^+$ constants chosen to ensure $y_{(k)}$ is a $C^0$ function.

We fix ϵ>0, and then p>0, as functions of $R_2^*$ and $R_3^*$ so that Lemma 6.39 applies for sufficiently small $\omega _\textrm{low}$ and sufficiently large $R_3^*$. In estimate (6.46) of Lemma 6.39, we take $\chi _2=1$ for $r^*\geqq R_3^*$ and $\chi _2=0$ for $r^*\leqq R_3^*/2$, and where $E\geqq 1+2y(\infty )$. We turn to estimating the last seven lines of (6.46) in the region $r^*\in [R_1^*, R_3^*]$.

Let us fix $\tilde{p}$ to be sufficiently small, as a function of $R_3^*$ is sufficiently large, ithat

$$\begin{aligned} y'_{(k)}(\omega ^2-\mathcal {V}_{(k)})\mathbb {1}_{(-\infty ,R_3^*]}+\frac{3}{4} h_{(k)}(\mathcal {V}_{(k)}-\omega ^2)\geqq \frac{5}{8} h_{(k)}(\mathcal {V}_{(k)}-\omega ^2)\,. \end{aligned}$$

The function $h^\textrm{left}$ was chosen so that $h_{(k)}$ grows for $r^*\in [R_1^*,R_2^*]$ without generating a large error:

$$\begin{aligned} \left(h_{(k)}''+(|s|-k)\frac{w'}{w}h_{(k)}'\right)\mathbb {1}_{[R_1^*,R_2^*]} \leqq \hat{p} \hat{y}'\omega _0^2 \mathbb {1}_{[R_1^*,R_2^*]}\,, \end{aligned}$$

thus, we fix $\hat{p} <\frac{1}{16}$ so that we may absorb the error generated by this term into the left hand side of (6.60). In turn, $h_{(k)}^\textrm{int}$ has the most allowable growth in $r^*$ that leads to no errors from derivatives of $h_{(k)}$,

$$\begin{aligned} \left(h_{(k)}''+(|s|-k)\frac{w'}{w}h_{(k)}'\right) \mathbb {1}_{[R_2^*, R_3^*]}=0\,, \end{aligned}$$

and that it is strong enough to control the error terms in (6.61). Since

$$\begin{aligned} h^\textrm{left}(R_2^*)&= \hat{p} \omega _0^2[|\hat{y}(R_1^*)|-|\hat{y}(R_2^*)|]R_2^*\\&\quad \times \left[1-\frac{R_1^*}{R_2^*}\left(1-\frac{|\hat{y}(R_2^*)|}{|\hat{y}(R_1^*)|}\right)^{-1}+\left(\frac{|\hat{y}(R_1^*)|}{|\hat{y} (R_2^*)|}-1\right)^{-1}-\frac{\int _{R_1^*}^{R_2^*}\hat{y} (r^*)\textrm{d}r^*}{|\hat{y}(R_1^*)-\hat{y}(R_2^*)|R_2^*}\right], \end{aligned}$$

we can fix $R_2^*$ to be sufficiently large, depending on *C*, compared to $R_1^*$, so that we have (having fixed $\hat{p}$ already)

$$\begin{aligned} b\omega _0^2|\hat{y}(R_1^*)-\hat{y}(R_2^*)|\frac{r^{2(|s|-k)+1}}{(R_2)^{2(|s|-k)+1}}\leqq h_{(k)}^{ \textrm{int}} \leqq B\omega _0^2|\hat{y}(R_1^*)-\hat{y}(R_2^*)|\frac{r^{2(|s|-k)+1}}{(R_2)^{2(|s|-k)+1}}\,. \end{aligned}$$

Thus, we can estimate that

6.62 $$\begin{aligned}&\sum _{k=0}^{|s|-1}E_W\omega _0^2|\uppsi _{(k)}(-\infty )|^2\nonumber \\&\quad \leqq \frac{B(\omega _0)E_W (R_2^*)^{2|s|}}{(R_3^*-R_2^*)|\hat{y}(R_1^*)-\hat{y}(R_2^*)|}\int _{-\infty }^\infty \sum _{k=0}^{|s|}\left[h_{(k)}|\uppsi _{(k)}'|^2 +h_{(k)}(\mathcal {V}_{(k)}-\omega ^2)|\uppsi _{(k)}|^2\right]\mathbb {1}_{[R_2^*,R_3^*]}\textrm{d}r^*\nonumber \\&\quad \leqq \frac{1}{8} \int _{-\infty }^\infty \sum _{k=0}^{|s|}\left[h_{(k)}|\uppsi _{(k)}'|^2 +h_{(k)}(\mathcal {V}_{(k)}-\omega ^2)|\uppsi _{(k)}|^2\right]\textrm{d}r^*\,, \end{aligned}$$

if we assume that $R_3^*$ is sufficiently large compared to $R_2^*$, depending on $\omega _0$, $R_1^*$, *C* and $E_W$.

For the coupling terms generated by $h_{(k)}$, we haveby the assumption that  in the interval where $h_{(k)}$ is supported. On the other hand, in the region $r^*\in [R_1^*,R_3^*]$, the coupling terms generated by the *T* and $y_{(k)}$ currents in the last five lines of (6.46) are controlled byas long as $\omega _\textrm{low}$ is sufficiently small depending on *E*. We also have localization errors associated to the *T* current

$$\begin{aligned}&E\chi _1\omega \operatorname {Im}[\uppsi _{(k)}'\overline{\uppsi _{(k)}}]\leqq \frac{E\omega _\textrm{low}}{\hat{p} \omega _0^2|\hat{y}(R_1^*)-\hat{y}(R_2^*)|} \left(h_{(k)}|\uppsi _{(k)}'|^2+h_{(k)}(\mathcal {V}_{(k)}-\omega ^2)|\uppsi _{(k)}|^2\right)\\&\quad \leqq \frac{1}{8}\int _{-\infty }^\infty \left(h_{(k)}|\uppsi _{(k)}'|^2+h_{(k)}(\mathcal {V}_{(k)}-\omega ^2)|\uppsi _{(k)}|^2\right)\textrm{d}r^*\,, \end{aligned}$$

if $\omega _\textrm{low}$ is chosen to be sufficiently small depending on *E* and $\omega _0$.

We deduce that the last seven lines of (6.46) can be controlled byThus, from Lemma 6.39 and (6.60), we obtain6.63*The boundary terms*. If the boundary term in the last line of (6.63) were absent, then by making (in addition to all previous requirements), *C* and $R_3^*$ sufficiently large depending on $\tilde{a}_0$ and $\omega _\textrm{low}$ sufficiently small depending *E* and $\tilde{a}_0$, we have enough smallness in the bulk terms involving k+1 that, by iterating the estimate for k=0,⋯,|s|, we can drop the bulk terms in $\uppsi _{(j)}$ for j<k from above. Estimate (6.62) allows us to absorb the boundary terms into the left hand side of (6.63), providing a way of finishing the proof. Otherwise, the iteration procedure we have outlined would generate an additional error

$$\begin{aligned}&\sum _{j=0}^{k} B(\varepsilon _\textrm{width},\omega _\textrm{high}, \tilde{a}_0)\omega _0^2 |\uppsi _{(k)}(-\infty )|^2\leqq B(\varepsilon _\textrm{width},\omega _\textrm{high}, \tilde{a}_0)\omega _0^2 \\&\quad \times \left(\sum _{j=0}^{k}\left|\frac{\mathfrak {G}_{(j)}}{w}\right|^2(-\infty )+|\uppsi _{(0)}(-\infty )|^2\right) \end{aligned}$$

to the estimate for $\uppsi _{(k)}$, by Lemma 6.38. Rescaling the currents so that y(∞)=1 then yields that these errors are weighted by a factor smaller than $R_2/R_3$. Thus, choosing $R_3=R_3(\gamma )$ even smaller so that $R_2/R_3\leqq \gamma$ and then making $\omega _\textrm{low}$ small enough depending on γ concludes the proof. □

#### Bounded frequencies with nonzero time frequency

In this section, we will prove Theorem 6.3 in the cases where the frequencies $(\omega ,m,\Lambda )$ are assumed to be bounded and the time frequency, ω, is bounded away from zero. Concretely, we will show

##### Proposition 6.44

Fix $s\in \mathbb {Z}$ and M>0. Then, for all $\tilde{a}_1>0$, for all $\delta _H,\delta _I\in (0,1]$, for all $\omega _\textrm{high}>0$, for all $\varepsilon _\textrm{width}>0$, for all $\omega _\textrm{low}>0$, for all E>0 sufficiently large, for all $R^*>0$ sufficiently large depending on $\delta _H$, $\delta _I$, $\omega _\textrm{high}$, $\varepsilon _\textrm{width}$, $\omega _\textrm{low}$ and $\tilde{\omega }_\textrm{low}$, there exist functions $y_{(k)}$, $\hat{y}_{(k)}$, $\chi _1$ and $\chi _2$ satisfying the satisfying the bounds (6.3) such that, for all $(\omega , m,\Lambda )\in \mathcal {F}_\textrm{int,1}(\varepsilon _\textrm{width},\omega _\textrm{high},\omega _\textrm{low},\tilde{a}_1)$, we have

$$\begin{aligned}&\omega ^2\left|\Psi (\infty )\right|^2+(\omega -m\upomega _+)^2\left|\Psi (-\infty )\right|^2 \\&\qquad + b(\delta _I,\omega _\textrm{low}, \tilde{\omega }_\textrm{low},\tilde{a}_1)\\&\qquad \int _{-\infty }^\infty \frac{\Delta }{r^2}\left[\frac{1}{r^{1+\delta _I}}|\Psi '|^2+\left(\frac{\Lambda }{r^3}+\frac{1}{r^{1+\delta _I}}\right)|\Psi |^2\right]\textrm{d}r^*\\&\quad \leqq B(E,R^*,\omega _\textrm{high}, \varepsilon _\textrm{width},\tilde{a}_1)\int _{-R^*}^{R^*}\sum _{k=0}^{|s|}\left(|\uppsi _{(k)}'|^2+|\uppsi _{(k)}|^2\right) \textrm{d}r^*\\&\qquad -\int _{-\infty }^\infty \left\rbrace 2(y+\hat{y})\operatorname {Re}\left[\mathfrak {G}\overline{\Psi }'\right]-E\left(\chi _1(\omega -m\upomega _+)+\chi _2\omega \right)\operatorname {Im}\left[\mathfrak {G}\overline{\Psi }\right]\right\lbrace \textrm{d}r^*\\&\qquad +B\sum _{k=0}^{|s|-1}\left|\int _{-\infty }^\infty \left\rbrace 2(y+\hat{y})\operatorname {Re}\left[\mathfrak {G}_{(k)}\overline{\uppsi _{(k)}}'\right]\right.\right.\\&\quad \quad \quad \qquad \qquad \qquad \quad \left.\left.-E\left(\chi _1(\omega -m\upomega _+)+\chi _2\omega \right)\operatorname {Im}\left[\mathfrak {G}_{(k)}\overline{\uppsi _{(k)}}\right]\right\lbrace \textrm{d}r^*\right|\,. \end{aligned}$$

If $\uppsi _{(k)}$ are compactly supported in $r^*\in (r_+,\infty )$, we drop the first line in the estimate and the dependence on $R^*$. If $a\leqq \tilde{a}_0<M$ with $\tilde{a}_0$ sufficiently small depending on $\delta _H$, $\delta _I$, $\omega _\textrm{high}$, $\varepsilon _\textrm{width}$, $\omega _\textrm{low}$ and *E*, we drop the first and third lines in the right hand side.

##### Proposition 6.45

Fix $s\in \mathbb {Z}$ and M>0. Then, for all $\tilde{a}_1>0$, for all $\delta _H\in (0,1)$ and $\delta _I\in (0,1]$, for all $\omega _\textrm{high}>0$, for all $\varepsilon _\textrm{width}>0$, for all $\omega _\textrm{low}, \tilde{\omega }_\textrm{low}>0$, for all E>0 sufficiently large, for all $R^*>0$ sufficiently large depending on $\delta _H$, $\delta _I$, $\omega _\textrm{high}$, $\varepsilon _\textrm{width}$, $\omega _\textrm{low}$ and $\tilde{\omega }_\textrm{low}$, for all $\tilde{a}_1>0$ sufficiently small depending on $\delta _H$, $\delta _I$, $\omega _\textrm{high}$, $\varepsilon _\textrm{width}$, $\omega _\textrm{low}$ and $\tilde{\omega }_\textrm{low}$, there exist functions $y_{(k)}$, $\hat{y}_{(k)}$, $\chi _1$ and $\chi _2$ satisfying the satisfying the bounds (6.3) such that, for all $(\omega , m,\Lambda )\in \mathcal {F}_\textrm{int,2}(\varepsilon _\textrm{width},\omega _\textrm{high},\omega _\textrm{low},\tilde{\omega }_\textrm{low},\tilde{a}_1)$, we have

$$\begin{aligned}&\omega ^2\left|\Psi (\infty )\right|^2+(\omega -m\upomega _+)^2\left|\Psi (-\infty )\right|^2 \\&\qquad + b(\delta _H,\delta _I,\omega _\textrm{low},\tilde{\omega }_\textrm{low})\int _{-\infty }^\infty \frac{\Delta }{r^2}\left[\left(1-\frac{M}{r}\right)^{\delta _H-1}\frac{1}{r^{1+\delta _I}}|\Psi '|^2\right.\\&\qquad \left.+\left(\frac{r-M}{r^4}\Lambda +\frac{(1-Mr^{-1})^{\delta _H-1}}{r^{1+\delta _I}}\right)|\Psi |^2\right]\textrm{d}r^*\\&\quad \leqq B(E,R^*,\omega _\textrm{high}, \varepsilon _\textrm{width})\int _{-R^*}^{R^*}\sum _{k=0}^{|s|}\left(|\uppsi _{(k)}'|^2+|\uppsi _{(k)}|^2\right) \textrm{d}r^*\\&\qquad -\int _{-\infty }^\infty \left\rbrace 2(y+\hat{y})\operatorname {Re}\left[\mathfrak {G}\overline{\Psi }'\right]-E\left(\chi _1(\omega -m\upomega _+)+\chi _2\omega \right)\operatorname {Im}\left[\mathfrak {G}\overline{\Psi }\right]\right\lbrace \textrm{d}r^*\\&\qquad +B\sum _{k=0}^{|s|-1}\left|\int _{-\infty }^\infty \left\rbrace 2(y+\hat{y})\operatorname {Re}\left[\mathfrak {G}_{(k)}\overline{\uppsi _{(k)}}'\right]\right.\right.\\&\qquad \qquad \qquad \qquad \qquad \quad \left.\left.-E\left(\chi _1(\omega -m\upomega _+)+\chi _2\omega \right)\operatorname {Im}\left[\mathfrak {G}_{(k)}\overline{\uppsi _{(k)}}\right]\right\lbrace \textrm{d}r^*\right|\,. \end{aligned}$$

If $\uppsi _{(k)}$ are compactly supported in $r^*\in (r_+,\infty )$, we drop the first line in the estimate and the dependence on $R^*$.

We briefly describe our strategy to prove Propositions 6.44 and 6.45. The result we seek will be significantly easier to prove than the analogous ones in the previous sections, as we are not trying to close estimates for Ψ: we allow an error in a bounded, but potentially quite large, $|r^*|$ region.

We can exploit the fact that $\omega \ne 0$ in $\mathcal {F}_\textrm{int}$ by introducing a *y* current in the large *r* region; this gives us control of bulk terms up to a boundary term error. To control the boundary term, we introduce a localized *T* Killing energy current, with the localization errors (and coupling errors if s≠0) constrained to the bounded $|r^*|$ region. We emphasize that, unlike in the previous sections, we make no attempt to control these errors! In an entirely similar fashion, if $\omega -m\upomega _+\ne 0$, then a *y* current in the $r\rightarrow r_+$ region coupled to a localized *K* Killing current can yield control of boundary and bulk terms in a region of very negative $r^*$ at the cost of some errors in a bounded $|r^*|$ region. If $\omega -m\upomega _+$ is very small then the *y* current near $r\rightarrow r_+$ may lose control over the zeroth order bulk term. We treat these frequencies only when *a* is assumed to be subextremal ($\mathcal {F}_\textrm{int,1}$); in that case, a Hardy estimate provides control of bulk terms for very negative $r^*$ regardless of the sign and strength of $\omega ^2- \mathcal {V}_{(k)}$ and its derivatives.

In $\mathcal {F}_\textrm{int,1}$, the strategy to obtain estimates in the $r^*\rightarrow -\infty$ is manifestly more involved than for the $r^*\rightarrow \infty$. We note, though, that *y* current built in the large *r* region to exploit the fact that $\omega ^2> 0$ could actually be extended all the way to $r=r_+$ as long as $\mathcal {V}_{(k)}'=-2\mathcal {V}_{(k)}$ is negative up to small errors. To extend the current *y*, we must have it degenerate exponentially as $r\rightarrow r_+$ at a sufficiently fast rate depending on the upper bounds on $(\omega ,m,\Lambda )$. However, this gives us only very weak control over a bounded $|r^*|$ region and the region $r^*\rightarrow -\infty$ especially, which is not enough to absorb the bounded $|r^*|$ energy localization errors except in the case where *a* (and thus superradiance) is taken to be sufficiently small depending on the remaining parameters in the problem.

##### Proof of Propositions 6.44 and 6.45

Let a∈[0,M] and $(\omega ,m,\Lambda )$ be an admissible frequency triple such that $(\omega ,m,\Lambda )\in \mathcal {F}_\textrm{int,2}\cup \mathcal {F}_\textrm{int,3}$, to that is assume all frequency parameters are bounded above in terms of $\varepsilon _\textrm{width}$ and $\omega _\textrm{high}$, that $|\omega |> \omega _\textrm{low}$, and that we are away from the double limit a→M, $\omega \rightarrow 0$. Let $\omega _0:=|\omega -m\upomega _+|>0$. We will apply virial currents of *y* type, as well as energy currents of *T* and *K* type, to the radial ODEs (4.19).

*The* $r^*\rightarrow \infty$* region.* For some $R^*>1$ to be determined and $\delta _I\in (0,1]$, we let

$$\begin{aligned} y=\left(1-\frac{(R^*)^{\delta }}{(r^*)^{\delta _I}}\right)\mathbb {1}_{[R^*,\infty )}\,. \end{aligned}$$

Choosing $R^*$ sufficiently large depending on $\omega _\textrm{high}$, $\varepsilon _\textrm{width}$, $\omega _\textrm{low}$ and $\delta _I$, we have

6.64 $$\begin{aligned} y'\omega ^2-(y\mathcal {V}_{(k)})' \geqq \frac{1}{2}y'\omega ^2\,, \end{aligned}$$

since $$\omega ^2\geqq \omega _\textrm{low}^2>0$$ and $$(y \mathcal {V}_{(k)})'$$ is lower order than $y^{\prime }$ as $$r\rightarrow \infty $$ by Lemma 6.34. For k=0,⋯,|s, applying $$Q^y$$ to the radial ODE (4.19) generates coupling errors by Lemma 5.1. For the coupling with k+1, if k<|s|, we have

$$\begin{aligned}&\int _{-\infty }^\infty 2y(|s|-k)w'\left\rbrace w|\uppsi _{(k+1)}|^2-\left(\omega -\frac{am}{r^2+a^2}\right)\operatorname {Re}\left[\uppsi _{(k+1)}\overline{\uppsi _{(k)}}\right]\right\lbrace \textrm{d}r^* \\&\qquad + \int _{-\infty }^\infty 2y(|s|-k)\frac{4amrw}{(r^2+a^2)}\left\rbrace w\operatorname {Im}\left[\uppsi _{(k+1)}\overline{\uppsi }_{(k)}\right]+ \left(\omega -\frac{am}{r^2+a^2}\right)|\uppsi _{(k)}|^2\right\lbrace \textrm{d}r^*\\&\quad \leqq \int _{-\infty }^\infty \frac{B(\omega _\textrm{high},\varepsilon _\textrm{width},\omega _\textrm{low},\delta _I)}{R^*}y'\omega ^2\left(|\uppsi _{(k)}|^2+(|s|-k)|\uppsi _{(k+1)}|^2\right)\textrm{d}r^*\,, \end{aligned}$$

which can be made small by choosing $R^*$ very large; for the coupling terms with j<k,

6.65 $$\begin{aligned}&\sum _{j=0}^{k-1}2yw\operatorname {Re}\left[\left(c_{s,k,j}^\textrm{id}+imc_{s,k,j}^\mathrm{\Phi }\right)\overline{\uppsi _{(k)}'}\uppsi _{(j)}\right]\nonumber \\&\quad \leqq \frac{1}{2} y|\uppsi _{(k)}'|^2+ B(\omega _\textrm{high},\varepsilon _\textrm{width},\omega _\textrm{low},\delta _I)\sum _{j=0}^{k-1} y'\omega ^2|\uppsi _{(j)}|^2\,. \end{aligned}$$

An application of a *y* current also produces boundary terms which we must control. To do this, for $\chi _2$ a smooth cutoff which is equal to 1 for $r^*\in [R^*,\infty )$ and vanishes for $r^*\leqq R^*-1$, we add $E\chi _2Q^T$ with E≧3. For the errors involving $\uppsi _{(k+1)}$, we again obtain for $r^*\geqq 2R^*$

$$\begin{aligned}&(|s|-k)E\chi _2\omega \left[w'\operatorname {Im}[\uppsi _{(k+1)}\overline{\uppsi _{(k)}}]+\frac{4amrw}{r^2+a^2}|\uppsi _{(k)}|^2\right] \\&\quad \leqq y'\omega ^2\left(\frac{1}{8}|\uppsi _{(k)}|^2 + \frac{B(\omega _\textrm{high},\varepsilon _\textrm{width},\omega _\textrm{low},\delta _I)E^2}{R^*}(|s|-k)|\uppsi _{(k+1)}|^2\right), \end{aligned}$$

where, choosing $R^*$ appropriately, the error terms come with a smallness parameter; and for the errors involving $\uppsi _{(j)}$ with j<k, in $r^*\geqq 2R^*$, we have

$$\begin{aligned} \sum _{j=0}^{k-1}&E\chi _2 w\omega \operatorname {Re}\left[\left(c_{s,k,j}^\textrm{id}+imc_{s,k,j}^\mathrm{\Phi }\right)\uppsi _{(j)}\overline{\uppsi _{(k)}}\right]\\&\quad \leqq \frac{1}{4} y'\omega ^2|\uppsi _{(k)}'|^2+ B(\omega _\textrm{high},\varepsilon _\textrm{width},\omega _\textrm{low},\delta _I)E^2\sum _{j=0}^{k-1} y'\omega ^2|\uppsi _{(j)}|^2\,. \end{aligned}$$

Combining the previous estimates we deduce that if $R^*$ is sufficiently large we have

$$\begin{aligned}&\frac{1}{4}\int _{-\infty }^\infty \left\rbrace y'|\uppsi _{(k)}'|^2+y'\omega ^2|\uppsi _{(k)}|^2\right\lbrace \textrm{d}r^*+\omega ^2|\uppsi _{(k)}(\infty )|^2\\&\quad \leqq E B(\omega _\textrm{high},\varepsilon _\textrm{width},R^*)\int _{-2R^*}^{2R^*}\left[\sum _{j=0}^{k}\left(|\uppsi _{(j)}'|^2+|\uppsi _{(j)}|^2\right)+(|s|-k)|\uppsi _{(k+1)}|^2\right]\textrm{d}r^* \\&\qquad +B(\omega _\textrm{high},\varepsilon _\textrm{width},\omega _\textrm{low},\delta _I)E^2\int _{-\infty }^\infty y'\omega ^2\left[\frac{(|s|-k)}{R^*}|\uppsi _{(k+1)}|^2+\sum _{j=0}^k |\uppsi _{(j)}|^2\right]\textrm{d}r^*\\&\qquad -\int _{-\infty }^\infty \left\rbrace 2y\operatorname {Re}[\mathfrak {G}_{(k)}\overline{\uppsi _{(k)}}']-E\omega \chi _2\operatorname {Im}[\mathfrak {G}_{(k)}\overline{\uppsi _{(k)}}]\right\lbrace \textrm{d}r^*\,. \end{aligned}$$

Making $R^*$ even larger depending on *E*, $\omega _\textrm{high}$, $\omega _\textrm{low}$, $\varepsilon _\textrm{width}$ and $\delta _I$, we can iterate in k=0,⋯,|s| (as in the proofs of Propositions 6.41, 6.42 and 6.43) to obtain

$$\begin{aligned}&\sum _{k=0}^{|s|}\frac{1}{8}\int _{R^*}^\infty \left\rbrace y'|\uppsi _{(k)}'|^2+y'\omega ^2|\uppsi _{(k)}|^2\right\lbrace \textrm{d}r^*+\sum _{k=0}^{|s|}\omega ^2|\uppsi _{(k)}(\infty )|^2\\&\quad \leqq E B(\omega _\textrm{high},\varepsilon _\textrm{width},R^*)\sum _{k=0}^{|s|}\int _{-2R^*}^{2R^*}\left(|\uppsi _{(j)}'|^2+|\uppsi _{(j)}|^2\right)\textrm{d}r^* \\&\qquad +B\sum _{k=0}^{|s|-1}\left|\int _{-\infty }^\infty \left\rbrace 2y\operatorname {Re}[\mathfrak {G}_{(k)}\overline{\uppsi _{(k)}}']-E\omega \chi _2\operatorname {Im}[\mathfrak {G}_{(k)}\overline{\uppsi _{(k)}}]\right\lbrace \textrm{d}r^*\right|\\&\qquad -\int _{-\infty }^\infty \left\rbrace 2y\operatorname {Re}[\mathfrak {G}\overline{\Psi }']-E\omega \chi _2\operatorname {Im}[\mathfrak {G}\overline{\Psi }]\right\lbrace \textrm{d}r^*\,. \end{aligned}$$

*The* $r^*\rightarrow -\infty$ *region.* Comparing statements (i) and (ii) in Lemma 6.34, it is not hard to see that the above construction can be implemented with few modifications at the $r^*\rightarrow -\infty$ end if $|\omega _0|\geqq \tilde{\omega }_\textrm{low}$ and if $a\geqq M-\tilde{a}_1$ with sufficiently small $\tilde{a}_1$ so that it can be used as a smallness parameter in the same way that $R^*$ is.^15^ Thus, by taking

$$\begin{aligned} \hat{y}= -\left(1-\frac{(-R^*)^{\delta _H}}{(-r^*)^{\delta _H}}\right)\mathbb {1}_{(-\infty , -R^*]}\,, \end{aligned}$$

for $\delta _H\in (0,1)$ and $R^*$ sufficiently large depending on $\omega _\textrm{high}$, $\varepsilon _\textrm{width}$, $\delta _H$, $\tilde{\omega }_\textrm{low}$, we have

$$\begin{aligned}&\sum _{k=0}^{|s|}\frac{1}{8}\int _{-\infty }^{-R^*}\left\rbrace \hat{y}'|\uppsi _{(k)}'|^2+\hat{y}'\omega ^2_0|\uppsi _{(k)}|^2\right\lbrace \textrm{d}r^*+\sum _{k=0}^{|s|}\omega _0^2|\uppsi _{(k)}(-\infty )|^2\\&\quad \leqq E B(\omega _\textrm{high},\varepsilon _\textrm{width},R^*)\sum _{k=0}^{|s|}\int _{-2R^*}^{2R^*}\left(|\uppsi _{(k)}'|^2+|\uppsi _{(k)}|^2\right)\textrm{d}r^* \\&\quad \qquad +\sum _{k=0}^{|s|}\int _{-\infty }^{\infty } \left\rbrace 2y\operatorname {Re}[\mathfrak {G}_{(k)}\overline{\uppsi _{(k)}}']-E\omega _0\chi _1 \operatorname {Im}[\mathfrak {G}_{(k)}\overline{\uppsi _{(k)}}]\right\lbrace \textrm{d}r^*\,. \end{aligned}$$

This concludes the proof of Proposition 6.45.

For Proposition 6.44 Let us now turn to the general case $0\leqq a\leqq M$. Lemma 6.34(ii) implies that, if $R^*$ is made sufficiently large depending on $\omega _\textrm{high}$ and $\varepsilon _\textrm{width}$,

6.66 $$\begin{aligned}&\hat{y}'\omega ^2_0-\hat{y} \hat{\mathcal {V}}_{(k)}'-\hat{y}' \hat{\mathcal {V}}_{(k)}\geqq \frac{1}{2}\left(\hat{y}'\omega ^2_0-\hat{y} \hat{\mathcal {V}}_{(k)}'\right)\,,\nonumber \\&\quad -\hat{y} \hat{\mathcal {V}}_{(k)}'-\left(-\hat{y} \Delta \frac{2(3r_+^2-a^2)}{r_+}a m\omega _0\right) \leqq B(\varepsilon _\textrm{width},\omega _\textrm{high})\Delta (r-M)\,, \end{aligned}$$

where, in the first line, we used that the last term on the left hand side is lower order as $r\rightarrow r_+$. To control the right hand side of the second line, we derive a Hardy estimate:

6.67 $$\begin{aligned} \int _{-\infty }^\infty (-\hat{y}) \Delta (r-M)|\uppsi _{(k)}|^2\textrm{d}r^*&\leqq B\int _{-\infty }^\infty w'\hat{y}|\uppsi _{(k)}|^2\textrm{d}r^* \nonumber \\&= -B\int _{-\infty }^\infty \left[\hat{y}' w|\uppsi _{(k)}|^2-2\hat{y}w\operatorname {Re}[\uppsi _{(k)}'\overline{\uppsi _{(k)}}]\right]\textrm{d}r^*\nonumber \\&\leqq \int _{-\infty }^\infty \left[Bw\hat{y}\left(\epsilon ^{-1} \frac{\hat{y}'}{\hat{y}}+\frac{w\hat{y}}{\hat{y}'}\right)|\uppsi _{(k)}|^2+ \epsilon \hat{y}'|\uppsi _{(k)}'|^2\right]\textrm{d}r^* \end{aligned}$$

for any ϵ>0, and hence, if $0\leqq a\leqq M-\tilde{a}_1<M$, we have

6.68 $$\begin{aligned} \int _{-\infty }^\infty (-\hat{y}) \Delta (r-M)|\uppsi _{(k)}|^2\textrm{d}r^*&\leqq \frac{1}{8}\int _{-\infty }^\infty \hat{y}'|\uppsi _{(k)}'|^2\textrm{d}r^* + B(R^*)\int _{-2R^*}^{2R^*}|\uppsi _{(k)}|^2 \textrm{d}r^*\,. \end{aligned}$$

We emphasize the conclusion holds only for $a\leqq M-\tilde{a}_1<M$, and choosing $R^*$ sufficiently large depending on $\tilde{a}_1$ and $\delta _H$, as for general a≦M we cannot absorb the first term on the right hand side of (6.67) by the left hand side. Including the factor r-M on the left hand side above just illustrates the failure of this estimate as a→M.

From now on, we restrict to $a\leqq M-\tilde{a}_1<M$, where not only does (6.68) hold but also it is easy to see that Δ(r-M) can be replaced by a weaker decaying weight such as Δ or $|r^*|^{-4}$. In particular, this means that the first term in the second line of (6.66) obeys the same estimate.

Applying the $\hat{y}$ current generates coupling terms. For the terms in (5.4) involving $\uppsi _{(k+1)}$, we have

$$\begin{aligned}&\int _{-\infty }^\infty 2\hat{y}(|s|-k)w'\left\rbrace w|\uppsi _{(k+1)}|^2-\left(\omega -\frac{am}{r^2+a^2}\right)\operatorname {Re}\left[\uppsi _{(k+1)}\overline{\uppsi _{(k)}}\right]\right\lbrace \textrm{d}r^* \\&\qquad + \int _{-\infty }^\infty 2\hat{y}(|s|-k)\frac{4amrw}{(r^2+a^2)}\left\rbrace w\operatorname {Im}\left[\uppsi _{(k+1)}\overline{\uppsi }_{(k)}\right]+ \left(\omega -\frac{am}{r^2+a^2}\right)|\uppsi _{(k)}|^2\right\lbrace \textrm{d}r^*\\&\quad \leqq \int _{-\infty }^\infty \left(\frac{B(\omega _\textrm{high},\varepsilon _\textrm{width},\delta _H)}{R^*}(-\hat{y})\Delta (r-M)|\uppsi _{(k+1)}|^2+\frac{1}{2} (\hat{y}'\omega _0^2+(-\hat{y})\Delta )|\uppsi _{(k)}|^2\right)\textrm{d}r^*\,, \end{aligned}$$

using the largeness of $R^*$, and for the coupling to j<k transformed variables,

6.69 $$\begin{aligned}&\sum _{j=0}^{k-1}2 \hat{y}w\operatorname {Re}\left[\left(c_{s,k,j}^\textrm{id}+imc_{s,k,j}^\mathrm{\Phi }\right)\uppsi _{(k)}'\uppsi _{(j)}\right]\nonumber \\&\quad \leqq \frac{1}{2} \hat{y}'|\uppsi _{(k)}'|^2+B(\omega _\textrm{high},\varepsilon _\textrm{width},\delta _H)\sum _{j=0}^{k-1}\hat{y} \Delta |\uppsi _{j}|^2\,. \end{aligned}$$

We proceed similarly with the errors arising from applying an energy current $E\chi _1Q^{K}$ with E≧3 and $\chi _2(r^*)=\chi _1(-r^*)$. Putting all of this together, we obtain

$$\begin{aligned}&\frac{1}{4} \int _{-\infty }^{-R^*} \left[\hat{y}'|\uppsi _{(k)}'|^2+(\hat{y}'\omega _0^2 -b(a_0)\hat{y}|r^*|^{-4})|\uppsi _{(k)}|^2\right]\textrm{d}r^* + \omega _0^2|\uppsi _{(k)}(-\infty )|^2\\&\quad \leqq EB(\omega _\textrm{high},\varepsilon _\textrm{width},R^*)\int _{-2R^*}^{2R^*}\left(|\uppsi _{(k)}|^2+|\uppsi _{(k)}'|^2\right)\textrm{d}r^* \\&\qquad - \int _{-\infty }^{\infty } \left\rbrace 2\hat{y}\operatorname {Re}[\mathfrak {G}_{(k)}\overline{\uppsi _{(k)}}']-E\omega _0\chi _1\operatorname {Im}[\mathfrak {G}_{(k)}\overline{\uppsi _{(k)}}]\right\lbrace \textrm{d}r^*\\&\qquad +E^2B(\omega _\textrm{high},\varepsilon _\textrm{width},\tilde{a}_1)\int _{-\infty }^\infty (-\hat{y})|r^*|^{-4}\left[\frac{(|s|-k)}{R^*}|\uppsi _{(k+1)}|^2+\sum _{j=0}^k |\uppsi _{(j)}|^2\right]\textrm{d}r^*\,, \end{aligned}$$

and, making $R^*$ even smaller depending on $\tilde{a}_1$, $\delta _H$, $\omega _\textrm{high}$, $\varepsilon _\textrm{width}$ and *E*, we deduce that

$$\begin{aligned}&\frac{1}{8} \sum _{k=0}^{|s|}\int _{-\infty }^{-R^*} \left[\hat{y}'|\uppsi _{(k)}'|^2+(\hat{y}'\omega _0^2 -b(a_0)\hat{y}|r^*|^{-4})|\uppsi _{(k)}|^2\right]\textrm{d}r^* + \sum _{k=0}^{|s|}\omega _0^2|\uppsi _{(k)}(-\infty )|^2\\&\quad \leqq EB(\omega _\textrm{high},\varepsilon _\textrm{width},R^*)x\sum _{k=0}^{|s|}\int _{-2R^*}^{2R^*}\left(|\uppsi _{(k)}|^2+|\uppsi _{(k)}'|^2\right)\textrm{d}r^*\\&\quad \quad + \sum _{k=0}^{|s|-1}\left|\int _{-\infty }^{\infty } \left\rbrace 2\hat{y}\operatorname {Re}[\mathfrak {G}_{(k)}\overline{\uppsi _{(k)}}']-E\omega _0\chi _1\operatorname {Im}[\mathfrak {G}_{(k)}\overline{\uppsi _{(k)}}]\right\lbrace \textrm{d}r^*\right|\\&\quad \quad +\int _{-\infty }^{\infty } \left\rbrace 2\hat{y}\operatorname {Re}[\mathfrak {G}\overline{\Psi }']-E\omega _0\chi _1\operatorname {Im}[\mathfrak {G}\overline{\Psi }]\right\lbrace \textrm{d}r^*\,, \end{aligned}$$

after iterating in k=0,⋯,|s|. This concludes the proof of the main estimate claimed in Proposition 6.44.

*The bounded* $|r^*|$ *region.* As a final step, we construct currents which allow us to control the outstanding bulk term in Ψ in a bounded $|r^*|$ region if $a\leqq \tilde{a}_0$ with $\tilde{a}_0$ sufficiently small. For this estimate, we modify the *y* current above: for some C>1 to be determined, we let

$$\begin{aligned} y(r^*)&=\left(1 -\frac{(R^*)^{\delta _I}}{2(r^*)^{\delta _I}}\right)\mathbb {1}_{(R^*,\infty )}+y(R^*)\exp \left(-C\int _{r(r^*)}^{R^*} \frac{\textrm{d}r}{\Delta } \right)\mathbb {1}_{(-\infty ,R^*]}\,, \end{aligned}$$

and $\hat{y}$ be as above. We take $R^*$ to be sufficiently large depending on $\omega _\textrm{high}$, $\varepsilon _\textrm{width}$, $\omega _\textrm{low}$ and $\delta _I$; then, estimates (6.64), (6.65), (6.66) and (6.69) hold for $r^*\geqq R^*$ and $r^*\leqq -R^*$. Choosing *C* sufficiently large depending on $\omega _\textrm{high}$, $\varepsilon _\textrm{width}$, $\omega _\textrm{low}$, $\delta _I$ and $R^*$, we have

$$\begin{aligned}&\left[y'\omega ^2-(y\mathcal {V}_{(k)})'\right]\mathbb {1}_{[-R^*/2,R^*]}\\&\quad \geqq y'\omega ^2\left(1-\frac{B(\varepsilon _\textrm{width},\omega _\textrm{high},\omega _\textrm{low},\delta _I,R^*)}{C}\right)\mathbb {1}_{[-R^*/2,R^*]}\geqq \frac{1}{4} y'\omega ^2\mathbb {1}_{[-R^*/2,R^*]}\,, \end{aligned}$$

by Lemma 6.33(iii) and, moreover,

$$\begin{aligned} -\int _{-\infty }^{-R^*/2}(y\mathcal {V}_{(k)})'|\uppsi _{(k)}|^2&\leqq \int _{-\infty }^{-R^*} 2 y\mathcal {V}_{(k)}\operatorname {Re}[\uppsi _{(k)}'\overline{\uppsi _{(k)}}]\textrm{d}r^*\\&\leqq \int _{-\infty }^{-R^*}\left[\frac{1}{2}y'|\uppsi _{(k)}'|^2+\frac{1}{4}y'\omega ^2|\uppsi _{(k)}|^2\right]\textrm{d}r^*\,. \end{aligned}$$

Since the *y* and $\hat{y}$ current introduce boundary term errors, we need add localized energy currents $E\chi _1Q^K$ and $E\chi _1Q^T$. We take E≧4, $0\leqq \chi _2\leqq 1$ to be a smooth function which satisfies $\chi _2=1$ for $r^*\geqq R^*$ and $\chi _2=0$ for $r^*\leqq R^*-1$, and $\chi _1=1-\chi _2$.

For these currents, we must estimate the coupling errors. Note that for $r^*\geqq R^*$ and for the $E\chi _1Q^K$ and $\hat{y}$ current in $r^*\leqq -R^*$ we have already estimated these errors in the previous steps. For the additional terms we have

$$\begin{aligned}&\sum _{j=0}^{k-1}2yw\operatorname {Re}\left[\left(c_{s,k,j}^\textrm{id}+imc_{s,k,j}^\mathrm{\Phi }\right)\uppsi _{(j)}\overline{\uppsi _{(k)}'}\right]\mathbb {1}_{(-\infty ,R^*]} \\&\quad \leqq \frac{1}{8} y'|\uppsi _{(k)}'|^2+\frac{1}{16} y'\omega ^2|\uppsi _{(k)}'|^2+ \frac{B(\omega _\textrm{high},\varepsilon _\textrm{width},\omega _\textrm{low},\delta _I)}{C^2}\sum _{j=0}^{k-1} y'\omega ^2|\uppsi _{(j)}|^2\,, \end{aligned}$$

and

$$\begin{aligned}&\sum _{j=0}^{k-1}Ew(\chi _1\omega _0+\chi _2\omega )\operatorname {Im}\left[a\left(c_{s,k,j}^\textrm{id}+imc_{s,k,j}^\mathrm{\Phi }\right)\uppsi _{(j)}\overline{\uppsi _{(k)}'}\right]\mathbb {1}_{(-\infty ,R^*]} \\&\quad \leqq \frac{1}{8} y'|\uppsi _{(k)}'|^2+\frac{1}{16} y'\omega ^2|\uppsi _{(k)}'|^2\\&\qquad + E^2B(\omega _\textrm{high},\varepsilon _\textrm{width},\omega _\textrm{low},\delta _I)(|s|-k+a^2)\sum _{j=0}^{k-1} w |\uppsi _{(j)}|^2\,, \end{aligned}$$

and the coupling to k+1 follows similarly to before.

Combining the previous estimates, we obtain

6.70 $$\begin{aligned}&\int _{-\infty }^\infty \left[\frac{1}{4}(y'+\hat{y})|\uppsi _{(k)}'|^2 + \frac{1}{8}\left(y'\omega ^2+\hat{y}'\omega _0^2-b(\tilde{a}_1)\hat{y}|r^*|^4\right)|\uppsi _{(k)}|^2\right]\textrm{d}r^*\nonumber \\&\qquad + \omega _0^2|\uppsi _{(k)}(-\infty )|^2+\omega ^2|\uppsi _{(k)}(\infty )|^2\nonumber \\&\quad \leqq E^2B(\omega _\textrm{high},\varepsilon _\textrm{width},\omega _\textrm{low},\delta _I,\delta _H)(|s|-k+\tilde{a}_0^2) \sum _{j=0}^{k-1}\int _{-\infty }^\infty (y'\omega ^2+\hat{y}'\omega _0^2\nonumber \\&\qquad +\hat{y} \Delta )|\uppsi _{(j)}|^2\textrm{d}r^* \nonumber \\&\qquad +\frac{E^2B(\omega _\textrm{high},\varepsilon _\textrm{width},\omega _\textrm{low},\delta _H,\delta _I)}{\min \{R^*,C\}}(|s|-k)\int _{-\infty }^\infty (y'\omega ^2-\hat{y}\Delta )|\uppsi _{(k+1)}|^2\textrm{d}r^* \nonumber \\&\quad \qquad + \int _{-\infty }^\infty \left\rbrace 2(y + \hat{y})\operatorname {Re}[\mathfrak {G}_{(k)}\overline{\uppsi _{(k)}}']-E(\chi _1\omega _0+\chi _2\omega )\operatorname {Im}[\mathfrak {G}_{(k)}\overline{\uppsi _{(k)}}] \right\lbrace \textrm{d}r^* \nonumber \\&\quad -\int _{-\infty }^\infty E\frac{am}{2Mr_+}\chi _1'\operatorname {Im}[\uppsi _{(k)}'\overline{\uppsi _{(k)}}]\,\textrm{d}r^* . \end{aligned}$$

For the localization errors, we have

$$\begin{aligned} \int _{-\infty }^\infty E \chi _2' \frac{a m}{2Mr_+} \operatorname {Im}[\Psi '\overline{\Psi }]\textrm{d}r^*&\leqq \int _{-\infty }^\infty \left[\frac{1}{4} y'|\Psi '|^2+\left(\frac{a m E}{Cy(R^*-1)\omega }\right)^2 y'\omega ^2|\Psi |^2\right]\textrm{d}r^*\\&\leqq \int _{-\infty }^\infty \left[\frac{1}{8} y'|\Psi '|^2+\frac{1}{32} y'\omega ^2|\Psi |^2\right]\textrm{d}r^*\,, \end{aligned}$$

where the conclusion follows only if we $a\leqq \tilde{a}_0$ with $\tilde{a}_0$ sufficiently small depending on *E*, $\varepsilon _\textrm{width}$, $\omega _\textrm{high}$, *C* and $R^*$.

To conclude the proof there are two possible routes. Making *C* and $R^*$ sufficiently large depending on $\omega _\textrm{high}$, $\varepsilon _\textrm{width}$,$\omega _\textrm{low}$, $\delta _I$, $\delta _H$ and *E*, we can iterate the above estimate for k=0,⋯,|s| to obtain

6.71 $$\begin{aligned}&\int _{-\infty }^\infty \left[\frac{1}{8}(y'+\hat{y})|\uppsi _{(k)}'|^2 + \frac{1}{16}\left(y'\omega ^2+\hat{y}'\omega _0^2-b(a_0)\hat{y}|r^*|^4\right)|\uppsi _{(k)}|^2\right]\textrm{d}r^*\nonumber \\&\quad + \omega _0^2|\uppsi _{(k)}(-\infty )|^2+\omega ^2|\uppsi _{(k)}(\infty )|^2\nonumber \\&\quad \leqq \frac{E^2B(\omega _\textrm{high},\varepsilon _\textrm{width},\omega _\textrm{low},\delta _H,\delta _I)}{\min \{R^*,C\}}\int _{-\infty }^\infty (y'\omega ^2-\hat{y}\Delta )|\uppsi _{(k+1)}|^2\textrm{d}r^* \nonumber \\&\qquad + \int _{-\infty }^\infty \left\rbrace 2(y + \hat{y})\operatorname {Re}[\mathfrak {G}_{(k)}\overline{\uppsi _{(k)}}']-E\chi _1\omega _0+\chi _2\omega \operatorname {Im}[\mathfrak {G}_{(k)}\overline{\uppsi _{(k)}}] \right\lbrace \textrm{d}r^*\nonumber \\&\quad \quad + B\sum _{j=0}^{k-1}\left|\int _{-\infty }^\infty \left\rbrace 2(y + \hat{y})\operatorname {Re}[\mathfrak {G}_{(j)}\overline{\uppsi _{(j)}}']-E\chi _1\omega _0+\chi _2\omega \operatorname {Im}[\mathfrak {G}_{(j)}\overline{\uppsi _{(j)}}] \right\lbrace \textrm{d}r^*\right|\,. \end{aligned}$$

On the other hand, we can consider the case k=|s| of (6.70) and apply the transport estimate (5.12) from Lemma 5.5 to estimate the coupling terms in the second line of (6.70). With $c=(r-r_+)/r$ if s>0 and c=-1/r if s<0,

6.72 $$\begin{aligned} \int _{-\infty }^\infty w|\uppsi _{(k)}|^2\textrm{d}r^* \leqq \int _{-\infty }^\infty w|\Psi |^2\textrm{d}r^* \,, \end{aligned}$$

so deduce that, for $\tilde{a}_0$ sufficiently small depending on $\omega _\textrm{high}$, $\varepsilon _\textrm{width}$, $\omega _\textrm{low}$, $\delta _I$ and $\delta _H$ or for s=0, we have

6.73 $$\begin{aligned}&\int _{-\infty }^\infty \left[\frac{1}{8}(y'+\hat{y})|\Psi '|^2 + \frac{1}{16}\left(y'\omega ^2+\hat{y}'\omega _0^2-b(\tilde{a}_1)\hat{y}|r^*|^4\right)|\Psi |^2\right]\textrm{d}r^*\nonumber \\&\qquad +\omega _0^2|\Psi (-\infty )|^2 +\omega ^2|\Psi (\infty )|^2\nonumber \\&\quad \leqq -\int _{-\infty }^\infty \left\rbrace 2(y + \hat{y})\operatorname {Re}[\mathfrak {G}\overline{\Psi }]-E(\chi _1\omega _0+\chi _2\omega )\operatorname {Im}[\Psi '\overline{\Psi }]\right\lbrace \textrm{d}r^*\,. \end{aligned}$$

This concludes the proof. □

### Putting everything together

In this section, we combine the results of the previous sections to establish Theorem 6.3 and Theorem 6.4.

#### Proof of Theorem 6.3

Recall that M>0, $s\in \{0,\pm 1, \pm 2\}$ and $a_0\in (0,M)$ are fixed. We fix the parameters used in the previous sections as follows:

(i) We fix $\beta _1=\beta _1(a_0)$ to be sufficiently small that Lemma 6.14(1) holds, as this is enough to meet the conditions of Propositions 6.21 and 6.22. Then, we choose $\varepsilon _\textrm{width}$ sufficiently small, depending on $a_0$, consistent with the requirements of Lemma 6.9 and of Propositions 6.21, 6.22, 6.25 and 6.29, and with the requirement that $\varepsilon _\textrm{width}^{-1}\geqq s^2+2M|s|$.
(ii) Afterwards, we fix $\beta _2$ to be sufficiently small, depending on $a_0$ and $\varepsilon _\textrm{width}$, that Propositions 6.21 and 6.22 hold; notice that means in particular that $\beta _2\leqq \sqrt{\varepsilon _\textrm{width}}$. We can simultaneously fix $\beta _3\leqq \min \{\beta _1,\varepsilon _\textrm{width}\}$, consistent with the requirements of Lemma 6.9 and Propositions 6.25 and 6.29. We also choose *E* to be sufficiently large, depending on the already fixed $\varepsilon _\textrm{width}$, in accordance with the statements of Propositions 6.21, 6.22, 6.25, 6.28, 6.29, 6.40, 6.41, 6.42, 6.43, 6.44 and 6.45.
(iii) By imposing that $\omega _\textrm{high}\geqq 1$ is sufficiently large in terms of all the remaining parameters, we can now determine $r_0'$ by Lemma 6.16 and then Propositions 6.21, 6.22, 6.25 and 6.29 can be applied; we fix $\omega _\textrm{high}$ so that this is the case. Moreover, from Definition 4.1IV(b), we can ensure that
  $$\begin{aligned} m^2\leqq \Lambda +2|s||a\omega |+s^2\leqq 2\varepsilon _\textrm{width}^{-1}\omega _\textrm{high}^2\,, \end{aligned}$$
  whenever $$|\omega |\leqq \omega _\textrm{high}$$ and $$\Lambda \leqq \varepsilon _\textrm{width}^{-1}\omega _\textrm{high}^2$$.
(iv) The only parameters left to fix are those used to separate the bounded frequency ranges with smallness. We first take $\tilde{a}_0$, $\tilde{a}_1$ and $\omega _\textrm{low}$ to be sufficiently small that Propositions 6.40, 6.42 can be applied. Fixing $\omega _\textrm{low}=\omega _\textrm{low}(\tilde{a}_0)$ as a suitable function allows us to conclude that Propositions 6.41 and 6.43 hold. Fixing $\tilde{a}_1=\tilde{a}_1(\omega _\textrm{low},\tilde{\omega }_\textrm{low})= \tilde{a}_1(\tilde{a}_0,\tilde{\omega }_\textrm{low})$ as a suitable function allows us to conclude that Proposition 6.45 holds. Then, we take $\gamma (\tilde{a}_0):=\max \{\gamma _{1a}(\tilde{a}_0),\gamma _{1b}(\omega _\textrm{low}(\tilde{a}_0)),\gamma _2(\omega _\textrm{low}(\tilde{a}_0))\}$. Finally, with $\omega _\textrm{low}(\tilde{a}_0)$, $\omega _\textrm{high}$ and $\varepsilon _\textrm{width}$ fixed, we choose $R^*=R^*(\tilde{a}_0,\tilde{\omega }_\textrm{low})$ to be sufficiently large so that Propositions 6.44 and Propositions 6.45 applies.

This concludes the proof. □

#### Proof of Theorem 6.4

We need only modify our proof of Theorem 6.3 as follows: we choose $\omega _\textrm{high}$ sufficiently large depending on |*m*| so that, in addition to all other constraints, the hypotheses of Lemma 6.12 hold. □

## Data Availability

Data sharing is not applicable to this paper as no datasets were generated or analyzed during the current study.

## Footnotes

1 We remark that [10] also proves decay of linear perturbations to the full subextremal (|a|<M) Kerr family **conditionally** on energy boundedness and integrated local energy decay estimates for the Teukolsky equation of which our Main Theorem gives the first proof.

2 More precisely, these extreme curvature components are invariant under linearized transformations at the level of coordinates or at the level of the null frame.

3 Ma and Zhang [132] in fact is **conditional** on energy boundedness and integrated local energy decay results, now essentially shown by our Main Theorem. In contrast, [123] is **unconditional** and relies on a strong version of mode stability [12] as well as a general setup [161] and several results [61, 162, 163] from microlocal analysis.

4 We will, in fact, reprove the main theorem of [57] as a particular case of the analysis of (1.2) with $s\in \mathbb {Z}$.

5 Note that, though the separable anzatz can in principle be considered for $\omega \in \mathbb {C}$, it is not clear that solutions to the resulting angular ODE always form a complete basis for the space of suitably regular spin-weighted functions.

6 Though we are considering here that $(\omega ,m,\Lambda )$ are all real (see preceding paragraphs and footnote for the reasons why), in the physics literature, mode stability is often taken to mean that there no nontrivial outgoing solutions of the form (1.6) which are exponentially growing in time, to that is which have $\operatorname {Im}\omega >0$ (see Section 1.2.3).

7 The precise version of (1.8) shown in [156, Chapter I.4] is the **only** black box result that our work assumes.

8 It may also be possible to find additional conservation laws, associated to the Teukolsky equation itself or arising from the entire system of linearized gravitational or electromagnetic perturbations, which hold in physical space, see [89] for a=0.

9 To be more precise, the existence of a maximum, $r_\textrm{max}$, of $\mathcal {V}$ such that $\mathcal {V}(r_\textrm{max})=\omega ^2$ depends on the relative sizes of $\omega ^2$ and Λ; however, the value of such $r_\textrm{max}$ can be shown to be independent of the precise value of Λ.

10 An important source of technical complexity is the requirement to work in an energy space with weights weaker than peeling, see the introduction to our [152] for more details.

11 This notation means that there are constants $\{c_k\}_{k=0}^\infty$ such that for every N≧1, as $r\rightarrow \infty$,

$$\begin{aligned} \upalpha ^{[s],\, a,\,\omega }_{ml,\,\mathcal {I}^+}(r) = e^{i\omega r+ 2iM\omega \log r}\sum _{k=0}^{N}c_kr^{-2\,s-k-1}+O(r^{-2\,s-N-2})\,. \end{aligned}$$

12 Indeed, this is the main (frequency-space) reason why one can expect the transformations described in Section 3.3 to be useful.

13 We have chosen to suppress in the discussion the comparison with the case |a|≪M, but the reader may find it useful to go back to Section 1.3.2 to read about this point.

14 In practice, to simplify the presentation, this approach to $\mathcal {F}_\textrm{comp,2}$ will also be used in .

15 One possible way to ensure this condition holds is to have m=0; in this case, the reader may recall this argument has already been used in the proofs Proposition 6.41 and Proposition 6.42.
